# The SWI/SNF subunit BRG1 affects alternative splicing by changing RNA binding factor interactions with nascent RNA

**DOI:** 10.1007/s00438-022-01863-9

**Published:** 2022-02-20

**Authors:** Antoni Gañez-Zapater, Sebastian D. Mackowiak, Yuan Guo, Marcel Tarbier, Antonio Jordán-Pla, Marc R. Friedländer, Neus Visa, Ann-Kristin Östlund Farrants

**Affiliations:** 1 Department of Molecular Biosciences, The Wenner-Gren Institute, Stockholm University, The Arrhenius Laboratories F4, 106 91 Stockholm, Sweden; 2 Science for Life Laboratory, Department of Molecular Biosciences, The Wenner-Gren Institute, Stockholm University, 106 91 Stockholm, Sweden; 3 Present Address: Center for Genomic Regulation, 08003 Barcelona, Spain; 4 Present Address: Max Planck Institute for Molecular Genetics, Ihnestraße 63-73, 14195 Berlin, Germany; 5 Present Address: Departamento de Bioquímica y Biología Molecular, Facultad de Ciencies Biológicas, Valencia University, C/Dr. Moliner, 50, 46100 Burjassot, Spain

**Keywords:** mRNA alternative splicing, Exon GC content, Chromatin remodelling, SWI/SNF, BRGG1, hnRNPL, hnRNPU, SAM68

## Abstract

BRG1 and BRM are ATPase core subunits of the human SWI/SNF chromatin remodelling complexes mainly associated with transcriptional initiation. They also have a role in alternative splicing, which has been shown for BRM-containing SWI/SNF complexes at a few genes. Here, we have identified a subset of genes which harbour alternative exons that are affected by SWI/SNF ATPases by expressing the ATPases BRG1 and BRM in C33A cells, a BRG1- and BRM-deficient cell line, and analysed the effect on splicing by RNA sequencing. BRG1- and BRM-affected sub-sets of genes favouring both exon inclusion and exon skipping, with only a minor overlap between the ATPase. Some of the changes in alternative splicing induced by BRG1 and BRM expression did not require the ATPase activity. The BRG1-ATPase independent included exons displayed an exon signature of a high GC content. By investigating three genes with exons affected by the BRG-ATPase-deficient variant, we show that these exons accumulated phosphorylated RNA pol II CTD, both serine 2 and serine 5 phosphorylation, without an enrichment of the RNA polymerase II. The ATPases were recruited to the alternative exons, together with both core and signature subunits of SWI/SNF complexes, and promoted the binding of RNA binding factors to chromatin and RNA at the alternative exons. The interaction with the nascent RNP, however, did not reflect the association to chromatin. The hnRNPL, hnRNPU and SAM68 proteins associated with chromatin in cells expressing BRG1 and BRM wild type, but the binding of hnRNPU to the nascent RNP was excluded. This suggests that SWI/SNF can regulate alternative splicing by interacting with splicing-RNA binding factor and influence their binding to the nascent pre-mRNA particle.

**Supplementary Information:**

The online version contains supplementary material available at 10.1007/s00438-022-01863-9.

## Background

Chromatin influences transcription not only at the level of initiation and elongation; RNA processing is also influenced by the chromatin structure and changes are required to establish proper gene expression responses to the environment. Alternative splicing and alternative polyadenylation produce different mature mRNA from the same pre-mRNA, being an important source of the diversity of proteins (Wang et al. 2008; Dyvinge 2018). mRNA processing, such as 5'-capping, splicing and polyadenylation events occur to a large extent co-transcriptionally (Ameur et al. 2011; Tilgner et al. 2012), and are tightly coupled to the transcription machinery and chromatin (reviewed in Shukla and Oberdoerffer 2012; Custódio and Carmo-Fonseca 2016; Saldi et al. 2016). Processing factors and RNA binding factors are recruited by the RNA polymerase II (RNA pol II) and by chromatin during elongation. RNA pol II recruits factors by its C-terminal domain (CTD); the 5'-capping enzymes are recruited by serine 5-phophorylated (Ser5-P CTD) RNA pol II CTD (McCracken et al. 1997; Cho et al. 1997; Moteki and Price 2002). Histone modifications in the gene body recruit chromatin proteins, such as the ATPase CHD1 which binds H3K4me3 at the start of a transcribed region and recruits U2snRNP (Sims et al. 2007). Histone-modifying and chromatin proteins are also recruited to the nascent RNA by endogenous small RNA bound to Argonaute (AGO) (Ameyar-Zazoua et al. 2012; Alló et al. 2014).

The mechanisms involved in the regulation of co-transcriptional alternative mRNA splicing are summarised in two general models: the recruitment and the kinetic model. The recruitment model proposes that the splicing outcome is a combinatorial event that depends on splicing factor recruited to the target exon. In addition to the general splicing machinery, many RNA binding proteins, such as serine-rich proteins (SR proteins) and heterogeneous nuclear ribonucleoproteins (hnRNPs) function as splicing enhancers and silencers (Witten and Ule 2011; De Conti et al. 2013; Lee and Rio 2015). These proteins bind to RNA and promote binding of the general splicing machinery or mask splice sites. The kinetic model postulates that the transcription rate determines the inclusion or skipping of alternative exons; a slow RNA polymerase II gives the splicing machinery more time to recognise splice sites and perform the splicing reaction (Kornblihtt 2007; Kornblihtt et al. 2009; Ip et al. 2011; Naftelberg et al. 2015; Saldi et al. 2016). However, recent studies have shown that the transcription rate must be optimal to achieve a normal set of splice forms (Fong et al. 2017; Saldi et al. 2018). How the transcription rate is established and changed in vivo is poorly understood. It has been proposed that it depends on the phosphorylation state of the RNA pol II and on the modifications of the chromatin template. A higher Ser5-P CTD slows down or even pauses the RNA pol II, allowing time for the splicing machinery to recognise weak splice sites (Batsché et al. 2006; Hirose and Ohkuma 2007; Hsin and Manley 2012; Custódio and Carmo-Fonseca 2016; Harlen et al. 2016; Garavis et al. 2017; Nojima et al. 2018). Furthermore, a number of histone modifications have been shown to localise with alternative exons and regulate transcription rate (Gunderson and Johnson 2009; Luco et al. 2010; Hnilicova et al. 2011; Spain and Govind 2011; Jonkers et al. 2014). It was recently shown that PARP-1 influences alternative splicing by reducing histone H3K4me3 and the nucleosome density creating a road bump that caused pausing of the RNA pol II (Matveeva et al. 2019). The rate has also been associated with histone modifications that recruit different proteins, such as HP1α, which results in a slowdown of the RNA pol II and inclusion of exons (Ionnone and Valcárcel 2013; Zhou et al. 2014; Chen et al. 2018).

The kinetic model and the recruitment model are not mutually exclusive but rather potentiate each other and many factors, such as CHD1 and MRG15, are recruited by histone modifications and connect to splice factors, such as U2snRNPs (Sims et al. 2007; Luco et al. 2010; Pradeepa et al. 2012; Dujardin et al. 2014). Some of the adaptors that promote alternative splicing are chromatin proteins involved in chromatin dynamics. These proteins are usually part of chromatin remodelling complexes and are important to establish specific chromatin states by altering the nucleosome occupancy (Hota and Bruneau 2016; Clapier et al. 2017). It is well known that the ATP-dependent chromatin remodelling SWI/SNF complexes function at the promoters but the mammalian ATPases, BRG1/SMARCA4 and BRM/SMARCA2, have also been implicated in different steps of RNA processing, splicing as well as polyadenylation (Bansché et al. 2006; Ito et al. 2008; Tyagi et al. 2009; Allemand et al. 2016; Yu et al. 2018). Three main SWI/SNF complexes have been identified in mammalian cells, being composed of BRG1 or BRM as the ATPase catalytic subunit, a few core subunits, such as BAF155/SMARCC1, BAF170/SMARCC2 and INI1/SMARCB1 and the signature components BAF200/ARID2 and BAF180/PBMR1 in the PBAF and BAF250/ARID1 in the BAF complex (Hargreaves and Crabtree 2011; Masliah-Planchon et al. 2015; Michel et al. 2018; Mashtalir et al. 2018). A third complex, the non-conventional BAF complex (ncBAF) with only BAF155/SSMARCC1 as core and the specific subunits BRD9 has recently been identified (Michel et al. 2018; Mashtalir et al. 2018). How these complexes contribute to RNA processing is not well understood, but BRM SWI/SNF complexes have been proposed to change the CTD-phosphorylation state of RNA pol II during elongation and thereby change the rate of transcription (Batsché et al. 2006; Ito et al. 2008). The *Drosophila* SWI/SNF complex changes the splicing outcome of a number of transcripts (Tyagi et al. 2009; Waldholm et al. 2011) by altering nucleosome stability (Zraly and Dingwall 2012). SWI/SNF ATPases have also been found to associate with the growing RNP (Tyagi et al. 2009) and to interact with general splicing factors (Dellaire et al. 2002; Ito et al. 2008; Waldholm et al. 2011; Allemand et al. 2016; Yu et al. 2018). Furthermore, the human BRG1 regulates alternative cleavage site choice by degrading the 3’ end processing factor CstF through interaction with BRCA/BARD (Fontana et al. 2017). BRG1 and its *Drosophila* orthologue Brm are also involved in cleavage site choice of mRNA by interacting with members of the cleavage and polyadenylation factor complexes (CPSF) (Yu et al. 2018). However, the mechanisms by which the SWI/SNF ATPases and complexes function in alternative mRNA processing are poorly understood.

In this study, we have performed an RNA-seq transcriptome analysis of C33A cells, a SWI/SNF deficient cell line (Muchardt and Yaniv 1993; Wong et al. 2000; Decristofaro et al. 2001), which exogenously expresses SWI/SNF ATPases, and we have identified a subset of genes whose splicing outcome was affected. Both exon inclusion and skipping of exons were affected by the expression of the ATPases, and approximately half did not require the ATPase activity. The included exons in the BRG1-ATPase-deficient group was signified by high GC content and we focused our study on this group. The splicing activity of BRG1 and BRM on these exons was not attributed to one specific SWI/SNF complex, nor did it correlate with an altered nucleosome density or change in RNA pol II accumulation. Instead, these exons accumulated Ser2-P and Ser5-P CTD and when ATPases associated splicing factors and regulators were recruited. Chromatin IP (ChIP) and Chromatin RNA IP (ChRIP) of the factors hnRNPU, hnRNPL and SAM68 suggest that the SWI/SNF ATPases subsequently contributed to the rearrangement of these RNA factors to the nascent RNA particle (RNP).

## Materials and methods

### Cell culture

Human HeLa and C33A cells (originally from ATCC) were cultured at 37 °C and 5% CO_2_ in DMEM (HyClone) medium supplemented with 10% FBS, 50 U/ml penicillin and 50 μg/ml streptomycin.

### Exogenous expression and knock-down

C33A cells were transiently transfected for the expression of hBRG1 and the ATPase-deficient BRG1 from the pBJ5-BRG1 and pBJ5-BRG1-K798R plasmids, respectively (Khavari et al. 1993). For hBRM and its ATPase-deficient versions, pCG-hBrm and pCG-hBrm-K798R (Muchardt and Yaniv 1993) were used. The pOPRSVI vector was used as control. Plasmids were transfected using Lipofectamin 2000 (Invitrogen) according to the manufacturer’s instructions for 48 h before harvesting. BRG1 and BRM were knocked down in HeLa cells using siRNA. siRNA was transfected using RNAiMAX (Invitrogen) according to the manufacturer’s instructions. For knock-down experiments for hnRNPL, hnRNPU and SAM68, the same cells were transfected 24 h after siRNA transfection with plasmids expressing BRG1, BRM and the mutated ATPases, and were incubated for an additional 48 h before harvesting. SiRNAs for BRG1 (called SMARCA4) and BRM (called SMARCA2) and the RNA binding factors investigated are presented in Supplementary Table S4.

### RNA isolation and cDNA synthesis

RNA was extracted using Tri-reagent (Ambion/ThermoFisher) and treated with DNAse I (ThermoFisher). cDNA was synthesised with SuperScript III (Invitrogen/ThermoFisher) and oligo-dT according to the manufacturer’s instructions.

### qPCR

qPCR was performed using a KAPA SYBR Fast qPCR Kit (KAPABiosystem) in a QIAGEN Rotor-GeneQ system. Primers used are presented in Supplementary Table S5.

### RNA-seq, differential exon inclusion and gene expression analysis

Sequencing of 1 µg of RNA was performed with an Illumina HiSeq 2500, with 50 million reads depth. Reads were mapped with Tophat/2.0.4 to the Human genome assembly, build GRCh37. Gene counts were generated using HTseq/0.6.1 on bam files with duplicates included. Alternative splicing was analysed using MISO (Katz et al. 2010), and exons with a Bayes factor > 10 were considered to be differentially spliced. Exons showing opposite effects in the two replicates or in two different groups were discarded, as were exons with the same 5’ or 3’, and less than 50% of the length of the longest exon form. A given exon was only counted once, even if it was reported more than once in the MISO output. Differential gene expression was determined using DESeq2 with default options, and we considered genes with an adjusted *p* value of > 0.05 to be differentially expressed, using Benjamini–Hochberg correction for multiple hypothesis testing (Ramirez et al. 2016). C33A-expressed exons were determined using FeatureCounts, and exons having a count in both replicates from pOPRSVI transfected cells were considered.

### Principle component analysis

Count data were normalised according to DESeq2’s median of ratios. For this, we calculated ratios between the expression of each genes in each sample and the geometric mean across all samples. The median of these ratios in each sample was used as size factor for normalisation. Next, we identified the most variable genes across all samples. We removed lowly expressed genes (fewer than two normalised reads) and plotted each genes mean expression against the squared coefficient of variation. It is known that these measures are linearly dependent on log-space. Therefore, we performed a linear fit on the log-transformed data to identify the 100 genes with the highest residual distance to said fit. Finally, we performed a principal component analysis on the log-transformed expression data (pseudocount = 1) of these most variable genes.

### Co-immunoprecipitation (Co-IP)

HeLa RNP extract was prepared as described in Tyagi et al. (2009). Briefly, nuclei were sonicated to obtain the chromatin fraction, and the chromatin was treated with RNAse A in PBS to release proteins bound to the nascent RNA (RNP fraction). Total cell extract was prepared by lysing cells in 150 mM NaCl, 20 mM Hepes at pH 7.4 supplemented with 0.5% Triton-X100 and 0.1% DOC. The antibodies used were BRG1 antibody (Östlund Farrants et al. 1997), and the BRM and IgG antibody were from Abcam. Antibodies are presented in Supplementary Table S6.

### Immunoblotting

Cells were lysed in SDS-PAGE Laemli buffer containing 5% 2-mercaptoethanol. Protein extracts were separated by SDS-PAGE and transferred to a PVDF membrane (Millipore). Tubulin was used as a loading control (Abcam) for cell extracts and IgG antibody as negative control for co-IP. Antibodies against hnRNPL, DHX9, SAM68, SAP155 and THOC2 were from Abcam and listed in Table S6.

### Chromatin immunoprecipitation (ChIP)

C33A cells were fixed with 1% formaldehyde for 10 min at room temperature and chromatin extracted as described in Ryme et al. (2009). For RNA regulatory protein 1.5–2% formaldehyde was used (Görnemann et al. 2005). The chromatin was fragmented by sonication to fragments with a mean length of 500 bp. The antibodies used: BRM, SAM68, hnRNPL, hnRNPU, BAF155/SMARCC1, BAF250/ARID1, BAF200/ARID2, and BAF180/PBRM1 were purchased from Abcam, BAF200/ARID2 and BAF180/PBRM1 were from Bethyl Laboratories Inc and BRD9 were from Cell signalling and Anova (Supplementary Table S6). Primers used in the analysis are presented in Supplementary Table S5. The standard was SD, and *p* value calculated according to Student’s *t* test.

### Chromatin RNA immunoprecipitation (ChRIP)

ChRIP was performed according to Vizlin-Hodzic et al. (2011) and Hung et al. (2008), with modifications. In brief, C33A cells were cross-linked by 1% formaldehyde-treated chromatin, sonicated and digested with DNAse (ThermoFisher). RNA was extracted from the immunoprecipitated chromatin with Tri-reagent (Ambion/ThermoFisher) and cDNA was synthesised with SuperScript III (Invitrogen/ThermoFisher) using random primers according to the manufacturer’s instructions. The antibodies used against SAM68, hnRNPL and hnRNPU were purchased from Abcam (Supplementary Table S6). Primers used in the analysis are presented in Supplementary Table S5.

### ChIP-seq data analysis

Published signal (bigwig files) from Nucleosome positioning in K562 cells was downloaded from ENCODE/Stanford/BYU. The files were transferred to the MISSISSIPPI Galaxy server (https://mississippi.snv.jussieu.fr/), lifted to hg19 reference genome when necessary using CrossMap (v0.2.7), and plotted using DeepTools2. The data for the plots were generated with computeMatrix 3.1.2, providing lists of included or skipped exons; 100 bp of exon and and 500 bp upstream and downstream of those regions, 20 bp bin, missing values converted to 0 and mean selected as the statistic. The plot was generated with plotProfile (3.1.2) and used the “add standard error” mode.

### Regulatory regions and RNP motifs

RNP binding motifs present in SWI/SNF-affected exons were determined using RBPmap (Paz et al. 2014) providing the exon coordinates and requesting all Human/Mouse motifs, with a high stringency level and conservation filter switched off.

### GC content and splice site analyses

The sequences of affected exons and 500 bp upstream and downstream were retrieved with the Extract Genomic DNA tool (Galaxy Version 2.2.4). All sequences were aligned with transcription orientation and the average of C or G in each position was calculated. All exons were fitted in 100 bp: for this, the average of C or G was previously calculated for 1% of the total length of the exon. Smoothing was done with a moving average, factor 10 in Excel.

The splice site sequences of affected exons were analysed according to Cui et al. 2017. The trinucleotides marking the 5’ and 3’ splice sites were compared to all exons expressed in C33A.

## Results

### BRG1 and BRM affect the splicing outcome of a subset of genes.

SWI/SNF ATPases and complexes have been shown to affect splicing in both human cell lines and in *Drosophila* (Batsché et al. 2006; Ito et al. 2008; Tyagi et al. 2099; Zraly and Dingwall 2012; Allemand et al. 2016), but only a few genes have been studied. To determine the extent of the effect of SWI/SNF ATPases in splicing, we performed RNA-seq of the polyadenylated transcriptome of C33A cells transfected with either BRG1 or BRM. C33A cells transfected with empty vector (pOPRSVI) was used as control reference, and duplicates of BRG1 or BRM transfected cells were performed. In addition to BRG1-wt and BRM-wt with functional ATPase domains, we transfected cells with BRG1 and BRM vectors harbouring point mutation in the ATPase domain (BRG1-mut and BRM-mut), which renders the proteins devoid of chromatin remodelling activity (Muchardt and Yaniv 1993; Khavari et al. 1993). After 48 h of transfection, RNA was prepared and converted to cDNA using oligo-dT primers. The BRG1 mRNA was detected in the BRG1-wt and BRG1-mut transfected cells at a similar level, and the BRM mRNA was detected in the BRM-wt and BRM-mut transfected cells (Supplementary Figure S1A). Similarly, the proteins were expressed in the respective cells, with the protein levels of BRG1-wt and BRM-wt slightly higher than their mutated version (Supplementary Figure S1A).

**Fig. 1:**
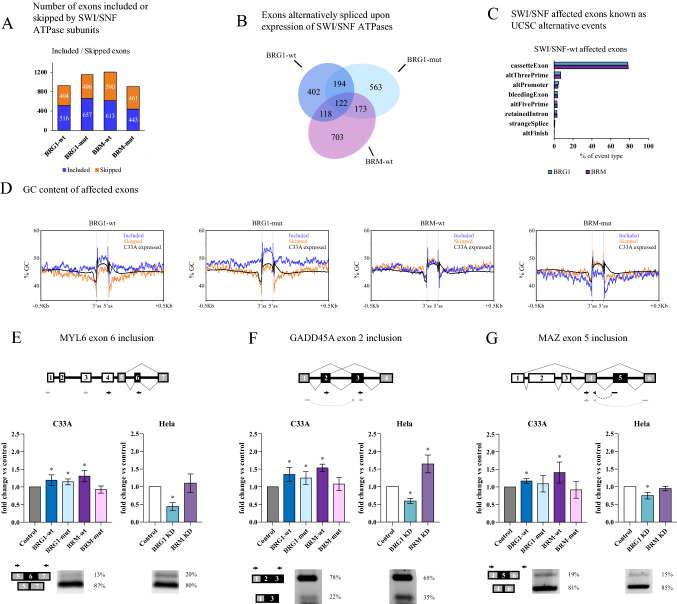
BRG1 and BRM affect alternative splicing of a subset of genes. **A** Number of exons with increased inclusion (blue) or skipped (orange) upon expression of BRG1-wt, BRG-mut, BRM-wt and BRM-mut. **B** Venn diagram showing exons affected by the exogenous expression of BRG1-wt, BRG1-mut and BRM-wt. The pOPRSVI plasmid was used as control. RNA was harvested 48 h after transfection and measured by RNA-seq, where differential exon levels were estimated by the MISO algorithm (Katz et al. 2010). **C** UCSC alternative events classification of BRG1 and BRM-affected exons detected by MISO, represented as percentage of the total amount of affected exons detected. **D** GC content in BRG-wt, BRG1-mut, BRM-wt and BRM-mut at affected exons and the surrounding ± 500 bp regions divided into included (blue) and skipped (orange) exons. Exons are plotted as 100 bp, each bp representing the average GC content of the 1% of the total length for each exon. Exons and the surrounding ± 500 bp regions show GC level at each position. The black line represents the mean GC content of all expressed exons in C33A cells. **E**–**G** Top panels show the scheme of the affected exon in the gene context displaying the affected exon (black), exons taken as reference by MISO to calculate differential inclusion (grey), and constitutive exons (white); arrows show the position of primers used in qPCR with one pair targeting the affected exon (black) and another pair used for normalisation (grey). Middle panels show the differential exon inclusion upon exogenous expression of SWI/SNF ATPases in C33A (left) or knock-down in HeLa (right) measured by qPCR; asterisks denote significant differences compared to the control (*p* value < 0.05, *n* = 5). Bottom panels show the relative basal levels of the two isoforms with included or skipped exons in control cells in C33A (left) and Hela cells (right): arrows show the position of primers used in the PCR

A principal component analysis (PCA) of the duplicate samples from the RNA-seq showed that the first component of the PCA clearly separated the SWI/SNF ATPases from control and ATPase mutant samples (Supplementary S1B). This component explains the majority (66%) of the variance. The RNA-seq samples were then analysed for differentially spliced exons using the MISO algorithm (Katz et al. 2010), and a subset of genes with differentially spliced exons was identified in all ATPase-expressing cells (Supplementary Table S1). BRG1-wt expression resulted in altered splicing of 836 exons, of which 56% exhibited an increased inclusion (Fig. 1A). The ATPase activity was not required for altered splicing in 38% (316 exons) of the affected exons in cells expressing BRG1-wt (Fig. 1B). Expression of BRG1-mut affected splicing of 1052 exons of which 57% exhibiting an increased inclusion (Fig. 1A). The corresponding numbers for BRM-wt and BRM-mut were 1116 exons and 904, respectively, with 49% favouring inclusion in both group (Fig. 1A and B). Only a small number, 195, of the BRM-wt-affected exons were ATPase independent (Supplementary Figure S1C). Most target exons affected by BRM-wt was not affected by BRG1-wt showing that exons were preferentially targeted by one of the ATPases; of the 836 exons affected by BRG1-wt, only 240 were also affected by the expression of BRM-wt (Fig. 1B and Supplementary Figure S1C).

Next, we analysed whether the genes with the affected exons were differentially expressed, since the primary action of SWI/SNF complexes is to regulate chromatin at promoters and enhancers. Analysis performed using DESeq2 (Supplementary Table S2) showed that the expression of 255 genes were affected upon expression of BRG1 in C33A cells, and in contrast to affected exons, the genes affected showed a great overlap with those found in BRM-expressing cells (Supplementary Figure S1D). Interestingly, BRG1-mut expression also affected gene expression, which indicates that genes in human cells are regulated by SWI/SNF in an ATPase-independent manner (Supplementary Table S2), similar to BRM in *Drosophila* cells (Jordán-Pla et al. 2018). Only 102 genes were affected at the levels of both gene expression and splicing, suggesting that SWI/SNF complexes in most cases affect splicing independent of their activity at transcription initiation (Supplementary Table S3).

### Included exons affected by BRG1-mut exhibit a high GC content

To investigate whether the exons affected by BRG1 and BRM had any specific sequence signatures, we examined the kind of splicing event that was preferentially affected by the different ATPases. The MISO analysis identifies internal exons and to assess the different kinds of splicing events used, we performed the UCSC classification of the different exons. The majority of the BRG1- and BRM-affected exons identified were cassette exons (80%), but a small percentage of bleeding exons, exons with poorly defined exon–intron boundaries, and retained introns was also found (Fig. 1C). We examined the exons for sequence signatures, such as GC content (Amit et al. 2012) and splice site preference (Cui et al. 2017; Chen et al. 2019). These analyses revealed that the BRG1-mut included exons exhibited a high GC content, whereas skipped exons had lower GC content similar to most exons expressed (Fig. 1D). This feature was specific to BRG1-mut exons, since BRG1-wt ATPase dependent (BRG1-wt without the BRG1-mut overlapping exons) had a GC content similar to the average expressed exons and did not display a difference in GC content between skipped and included exons (Supplementary Figure S1E). BRM-mut displayed a different pattern with the included exons having lower than average GC content (Fig. 1D). Differences in GC contents between exons and the flanking introns have been suggested to result in an intron–exon definition mechanism, different from the more commonly used exon definition mechanism (Amit et al. 2012; Zhang et al. 2011; Gelfman et al. 2013; Georgomanolis et al. 2016).

The splice site sequence preferences were similar among the SWI/SNF-affected exons, but did differ slightly from the global features from expressed exons in C33A cells. In particular, the 5’splices sites starting with dinucleotides AC and AA were more abundant in SWI/SNF-affected exons (Supplementary Figure S1F). Some differences between the ATPase were also identified, such as a 3’ splice site of CCC in all but BRG1-wt and 3’ TCC in BRM- and BRM-mut-expressing cells. The high content of A- and G-containing sites in SWI/SNF-affected exons was similar to one of the strongest features of cassette exon; weak splice sites which contain the nucleotides AG and GA (Cui et al. 2017).

### BRG1 acts on a subset of exons partly separate from BRM-affected exons

Most studies have focused on the function of BRM in alternative splicing, and it affects the nucleosomal configuration, the RNA pol II rate and the phosphorylation state of RNA polymerase (Batsché et al. 2006; Ito et al. 2008; Tyagi et al. 2099; Allemand et al. 2016). Our results show that BRG1 also affected alternative splicing but not in exactly the same subset of genes as BRM (Fig. 1B and Supplementary Figure S1C). It has previously been shown that BRG1 and BRM cannot substitute for one another on the human CD44 gene (Batsché et al. 2006) and may work through different mechanisms. Our analyses showed that a large number of the BRG1-affected exons were ATPase independent (Fig. 1A) and these included exons had a high GC content. Splicing of these exons may function by a slightly different mechanism from low GC content exons, and to identify differences between BRG1 and BRM, we focused on investigating the mechanism behind the inclusion of exons with a high GC content. BRG1 also associate with several splice factors (Zhao et al. 1998; Dellaire et al. 2002; Allemand et al. 2016). Three genes with increased inclusion in C33A cells expressing BRG1 were selected for further investigation: MYL6, GADD45Aand MAZ. These genes were identified by our genome-wide MISO analysis (Supplementary Table S1) as having a higher level of a cassette exon included in the BRG1-mut and in one other wt-group, BRG1-wt for MYL6 and GADD45A and MAZ for BRM-wt. Sashimi plots of the three genes are shown in Supplementary Figure S1G.

Two alternatively spliced forms of MYL6 were expressed in C33A cells; the one with the cassette exon 6 included was less abundant, 13% of the expressed transcripts (Fig. 1E, bottom panel). The RNA-seq estimated the MYL6 isoform with exon 6 included to 8.5%. qPCR analysis (using primers in Fig. 1E, top panel) validated the higher inclusion level of exon 6 in BRG1-wt-, BRG1-mut- and BRM-wt-expressing C33A cells (Fig. 1E, middle panel) with more than 20% above the level observed in control cells (transfected with empty pOPRSVI vector). BRG1 and BRM were also knocked down by siRNA in HeLa cells to study the effect in a cell expressing both BRG1 (approximately fivefold higher levels than they were exogenously expressed in C33A, Supplementary Figure S1H) and BRM. The ratio between the two MYL6 isoforms in HeLa cells is similar to that in C33A cells; 20% of the form with included exon 6. The inclusion of MYL6 exon 6 was impaired in BRG1 knock-down cells, while BRM knock-down did not change the splicing pattern from control cells (Fig. 1E).

GADD45A exon 2 was identified as being more included by BRG1-wt in the MISO analysis; the splice form was the most abundant one in C33A, with 78% of isoform containing exon 2 in C33A cells (75% in the RNA-seq analysis) (Fig. 1F, bottom panel). qPCR analysis showed that expression of the BRG1-wt, BRG1-mut and BRM-wt increased the inclusion of exon 2 by between 40 and 60% (Fig. 1F, middle panel). BRG1 knock-down in HeLa cells reduced the inclusion of exon 2, while BRM knock-down increased the inclusion level (Fig. 1F). The MAZ transcript with exon 5 included was the low abundant form, only constituting 19%, in C33A cells (8% in the RNA-seq analysis), and 15% in HeLa cells (Fig. 1G, bottom panel). qPCR analysis showed that exon 5 was significantly more included upon BRG1-wt and BRM-wt expression when compared to the transcript without exon 5, by 20% and 40%, respectively (Fig. 1G, middle panel), while BRG1-mut did not increase significantly the inclusion of exon 5 even if the RNA-seq showed that the exon belonged to the BRG1-mut-affected exons. BRG1 knock-down reduced the inclusion of the exon in HeLa cells, whereas BRM knock-down did not induce a changed inclusion rate to control cells. In summary, BRM-mut did not affect the splicing of any of the exons suggesting that the function of BRM is ATP dependent, whereas BRG1 also functions in an ATP-independent manner. Furthermore, the response of the alternative exons of the three genes was slightly different, where MYL6 and GADD45A had exons whose inclusion was influenced by BRG1-mut and the two wild-type ATPases, while MAZ exon 5 required the ATPase activity in our validation experiments and may represent an alternative splicing event which is preferentially ATPase dependent. In addition, the knock-down of BRG1 or BRM in Hela cells suggests that BRG1 is the ATPase responsible for the enhanced inclusion of the exons on these three genes in a more complex SWI/SNF background. On GADD45A, the inclusion rate was increased in BRM knock-down cells, indicating that BRM and BRG1 may function differently on different targets and compete and block the activity of the other ATPase.

### BRG1 and BRM affect splicing as part of the SWI/SNF complex

**Fig. 2:**
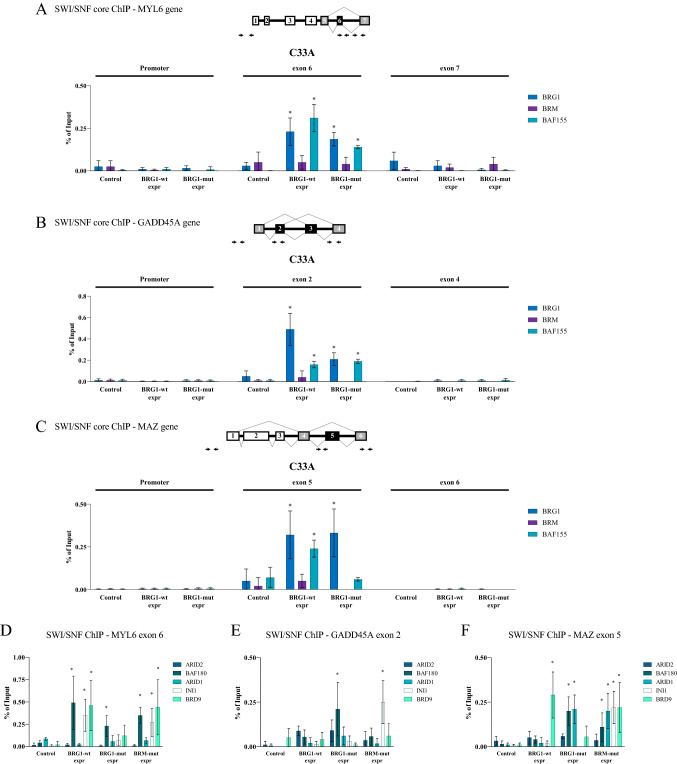
Validation of MYL6, GADD45A and MAZ genes as genes harbouring differentially included exons by BRG1. **A** Schemes of the MYL6 gene with location of the primers used for the ChIP-qPCR are shown at the top. ChIP-qPCR shows the association of BRG1 (dark blue), BRM (purple) and BAF155/SMARCC1 (turquoise) at the promoter, alternative exon 6, and the constitutive exon 7 in control, BRG1-wt- and BRG1-mut-expressing cells. The association is presented as % of input, *n* = 6. *p < 0.05 against control. **B** Schemes of the GADD45A gene with location of the primers used for the ChIP-qPCR are shown at the top. ChIP-qPCR shows the association of BRG1 (dark blue), BRM (purple) and BAF155/SMARCC1 (turquoise) at the promoter, alternative exon 2, and the constitutive exon 4 in control, BRG1-wt- and BRG1-mut-expressing cells. The association is presented as % of input, *n* = 6. **C** Schemes of the MAZ gene with location of the primers used for the ChIP-qPCR are shown at the top. ChIP-qPCR shows the association of BRG1 (dark blue), BRM (purple) and BAF155/SMARCC1 (turquoise) at the promoter, alternative exon 5, and the constitutive exon 6, in control, BRG1-wt- and BRG1-mut-expressing cells. The association is presented as % of input, *n* = 6. **D** ChIP-seq of signature subunits BAF200/ARID2 (blue green) BAF180/PBRM1 (olive green), BAF250/ARID1 (green), INI1/SMACB1 (white), BRD9 (bright green) recruited to MYL6 exon 6 in BRG1-wt-, BRG-mut- and BRM-wt-expressing C33A. The association is presented as % of input, *n* = 4. **E** ChIP-seq of signature subunits BAF200/ARID2 (blue green) BAF180/PBRM1 (olive green), BAF250/ARID1 (green), INI1/SMACB1 (white), BRD9 (bright green) recruited to GADD45A exon 2 in BRG1-wt-, BRG-mut- and BRM-wt-expressing C33A. The association is presented as % of input, *n* = 4. **F** ChIP-seq of signature subunits BAF200/ARID2 (blue green) BAF180/PBRM1 (olive green), BAF250/ARID1 (green), INI1/SMACB1 (white), BRD9 (bright green) recruited to MAZ exon 5 in BRG1-wt-, BRG-mut- and BRM-wt-expressing C33A. The association is presented as % of input, *n* = 4. GADD45A gene at the promoter, affected alternative exon 2 and constitutive exon 4, **F** MAZ gene at the promoter, affected alternative exon 5 and constitutive exon 6. The association is presented as percentage of input

The alternative splicing patterns shown on the selected genes indicated that BRG1 and BRM may use slightly different mechanisms; BRG1 used an ATPase-independent mechanism, whereas BRM used an ATPase-dependent mechanism to achieve the same splicing outcome. This difference prompted us to examine the recruitment of the exogenously expressed ATPases and SWI/SNF subunits to the three alternative exons in C33A cells by ChIP-qPCR. We included MAZ although it was not affected by BRG1-mut in the validation or by BRG1-wt in the RNA sequencing to see if it behaved differently. The expressed ATPases were recruited to the affected exons of MYL6, GADD45A and MAZ (Fig. 2A–C and Supplementary Figures S2A–S2C), also by the BRM-mut protein despite the fact that it was unable to enhance the inclusion of the exons. The binding to the exons was specific to the alternative exons since none of the BRG1-wt, BRG-mut and BRM-wt were recruited to the promoter or to a constitutive exon in the vicinity (Figs. 2A–C and Supplementary Figure S2A). We next investigated whether the ATPases were recruited as part of specific SWI/SNF complexes. The core subunit BAF155/SMACC1 associated with all ATPases at the alternative exons (Figs. 2A–C and Supplementary Figure S2A–C), which supports that the BRG1 and BRM function as part of SWI/SNF complexes on these exons. No specific sub-complex of SWI/SNF (Michel et al., 2018; Mashtalir et al. 2018) was responsible for the intron inclusion since different signature subunits of the preferred complexes were found on all three exons in different constellations (Fig. 2D–F). BRG1-wt and BRM-wt recruited the BAF180/PBRM1and INI1/SMARCB1 in the PBAF complex as well as the ncBAF subunit BRD9 to the MYL6 exon 6, whereas BRG1-mut only recruited BAF180/PBRM1. GADD45A exon 2 displayed another profile with BRG1-mut recruiting BAF180/PBRM1 and BRM associated with INI1/SMARCB1, with BRG1 not strongly associating with any of the specific subunits. MAZ exon 5 had more subunits associated with the exon, in particular BRM had subunits from all three SWI/SNF complexes, BAF180/PBRM1 and BAF250/ARID1 in the PBAF and BAF complex, respectively, and the common subunit INI1/SMARCB1 and the BRD9. BRG1-mut also recruited BAF180/PBRM1 and BAF250/ARID1, while BRG1-wt only associated with BRD9. In conclusion, the role in splicing was not attributed to any specific SWI/SNF complex but rather displayed an exon preference and a preference of the ATPase associating.

### Expression of BRG1 and BRM does not change the chromatin at the affected exons

**Fig. 3:**
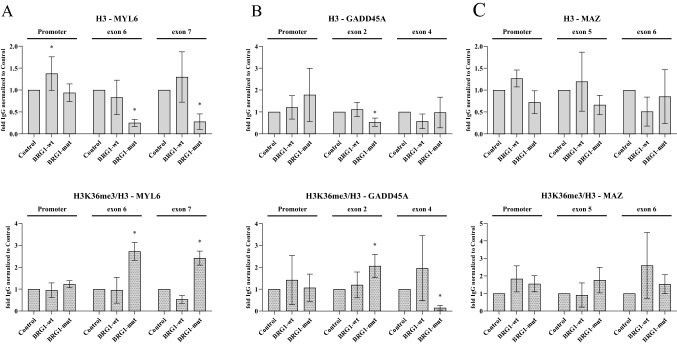
Nucleosome and polymerase density in the affected exons. **A**–**C** ChIP-qPCR using antibodies against histone H3 (top panels) and histone H3K36me3 (bottom panels) targeting **A** the promoter, exon 6 and exon 7 of the MYL6 gene, **B** the promoter, exon 2 and exon 4 of GADD45A, **C** promoter, exon 5 and exon 6 of the MAZ gene in C33A cells expressing BRG1-wt and BRG1-mut. Control is C33A cells transfected with the empty pOPRSVI. Histone H3K36me3 levels are normalised to histone H3 association, and related to the level in control cells. Significantly different values (*p* value < 0.05) are denoted by asterisks (*n* = 3)

BRM and SNR1 (INI1 ortholog) in *Drosophila* cells have been shown to change the nucleosome configuration to change the splicing outcome of a number of transcripts (Zraly and Dingwall 2012). This led us to investigate whether the expression of the ATPases in C33A cells induced changes in the nucleosome density over the selected exons by performing ChIP of histone H3. The histone H3 occupancy did not change in BRG1-wt-expressing cells at the affected exons or over a constitutive exon in the vicinity at any of the genes (Fig. 3A to C). BRG1-mut expression resulted in a lower density over the MYL exon 6 and over the GADD45A exon 2, suggesting that the ATPase-independent function of BRG1 leads to a change in nucleosome density. BRM-wt and BRM-mut displayed lower densities at the MYL6 exon 6 and MAZ exon 5, but no change was observed at GADD45A. In addition, BRG1-mut and BRM-mut expression resulted in a reduced histone occupancy at the constant MYL6 exon 7 (Supplementary Fig. 3A–3B). The BRG1-wt or BRG1-mut did not result in an altered histone H3 occupancy at a non-transcribed region (Supplementary Figure S3D). Taken together, altered histone occupancy over the alternative exons did not correlate with the splicing outcome. BRG1-wt affected the H3 density at the promoter in a similar manner as BRM-wt, but we suggest that this was indirect since the ATPases did not associate with the promoter. We also investigated the exons with high GC content (BRG1-mut) globally for nucleosome occupancy using ENCODE data and BRG1-mut-included exons did not exhibit a positioned nucleosome over the exon in contrast to BRG1-mut-skipped exons and BRG1-wt-skipped and -included exons (Supplementary Figure S3H). A positioned nucleosome at an exon is an important feature for exon definition, but since it is lacking on the high GC-content exons (Kornblihtt et al. 2009; Schwartz and Ast 2010; Amit et al. 2012), other definition mechanisms may operate.

Since the pattern of histone H3 occupancy in the cells expressing the ATPases did not correlate with the splicing outcome, we investigated whether inclusion of the exons associated with changes in histone modifications. Changes in H3K36me3 are associated with splicing (Luco et al. 2010; Guo et al. 2014), and we detected an enrichment of the modification on the low density histone H3 at the exons; in BRG1-mut-expressing cells at MYL6 exon 6 and exon 7 as well as at exon 2 in GADD45A (Fig. 3A to C), a higher H3K36me3 could compensate for the lower nucleosome density. BRM and BRM-mut did not give an enrichment of H3K36me3 on MYL 6 exon 6, although a lower histone occupancy was detected. Instead, an enrichment was found on GADD45A without a decreased histone density (Fig. 3A–C, lower panel, Supplementary Figure S3A to S3C, lower panel). BRG1-mut did not change histone density or H3K36me3 enrichment on MAZ exon 5, while BRM-mut did. BRG1 expression had no effect on the enrichment of H3K36me3. A non-transcribed region 79 kb upstream of the GADD45A gene promoter was used as control and here no changes in histone occupancy or H3K36me3 accumulation were detected (Supplementary Figure S3D).

Other histone modifications have been associated with splicing, H3K4me3, H3K9Ac and H3K27Ac (Iannone and Valcarcel 2013; Enroth et al. 2012; Curado et al. 2015; Hou et al. 2017; Kim et al. 2018) and we also investigated those in cells expressing the ATPases. Only BRG1-wt expression resulted in a change in the enrichment of H3K4me3 at the exons in all three genes; higher in MYL6 exon 6 and GADD45A exon 2, while reduced at MAZ exon 5 (Supplementary Figures S3E–S3G). BRM-wt and BRM-mut affected the H3K4me3 enrichment at GADD45A exon 2, a reduced level in BRM-wt and an increased level in BRM-mut. No changed enrichment could be detected in H3K9Ac or H3K27Ac (Supplementary Figures S3E–S3G). Taken together, the variations in nucleosome density and enrichment of histone modification between cells expressing the different ATPases did not correlate with the splicing outcome, suggesting that the nucleosome state was not the underlying mechanism by which the ATPases influenced splicing decision on this type of exon.

### Expression of the SWI/SNF ATPases does not change the RNA pol II occupancy

**Fig. 4:**
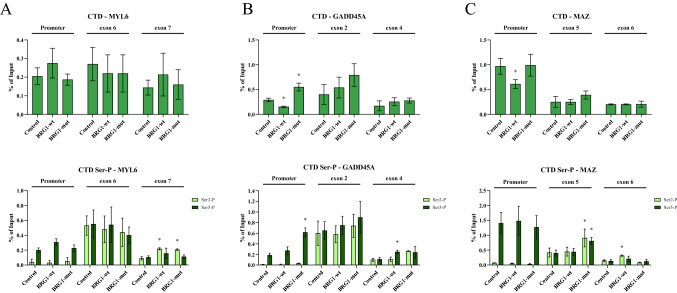
RNA polymerase II density and CTD-phosphorylation in the affected exons. **A**–**C** ChIP-qPCR using antibodies against polymerase II CTD (top panels), and phosphorylated serine 2 (Ser2-P) and serine 5 (Ser5-P) of RNA polymerase II CTD (bottom panels) at **A** MYL6 promoter, exon 6, and exon 7, **B** GADD45A promoter, exon 2 and exon 4, **C** MAZ promoter, exon 5, and exon 6 in C33A cells expressing BRG1-wt, BRG1-mut and control cells transfected with empty vector. The associations are presented as percentage of input. Significantly different values (*p* value < 0.05) are denoted by asterisks (*n* = 5)

In mammalian cells, BRM-containing SWI/SNF complexes alter the RNA pol II CTD phosphorylation level and the RNA pol II density to favour inclusion of exons (Batsché et al. 2006; Ito et al. 2008). We investigated whether the RNA pol II occupancy and phosphorylation state of the CTD were involved in the increased inclusion rate of the alternative exon in the selected genes. No difference was observed in RNA pol II occupancy at the exons between control cell and cells expressing the ATPases (Fig. 4A–3C, top panel, and Supplementary Figure S4A–S4C) (Figs. 3B, top panel). A higher density of RNA pol II compared to the constant exon was exhibited on GADD45A in all cells, including control cells, and was not a result of the expression of the ATPases. This suggests that the RNA pol II rate was not affected by the ATPases. No RNA pol II CTD was detected at the non-transcribed site (Supplementary Figure S4D). The phosphorylations of the CTD at serine 2 (Ser2-P CTD) and at serine 5 (Ser5-P CTD) also play important roles in the dynamics of RNA pol II and splicing (Batsché et al. 2006; Ip et al. 2011; Ito et al. 2008; Nojima et al. 2018). The three promoters exhibited the expected phosphorylation pattern, with high Ser5-P CTD and low to none Ser2-P CTD, indicative of initiating RNA pol II (Fig. 4A–C, lower panels). At the alternative exons, the level of Ser2-P CTD had increased to a level similar to that of the Ser5-P CTD, but no difference in the ratio between the two phosphorylation states could be observed in control cells and the cells expressing the ATPases1 (Fig. 4A to C and Supplementary Figures S4A–S4C, lower panels). The variation in the ratio between Ser2-P CTD and Ser5-P CTD was larger at the constant exons, but only the higher Ser5-P CTD at GADD45A exon 4 and the lower level in MAZ at exon 6 in BRG1-wt cells were significant (*p* = 0.01). Nevertheless, the levels of CTD-phosphorylation were higher at the alternative exons than at the constant exons in all cells, suggesting that it is a defining feature most probably depending on sequence and not chromatin state.

### BRG1 and BRM interact with RNA binding proteins, including splicing regulatory proteins

**Fig. 5:**
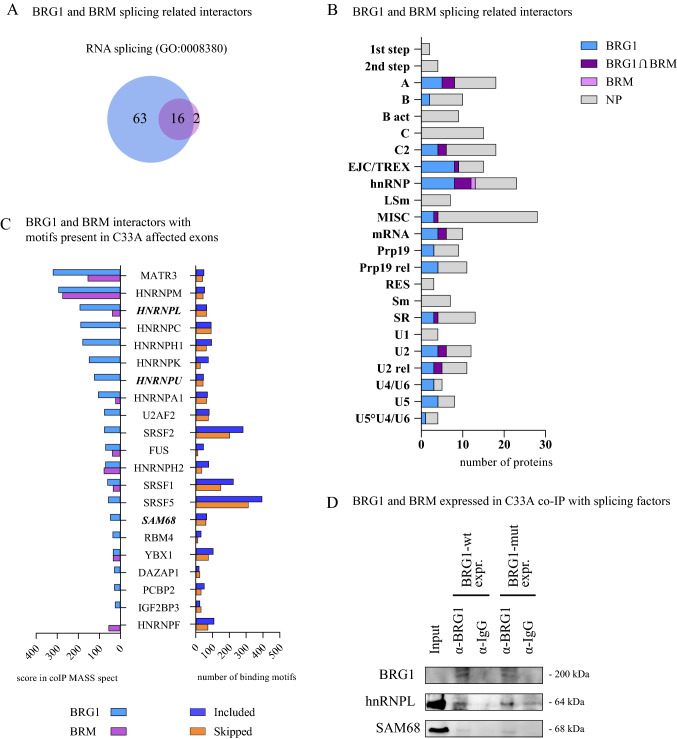
SWI/SNF interactions with RNA binding factor. **A** Venn diagram with the number of RNPs interacting with BRG1 and BRM in the nascent RNP, data from Yu et al. (2018), that are associated with the GO term RNA splicing (GO:0008380). **B** Number of BRG1 and BRM interacting proteins extracted from Yu et al. (2018), known to be involved in splicing [classification from Hegele et al. (2012)]. The total length of the bar corresponds to the total number of proteins classified in each group. BRG1 interacting protein in blue, BRM in pink, both present in BRG1 and BRM in purple, and not present (NP) in any IP in grey. **C** RNA binding proteins interacting with BRG1 or BRM ordered by their score from mass spectrometry (left) with known motifs present in affected exons (right) classified into included (blue) or skipped (orange) exons. The RNA binding proteins selected for detailed mechanistic studies (hnRNPL, hnRNPU and SAM68) are depicted in bold-italics. **D** Immunoblot of immunoprecipitations of hnRNPL and SAM68 from cell extracts (150 mM) of C33A transfected with BRG1-wt or BRG1-mut with a BRG1 antibody

Since the higher CTD-phosphorylation level at the alternative exons did not result in a higher inclusion level, we hypothesised that it defined the exons for further processes and that the SWI/SNF complexes may be responsible for the recruitment of splicing regulatory factors. It has been shown that both BRG1 and BRM interact with several RNA binding proteins and general splicing factors (Zhao et al. 1998; Dellaire et al. 2002; Tyagi et al. 2009; Allemand et al. 2016). To obtain candidates for interaction partners to the ATPases, we analysed mass spectrometry data of BRG1 and BRM interacting proteins in the RNAse treated chromatin fraction (RNP fraction) from HeLa cells (Yu et al. 2018). Several RNA binding proteins were found in the co-immunoprecipitate; an enrichment analysis for the GO term “RNA binding” (GO:0008380) revealed 79 interactors with BRG1 and 18 interactors with BRM, 16 of them identical in the two groups (Fig. 5A). The top significant GO terms from BRG1 and BRM interactors include “mRNA splicing, via spliceosome” and “spliceosome complex” (Supplementary Figure S5A), strongly suggesting a close relation between SWI/SNF ATPases and the splicing machinery. We compared the interactors associated with BRG1 and BRM in the RNP fraction to defined splicing factors and RNA binding factors (Hegele et al. 2012) and the results showed that BRG1 and BRM interacted with factors involved in different steps in the splicing cycle (Fig. 5B). BRG1, and to some extent BRM, interacted mainly with the peripheral protein complexes that are recruited during the assembly of the A or exon definition complex (Hegele et al. 2012; Wongpalee et al. 2016), to Bact/B* complexes (or B-like complex functioning in exon definition complexes), such as the 3'-splice recognition proteins U2AF2 and SF1 in the E complex, the general splicing factors in the U2 snRNP complexes SF3a and SF3b, the U4/U6 snRNP factors Prp31 and Prp3, U5 snRNA component BRR2, Prp8 and Prp6, and the Prp19/CDC5L complex in the Bact/B*. In addition to factors recruited early, proteins involved in later steps, such as the release of the spliceosome by Prp43/DHX15, as well as proteins of the exon junction complex and the THO complex (EJC/TREX) also bound to BRG1. During the assembly of the splicing complex, many splicing regulators are recruited and several of these were found in association with the SWI/SNF ATPases in the nascent RNP; hnRNP proteins constituted a large group that interacted with BRG1 and BRM. Binding motifs of several RNA binding factors were present at the exons affected by BRG1 expression or in the immediate flanking regions (Fig. 5C, right lane). We compared factors whose motifs were found at the exons with the factors found as interactors with BRG1 and BRM in the mass spectrometry (Fig. 5C, left lane) and found that many hnRNPs were well represented. They had both binding motifs and were found to bind to the SWI/SNF ATPases in the mass spectroscopy analyses with high scores.

The interaction of different proteins found in the mass spectrometry were also validated by co-IP with BRG1 and BRM from HeLa RNP fraction. We detected more factors interacting with BRG1 than BRM, in agreement with the mass spectrometry analysis (Yu et al. 2018); the RNA binding factors hnRNPL, DHX9, THOC2, the U2 splice factor SF3B1/SAP155 and the alternative splicing regulator SAM68 interacted with BRG1, while BRM interacted with DHX9 and SAM68 (Supplementary Figures S5B and S5C). The interaction between BRG1 and hnRNPL and SAM68 was also detected in cell extracts (150 mM NaCl) from C33A expressing BRG1-wt and BRG1-mut (Fig. 5D), even though 150 mM NaCl only extracts a minor fraction of the BRG1 protein (Ryme et al. 2009).

### BRG1 recruits RNA binding factors to the affected exons

**Fig. 6:**
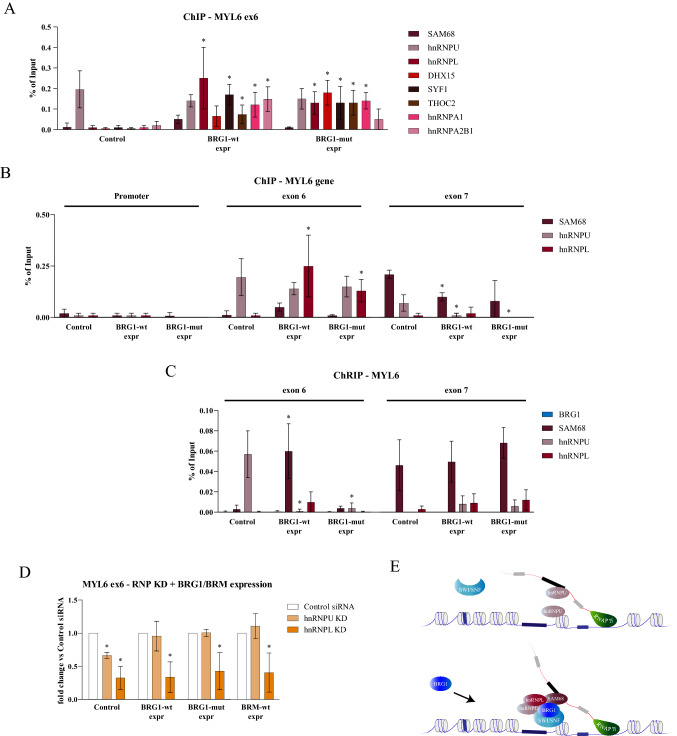
RNA binding proteins are recruited by BRG1 and BRM to the target sites. **A** ChIP-qPCR targeting MYL6 exon 6 in C33A cells expressing BRG1-wt and BRG1-mut was performed with antibodies against SAM68, hnRNPU, hnRNPL, DHX15, SYF1, THOC2, hnRNPA1 and hnRNP2B1; significant changes (*p* value < 0.05) compared to control cells transfected with pOPRSVI empty vector are marked with asterisks (*n* = 6). **B** ChIP was performed with antibodies against SAM68, hnRNPU, hnRNPL and analysed with qPCR with the same primers as in Fig. 2C for MYL6 promoter, exon 6 and exon 7 in cells expressing BRG1-wt and BRG1-mut. Results are presented as percentage of input, and asterisks show significant changes (*p* value < 0.05) compared to control (*n* = 6). **C** ChRIP was performed with antibodies against BRG1, SAM68, hnRNPU, and hnRNPL, and analysed with qPCR for MYL6 exon 6 and exon 7 in cells expressing BRG1-wt and BRG1-mut. Results are presented as percentages of input, and asterisks show significant changes (*p* value < 0.05) compared to control (*n* = 4). **D** Changes in the inclusion of exon 6 in MYL6 after siRNA silencing hnRNPU and hnNRPL in the absence (Control) and presence of BRG1-wt, BRG1-mut and BRM-wt expression. The mRNA values were related to control siRNA for each ATPase expressed and asterisks show significant changes (*p* value < 0.05) compared to control **(n** = 3). **E** Model: expression of BRG1 favours the recruitment of several factors to the site in chromatin, but not to the nascent RNP. hnRNPU associates with both the chromatin and the nascent RNP at the MYL6 exon 6 in control C33A cells, while hnRNPU is removed in BRG1-expressing cells. Instead, SAM68 associates with the nascent RNP in BRG1-wt cells

Next, we investigated whether the changes in splicing outcome observed upon expression of BRG1 and BRM in C33A cells were caused by increased recruitment of RNA binding proteins to the exons investigated. Expression of BRG1-wt and BRG1-mut changed the pattern of factors associating with chromatin at MYL6 exon 6; a number of factors, such as hnRNPL, hnRNPU, hnRNPA1, hnRNPA2B1, DHX15, SYF1 and SAM68, associated with chromatin, although at different levels (Fig. 6A). Only hnRNPU of these factors was present at exon 6 in control C33A cells. Expression of BRM-wt, and particularly BRM-mut, recruited fewer factors to the exon than BRG1 expression (Supplementary Figure S6A). We focused on hnRNPL, hnRNPU and SAM68 and their recruitment to MYL6, where hnRNPL and hnRNPU were recruited to exon 6 by BRG1-wt, BRG-mut and BRM-wt, while SAM68 was only clearly recruited by BRM-wt (Fig. 6B and Supplementary Figure S6B). The factors did not associate with the promoter and SAM68 was present at the exon 7 in all cells, whereas the hnRNPL and hnRNPU bound at low levels. The differences in association patterns in the transfected cell lines, were not caused by changes in expression level of the RNA binding proteins (Supplementary Figure S6C).

The patterns of splicing regulatory factors associating at the alternatively spliced exons of GADD45A and MAZ were slightly different from MYL6, but similar to MYL6 the hnRNPU, hnRNPL and SAM68 associated to the alternative exon to higher levels than to the constitutive exon in the vicinity (Supplementary Figure S6D and S6E). In both GADD45A exon 2 and MAZ exon 5, hnRNPU was recruited by the ATPases from a low level in control cells. SAM68 was present at the exons already in control cells and stayed in the cells expressing BRG1-wt, BRG1-mut and BRM-wt. Only hnRNPL displayed a differential pattern on these exons, with low recruitment levels in BRG1-mut-expressing cells. This suggests that the SWI/SNF ATPases, in particular BRG1, recruit factors directly to chromatin at these alternative exons without affecting the exons in the vicinity and the larger number of factors may contribute to an altered splicing outcome depending on context.

### SWI/SNF ATPases alter the association of factors to the RNP

Many RNA binding proteins associate with both chromatin and the nascent RNA (Chen et al. 2018; Zhou et al. 2014), and we used chromatin–RNA immunoprecipitation (ChRIP) (Mondal et al. 2018; Selth et al. 2011) to assess direct and indirect interactions with RNA and the RNP. No binding of BRG1 or BRM to MYL6 exon 6 or exon 7 in the mRNP was detected in cells expressing the ATPases (Fig. 6C), suggesting that BRG1 and BRM are not directly associated with the RNP but rather with chromatin. Nevertheless, the association of the ATPases altered the interaction between the factors and RNP. Only hnRNPU associated with the nascent mRNP at MYL6 exon 6 in control cells (Fig. 6C) and it was absent on the nascent mRNP in BRG1-wt-, BRG1-mut- and BRM-wt-expressing cells, despite it being associated with chromatin in all cases (Fig. 6C and Supplementary Figure S6C). SAM68 associated with the RNP at the alternative exon 6 only in BRG1-wt-expressing cells and not in BRM-wt-expressing cells where it associated with chromatin. This suggests that the association of the ATPases affects interaction pattern at chromatin and the nascent RNP. On exon 7, no changes were imposed by the expression of the ATPases, and the pattern interacting with RNP reflected the chromatin association with SAM68 being present in all cells (Fig. 6C and Supplementary S6C, bottom panel).

The interaction patterns of the three factors with the RNP at the GADD45A exon 2 and MAZ exon 5 were different from that at the MYL6 exon 6. ChRIP revealed that no association of the factors at GADD45A exon 2 occurred and both hnRNPU and hnRNPL associated with the MAZ exon 5 in control cells (Supplementary Figures S6D and S6E). These patterns were altered in the ATPase-expressing cells, with SAM68 and hnRNPL associated with the RNPs in BRG1-wt cells and very low levels of the factors in BRG1-mut- and BRM-wt-expressing cells. A common feature in all of the ATPase-expressing cells was the exclusion of the binding of hnRNPU to the alternative exon, although it was recruited to chromatin.

### SWI/SNF expression overcomes hnRNPU knock-down

To investigate the role of the interaction between the ATPases, hnRNPU and hnRNPL for the splicing outcome of MYL6 exon 6, we knocked down the factors using siRNAs (Supplementary Figure S6F). MYL6 is an hnRNPL-dependent gene (Cole et al. 2015) and knock-down reduced the inclusion rate of MYL6 exon 6 in control cells and it was not restored in cells expressing the ATPases (Fig. 6D), suggesting that they could not replace the action of hnRNPL. Knock-down of hnRNPU also resulted in a lower inclusion rate in control cells, but the level of inclusion was restored in the cells expressing the ATPases to the higher level (Fig. 6D). This suggests that the ATPases can replace hnRNPU or alter the interaction pattern in the nascent mRNP to allow for other factors to take its place, as shown in the model of MYL6 exon 6 (Fig. 6E).

## Discussion

mRNA alternative splicing is a combinatorial process, depending on a number of regulatory RNA binding proteins in addition to the general splicing machinery, chromatin states and transcription rate. Here, we propose that the SWI/SNF complexes, which are mainly found at the promoter regulating transcription initiation (Masliah-Planchon et al. 2015; Kadoch et al. 2015; Clapier et al. 2017), influence alternative splicing by affecting the interaction of RNA binding proteins with chromatin and the nascent RNA at exons with high GC-content. Expression of the SWI/SNF ATPases BRG1 and BRM in the human SWI/SNF deficient cell line C33A promoted both exon inclusion and exon skipping, emphasising the complexity of splicing events: the splicing outcome depends on specific exon and intron features, as well as on chromatin states and the association of RNA binding protein. The ATPase BRM has been shown to be involved in alternative splicing of specific exons in mammalian cells, favouring inclusion in a process that does not require the ATPase activity (Batsché et al. 2006; Ito et al. 2008). The BRG1 protein binds RNA binding proteins, such as many proteins in snU2 and snU5 spliceosomes (Allemand et al. 2016; Yu et al. 2018), and participates in cleavage and polyadenylation of mRNAs (Yu et al. 2018). We show here that also BRG1 is involved in splicing targeting in part another subset of genes than BRM. Some of these exons did not require the ATPase activity of BRG1 for the splicing event, and the most prominent feature found for the ATPase-independent BRG1 exons is a high GC content surrounded by high GC-content flanking regions compared to exons genome wide. BRG1 expression favoured inclusion of the GC-rich exons in an ATP-independent manner, and BRM could substitute for BRG1 on the exons investigated but only in an ATP-dependent way.

SWI/SNF complexes exist in at least three different constellations with different function (Michel et al. 2018; Mashtalir et al. 2018). In T cells, two of the complexes, ncBAF and PBAF, regulate FOX3 expression differently, one activates and one represses transcription (Loo et al. 2020). No specific subtype of SWI/SNF complex was associated with splicing of the exons with high GC content and our results revealed that even less conventional compositions without signature subunits were present at the exons. We have previously shown that a variation of sub-complexes exist in HeLa cells, also smaller complexes (Ryme et al. 2009). On the investigated genes all ATPases associated with the core subunit BAF155/SMARCC1, suggesting that they function in a SWI/SNF complex. Other subunits may be recruited depending on contexts, such as the BAF180/PBRM1 on MYL6 exon 6. It has been shown that many SWI/SNF component in particular BAF180/PBRM1, BAF200/ARID1 and INI1/SMARCB1 together with BRG1/SMARCA4, associate with the splice factor FUS in liquid–liquid phase separation droplets (Reber et al. 2021). Phase separation mechanisms may contribute to the context specificity at alternative exons and help SWI/SNF subunits and splicing factors to associate with the RNPs.

The histone density or histone modifications patterns at the exons did not show any consistent trends upon expression of the ATPases. The varied response to the ATPases and the fact that the ATPase-deficient BRM-mut also resulted in a change in histone density suggests that the change was not correlated to alternative splicing. This is in contrast to the function of many chromatin proteins which play a role in alternative splicing by affecting the chromatin state and the transcription rate. The nucleosome density (Luco et al. 2010; Zhou et al. 2014; Selth et al. 2011) and the histone modification state at exons (Luco et al. 2019; Chen et al. 2018; Guo et al. 2014; Enroth et al. 2012; Tilgner et al. 2009; Alló et al. 2009) have been proposed to define alternative exons for the splicing machinery and to affect the RNA polymerase phosphorylation level and the transcription rate (Naftelberg et al. 2015; Fong et al. 2017; Nojima et al. 2018; Jonkers et al. 2014; Zhou et al. 2014; Braunschweig et al. 2013; Fu and Ares 2014). It has been shown that proteins, such as PARP1 in the gene body increases inclusion rate by acting as a road-bump, decreasing histone density and altering histone modifications pattern which together slow down the RNA pol II elongation rate (Matveeva et al. 2019). SWI/SNF complexes have been suggested to function by various mechanism. BRM affects alternative splicing by increasing the Ser5-P CTD state of RNA polymerase II and by affecting the elongation rate at alternative exons in HeLa cells (Batsché et al. 2006; Vorobyeva et al. 2013). The higher phosphorylation of RNA pol II at the high GC-content exons did not coincide with a higher RNA pol II density, which suggests a different mechanisms is applied on these exons.

In *Drosophila,* the dSWI/SNF complex, which only have BRM as an ATPase, has been proposed to use a different mechanism from the mammalian BRM (Zraly and Dingwall 2012): it reduces the elongation rate by stalling the RNA pol II at a nucleosome block at exons in ecdysone regulated genes. The SNR1 (the *Drosophila* INI1/SMARCB1 orthologue) is responsible for the stalling of RNA pol II with a relative high level Ser5-P CTD that is suggested to inhibit splicing factors from binding to the RNA, which in turn leads to intron retention (Zraly and Dingwall 2012). To achieve the change in splicing the BRM subunit is required for the release of the nucleosome block and a suggested increase in Ser2-P CTD. The alternative inclusion of cassette exons with high GC content in our study had a high Ser5-P CTD, but also high levels of Ser2-P CTD level, without an enrichment of RNA pol II compared to the constitutive exons. In contrast to the regulation in *Drosophila,* the higher phosphorylation level coincided with the recruitment of splicing factors to the exons without changing the chromatin landscape. Affecting splicing factor recruitment to exons has also been observed in *S. cerevisiae*, where SWI/SNF are involved in splicing of meiotic mRNA by releasing splice factors from down-regulated metabolic genes (Venkataramanan et al. 2017). RNA pol II has been shown to interact with BRG1 (Neish et al. 1997) which could be a way to recruit the SWI/SNF complexes. We propose that SWI/SNF complexes then operate by recruiting splicing regulators and splicing factors to GC-rich SWI/SNF-dependent exons to favour inclusion.

The GC-rich exons resembled a group of exons defined in mammalian and avian genomes with a high GC content, no differential GC content in flanking regions, short introns, and no clear positioned nucleosome at the exon (Amit et al. 2012). This exon architecture is suggested to be recognised by intron definition mechanisms using splicing regulators (Amit et al. 2012; Gelfman et al. 2013). Many splicing regulatory factors and general splicing factors purify with SWI/SNF subunits (Zhao et al. 1998; Dellaire et al. 2002) and purifications of the snRNP U2 spliceosome component also include several SWI/SNF subunits (Makarov et al. 2012; Allemand et al. 2016). We performed an analysis of BRG1 and BRM interacting proteins in the nascent RNP fraction (Yu et al. 2018) and it revealed that BRG1, in particular, interacted with U2 snRNP and U5-U6 snRNP factors that assemble early in the splicing cycle (Lardelli et al. 2010; Makarov et al. 2012; Fu and Ares 2014). In addition to general splicing factors, we also found that BRG1 interacted with many regulatory RNA binding factors, such as hnRNPs and RNA helicases. Recent structural determinations of the spliceosome at different steps show that many rearrangements and compositional changes occur during the splicing cycle and require snRNA, splicing factors and regulatory factors (Agafonov et al. 2016; Bertram et al. 2017a, b; Haselbach et al. 2018; Zhang et al. 2018). We propose that BRG1 and BRM recruit splicing factors and RNA binding proteins to specific exons, and that they may affect the interactions between different splicing regulatory factors in the nascent mRNP, which in turn influence the composition and the activity of the spliceosome and promote changes in splicing outcome. The lower number of splicing regulatory factors recruited by BRM-mut on these GC-rich exons may contribute to the lack of change in splicing outcome.

Alternative splicing does not depend only on one RNA regulatory splicing factor. Instead, splicing is achieved by several different mechanisms using the concurrent actions of a vast number of proteins and RNAs (Shenasa and Hertel 2019). Large-scale network analyses suggest that splicing enhancer proteins promote the assembly of spliceosome sub-complexes, whereas silencing occurs through direct interaction with the RNA (Ulrich and Wahl 2017; Guimarães et al. 2018). Alternative splicing of MYL6 exon 6 is dependent on several factors, such as hnRNPL and U2AF2 (Cole et al. 2015; Sutandy et al. 2018) but not all bind directly to the exon. HnRNPL does not associate directly with exon 6 (Hung et al. 2008; Rossbach et al. 2014; Vu et al. 2013; Shankarling et al. 2014; Cole et al. 2015), which also is supported by publicly available CLIP data. We show here that the ATPases recruited hnRNPL to chromatin, but not to the MYL6 RNP. The knock-down of hnRNPL, however, showed that it was important for the inclusion in an SWI/SNF-independent manner. In hnRNPU knock-down cells, which similar to hnRNPL knock-down cells exhibited a reduced inclusion rate, the expression of BRG1-wt, BRG-mut and BRM-wt restored the higher inclusion level. This may be a result of rearrangements in the spliceosome caused by the SWI/SNF complex associated to the chromatin at exon 6. Combinatorial mechanisms have been proposed in which hnRNPs help to position splicing factors in the spliceosome and to block splice sites (Heinrich et al. 2019; Grillari et al. 2019; Howard et al. 2018), such as ZMAT2, which fine-tune splicing by causing rearrangement in the spliceosome (Tanis et al. 2018). It has also been shown that the binding of RNA binding proteins and splice factors is the major determining factor for the splicing efficiency and alters between cell types (Bedi et al. 2021). Interactions between splicing factors are based on both protein–protein interaction and phase separation mechanisms. It was recently shown that SWI/SNF subunits, BRG1, BAF250/ARID1, BAF180/PBRM1 and INI1/SMARCB1 interact with FUS by phase separation droplet formation (Reber et al. 2021). We speculate that these chromatin remodelling factors influence the spatial association of the general splicing machinery with nascent RNA and rearrange their interactions by recruiting, stabilising, and evicting splicing factors at the exons to determine the splicing outcome (Fig. 6).

Dysregulated expressions of SWI/SNF components are often found in malignant transformation, and may contribute to an altered gene expression that promotes cancer development (Kadoch et al. 2015; Biegel et al. 2014; Mashtalir et al. 2018). In addition, changes in splicing caused by mutations or deletions of snRNP and non-snRNP proteins are tightly connected to malignant transformation and a dysregulated SWI/SNF may also contribute to these splicing changes. Mutations in the splice factor SF3B1/SAP155 affects the formation of BRD9 in the ncBAF complex and this changes transcriptional output and promotes tumour growth (Inoue et al. 2019). We show that BRD9 in the ncBAF complex was involved in the alternative splicing of exons in MYL6 and MAZ. Furthermore, the functions of the different splice variants of the investigated genes have been associated with cancer transformation. MYL6 exon6 is more prevalent in smooth muscle, and the ratio between splice variants are changed during cancer transformation, favouring a splice variant promoting migration (Li et al. 2014; Roberti et al. 2018). GADD45A also exhibits a changed splicing pattern in cancer cells, with the shorter splice form inhibiting cell cycle progression during stress (Zhang et al. 2009; Salvador et al. 2013; Carbonell et al. 2019). The longer MAZ exon 5 variant has been shown to bind more strongly to DNA and inhibit the activation of inflammatory genes induced by the shorter isoform (Ray et al. 2002; Triner et al. 2018). These examples indicate that SWI/SNF complexes affect the balances between splice variants with different functions, and provide an additional way for SWI/SNF complexes to dysregulate targets during cancer progression.

A number of mechanisms operate to regulate the abundance of alternatively spliced exons. These mechanisms affect chromatin structure, transcription rate and the binding of splicing factor and RNA binding proteins. The SWI/SNF complexes have previously been shown to use nucleosome remodelling and transcription rate to influence the splicing outcome, and we show here that SWI/SNF complexes also use an alternative mechanism on a subset of genes. We show that BRG1-containing complexes function through an ATP-independent mechanism to include alternative exons that are defined by a high GC content. These exons have an enrichment of phosphorylated RNA pol II CTD and we postulate that this recruits SWI/SNF ATPases, mainly BRG1, and use a mechanism in which they recruit RNA binding proteins to alternative exons, and change the interaction of regulatory proteins and splice factors in the nascent RNP to promote inclusion.

## Supplementary Information

Below is the link to the electronic supplementary material.

Supplementary file1 (PDF 34751 KB) Supplementary Figure S1: A) The level of mRNA of BRG1 and BRM expressed from the pBJ-BRG1, pBJ-BRG-mut, pCG-BRM-wt and pCG-BRM-mut vectors in C33A (top panel) measured by qPCR using specific primer pairs (see Table S5) and related to the signal in control C33A cells (tranfected with empty pOPRSVI vector). Bottom panels show immunoblots of the BRG1 and BRM protein in BRG1-wt and BRG1-mut expressing cells and the BRM protein in BRM-wt and BRM-mut expressing cells. The immoblot depicts to the left the antibody used to detect the protein in the cell extracts from control, BRG1-wt and BRG1-mut -expressing cells as marked on the top. Molecular weights are marked on the right. B) PCA plot from the 100 most variable genes among the RNA-seq replicates. C) Upset plot showing number of exons affected by the exogenous expression of BRG1-wt, BRG1-mut, BRM-wt and BRM-mut and the intersections between the four groups. D) Venn diagram showing genes affected by the expression of SWI/SNF ATPase subunits. E) GC content in BRG1-wt with the BRG1-mut removed, BRG1-wt with the overlapped BRG1-mut exons. The surrounding +/- 500bp regions were divided into included (blue) and skipped (orange) exons. Exons are plotted as 100 bp, each bp representing the average GC content of the 1% of the total length for each exon. Exons and the surrounding +/-500 bp regions show GC-level at each position. The black line represents the mean GC-content of all expressed exons in C33A cells. F) Splice site trinucleotide at the affected exons in BRG1-wt, BRG-mut, BRM-wt and BRM-mut, presented as 5’ site (red bars) and 3’ site (yellow bars). The trinucleotide abundance was normalised to the presence at all expressed exons in C33A cells. G) Sashimi plot of the affected exons for each of the replicates used for RNA-seq analysis and the percentage of spliced in (PSI) determined by MISO. The PSI is shown for hits with a Bayes Factor > 10 compared to control, others stated as non-significant (n.s). H) mRNA levels of BRG1 and BRM in HeLa cells transfected with either siBRG1, siBRM or control siRNA for 48 h (left panel). Immunoblot of cell extracts prepared at 0.35 M NaCl of Hela cells knocked down with control siRNA, BRG1 siRNA and BRM siRNA (middle panel to the left) and C33A cells transfected with control vector, BRG1-wt and BRG1-mut as marked at the top (middle panel to the right). The membranes were probed for BRG1 and tubulin as marked to the left. Number below show the relative abundance of BRG1 compared to HeLa control cells. Quantification of the endogenous HeLa BRG1 level cell in relation to the exogenously expressed BRG1-wt (n=5) (panel to the right). Supplementary Figure S2: A) ChIP-qPCR shows the association of BRG1 (dark blue), BRM (purple) and BAF155/SMARCC1 (turquoise) at the promoter, alternative exon 6, and the constitutive exon 7 of MYL6 in control, BRM-wt and BRM-mut expressing cells. The association is presented as % of input, n= 6. * p<0.05 against control. B) ChIP-qPCR shows the association of BRG1 (dark blue), BRM (purple) and BAF155/SMARCC1 (turquoise) at alternative exon 2 of GADD45A in control, BRM-wt and BRM-mut expressing cells. The association is presented as % of input, n= 6. * p<0.05 against control. C) ChIP-qPCR shows the association of BRG1 (dark blue), BRM (purple) and BAF155/SMARCC1 (turquoise) at the alternative exon 5 of MAZ in control, BRM-wt and BRM-mut expressing cells. The association is presented as % of input, n= 6. * p<0.05 against control. Supplementary Figure S3: A-D) ChIP-qPCR using antibodies against histone H3 (top panels) and histone H3K36me3 (bottom panels) targeting (A) MYL6 promoter, exon 6 and exon 7, (B) GADD45A exon 2, (C) MAZ exon 5 and (D) a non-coding region 79 kb upstream of GADD45A, in C33A cells expressing BRM-wt and BRM-mut. Histone H3 are related to the association in control cells and significant values (p-value < 0.05) are denoted by asterisks (n = 3). H3K36me3 levels were normalized to H3 before related to the association in control cells and significant values (p-value < 0.05) are denoted by asterisks (n = 3). E) Nucleosome position at BRG1-wt (left panel) and BRG1-mut (right panel) affected exons from publicly available data from the K562 cell line. The nucleosome position over exons is presented as differentially included (blue) or skipped (orange) affected exons and the +/- 0.5 Kb surrounding regions (light colour shows standard error). The black line represents the mean nucleosome position of all C33A expressed exons. F-H) ChIP-qPCR using antibodies against H3K4me3 (top panels), H3K9ac (middle panels) and H3K27ac (bottom panels) in control C33A cells and C33A cells expressing BRG1-wt, BRG1-mut, BRM-wt and BRM-mut, targeting (F) MYL6 exon 6, (G) GADD45A exon 2 and (H) MAZ exon 5. The signal from histone modifications was first normalized to the H3 signal for each treatment, and the association is related to the association in control cells. Asterisks denote significant differences (p-value < 0.05) compared to control (n = 3). Supplementary Figure S4: A-C) ChIP-qPCR using antibodies against polymerase II CTD (top panels), phosphorylated serines 2 (Ser2-P) and serine 5 (Ser5-P) of RNA polymerase II CTD (bottom panels) targeting (A) MYL6 exons 6 and 7, (B) GADD45A exons 2 and 4, and (C) MAZ exons 5 and 6, in C33A cells expressing BRM-wt and BRM-mut. Asterisks denote significant differences (p-value < 0.05) compared to control (n = 5). The values are presented as % of input. D) ChIP-qPCR using antibodies against CTD targeting a non-coding region 79 kb upstream of GADD45A, in C33A cells expressing BRG1-wt and BRG1-mut. Supplementary Figure S5: A) Most enriched GO terms from BRG1 and BRM interacting proteins related to splicing, represented in –log10(p-value). The GO Ontology database, released on 9 August 2018 was used, and the PANTHER Overrepresentation Test (released on 5 December 2017). B) Immunoblots from co-IP performed with BRG1 and BRM antibodies in the RNP fraction of HeLa cells. PVDF membranes were probed with antibodies against proteins detected in the MASS spectroscopy data from Yu et al., (2018). IgG was used as negative control. C) Immunoblot from co-IP using antibodies against SAM68 and membranes probed with BRG1 and BRM antibodies. IgG was used as negative control. Supplementary Figure S6: A) ChIP-qPCR targeting MYL6 exon 6 in cells expressing BRM-wt and BRM-mut and analysed with antibodies against SAM68, hnRNPU, hnRNPL, DHX15, SYF1, THOC2, hnRNPA1 and hnRNP2B1. Significant changes (p-value < 0.05) compared to control are marked with asterisks (n=4). B) ChIP-qPCR performed in cells expressing BRM-wt with antibodies against SAM68, hnRNPU, hnRNPL and analysed with the same primers as in Figure 2A for MYL6 promoter, exon 6 and exon 7 (top panel). Results are presented as percentage of input and asterisks show significant changes (p-value < 0.05) compared to control (n = 6). ChRIP-qPCR was performed in cells expressing BRM-wt with antibodies against SAM68, hnRNPU, and hnRNPL, and analysed with the same primers as in Figure 2D for MYL6 exon 6 (bottom panel). Results are presented as percentage of input and asterisks show significant changes (p-value < 0.05) compared to control (n = 4). C) Immunoblots of cell extracts of using antibodies against hnRNPU, hnRNPL, SAM68 and tubulin, as marked to the left, in C33A cells transfected with empty vector, BRG1-wt, and BRG1-mut, as marked on the top. The molecular weights are depicted on the right hand side. D) ChIP-qPCR was performed in cells expressing BRG1-wt and BRG-mut with antibodies against SAM68, hnRNPU, and hnRNPL, and analysed with the same primers as in Figure 2B for GADD45A promoter, alternative exon 2 and constitutive exon 4 (top panel). Results are presented as percentage of input, and asterisks show significant changes (p-value < 0.05) compared to control (n = 6). ChRIP-qPCR was performed in cells expressing BRG1-wt and BRG1-wt with antibodies against SAM68, hnRNPU, and hnRNPL, and analysed with the same primers as in Figure 2B for GADD45A exon 2. Results are presented as percentage of input, and asterisks show significant changes (p-value < 0.05) compared to control (n = 4). E) ChIP-qPCR was performed in cells expressing BRG1-wt and BRG-mut with antibodies against SAM68, hnRNPU, and hnRNPL, and analysed with the same primers as in Figure 2C for the MAZ promoter, alternative exon 2 and constitutive exon 4 (top panel). Results are presented as percentage of input, and asterisks show significant changes (p-value < 0.05) compared to control (n = 6). ChRIP-qPCR was performed in cells expressing BRG1-wt and BRG1-wt with antibodies against SAM68, hnRNPU, and hnRNPL, and analysed with the same primers as in Figure 2C for MAZ exon 5. Results are presented as percentages of input, and asterisks show significant changes (p-value < 0.05) compared to control (n = 4). F) Immunoblots showing the efficiency of knock-downs of hnRNPU and hnRNPL, 3 days after transfection of siRNA. Tubulin was used as loading control. The size in KDa is indicated to the right

## Data Availability

The RNA-seq data have been submitted to GEO Submission with the access no (GSE140474) [NCBI tracking system #20437516]. The Mass-spec data are available through the reference Yu et al. 2018.

## Abbreviations

SWI/SNF: Switch/sucrose non-fermenting
BRG1: Brahma related gene 1
BAF155: BRG1 associated factor of 155 kDa
INI/SNF5: Integrase interactor 1/sucrose non-fermenting
hnRNP: Heterogenous nuclear RNP
Sam68: Src-associated substrate in mitosis of 68 kDa
DHX15: DEAH-box helicase 15
THOC: THO/TREX (transcription-export) complex
SYF1: Synthetic lethal with cdc forty 1
MAZ: MYC associated zinc finger protein
MYL6: Myosin light chain 6
GADD45A: Growth arrest and DNA-damage-inducible protein
ChIP: Chromatin immunoprecipitation
ChRIP: Chromatin-RNA immunoprecipitation
