# Supplementary material for: The SWI/SNF subunit BRG1 affects alternative splicing by changing RNA binding factor interactions with nascent RNA
Source: Mol Genet Genomics. 2022 Feb 20;297(2):463–84. doi: 10.1007/s00438-022-01863-9 (PMC8960663; doi:10.1007/s00438-022-01863-9)
Supplement: Supplementary file 1 — Supplementary file1 (PDF 34751 KB) Supplementary Figure S1: A) The level of mRNA of BRG1 and BRM expressed from the pBJ-BRG1, pBJ-BRG-mut, pCG-BRM-wt and pCG-BRM-mut vectors in C33A (top panel) measured by qPCR using specific primer pairs (see Table S5) and related to the signal in control C33A cells (tranfected with empty pOPRSVI vector). Bottom panels show immunoblots of the BRG1 and BRM protein in BRG1-wt and BRG1-mut expressing cells and the BRM protein in BRM-wt and BRM-mut expressing cells. The immoblot depicts to the left the antibody used to detect the protein in the cell extracts from control, BRG1-wt and BRG1-mut -expressing cells as marked on the top. Molecular weights are marked on the right. B) PCA plot from the 100 most variable genes among the RNA-seq replicates. C) Upset plot showing number of exons affected by the exogenous expression of BRG1-wt, BRG1-mut, BRM-wt and BRM-mut and the intersections between the four groups. D) Venn diagram showing genes affected by the expression of SWI/SNF ATPase subunits. E) GC content in BRG1-wt with the BRG1-mut removed, BRG1-wt with the overlapped BRG1-mut exons. The surrounding +/- 500bp regions were divided into included (blue) and skipped (orange) exons. Exons are plotted as 100 bp, each bp representing the average GC content of the 1% of the total length for each exon. Exons and the surrounding +/-500 bp regions show GC-level at each position. The black line represents the mean GC-content of all expressed exons in C33A cells. F) Splice site trinucleotide at the affected exons in BRG1-wt, BRG-mut, BRM-wt and BRM-mut, presented as 5’ site (red bars) and 3’ site (yellow bars). The trinucleotide abundance was normalised to the presence at all expressed exons in C33A cells. G) Sashimi plot of the affected exons for each of the replicates used for RNA-seq analysis and the percentage of spliced in (PSI) determined by MISO. The PSI is shown for hits with a Bayes Factor > 10 compared to control, others stated as non-si [file 438_2022_1863_MOESM1_ESM.pdf]

Supplementary Figure 1

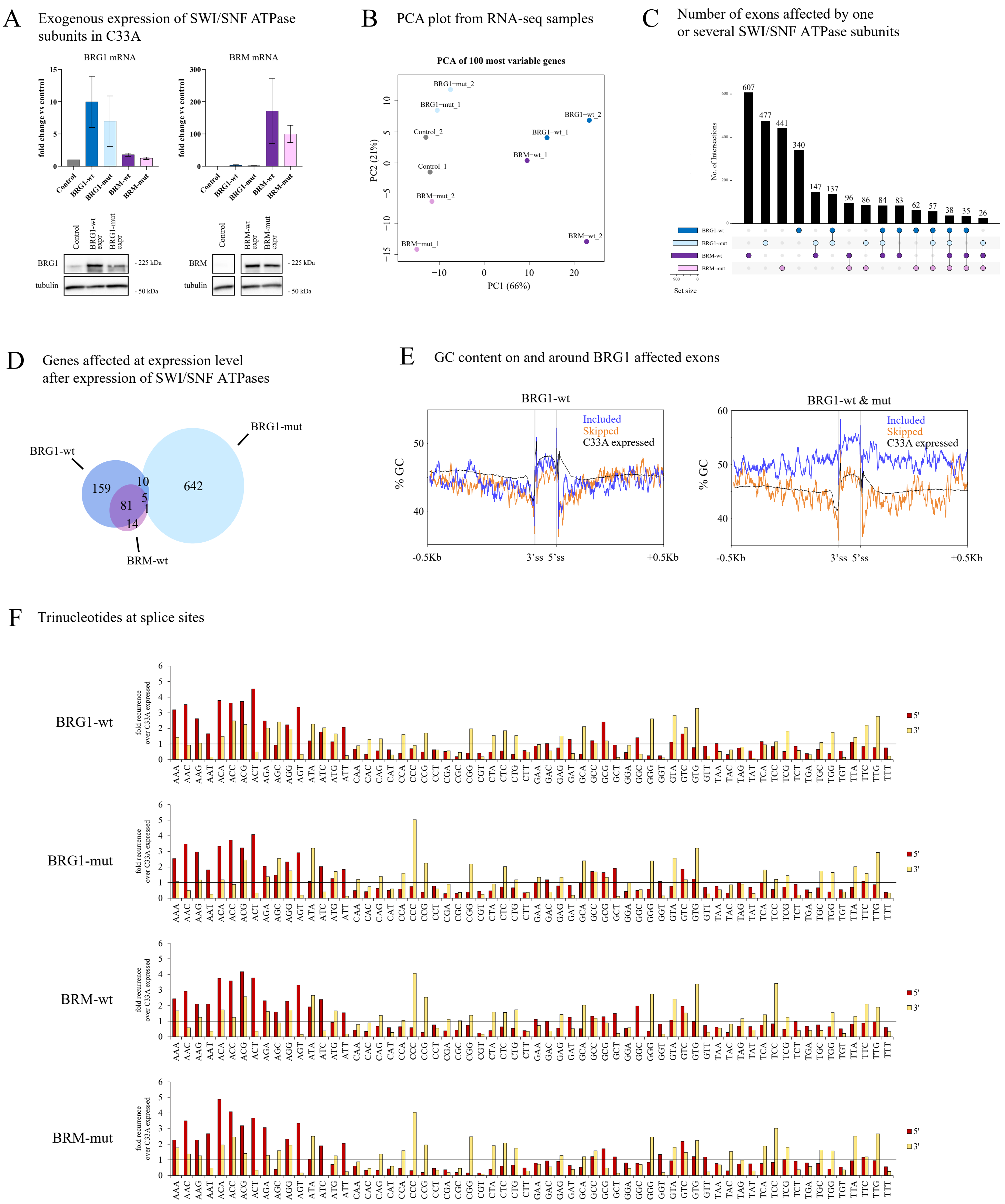

G Sashimi plots from affected exons area

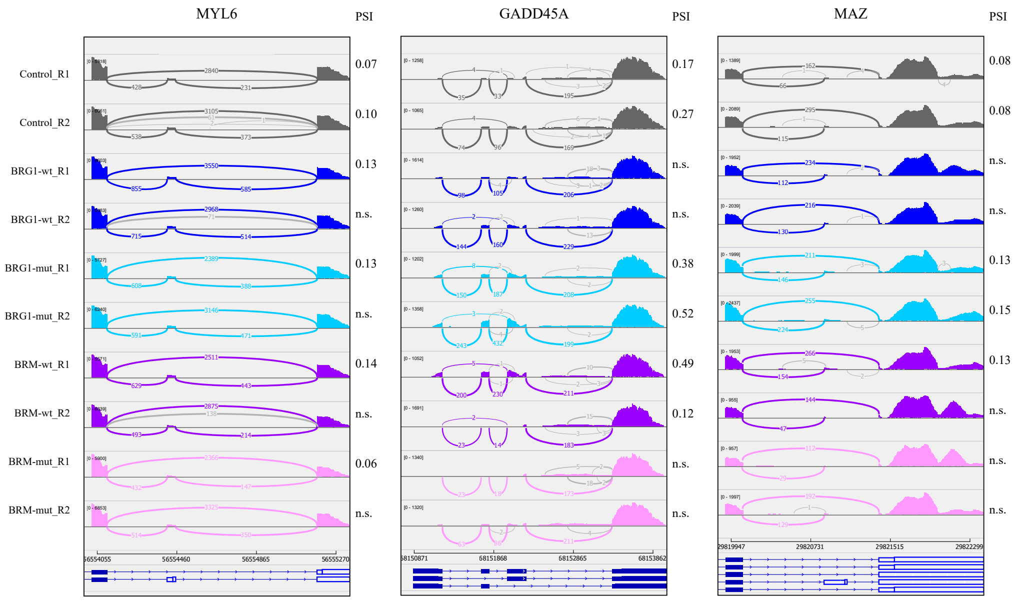

H SWI/SNF KD in HeLa westerns

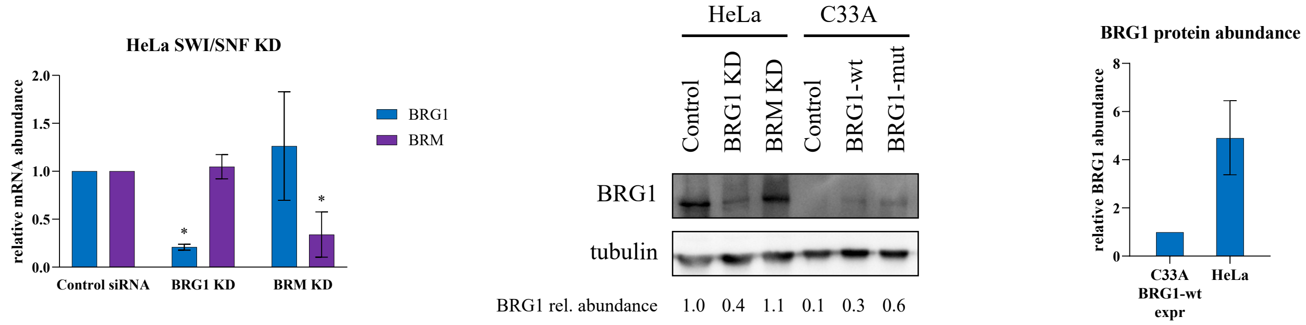

Supplementary Figure 2

A SWI/SNF ATPases ChIP - BRM-wt and BRM-mut expression in C33A

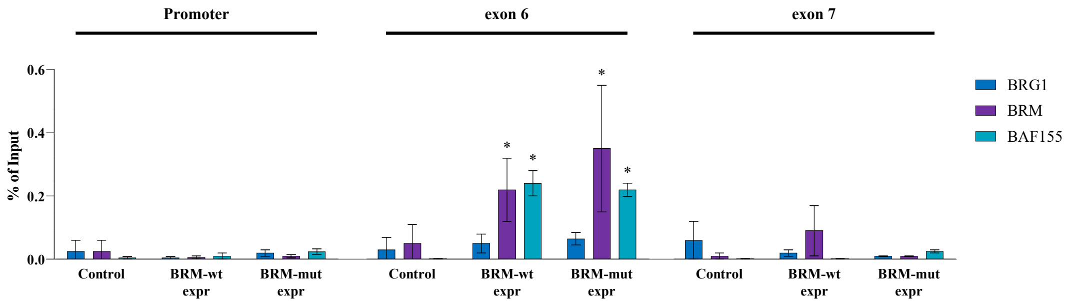

B

SWI/SNF ChIP - GADD45A ex2

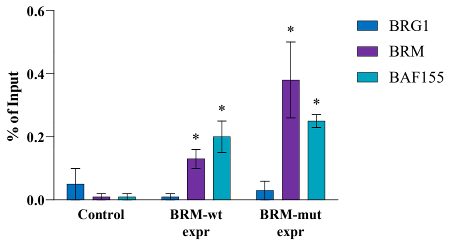

C

SWI/SNF ChIP - MAZ ex5

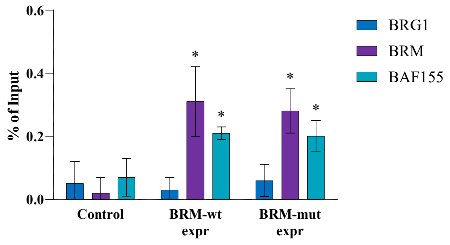

Supplementary Figure 3

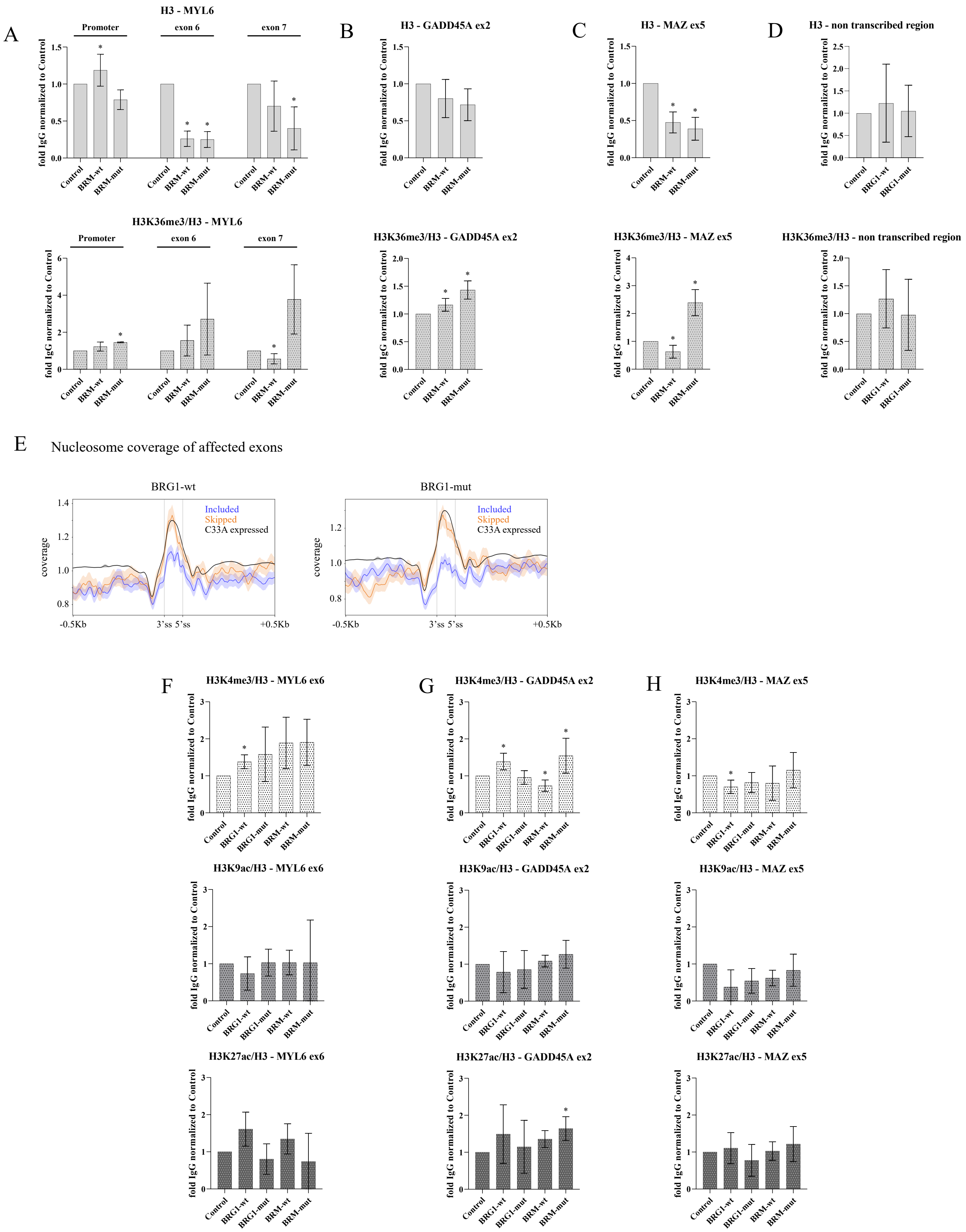

Supplementary Figure 4

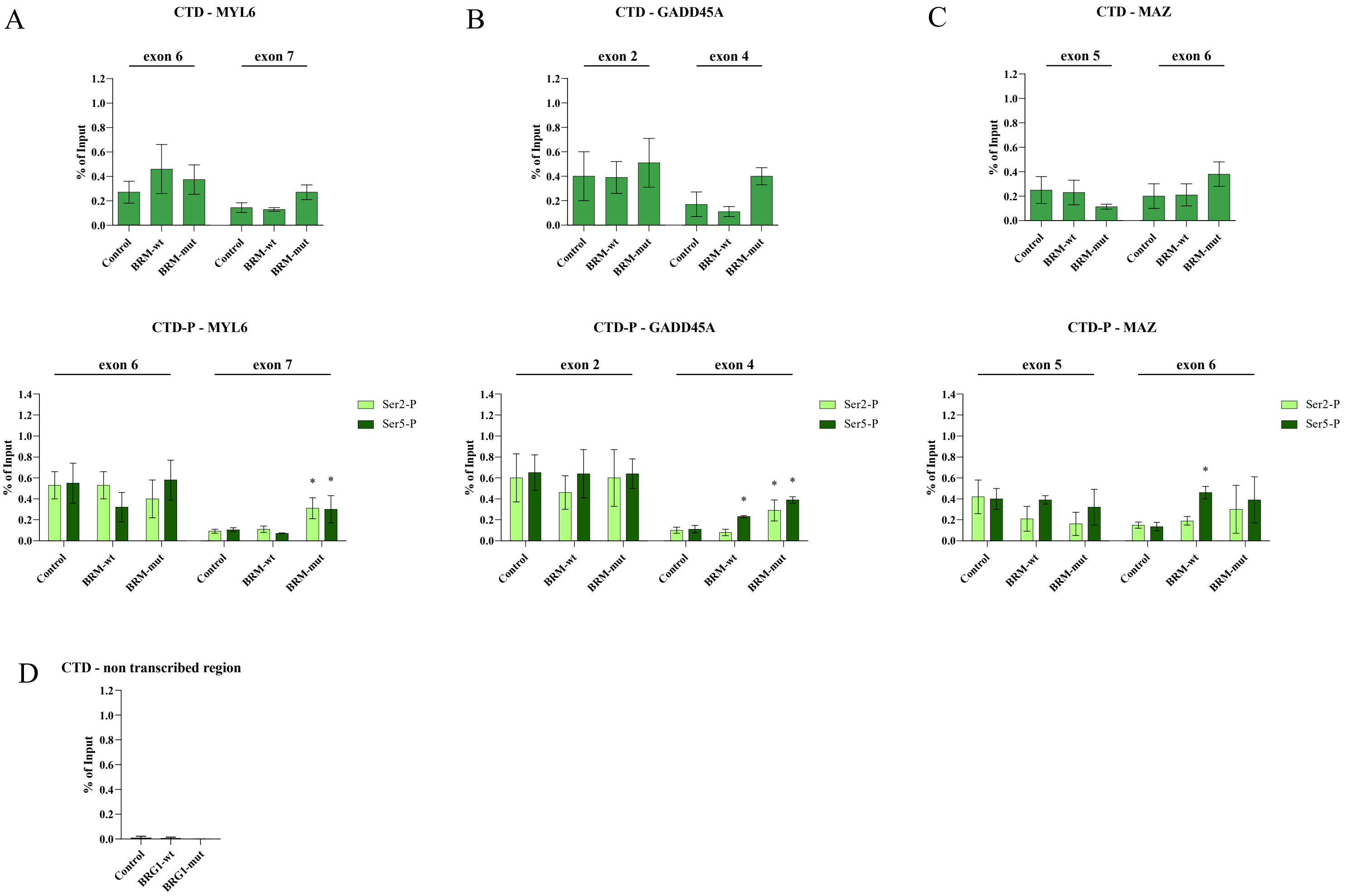

Supplementary Figure 5

A GO terms related to splicing from BRG1 and BRM interactors

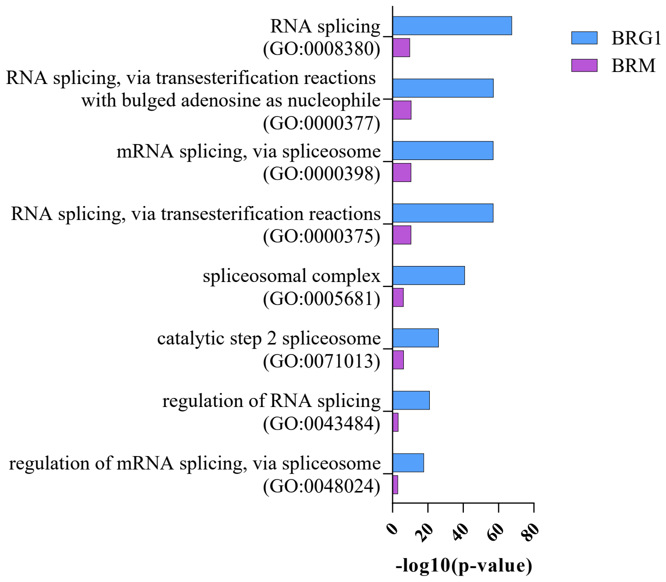

B SWI/SNF ATPase interactors in the RNP fraction

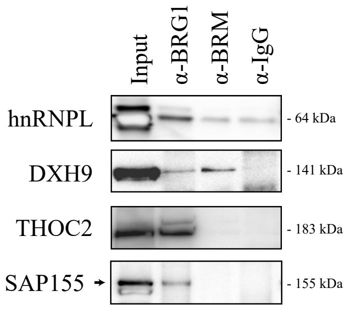

C SAM68 interacts with SWI/SNF ATPases in the RNP fraction

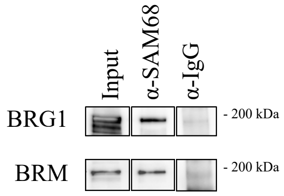

Supplementary Figure 6

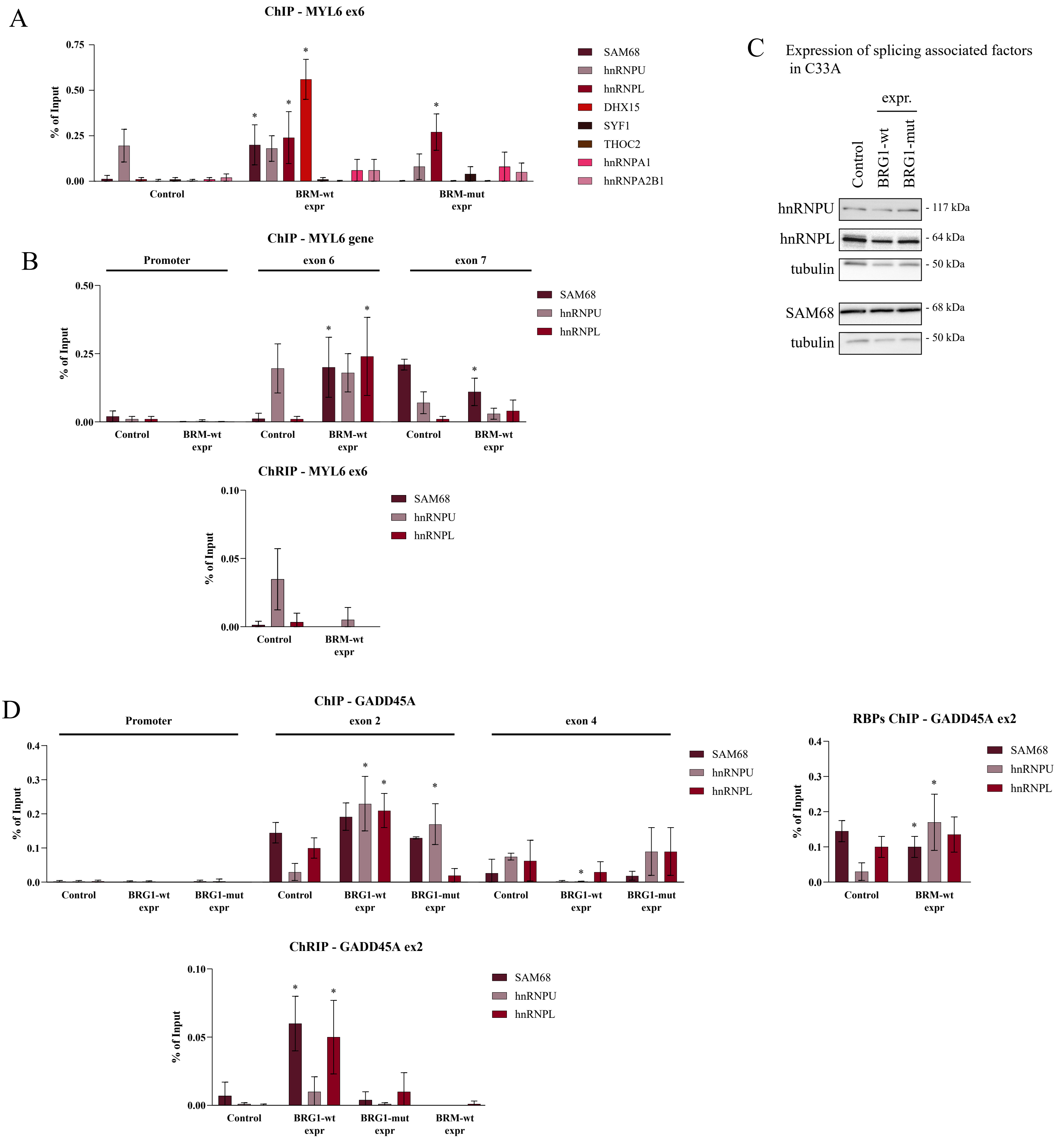

E

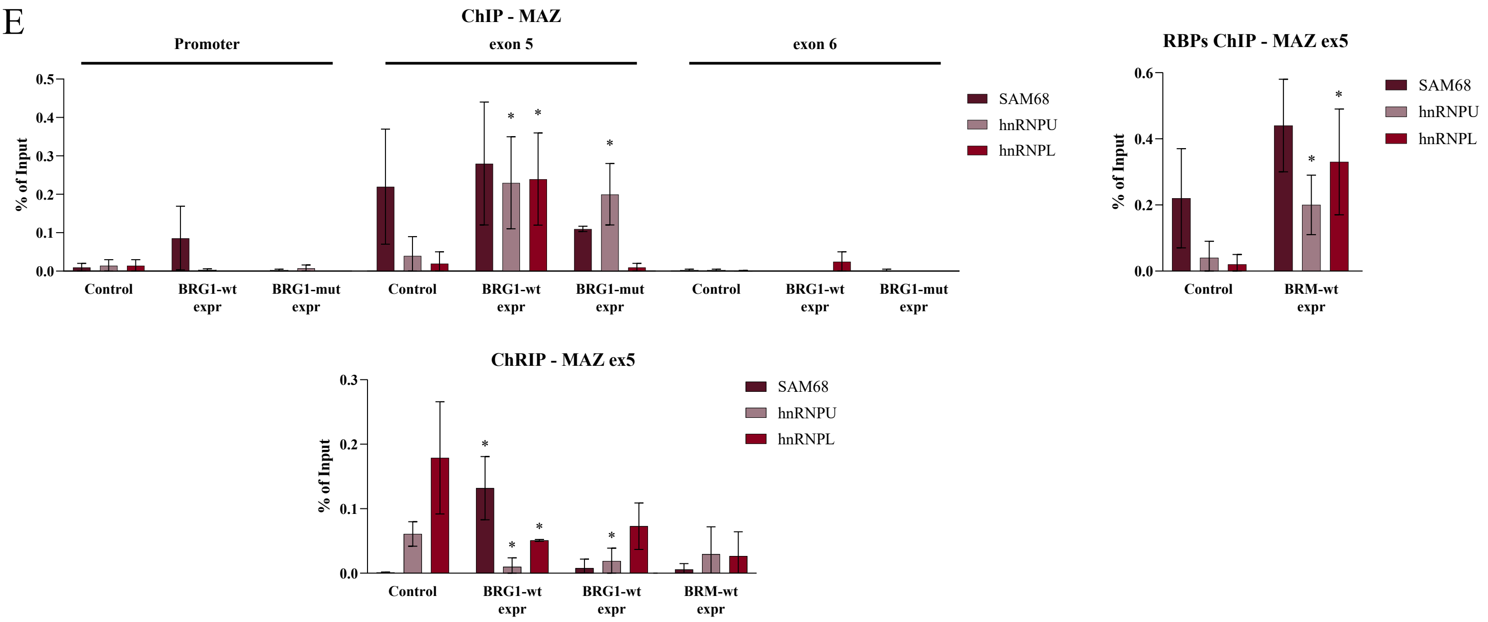

F

Splicing associated factors knock down efficiency in C33A cells

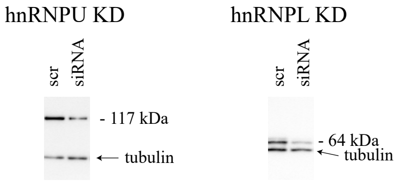

Table S1. Exons affected by SWI/SNF ATPase subunits

BRG1-wt

Affected exon coordinates	included/skipped	ENSEMBL gene ID	gene coordinates	Gene name
1:6078695:6078895:+	skipped	ENSG00000069424	1:6051525:6160235:+	KCNAB2
1:6293534:6293703:-	skipped	ENSG00000116237	1:6281252:6296000:-	ICMT
1:9165545:9165739:-	skipped	ENSG00000180758	1:9164590:9171429:-	GPR157
1:10167280:10167432:+	skipped	ENSG00000130939	1:10167388:10192538:+	UBE4B
1:23660011:23660124:-	skipped	ENSG00000125944	1:23631180:23670829:-	HNRNPR
1:23667345:23667513:-	skipped	ENSG00000125944	1:23664762:23670810:-	HNRNPR
1:26149731:26149930:+	skipped	ENSG00000117640	1:26149486:26156288:+	MTFR1L
1:27682160:27682236:-	skipped	ENSG00000142733	1:27681674:27682962:-	MAP3K6
1:29547350:29547433:-	skipped	ENSG00000116353	1:29542548:29550061:-	MECR
1:36833449:36833685:-	skipped	ENSG00000196182	1:36833454:36851477:-	STK40
1:38187678:38187968:-	skipped	ENSG00000183317	1:38179551:38197166:-	EPHA10
1:43161870:43161959:+	skipped	ENSG00000065978	1:43148097:43168020:+	YBX1
1:52924008:52924121:-	skipped	ENSG00000134744	1:52898760:52928584:-	ZCCHC11
1:68947729:68948580:-	skipped	ENSG00000233589	1:68944811:68949222:+	RP4-694A7.2
1:70696240:70696301:+	skipped	ENSG00000116754	1:70687162:70696951:+	SRSF11
1:85035614:85035822:-	skipped	ENSG00000117151	1:85015288:85040147:-	CTBS
1:95426873:95426989:+	skipped	ENSG00000235501	1:95426880:95428826:+	RP4-639F20.1
1:110027954:110028131:+	skipped	ENSG00000162650	1:110026100:110035326:+	ATXN7L2
1:110037765:110037802:+	skipped	ENSG00000174151	1:110036673:110038894:+	CYB561D1
1:110199972:110200293:+	skipped	ENSG00000168765	1:110198873:110204322:+	GSTM4
1:110200212:110200293:+	skipped	ENSG00000168765	1:110198873:110204322:+	GSTM4
1:144619347:144619419:+	skipped	ENSG00000225241	1:144614531:144619419:+	RP11-640M9.2
1:145290781:145290850:+	skipped	ENSG00000213240	1:145290421:145291972:+	NOTCH2NL
1:147599424:147599571:-	skipped	ENSG00000203836	1:147596183:147615861:-	NBPF24
1:147908554:147908674:-	skipped	ENSG00000225871	1:147907214:147909036:+	RP11-495P10.10
1:148342470:148342542:-	skipped	ENSG00000203832	1:148341795:148346929:-	NBPF20
1:154186369:154186422:-	skipped	ENSG00000143612	1:154184932:154186992:-	C1orf43
1:154241838:154241888:+	skipped	ENSG00000143569	1:154193324:154243328:+	UBAP2L
1:155172914:155173062:+	skipped	ENSG00000169231	1:155165378:155177688:-	THBS3
1:155213886:155214021:-	skipped	ENSG00000177628	1:155208003:155214427:-	GBA
1:156703801:156704285:+	skipped	ENSG00000143303	1:156698233:156706741:+	RRNAD1
1:172522400:172522510:+	skipped	ENSG00000094975	1:172501488:172580971:+	SUCO
1:173684662:173684793:+	skipped	ENSG00000076321	1:173684079:173702967:+	KLHL20
1:226346885:226347059:-	skipped	ENSG00000182827	1:226342342:226349333:-	ACBD3
1:227838657:227838777:+	skipped	ENSG00000181450	1:227751243:227843529:+	ZNF678
1:234529108:234529232:-	skipped	ENSG00000059588	1:234527058:234614849:-	TARBP1
1:241767562:241767881:-	skipped	ENSG00000054277	1:241756425:241803663:-	OPN3
10:7843476:7843532:+	skipped	ENSG00000165629	10:7830091:7849778:+	ATP5C1
10:7848937:7848973:+	skipped	ENSG00000165629	10:7830091:7849778:+	ATP5C1
10:14878250:14878293:-	skipped	ENSG00000185267	10:14861248:14880574:-	CDNF
10:21860904:21860955:+	skipped	ENSG00000078403	10:21823630:21884511:+	MLLT10
10:27442156:27442210:-	skipped	ENSG00000136758	10:27399382:27443288:-	YME1L1
10:32128565:32128639:-	skipped	ENSG00000165322	10:32094364:32197802:-	ARHGAP12
10:33242521:33242737:-	skipped	ENSG00000150093	10:33189246:33246822:-	ITGB1
10:48922187:48922255:-	skipped	ENSG00000204164	10:48901101:48922972:-	BMS1P5
10:51338277:51338363:-	skipped	ENSG00000244393	10:51253925:51371300:-	RP11-592B15.3
10:51613218:51613311:-	skipped	ENSG00000138297	10:51592079:51623338:-	TIMM23
10:61468364:61468541:-	skipped	ENSG00000165449	10:61410522:61469837:-	SLC16A9
10:73983646:73983814:+	skipped	ENSG00000166295	10:73975786:73993373:+	ANAPC16
10:75280666:75280785:-	skipped	ENSG00000166348	10:75257295:75301460:-	USP54
10:75898564:75898689:-	skipped	ENSG00000185009	10:75898013:75910515:-	AP3M1
10:104865115:104865138:-	skipped	ENSG00000076685	10:104857728:104866407:-	NT5C2

Table S1. Exons affected by SWI/SNF ATPase subunits

BRG1-wt

Affected exon coordinates	included/skipped	ENSEMBL gene ID	gene coordinates	Gene name
10:117093792:117093929:+	skipped	ENSG00000107518	10:117059740:117221526:+	ATRNL1
10:123903087:123903221:+	skipped	ENSG00000138162	10:123872544:123970084:+	TACC2
11:3394807:3394905:-	skipped	ENSG00000005801	11:3392209:3400384:-	ZNF195
11:3398847:3399130:-	skipped	ENSG00000005801	11:3392209:3400384:-	ZNF195
11:18436731:18436848:+	skipped	ENSG00000166796	11:18433853:18473605:+	LDHC
11:58876141:58876185:+	skipped	ENSG00000189057	11:58874708:58877480:+	FAM111B
11:62401782:62401847:-	skipped	ENSG00000089597	11:62392297:62414087:-	GANAB
11:63486174:63488504:+	skipped	ENSG00000133318	11:63448917:63527354:+	RTN3
11:64507840:64507921:-	skipped	ENSG00000068831	11:64506850:64510418:-	RASGRP2
11:71800463:71800642:+	skipped	ENSG00000184154	11:71794817:71806016:+	LRTOMT
11:74562133:74562248:-	skipped	ENSG00000166435	11:74559169:74563109:-	XRRA1
11:76170977:76171129:+	skipped	ENSG00000158636	11:76155966:76208275:+	C11orf30
11:102953985:102954055:-	skipped	ENSG00000137692	11:102932804:102960047:-	DCUN1D5
11:111956691:111957034:-	skipped	ENSG00000150779	11:111955523:111957522:-	TIMM8B
11:117077783:117077876:-	skipped	ENSG00000160613	11:117076662:117078852:-	PCSK7
11:117234145:117234222:+	skipped	ENSG00000110274	11:117198570:117283984:+	CEP164
11:125445201:125445304:+	skipped	ENSG00000149547	11:125439303:125450094:+	EI24
12:514677:514730:+	skipped	ENSG00000120647	12:510816:518947:+	CCDC77
12:3938076:3938196:-	skipped	ENSG00000111224	12:3938139:3982608:-	PARP11
12:8340328:8340821:+	skipped	ENSG00000196946	12:8328452:8341133:+	ZNF705A
12:9144802:9144906:+	skipped	ENSG00000139187	12:9102639:9162044:+	KLRG1
12:10761697:10761982:-	skipped	ENSG00000111196	12:10758611:10766222:-	MAGOHB
12:21654104:21654149:-	skipped	ENSG00000004700	12:21643308:21654527:-	RECQL
12:30839255:30839593:-	skipped	ENSG00000133704	12:30837234:30841680:-	IPO8
12:50414845:50414960:-	skipped	ENSG00000161800	12:50382944:50419307:-	RACGAP1
12:52437586:52437695:+	skipped	ENSG00000123358	12:52431025:52439352:+	NR4A1
12:52446280:52446429:+	skipped	ENSG00000123358	12:52445190:52453285:+	NR4A1
12:53587492:53587685:-	skipped	ENSG00000139626	12:53586751:53588042:-	ITGB7
12:53849245:53849277:+	skipped	ENSG00000197111	12:53846600:53849734:+	PCBP2
12:56396388:56396504:+	skipped	ENSG00000139531	12:56395281:56399309:+	SUOX
12:56617368:56617496:+	skipped	ENSG00000139579	12:56615798:56622816:+	NABP2
12:57064059:57064148:-	skipped	ENSG00000110958	12:57057126:57082084:-	PTGES3
12:57883050:57883341:+	skipped	ENSG00000166986	12:57881838:57884125:+	MARS
12:77256590:77256739:-	skipped	ENSG00000175183	12:77252495:77258734:-	CSRP2
12:109046814:109046836:-	skipped	ENSG00000110880	12:109038884:109125372:-	CORO1C
12:110937340:110937351:-	skipped	ENSG00000111237	12:110936600:110939912:-	VPS29
12:120648733:120648909:+	skipped	ENSG00000255857	12:120639093:120650722:+	PXN-AS1
12:123425354:123425542:-	skipped	ENSG00000150967	12:123424672:123429249:-	ABCB9
12:124440831:124440991:-	skipped	ENSG00000119242	12:124420955:124457371:-	CCDC92
12:132249001:132249231:+	skipped	ENSG00000061936	12:132195633:132284282:+	SFSWAP
12:132448077:132448187:+	skipped	ENSG00000183495	12:132434507:132565005:+	EP400
12:133720106:133720208:+	skipped	ENSG00000256223	12:133707160:133727709:+	ZNF10
13:39616242:39616442:+	skipped	ENSG00000188811	13:39612447:39624246:+	NHLRC3
13:42795298:42795530:+	skipped	ENSG00000102780	13:42614175:42803294:+	DGKH
13:76111800:76111811:-	skipped	ENSG00000188243	13:76103844:76111980:-	COMMD6
13:78317151:78317300:+	skipped	ENSG00000139737	13:78315672:78320993:+	SLAIN1
13:95918366:95918518:-	skipped	ENSG00000125257	13:95672082:95953687:-	ABCC4
13:98659616:98659841:+	skipped	ENSG00000065150	13:98655262:98659841:+	IPO5
14:21731826:21731988:-	skipped	ENSG00000092199	14:21730761:21737601:-	HNRNPC
14:21840628:21840876:-	skipped	ENSG00000092201	14:21840233:21852172:-	SUPT16H
14:23469166:23469326:-	skipped	ENSG00000100802	14:23465448:23471978:-	C14orf93
14:24459984:24460161:+	skipped	ENSG00000187630	14:24439147:24475610:+	DHRS4L2

Table S1. Exons affected by SWI/SNF ATPase subunits

BRG1-wt

Affected exon coordinates	included/skipped	ENSEMBL gene ID	gene coordinates	Gene name
14:24710226:24710982:-	skipped	ENSG00000092330	14:24708848:24711880:-	TINF2
14:24911304:24911466:-	skipped	ENSG00000100445	14:24908971:24911982:-	SDR39U1
14:32586349:32586493:+	skipped	ENSG00000100852	14:32546172:32624217:+	ARHGAP5
14:53327732:53327752:-	skipped	ENSG00000073712	14:53323989:53328021:-	FERMT2
14:55310719:55310861:-	skipped	ENSG00000131979	14:55308725:55369542:-	GCH1
14:58759784:58759856:-	skipped	ENSG00000257621	14:58732082:58764847:-	RP11-349A22.5
14:68059352:68059432:-	skipped	ENSG00000100564	14:68048671:68066886:-	PIGH
14:69670634:69670717:+	skipped	ENSG00000258957	14:69649736:69680809:-	RP11-363J20.1
14:75528387:75528465:-	skipped	ENSG00000119640	14:75519923:75530752:-	ACYP1
14:91751739:91752180:-	skipped	ENSG00000015133	14:91737666:91755675:-	CCDC88C
14:100341268:100341324:+	skipped	ENSG00000066629	14:100317186:100349883:+	EML1
14:100835424:100835595:-	skipped	ENSG00000140105	14:100828039:100841810:-	WARS
15:23027801:23027922:-	skipped	ENSG00000140157	15:23019818:23034378:-	NIPA2
15:23196162:23196291:-	skipped	ENSG00000187667	15:23187727:23208417:-	WHAMMP3
15:25300221:25300377:+	skipped	ENSG00000224078	15:25295778:25367623:+	SNHG14
15:41104897:41105100:+	skipped	ENSG00000166140	15:41099283:41106543:+	ZFYVE19
15:41556363:41556433:+	skipped	ENSG00000187446	15:41523036:41571633:+	CHP1
15:42852980:42853068:+	skipped	ENSG00000137814	15:42851581:42853885:+	HAUS2
15:43924915:43925132:-	skipped	ENSG00000166762	15:43922768:43924935:-	CATSPER2
15:44862723:44862856:-	skipped	ENSG00000104133	15:44859535:44865881:-	SPG11
15:45884333:45884474:+	skipped	ENSG00000104164	15:45879320:45901909:+	BLOC1S6
15:45895298:45895385:+	skipped	ENSG00000104164	15:45879320:45901909:+	BLOC1S6
15:63826002:63826117:+	skipped	ENSG00000140455	15:63820031:63829255:+	USP3
15:65112046:65112218:-	skipped	ENSG00000140451	15:65111235:65113656:-	PIF1
15:69745986:69747626:+	skipped	ENSG00000137818	15:69745122:69748255:+	RPLP1
15:76587932:76588078:-	skipped	ENSG00000140374	15:76566823:76603754:-	ETFA
15:78888882:78889074:-	skipped	ENSG00000080644	15:78885393:78889074:-	CHRNA3
15:83087938:83087977:+	skipped	ENSG00000259429	15:83084256:83088121:+	UBE2Q2P3
15:83145986:83146023:-	skipped	ENSG00000259472	15:83145206:83146651:-	RP13-996F3.3
15:84872929:84873083:-	skipped	ENSG00000225151	15:84869632:84873277:-	GOLGA2P7
15:86201768:86201821:+	skipped	ENSG00000259367	15:86162853:86214179:-	RP11-815J21.4
16:2012737:2012910:-	skipped	ENSG00000140988	16:2012052:2014861:-	RPS2
16:2012874:2012910:-	skipped	ENSG00000140988	16:2012052:2014861:-	RPS2
16:3336023:3336149:+	skipped	ENSG00000006194	16:3332942:3341460:+	ZNF263
16:4776660:4776781:-	skipped	ENSG00000168096	16:4746512:4777170:-	ANKS3
16:11655002:11655430:-	skipped	ENSG00000189067	16:11641852:11681419:-	LITAF
16:18466435:18466711:-	skipped	ENSG00000233024	16:18451942:18468926:-	NPIPA7
16:18466580:18466711:-	skipped	ENSG00000233024	16:18451942:18468926:-	NPIPA7
16:27252674:27253210:-	skipped	ENSG00000169189	16:27236311:27280115:-	NSMCE1
16:27352391:27352634:+	skipped	ENSG00000077238	16:27324988:27356341:+	IL4R
16:28164251:28164285:-	skipped	ENSG00000169180	16:28143718:28167296:-	XPO6
16:29983363:29983461:+	skipped	ENSG00000149932	16:29952205:29984371:+	TMEM219
16:57212414:57212764:-	skipped	ENSG00000172775	16:57186377:57219976:-	FAM192A
16:66597025:66597120:+	skipped	ENSG00000217555	16:66586465:66600154:+	CKLF
16:67855002:67855128:+	skipped	ENSG00000102904	16:67840667:67861917:+	TSNAXIP1
16:89615388:89615744:+	skipped	ENSG00000197912	16:89611133:89619487:+	SPG7
17:1273003:1273035:-	skipped	ENSG00000108953	17:1247565:1303672:-	YWHAE
17:8347245:8347494:+	skipped	ENSG00000166579	17:8339968:8351895:+	NDEL1
17:16342842:16343017:+	skipped	ENSG00000175061	17:16342288:16345052:+	FAM211A-AS1
17:17150794:17150874:-	skipped	ENSG00000141030	17:17150148:17152341:-	COPS3
17:18762653:18762788:+	skipped	ENSG00000141127	17:18761408:18775941:+	PRPSAP2
17:30344991:30345073:+	skipped	ENSG00000185158	17:30334890:30348875:+	LRRC37B

Table S1. Exons affected by SWI/SNF ATPase subunits

BRG1-wt

Affected exon coordinates	included/skipped	ENSEMBL gene ID	gene coordinates	Gene name
17:31258345:31258420:+	skipped	ENSG00000006042	17:31254927:31268631:+	TMEM98
17:33434385:33434466:-	skipped	ENSG00000185379	17:33426810:33448516:-	RAD51D
17:33445520:33445638:-	skipped	ENSG00000185379	17:33443962:33446888:-	RAD51D
17:38189896:38190024:-	skipped	ENSG00000008838	17:38188923:38189899:-	MED24
17:39983678:39983878:-	skipped	ENSG00000141698	17:39981334:39992523:-	NT5C3B
17:41258495:41258550:-	skipped	ENSG00000012048	17:41196311:41277387:-	BRCA1
17:44067244:44067441:+	skipped	ENSG00000186868	17:44050148:44101797:+	MAPT
17:49231586:49231805:+	skipped	ENSG00000239672	17:49230932:49233907:+	NME1
17:61803998:61804055:-	skipped	ENSG00000266173	17:61780191:61819143:-	STRADA
17:61905034:61905283:+	skipped	ENSG00000108592	17:61904474:61907372:-	FTSJ3
17:65882244:65882432:+	skipped	ENSG00000171634	17:65871035:65888078:+	BPTF
17:78252563:78252709:+	skipped	ENSG00000173821	17:78234664:78369112:+	RNF213
18:23615795:23615887:-	skipped	ENSG00000141380	18:23596577:23670589:-	SS18
18:47018106:47018203:-	skipped	ENSG00000265496	18:47013708:47018248:+	MIR1539
18:77135394:77135426:+	skipped	ENSG00000166377	18:77090079:77138141:+	ATP9B
18:77693969:77694022:-	skipped	ENSG00000122490	18:77662419:77711664:-	PQLC1
19:5901346:5901465:-	skipped	ENSG00000266941	19:5899905:5901472:+	AC104532.3
19:9448472:9448534:+	skipped	ENSG00000188321	19:9434447:9454519:+	ZNF559
19:9947499:9947551:+	skipped	ENSG00000127445	19:9945998:9960358:+	PIN1
19:11150134:11150229:+	skipped	ENSG00000127616	19:11141488:11152142:+	SMARCA4
19:14812396:14812709:+	skipped	ENSG00000160961	19:14800612:14818074:+	ZNF333
19:16623825:16623920:+	skipped	ENSG00000105072	19:16623879:16628931:+	C19orf44
19:19144940:19145047:+	skipped	ENSG00000105676	19:19144383:19168987:+	ARMC6
19:37871179:37871274:+	skipped	ENSG00000189164	19:37862060:37876546:+	ZNF527
19:46886668:46886733:+	skipped	ENSG00000011485	19:46878883:46886968:+	PPP5C
19:50183129:50183182:+	skipped	ENSG00000126457	19:50180523:50183376:+	PRMT1
19:52703527:52703651:+	skipped	ENSG00000105568	19:52693291:52730687:+	PPP2R1A
19:55595135:55596010:+	skipped	ENSG00000131037	19:55591759:55599287:+	EPS8L1
19:58426706:58426899:-	skipped	ENSG00000269476	19:58388132:58427959:-	CTD-2583A14.9
19:58515751:58516068:+	skipped	ENSG00000176593	19:58513428:58522600:+	CTD-2368P22.1
2:224864:224920:-	skipped	ENSG00000035115	2:218137:242800:-	SH3YL1
2:262631:262786:-	skipped	ENSG00000035115	2:218135:264032:-	SH3YL1
2:675758:676238:-	skipped	ENSG00000151353	2:675557:676642:-	TMEM18
2:9554307:9554385:-	skipped	ENSG00000119185	2:9552397:9563216:-	ITGB1BP1
2:9616116:9616168:+	skipped	ENSG00000134330	2:9613786:9624677:+	IAH1
2:27573181:27573499:-	skipped	ENSG00000115207	2:27548715:27579359:-	GTF3C2
2:38973286:38973321:-	skipped	ENSG00000115875	2:38971858:38976706:-	SRSF7
2:62107421:62107529:-	skipped	ENSG00000115484	2:62095223:62115939:-	CCT4
2:62115159:62115392:-	skipped	ENSG00000115484	2:62115082:62115593:-	CCT4
2:73635750:73635875:+	skipped	ENSG00000116127	2:73612885:73836842:+	ALMS1
2:98088032:98088252:-	skipped	ENSG00000230606	2:98081773:98089122:-	AC159540.1
2:98317617:98317767:+	skipped	ENSG00000228486	2:98305387:98319378:+	LINC01125
2:109404488:109404545:+	skipped	ENSG00000163006	2:109403228:109411126:+	CCDC138
2:113170008:113170203:-	skipped	ENSG00000169629	2:113127668:113191222:-	RGPD8
2:114351865:114352093:+	skipped	ENSG00000146556	2:114346126:114356611:+	WASH2P
2:122514816:122515010:+	skipped	ENSG00000211460	2:122513235:122515184:+	TSN
2:136511109:136511847:+	skipped	ENSG00000144224	2:136499188:136542625:+	UBXN4
2:174223983:174224219:+	skipped	ENSG00000144354	2:174219547:174233632:+	CDCA7
2:174224071:174224219:+	skipped	ENSG00000144354	2:174219547:174233632:+	CDCA7
2:174828489:174828611:-	skipped	ENSG00000172845	2:174820544:174830038:-	SP3
2:190650072:190650197:+	skipped	ENSG00000064933	2:190649214:190656667:+	PMS1
2:191514557:191514708:+	skipped	ENSG00000138386	2:191513620:191524374:+	NAB1

Table S1. Exons affected by SWI/SNF ATPase subunits

BRG1-wt

Affected exon coordinates	included/skipped	ENSEMBL gene ID	gene coordinates	Gene name
2:191819481:191819544:+	skipped	ENSG00000115419	2:191792397:191827884:+	GLS
2:202141550:202141827:+	skipped	ENSG00000064012	2:202122702:202141627:+	CASP8
2:207025611:207025856:+	skipped	ENSG00000114942	2:207024308:207027652:+	EEF1B2
2:209138745:209138777:+	skipped	ENSG00000115020	2:209130990:209190831:+	PIKFYVE
2:220072370:220072496:+	skipped	ENSG00000158552	2:220071505:220073622:+	ZFAND2B
2:223765392:223765498:+	skipped	ENSG00000123983	2:223725651:223809357:+	ACSL3
2:242259618:242259702:+	skipped	ENSG00000168385	2:242255308:242264659:+	SEPT2
2:242621334:242621748:-	skipped	ENSG00000168393	2:242615156:242626406:-	DTYMK
20:32661625:32661672:+	skipped	ENSG00000125970	20:32659871:32664852:+	RALY
20:33721908:33721958:-	skipped	ENSG00000088298	20:33703166:33735122:-	EDEM2
20:56965184:56965396:+	skipped	ENSG00000124164	20:56964177:57026157:+	VAPB
20:60702641:60702757:+	skipped	ENSG00000149657	20:60697516:60710434:+	LSM14B
20:62367133:62369000:+	skipped	ENSG00000203896	20:62366814:62369794:+	LIME1
20:62507169:62507228:+	skipped	ENSG00000101150	20:62496595:62521777:+	TPD52L2
20:62579578:62579828:-	skipped	ENSG00000198276	20:62571276:62582479:-	UCKL1
20:62609598:62609714:-	skipped	ENSG00000130590	20:62605465:62610995:-	SAMD10
21:34809190:34809299:-	skipped	ENSG00000159128	21:34775201:34809828:+	IFNGR2
21:35199122:35199167:+	skipped	ENSG00000205726	21:35190329:35201333:+	ITSN1
21:43422459:43422672:-	skipped	ENSG00000173276	21:43406939:43430466:-	ZBTB21
21:45380513:45380660:+	skipped	ENSG00000160216	21:45366810:45404554:+	AGPAT3
21:45482934:45483049:+	skipped	ENSG00000160218	21:45432199:45526433:+	TRAPPC10
21:47711248:47711376:+	skipped	ENSG00000182362	21:47706250:47717665:+	YBEY
22:17990845:17990928:+	skipped	ENSG00000099954	22:17840836:18018834:+	CECR2
22:18232871:18232940:-	skipped	ENSG00000015475	22:18220823:18257261:-	BID
22:24379361:24379450:-	skipped	ENSG00000184674	22:24376132:24384284:-	GSTT1
22:38347467:38347529:-	skipped	ENSG00000128346	22:38339527:38349654:-	C22orf23
22:42300539:42300736:+	skipped	ENSG00000198911	22:42294679:42300977:+	SREBF2
3:9437190:9437263:-	skipped	ENSG00000206573	3:9391372:9438338:-	SETD5-AS1
3:10360823:10361000:-	skipped	ENSG00000157020	3:10354763:10362796:-	SEC13
3:15094798:15094974:-	skipped	ENSG00000177463	3:15073933:15095107:+	NR2C2
3:17560394:17560487:-	skipped	ENSG00000131374	3:17198653:17782399:-	TBC1D5
3:17665341:17665405:-	skipped	ENSG00000131374	3:17198653:17782399:-	TBC1D5
3:31871532:31871723:-	skipped	ENSG00000144645	3:31699381:32022794:-	OSBPL10
3:37196105:37196147:-	skipped	ENSG00000093167	3:37094116:37217851:-	LRRFIP2
3:48341921:48342124:-	skipped	ENSG00000172113	3:48334753:48342848:-	NME6
3:48731892:48731958:-	skipped	ENSG00000068745	3:48726970:48733021:-	IP6K2
3:48752748:48752960:-	skipped	ENSG00000068745	3:48725435:48754683:-	IP6K2
3:48752822:48752960:-	skipped	ENSG00000068745	3:48725435:48754683:-	IP6K2
3:50599804:50600019:-	skipped	ENSG00000088543	3:50595461:50605096:-	C3orf18
3:51424340:51424477:+	skipped	ENSG00000145050	3:51422477:51426828:+	MANF
3:58351601:58351651:+	skipped	ENSG00000168297	3:58318606:58410878:+	PXK
3:119170056:119170164:-	skipped	ENSG00000176142	3:119148346:119182444:-	TMEM39A
3:119197181:119197321:+	skipped	ENSG00000163389	3:119196969:119198826:+	POGLUT1
3:119222379:119222467:+	skipped	ENSG00000113845	3:119217378:119243937:+	TIMMDC1
3:131220688:131220768:-	skipped	ENSG00000114686	3:131206524:131221795:-	MRPL3
3:137790485:137790677:-	skipped	ENSG00000158163	3:137780831:137834451:-	DZIP1L
3:179333771:179333837:+	skipped	ENSG00000136521	3:179332993:179336310:+	NDUFB5
3:195388627:195388712:+	skipped	ENSG00000242086	3:195384932:195393553:+	LINC00969
3:197607383:197607628:+	skipped	ENSG00000186001	3:197556498:197612701:+	LRCH3
4:466364:466490:-	skipped	ENSG00000251595	4:419223:467918:-	ABCA11P
4:2243247:2243564:-	skipped	ENSG00000214367	4:2229190:2243848:-	HAUS3
4:4314768:4314841:+	skipped	ENSG00000168826	4:4291923:4323512:+	ZBTB49

Table S1. Exons affected by SWI/SNF ATPase subunits

BRG1-wt

Affected exon coordinates	included/skipped	ENSEMBL gene ID	gene coordinates	Gene name
4:15686473:15686582:+	skipped	ENSG00000237765	4:15683284:15688935:+	FAM200B
4:39779302:39779430:+	skipped	ENSG00000078140	4:39699663:39784412:+	UBE2K
4:40101656:40101746:+	skipped	ENSG00000078177	4:40058555:40104226:+	N4BP2
4:83292681:83292737:-	skipped	ENSG00000138668	4:83279910:83295314:-	HNRNPD
4:83742190:83742261:-	skipped	ENSG00000138674	4:83740173:83748765:-	SEC31A
4:99830829:99831110:-	skipped	ENSG00000249055	4:99830687:99830999:-	TBCAP3
4:110786275:110786431:+	skipped	ENSG00000183423	4:110769357:110793471:+	LRIT3
4:111542320:111542525:-	skipped	ENSG00000164093	4:111538578:111558508:-	PITX2
4:123818779:123818833:-	skipped	ENSG00000138685	4:123747862:123819391:+	FGF2
4:128886227:128886637:-	skipped	ENSG00000164073	4:128851860:128887099:-	MFSD8
4:128942292:128942393:+	skipped	ENSG00000164074	4:128886460:128952445:+	C4orf29
4:183837439:183837692:-	skipped	ENSG00000129187	4:183836075:183838498:-	DCTD
5:32235068:32235235:-	skipped	ENSG00000150712	5:32227099:32313044:-	MTMR12
5:64858630:64858835:-	skipped	ENSG00000123219	5:64856796:64858963:-	CENPK
5:79723311:79723364:+	skipped	ENSG00000039319	5:79703831:79775169:+	ZFYVE16
5:80546522:80546608:-	skipped	ENSG00000247572	5:80499902:80597304:-	CKMT2-AS1
5:102323222:102323275:+	skipped	ENSG00000145730	5:102285280:102365298:+	PAM
5:102523015:102523077:+	skipped	ENSG00000145725	5:102519943:102526759:+	PPIP5K2
5:122495208:122495332:+	skipped	ENSG00000061455	5:122424815:122529960:+	PRDM6
5:125929800:125929959:-	skipped	ENSG00000164904	5:125877532:125931110:-	ALDH7A1
5:132109615:132109904:-	skipped	ENSG00000164402	5:132086508:132113067:-	SEPT8
5:135483521:135483595:+	skipped	ENSG00000113658	5:135483108:135489370:+	SMAD5
5:139244700:139244758:-	skipped	ENSG00000158458	5:139227501:139283982:-	NRG2
5:139661034:139661118:-	skipped	ENSG00000113068	5:139624623:139682706:-	PFDN1
5:140020603:140020805:+	skipped	ENSG00000131495	5:140018324:140026899:-	NDUFA2
5:140073520:140073639:+	skipped	ENSG00000112855	5:140071010:140078876:+	HARS2
5:145889630:145889723:+	skipped	ENSG00000113649	5:145886933:145891068:+	TCERG1
6:2969013:2969147:-	skipped	ENSG00000124570	6:2948392:2972090:-	SERPINB6
6:10687713:10687794:-	skipped	ENSG00000137434	6:10671650:10694766:-	C6orf52
6:26368218:26368495:+	skipped	ENSG00000186470	6:26365386:26370831:+	BTN3A2
6:34614378:34614575:-	skipped	ENSG00000196821	6:34555064:34664636:-	C6orf106
6:41086980:41087202:+	skipped	ENSG00000161912	6:41068760:41106465:+	ADCY10P1
6:41884523:41884677:-	skipped	ENSG00000124641	6:41884593:41888843:-	MED20
6:42848599:42848704:+	skipped	ENSG00000146223	6:42847355:42855039:+	RPL7L1
6:43515053:43515190:-	skipped	ENSG00000124571	6:43501090:43515418:-	XPO5
6:90962232:90962309:-	skipped	ENSG00000112182	6:90636247:91006461:-	BACH2
6:90981568:90981660:-	skipped	ENSG00000112182	6:90636247:91006461:-	BACH2
6:109421395:109421489:+	skipped	ENSG00000183137	6:109416312:109479691:+	CEP57L1
6:109450507:109450695:+	skipped	ENSG00000183137	6:109450506:109485113:+	CEP57L1
6:111805947:111806064:+	skipped	ENSG00000231889	6:111804713:111814206:+	TRAF3IP2-AS1
6:119281934:119282028:-	skipped	ENSG00000111879	6:119280993:119285925:-	FAM184A
6:145956381:145956622:-	skipped	ENSG00000112425	6:145954854:146008013:-	EPM2A
6:170013655:170013752:-	skipped	ENSG00000184465	6:169958416:170067371:-	WDR27
7:2584543:2584690:-	skipped	ENSG00000106009	7:2577510:2595361:-	BRAT1
7:5568792:5569294:-	skipped	ENSG00000075624	7:5566781:5570340:-	ACTB
7:6054777:6054983:+	skipped	ENSG00000106305	7:6048920:6054882:+	AIMP2
7:7607657:7607754:+	skipped	ENSG00000164654	7:7606591:7612556:+	MIOS
7:23157453:23157583:+	skipped	ENSG00000122550	7:23145376:23183520:+	KLHL7
7:44161881:44162058:-	skipped	ENSG00000106628	7:44155715:44163136:-	POLD2
7:64004684:64004810:-	skipped	ENSG00000173041	7:63980261:64023484:-	ZNF680
7:65551731:65551808:+	skipped	ENSG00000126522	7:65540820:65552147:+	ASL
7:72514920:72515008:+	skipped	ENSG00000174384	7:72507940:72515008:+	RP11-313P13.4

Table S1. Exons affected by SWI/SNF ATPase subunits

BRG1-wt

Affected exon coordinates	included/skipped	ENSEMBL gene ID	gene coordinates	Gene name
7:72957878:72957974:-	skipped	ENSG00000106635	7:72951488:72957931:-	BCL7B
7:72990871:72991031:-	skipped	ENSG00000106638	7:72983261:72993121:-	TBL2
7:74317004:74317092:+	skipped	ENSG00000123965	7:74306893:74322330:+	PMS2P5
7:99923314:99923402:-	skipped	ENSG00000078319	7:99918614:99939531:-	PMS2P1
7:102183972:102184144:-	skipped	ENSG00000168255	7:102178365:102184083:-	POLR2J3
7:102228681:102228892:-	skipped	ENSG00000105808	7:102222796:102234438:-	RASA4
7:102724477:102724509:+	skipped	ENSG00000170632	7:102715327:102740205:+	ARMC10
7:112428509:112428599:-	skipped	ENSG00000146802	7:112402436:112430647:-	TMEM168
7:116738667:116738869:+	skipped	ENSG00000004866	7:116654989:116769863:+	ST7
7:116739816:116739898:+	skipped	ENSG00000004866	7:116654989:116769863:+	ST7
7:127981172:127981273:-	skipped	ENSG00000106344	7:127975598:127983928:-	RBM28
7:129269919:129270009:+	skipped	ENSG00000106459	7:129251579:129273621:+	NRF1
7:130652148:130652276:-	skipped	ENSG00000231721	7:130628926:130668868:-	LINC-PINT
7:134620439:134620516:+	skipped	ENSG00000122786	7:134429002:134653153:+	CALD1
7:151854846:151855010:-	skipped	ENSG00000055609	7:151849983:151859249:-	KMT2C
8:12044209:12044342:-	skipped	ENSG00000186523	8:12042657:12051636:-	FAM86B1
8:19706673:19706750:+	skipped	ENSG00000104613	8:19694563:19709594:+	INTS10
8:27147673:27147695:-	skipped	ENSG00000104228	8:27142403:27168836:-	TRIM35
8:27954736:27954835:+	skipped	ENSG00000134014	8:27947189:27965481:+	ELP3
8:29931393:29931544:-	skipped	ENSG00000133872	8:29920527:29940723:-	TMEM66
8:37963549:37963657:+	skipped	ENSG00000129691	8:37963017:37968329:+	ASH2L
8:38251616:38251759:+	skipped	ENSG00000165046	8:38243820:38251952:+	LETM2
8:53598497:53598716:-	skipped	ENSG00000023287	8:53535015:53626992:-	RB1CC1
8:59472843:59474354:+	skipped	ENSG00000137575	8:59465482:59484860:+	SDCBP
8:74872000:74872053:-	skipped	ENSG00000154582	8:74851403:74884421:-	TCEB1
8:74876722:74876864:-	skipped	ENSG00000154582	8:74851403:74884421:-	TCEB1
8:91018264:91018505:+	skipped	ENSG00000104325	8:91013704:91018461:+	DECR1
8:91643780:91643935:-	skipped	ENSG00000180694	8:91634222:91658311:-	TMEM64
8:103270544:103270652:-	skipped	ENSG00000104517	8:103266173:103274214:-	UBR5
8:144902836:144902886:-	skipped	ENSG00000179950	8:144900119:144911214:-	PUF60
9:2651415:2651498:+	skipped	ENSG00000147852	9:2621833:2660053:+	VLDLR
9:16832242:16832348:-	skipped	ENSG00000173068	9:16409500:16870704:-	BNC2
9:33928370:33928610:-	skipped	ENSG00000137073	9:33921856:33928833:-	UBAP2
9:34319438:34319621:-	skipped	ENSG00000229207	9:34318404:34319653:+	SERPINH1P1
9:35743273:35743323:-	skipped	ENSG00000070610	9:35736862:35749225:-	GBA2
9:37903528:37903554:-	skipped	ENSG00000122696	9:37887600:37904107:-	SLC25A51
9:38573081:38573126:-	skipped	ENSG00000273170	9:38540565:38577204:-	ANKRD18A
9:72001475:72002599:+	skipped	ENSG00000135063	9:72000735:72006629:+	FAM189A2
9:95002925:95002973:-	skipped	ENSG00000196305	9:95002570:95005510:-	IARS
9:95591220:95591445:+	skipped	ENSG00000187984	9:95584503:95592045:+	ANKRD19P
9:99271955:99272071:-	skipped	ENSG00000081377	9:99264893:99329536:-	CDC14B
9:99277931:99278074:-	skipped	ENSG00000081377	9:99276784:99284885:-	CDC14B
9:124931924:124932035:+	skipped	ENSG00000185681	9:124931939:124937033:+	MORN5
9:130211889:130211987:-	skipped	ENSG00000197958	9:130209952:130213684:-	RPL12
9:130678687:130678773:-	skipped	ENSG00000136840	9:130670164:130679305:-	ST6GALNAC4
9:131035064:131035144:-	skipped	ENSG00000167110	9:131023450:131038274:-	GOLGA2
9:134379576:134379727:+	skipped	ENSG00000130714	9:134378288:134399193:+	POMT1
9:135144790:135144876:-	skipped	ENSG00000107290	9:135136742:135150769:-	SETX
X:18918788:18918817:-	skipped	ENSG00000044446	X:18912199:18919721:-	PHKA2
X:47057566:47057754:+	skipped	ENSG00000130985	X:47056597:47060361:+	UBA1
X:47081660:47081779:+	skipped	ENSG00000102225	X:47077258:47081779:+	CDK16
X:52929597:52929686:+	skipped	ENSG00000179304	X:52920645:52929617:+	FAM156B

Table S1. Exons affected by SWI/SNF ATPase subunits

BRG1-wt

Affected exon coordinates	included/skipped	ENSEMBL gene ID	gene coordinates	Gene name
X:62974184:62974556:-	skipped	ENSG00000131089	X:62854846:62974993:-	ARHGEF9
X:80457936:80458075:+	skipped	ENSG00000131171	X:80457441:80554046:+	SH3BGRL
X:100306633:100306722:-	skipped	ENSG00000188917	X:100278574:100307071:-	TRMT2B
X:129312967:129314017:+	skipped	ENSG00000134594	X:129305622:129318844:+	RAB33A
X:134482679:134482762:+	skipped	ENSG00000173275	X:134478720:134482954:+	ZNF449
X:154294181:154294332:-	skipped	ENSG00000182712	X:154289896:154299637:-	CMC4
1:18913:19139:-	included	ENSG00000227232	1:14362:29370:-	WASH7P
1:1326677:1326955:-	included	ENSG00000221978	1:1322501:1327029:-	CCNL2
1:1688178:1688321:-	included	ENSG00000008130	1:1683909:1690081:-	NADK
1:16719723:16719977:+	included	ENSG00000055070	1:16679069:16719850:+	SZRD1
1:22408215:22408287:+	included	ENSG00000070831	1:22379789:22413283:+	CDC42
1:25644554:25644762:-	included	ENSG00000117616	1:25568746:25664656:-	C1orf63
1:26152793:26152902:+	included	ENSG00000117640	1:26149486:26156288:+	MTFR1L
1:27440317:27440777:-	included	ENSG00000090020	1:27425305:27481401:-	SLC9A1
1:27736186:27736700:-	included	ENSG00000158195	1:27730729:27816669:-	WASF2
1:31733802:31734023:-	included	ENSG00000060688	1:31732519:31734669:-	SNRNP40
1:33290914:33291086:+	included	ENSG00000116497	1:33282367:33292090:+	S100PBP
1:38262416:38262492:+	included	ENSG00000185090	1:38259473:38266805:+	MANEAL
1:39902154:39902162:+	included	ENSG00000127603	1:39901297:39907961:+	MACF1
1:40919875:40919960:+	included	ENSG00000187801	1:40915773:40929386:+	ZFP69B
1:41232590:41232646:+	included	ENSG00000066136	1:41231761:41232603:+	NFYC
1:43315236:43315413:+	included	ENSG00000164011	1:43312279:43318148:+	ZNF691
1:44436578:44436861:+	included	ENSG00000132768	1:44435671:44437198:+	DPH2
1:46156646:46156782:+	included	ENSG00000159596	1:46153867:46159517:+	TMEM69
1:68151708:68151809:+	included	ENSG00000116717	1:68150743:68154021:+	GADD45A
1:76254844:76255041:+	included	ENSG00000137955	1:76251878:76260350:+	RABGGTB
1:85136872:85136991:-	included	ENSG00000117155	1:85109389:85156228:-	SSX2IP
1:85506693:85506860:-	included	ENSG00000055732	1:85483764:85514129:-	MCOLN3
1:89270074:89270217:+	included	ENSG00000065243	1:89149904:89301938:+	PKN2
1:89453935:89454034:-	included	ENSG00000137944	1:89401455:89458459:-	CCBL2
1:93676359:93676483:+	included	ENSG00000122483	1:93646325:93682236:+	CCDC18
1:110020440:110020631:+	included	ENSG00000143028	1:110009179:110022410:+	SYPL2
1:113247722:113248874:-	included	ENSG00000155366	1:113247261:113249749:-	RHOC
1:114267381:114267515:-	included	ENSG00000116793	1:114239823:114301777:-	PHTF1
1:146057587:146057734:-	included	ENSG00000152042	1:146032646:146082765:-	NBPF11
1:146397359:146397461:+	included	ENSG00000186275	1:146373545:146467638:+	NBPF12
1:148344640:148344742:-	included	ENSG00000203832	1:148341795:148346929:-	NBPF20
1:151001262:151001420:+	included	ENSG00000143363	1:150997217:151008187:+	PRUNE
1:153614719:153614905:+	included	ENSG00000160679	1:153606524:153618782:+	CHTOP
1:153615703:153615840:+	included	ENSG00000160679	1:153606524:153618782:+	CHTOP
1:153934696:153934826:-	included	ENSG00000143570	1:153931574:153936048:-	SLC39A1
1:154245812:154246010:+	included	ENSG00000143575	1:154244986:154248351:+	HAX1
1:154956318:154956542:+	included	ENSG00000160688	1:154955813:154960608:+	FLAD1
1:154962815:154963004:+	included	ENSG00000160688	1:154960505:154965587:+	FLAD1
1:155746186:155746272:-	included	ENSG00000116580	1:155736326:155746252:-	GON4L
1:155990057:155990137:-	included	ENSG00000163479	1:155978838:155990750:-	SSR2
1:202986884:202987047:+	included	ENSG00000163444	1:202984967:202992089:+	TMEM183A
1:205633613:205634014:-	included	ENSG00000158715	1:205626978:205649587:-	SLC45A3
1:207963598:207963690:+	included	ENSG00000117335	1:207925401:207968858:+	CD46
1:213061233:213061343:+	included	ENSG00000162769	1:213031596:213072705:+	FLVCR1
1:228681584:228681970:+	included	ENSG00000168159	1:228674761:228683467:+	RNF187
1:242012411:242012518:+	included	ENSG00000174371	1:242011268:242013762:+	EXO1

Table S1. Exons affected by SWI/SNF ATPase subunits

BRG1-wt

Affected exon coordinates	included/skipped	ENSEMBL gene ID	gene coordinates	Gene name
10:5777267:5777509:+	included	ENSG00000108021	10:5772624:5781776:+	FAM208B
10:6268155:6268328:+	included	ENSG00000170525	10:6263366:6275070:+	PFKFB3
10:27469882:27469995:+	included	ENSG00000120539	10:27443752:27475848:+	MASTL
10:30315032:30318795:-	included	ENSG00000165757	10:30301728:30348453:-	KIAA1462
10:30726105:30726335:+	included	ENSG00000107968	10:30723172:30728410:+	MAP3K8
10:31661947:31662102:+	included	ENSG00000148516	10:31608140:31676198:+	ZEB1
10:35426710:35426807:+	included	ENSG00000095794	10:35415718:35501053:+	CREM
10:45958599:45959444:-	included	ENSG00000165406	10:45950034:46030842:-	MARCH8
10:69408498:69408574:-	included	ENSG00000183230	10:69281599:69425410:-	CTNNA3
10:72083619:72083786:-	included	ENSG00000172731	10:72058728:72141670:-	LRRC20
10:88603163:88603267:+	included	ENSG00000107779	10:88516406:88692595:+	BMPR1A
10:97174251:97174619:-	included	ENSG00000095637	10:97158773:97175452:-	SORBS1
10:102738606:102738775:+	included	ENSG00000095539	10:102729274:102740677:+	SEMA4G
10:102743062:102744114:+	included	ENSG00000055950	10:102729214:102746953:-	MRPL43
10:103345619:103345913:-	included	ENSG00000166169	10:103338638:103347966:-	POLL
10:103364897:103364969:+	included	ENSG00000166171	10:103348044:103369425:+	DPCD
10:103368624:103368801:+	included	ENSG00000166171	10:103348044:103369425:+	DPCD
10:104860509:104860700:-	included	ENSG00000076685	10:104857728:104866407:-	NT5C2
10:120879853:120879964:+	included	ENSG00000119979	10:120863599:120896139:+	FAM45A
10:123658356:123658484:-	included	ENSG00000107669	10:123499938:123687551:-	ATE1
10:124746850:124747020:+	included	ENSG00000179988	10:124713896:124749906:+	PSTK
10:126100542:126100769:-	included	ENSG00000065154	10:126095924:126106762:-	OAT
10:135197464:135197716:+	included	ENSG00000148832	10:135192694:135205195:+	PAOX
11:836769:836843:+	included	ENSG00000177697	11:836062:838798:+	CD151
11:9801364:9801489:+	included	ENSG00000133812	11:9800700:9809520:-	SBF2
11:18312989:18313566:-	included	ENSG00000110756	11:18305339:18313489:-	HPS5
11:18314446:18314523:-	included	ENSG00000110756	11:18300222:18343721:-	HPS5
11:47434951:47435058:+	included	ENSG00000165915	11:47433852:47435431:+	SLC39A13
11:63996719:63996816:+	included	ENSG00000149761	11:63996602:63997488:+	NUDT22
11:64572506:64572670:-	included	ENSG00000133895	11:64570981:64577957:-	MEN1
11:65661485:65661592:-	included	ENSG00000175592	11:65659519:65667890:-	FOSL1
11:66391685:66393149:+	included	ENSG00000239306	11:66384052:66394818:+	RBM14
11:66410921:66411611:+	included	ENSG00000173933	11:66406087:66413940:+	RBM4
11:70197100:70197129:+	included	ENSG00000131626	11:70192020:70201818:+	PPFIA1
11:71809335:71809461:-	included	ENSG00000149357	11:71808337:71810304:-	LAMTOR1
11:71949086:71949219:+	included	ENSG00000165458	11:71944723:71950149:+	INPPL1
11:74644870:74644917:-	included	ENSG00000166435	11:74617397:74660065:-	XRRA1
11:82639819:82639980:+	included	ENSG00000165490	11:82611016:82639931:+	C11orf82
11:85979498:85979603:+	included	ENSG00000074266	11:85974986:85979572:+	EED
11:95512242:95512299:-	included	ENSG00000077458	11:95504689:95512383:-	FAM76B
11:103270391:103270600:+	included	ENSG00000187240	11:102980159:103350591:+	DYNC2H1
11:111624167:111624301:-	included	ENSG00000137713	11:111597631:111637106:-	PPP2R1B
11:112100931:112100953:+	included	ENSG00000150787	11:112100816:112104121:+	PTS
11:116746582:116746725:-	included	ENSG00000160584	11:116714117:116969137:-	SIK3
11:118897216:118897398:-	included	ENSG00000137700	11:118894823:118901616:-	SLC37A4
11:118897313:118897398:-	included	ENSG00000137700	11:118894823:118901616:-	SLC37A4
11:125333380:125333466:-	included	ENSG00000149557	11:125330136:125333436:-	FEZ1
11:126277994:126278089:+	included	ENSG00000110080	11:126273310:126284533:+	ST3GAL4
11:129993507:129993674:+	included	ENSG00000084234	11:129939731:130013417:+	APLP2
12:2994328:2994700:+	included	ENSG00000171792	12:2985423:2997334:+	RHNO1
12:2994449:2994658:+	included	ENSG00000171792	12:2985423:2997334:+	RHNO1
12:4665519:4665716:+	included	ENSG00000111247	12:4659680:4668848:+	RAD51AP1

Table S1. Exons affected by SWI/SNF ATPase subunits

BRG1-wt

Affected exon coordinates	included/skipped	ENSEMBL gene ID	gene coordinates	Gene name
12:6839835:6839986:+	included	ENSG00000111652	12:6832906:6841022:+	COPS7A
12:6959997:6960173:-	included	ENSG00000111665	12:6953956:6960433:-	CDCA3
12:7075074:7075079:-	included	ENSG00000215021	12:7074489:7076738:-	PHB2
12:9072352:9072513:+	included	ENSG00000111752	12:9066491:9075287:+	PHC1
12:27523062:27523163:+	included	ENSG00000029153	12:27485786:27576241:+	ARNTL2
12:31237517:31237603:+	included	ENSG00000013573	12:31231453:31242064:+	DDX11
12:31237903:31238060:+	included	ENSG00000013573	12:31231453:31242064:+	DDX11
12:53421566:53421703:+	included	ENSG00000063046	12:53399941:53435993:+	EIF4B
12:53621146:53621471:-	included	ENSG00000172819	12:53621157:53625998:-	RARG
12:53849669:53849785:+	included	ENSG00000197111	12:53846600:53849734:+	PCBP2
12:54581603:54581689:-	included	ENSG00000123415	12:54575236:54582724:-	SMUG1
12:54675579:54675725:+	included	ENSG00000135486	12:54673976:54680871:+	HNRNPA1
12:56223273:56223420:-	included	ENSG00000135392	12:56214743:56224565:-	DNAJC14
12:56554410:56554454:+	included	ENSG00000092841	12:56551944:56555358:+	MYL6
12:58197307:58197452:-	included	ENSG00000135407	12:58197078:58200272:-	AVIL
12:70688017:70688074:+	included	ENSG00000111596	12:70671911:70726626:+	CNOT2
12:95650326:95650398:+	included	ENSG00000028203	12:95611521:95650957:+	VEZT
12:95650926:95651015:+	included	ENSG00000028203	12:95611521:95650957:+	VEZT
12:108936546:108936627:-	included	ENSG00000075856	12:108923986:108954936:-	SART3
12:110019200:110019355:+	included	ENSG00000110921	12:110019283:110024904:+	MVK
12:110930801:110931036:-	included	ENSG00000111237	12:110929535:110934022:-	VPS29
12:118839788:118839809:+	included	ENSG00000111707	12:118814184:118855836:+	SUDS3
12:120636657:120636803:-	included	ENSG00000089157	12:120636160:120638635:-	RPLP0
12:120653733:120653893:-	included	ENSG00000089159	12:120652638:120653809:-	PXN
12:122831922:122832026:-	included	ENSG00000130779	12:122817531:122879974:-	CLIP1
12:122967826:122967891:-	included	ENSG00000033030	12:122962384:122968709:-	ZCCHC8
12:123687797:123687922:-	included	ENSG00000051825	12:123637079:123706441:-	MPHOSPH9
13:50237193:50237313:-	included	ENSG00000123179	13:50234858:50265574:-	EBPL
13:111546456:111546549:-	included	ENSG00000088448	13:111545374:111553086:-	ANKRD10
13:111552877:111553041:-	included	ENSG00000088448	13:111545374:111553086:-	ANKRD10
14:19655734:19655871:+	included	ENSG00000225210	14:19650020:19656210:+	AL589743.1
14:19919492:19919629:-	included	ENSG00000244306	14:19918475:19919581:-	CTD-2314B22.3
14:20923737:20923932:+	included	ENSG00000100823	14:20923349:20925927:+	APEX1
14:21491023:21491064:-	included	ENSG00000165795	14:21484921:21493982:-	NDRG2
14:21679565:21679722:-	included	ENSG00000092199	14:21679095:21680161:-	HNRNPC
14:21990967:21993065:-	included	ENSG00000165821	14:21989672:21994634:-	SALL2
14:24025952:24026513:+	included	ENSG00000157306	14:24025196:24028118:+	RP11-66N24.4
14:24656873:24657019:-	included	ENSG00000196497	14:24656696:24657275:-	IPO4
14:24675715:24676715:+	included	ENSG00000139908	14:24674902:24677454:+	TSSK4
14:24910884:24911001:-	included	ENSG00000100445	14:24908971:24911982:-	SDR39U1
14:52470958:52470960:+	included	ENSG00000087302	14:52470729:52471410:+	C14orf166
14:65392725:65392798:+	included	ENSG00000258289	14:65390727:65397655:+	CHURC1
14:68271934:68272022:-	included	ENSG00000072121	14:68213236:68283306:-	ZFYVE26
14:69259599:69259787:-	included	ENSG00000185650	14:69255321:69261453:-	ZFP36L1
14:77250036:77250194:-	included	ENSG00000258301	14:77248082:77253067:-	RP11-488C13.5
14:91044450:91044652:-	included	ENSG00000165914	14:91007529:91084367:-	TTC7B
14:100841620:100841883:-	included	ENSG00000140105	14:100828039:100841810:-	WARS
15:23029840:23029966:-	included	ENSG00000140157	15:23019818:23034378:-	NIPA2
15:28878736:28878886:+	included	ENSG00000206149	15:28878244:28887539:+	HERC2P9
15:34653601:34653733:-	included	ENSG00000176454	15:34653112:34655091:-	LPCAT4
15:41191342:41193212:+	included	ENSG00000104142	15:41186627:41196173:+	VPS18
15:42820460:42820618:+	included	ENSG00000092531	15:42820140:42823849:+	SNAP23

Table S1. Exons affected by SWI/SNF ATPase subunits

BRG1-wt

Affected exon coordinates	included/skipped	ENSEMBL gene ID	gene coordinates	Gene name
15:56960195:56960439:-	included	ENSG00000137871	15:56922378:56974614:-	ZNF280D
15:65748050:65748179:-	included	ENSG00000074603	15:65738974:65759530:-	DPP8
15:67687629:67687901:+	included	ENSG00000103599	15:67664857:67692564:+	IQCH
15:73047897:73047995:-	included	ENSG00000159322	15:73043709:73048608:-	ADPGK
15:74324913:74325056:+	included	ENSG00000140464	15:74324735:74325546:+	PML
15:75198619:75198706:-	included	ENSG00000178761	15:75192327:75199462:-	FAM219B
15:75198665:75198706:-	included	ENSG00000178761	15:75192327:75199462:-	FAM219B
15:75336727:75336855:+	included	ENSG00000138621	15:75335615:75342079:+	PPCDC
15:84873352:84873443:-	included	ENSG00000225151	15:84869931:84874024:-	GOLGA2P7
15:85752430:85752521:-	included	ENSG00000229212	15:85749008:85753102:-	RP11-561C5.4
15:89079543:89079612:-	included	ENSG00000140543	15:89054789:89089876:-	DET1
15:89442887:89443042:-	included	ENSG00000140545	15:89441915:89456610:-	MFGE8
15:90229662:90229777:-	included	ENSG00000166821	15:90220994:90234013:-	PEX11A
15:91527923:91528055:-	included	ENSG00000198901	15:91509269:91537725:-	PRC1
16:258600:258663:-	included	ENSG00000007392	16:239096:259772:-	LUC7L
16:685281:685340:-	included	ENSG00000130731	16:684428:686342:-	C16orf13
16:1876508:1876603:-	included	ENSG00000063854	16:1868515:1876803:-	HAGH
16:2012498:2012657:-	included	ENSG00000140988	16:2012052:2014861:-	RPS2
16:2305586:2305727:-	included	ENSG00000205937	16:2303123:2306867:-	RNPS1
16:2722708:2722977:-	included	ENSG00000260565	16:2722326:2723445:-	ERVK13-1
16:3021275:3021379:+	included	ENSG00000127564	16:3018102:3030417:-	PKMYT1
16:4393208:4393292:-	included	ENSG00000217930	16:4391652:4393694:-	PAM16
16:4504812:4504928:+	included	ENSG00000103423	16:4475805:4506776:+	DNAJA3
16:4851268:4851322:-	included	ENSG00000067836	16:4851029:4852881:-	ROGDI
16:11647389:11647604:-	included	ENSG00000189067	16:11643215:11650300:-	LITAF
16:16165499:16165586:+	included	ENSG00000103222	16:16043433:16236931:+	ABCC1
16:16232221:16232415:+	included	ENSG00000103222	16:16043433:16236931:+	ABCC1
16:21430956:21431087:-	included	ENSG00000169246	16:21430989:21436657:-	NPIPB3
16:21433127:21433464:-	included	ENSG00000169246	16:21430989:21436657:-	NPIPB3
16:21863091:21863367:-	included	ENSG00000185864	16:21858802:21869003:-	NPIPB4
16:21863236:21863367:-	included	ENSG00000185864	16:21858802:21869003:-	NPIPB4
16:22528086:22528423:+	included	ENSG00000243716	16:22516171:22538951:+	NPIPB5
16:22530459:22530735:+	included	ENSG00000243716	16:22516171:22538951:+	NPIPB5
16:24939005:24939053:-	included	ENSG00000140750	16:24930705:25026652:-	ARHGAP17
16:29511319:29511595:-	included	ENSG00000169203	16:29496010:29517141:-	RP11-231C14.4
16:29511464:29511595:-	included	ENSG00000169203	16:29496010:29517141:-	RP11-231C14.4
16:30012533:30015978:+	included	ENSG00000169592	16:30007564:30012896:+	INO80E
16:30251287:30251563:-	included	ENSG00000198064	16:30246819:30257150:-	RP11-347C12.1
16:31712984:31713179:+	included	ENSG00000131797	16:31711910:31718758:+	CLUHP3
16:32163770:32163966:-	included	ENSG00000230267	16:32163039:32164308:-	HERC2P4
16:32265142:32265308:+	included	ENSG00000260575	16:32261940:32265743:-	RP11-56L13.7
16:32686098:32686386:-	included	ENSG00000260974	16:32686357:32690152:+	RP11-586K12.8
16:33263182:33263470:+	included	ENSG00000261509	16:33261514:33264716:+	TP53TG3B
16:66608843:66609040:+	included	ENSG00000254788	16:66586515:66613038:+	CKLF-CMTM1
16:67988007:67988122:-	included	ENSG00000124067	16:67977376:68002597:-	SLC12A4
16:74383640:74383757:-	included	ENSG00000214331	16:74366299:74402059:-	RP11-252A24.2
16:84219086:84219189:-	included	ENSG00000103168	16:84215101:84220595:-	TAF1C
17:4885384:4885470:-	included	ENSG00000108509	17:4883730:4890927:-	CAMTA2
17:5391516:5391909:+	included	ENSG00000167842	17:5389604:5392557:+	MIS12
17:7296463:7296683:-	included	ENSG00000187838	17:7293045:7297889:-	TMEM256-PLSCR3
17:8079277:8079344:-	included	ENSG00000179029	17:8077316:8079706:-	TMEM107
17:8193933:8193996:-	included	ENSG00000125434	17:8192177:8198154:-	SLC25A35

Table S1. Exons affected by SWI/SNF ATPase subunits

BRG1-wt

Affected exon coordinates	included/skipped	ENSEMBL gene ID	gene coordinates	Gene name
17:15457079:15457143:-	included	ENSG00000259024	17:15339337:15466875:-	TVP23C-CDRT4
17:15990486:15990659:-	included	ENSG00000141027	17:15932470:16118863:-	NCOR1
17:17726832:17726921:-	included	ENSG00000072310	17:17722340:17726932:-	SREBF1
17:33341759:33341782:-	included	ENSG00000092871	17:33333008:33416338:-	RFFL
17:35880282:35880317:-	included	ENSG00000006114	17:35879112:35897580:-	SYNRG
17:36291899:36292527:+	included	ENSG00000185128	17:36283970:36294912:+	TBC1D3F
17:36339906:36340198:-	included	ENSG00000197681	17:36337710:36347030:-	TBC1D3
17:36361697:36361804:-	included	ENSG00000174093	17:36344879:36375123:-	RP11-1407O15.2
17:36971878:36971994:-	included	ENSG00000108296	17:36963052:36981608:-	CWC25
17:37876040:37876087:+	included	ENSG00000141736	17:37873595:37876550:+	ERBB2
17:37883548:37883800:+	included	ENSG00000141736	17:37844166:37884614:+	ERBB2
17:43545575:43545959:-	included	ENSG00000225190	17:43517528:43565224:-	PLEKHM1
17:47714121:47714171:-	included	ENSG00000121067	17:47676245:47755472:-	SPOP
17:48626647:48626848:+	included	ENSG00000006282	17:48624513:48626690:+	SPATA20
17:57208642:57208728:-	included	ENSG00000182628	17:57187311:57232630:-	SKA2
17:60629663:60629758:+	included	ENSG00000146872	17:60555426:60637395:+	TLK2
17:61784607:61784778:-	included	ENSG00000266173	17:61780191:61819143:-	STRADA
17:73887894:73887959:-	included	ENSG00000141569	17:73876415:73888384:-	TRIM65
17:76392389:76392466:+	included	ENSG00000087157	17:76374729:76394349:+	PGS1
17:79781288:79781389:-	included	ENSG00000215621	17:79780292:79784533:-	AC174470.1
17:79892202:79892365:-	included	ENSG00000183010	17:79890259:79895204:-	PYCR1
17:79977517:79977570:-	included	ENSG00000169689	17:79976577:79981983:-	STRA13
17:80407307:80407832:-	included	ENSG00000178927	17:80400464:80408646:-	C17orf62
18:9204474:9204542:+	included	ENSG00000101745	18:9182379:9211619:+	ANKRD12
18:33719382:33719576:+	included	ENSG00000134759	18:33718769:33725321:+	ELP2
18:34267091:34267141:+	included	ENSG00000134775	18:33877676:34360018:+	FHOD3
18:34340571:34340745:+	included	ENSG00000134775	18:33877676:34360018:+	FHOD3
18:45396846:45396935:-	included	ENSG00000175387	18:45357921:45457512:-	SMAD2
18:47014870:47014936:-	included	ENSG00000215472	18:47008050:47017956:-	RPL17-C18orf32
18:54293393:54293497:-	included	ENSG00000091164	18:54264438:54305876:-	TXNL1
19:3542774:3542975:-	included	ENSG00000183397	19:3539151:3544028:+	C19orf71
19:4501203:4501329:+	included	ENSG00000167674	19:4496329:4502116:+	HDGFRP2
19:9923907:9923950:-	included	ENSG00000127452	19:9921571:9924089:-	FBXL12
19:11144443:11144541:+	included	ENSG00000127616	19:11141488:11152142:+	SMARCA4
19:11629890:11630008:-	included	ENSG00000130159	19:11616730:11639989:-	ECSIT
19:12463802:12463928:-	included	ENSG00000198342	19:12460184:12476447:-	ZNF442
19:13994121:13994213:-	included	ENSG00000132016	19:13993160:13999193:-	C19orf57
19:19102149:19102362:-	included	ENSG00000064607	19:19101701:19102427:-	SUGP2
19:37063235:37063294:-	included	ENSG00000186020	19:37034517:37064190:-	ZNF529
19:37945736:37945975:-	included	ENSG00000196437	19:37902056:37958339:-	ZNF569
19:39663558:39664016:+	included	ENSG00000130669	19:39616409:39664443:+	PAK4
19:41811551:41811772:+	included	ENSG00000105323	19:41768400:41813503:+	HNRNPUL1
19:46269608:46270413:-	included	ENSG00000177045	19:46268650:46272113:-	SIX5
19:50518989:50519050:-	included	ENSG00000105053	19:50479723:50528643:-	VRK3
19:54372944:54373092:+	included	ENSG00000179820	19:54369476:54377510:+	MYADM
19:54373008:54373092:+	included	ENSG00000179820	19:54369476:54377510:+	MYADM
19:54682482:54682658:-	included	ENSG00000125505	19:54677112:54684507:-	MBOAT7
19:58003481:58003579:+	included	ENSG00000105136	19:57999078:58005458:+	ZNF419
19:58151280:58151435:+	included	ENSG00000121417	19:58141760:58153549:+	ZNF211
2:233101:233229:-	included	ENSG00000035115	2:230378:234231:-	SH3YL1
2:27595907:27595980:+	included	ENSG00000115234	2:27593388:27599995:+	SNX17
2:38976040:38976488:-	included	ENSG00000115875	2:38971858:38976706:-	SRSF7

Table S1. Exons affected by SWI/SNF ATPase subunits

BRG1-wt

Affected exon coordinates	included/skipped	ENSEMBL gene ID	gene coordinates	Gene name
2:39998655:39998780:-	included	ENSG00000138050	2:39996042:40006379:-	THUMPD2
2:43787373:43787524:-	included	ENSG00000115970	2:43779206:43819559:-	THADA
2:55526975:55527055:-	included	ENSG00000115355	2:55515223:55536343:-	CCDC88A
2:64069673:64069733:+	included	ENSG00000169764	2:64068087:64083567:+	UGP2
2:73198699:73198814:-	included	ENSG00000144040	2:73171731:73208246:-	SFXN5
2:106497784:106498505:+	included	ENSG00000071051	2:106433014:106509632:+	NCK2
2:111907621:111907724:+	included	ENSG00000153094	2:111878490:111909428:+	BCL2L11
2:131897740:131897848:+	included	ENSG00000115762	2:131862419:131905370:+	PLEKHB2
2:172314882:172314987:+	included	ENSG00000115827	2:172290726:172338668:+	DCAF17
2:178082433:178082573:+	included	ENSG00000170144	2:178081253:178084415:+	HNRNPA3
2:179337756:179337869:-	included	ENSG00000079150	2:179328390:179343285:-	FKBP7
2:179988442:179988556:-	included	ENSG00000187231	2:179974048:179989194:-	SESTD1
2:182785324:182785377:+	included	ENSG00000138434	2:182783587:182794404:+	SSFA2
2:192265475:192265561:+	included	ENSG00000128641	2:192110001:192290115:+	MYO1B
2:203075458:203075529:-	included	ENSG00000116030	2:203070902:203103331:-	SUMO1
2:203777757:203777837:+	included	ENSG00000138380	2:203776967:203807547:+	CARF
2:219516424:219517023:-	included	ENSG00000115568	2:219515223:219521126:-	ZNF142
2:223752548:223752606:+	included	ENSG00000123983	2:223725651:223809357:+	ACSL3
2:232656443:232656518:+	included	ENSG00000144524	2:232653398:232660855:+	COPS7B
2:236839409:236839567:+	included	ENSG00000157985	2:236792038:236945341:+	AGAP1
2:237995375:237995439:+	included	ENSG00000198612	2:237994524:238007261:+	COPS8
20:330282:330476:+	included	ENSG00000125841	20:327748:334146:+	NRSN2
20:13597504:13597770:-	included	ENSG00000089123	20:13567896:13619550:-	TASP1
20:18278629:18278706:+	included	ENSG00000125846	20:18269120:18297640:+	ZNF133
20:42159438:42159529:+	included	ENSG00000185513	20:42157296:42163043:+	L3MBTL1
20:42846007:42846322:+	included	ENSG00000223891	20:42839599:42854667:+	OSER1-AS1
20:45867640:45867882:-	included	ENSG00000101040	20:45837858:45984401:-	ZMYND8
20:48700666:48700791:-	included	ENSG00000244687	20:48697660:48732491:-	UBE2V1
20:49458303:49458437:+	included	ENSG00000124243	20:49411430:49493714:+	BCAS4
20:50407872:50408891:-	included	ENSG00000101115	20:50408606:50409617:-	SALL4
20:61833639:61835159:-	included	ENSG00000149658	20:61826780:61847586:-	YTHDF1
20:62333182:62333252:-	included	ENSG00000101246	20:62329995:62339355:-	ARFRP1
20:62562462:62562535:+	included	ENSG00000101152	20:62526517:62562472:+	DNAJC5
20:62657292:62657411:+	included	ENSG00000196700	20:62588054:62669938:-	ZNF512B
21:38466305:38466390:+	included	ENSG00000182670	21:38463626:38469185:+	TTC3
21:46604389:46604508:+	included	ENSG00000197381	21:46591556:46646475:+	ADARB1
21:46685937:46686142:-	included	ENSG00000186866	21:46685288:46686290:-	POFUT2
22:24381700:24381787:-	included	ENSG00000184674	22:24379873:24384271:-	GSTT1
22:24939810:24940051:-	included	ENSG00000138867	22:24936499:24944000:-	GUCD1
22:28394592:28394826:+	included	ENSG00000235954	22:28393564:28394770:+	TTC28-AS1
22:29706817:29706988:+	included	ENSG00000185340	22:29706268:29707097:+	GAS2L1
22:30419446:30419472:+	included	ENSG00000100330	22:30418451:30421952:+	MTMR3
22:30823109:30823390:+	included	ENSG00000242114	22:30821517:30825045:+	MTFP1
22:31368034:31368158:+	included	ENSG00000253352	22:31366662:31372049:+	TUG1
22:40760884:40761060:+	included	ENSG00000239900	22:40742506:40763008:+	ADSL
22:51012237:51012350:-	included	ENSG00000205560	22:51007289:51016449:-	CPT1B
3:9739395:9739550:+	included	ENSG00000163719	3:9710426:9744071:+	MTMR14
3:9796388:9796569:+	included	ENSG00000114026	3:9791627:9808421:+	OGG1
3:9876365:9876591:+	included	ENSG00000214021	3:9870846:9877087:+	TTLL3
3:11849307:11849369:-	included	ENSG00000144559	3:11831915:11851533:-	TAMM41
3:15674098:15674373:+	included	ENSG00000169814	3:15642847:15677122:+	BTD
3:25773810:25773974:-	included	ENSG00000151092	3:25760765:25775475:-	NGLY1

Table S1. Exons affected by SWI/SNF ATPase subunits

BRG1-wt

Affected exon coordinates	included/skipped	ENSEMBL gene ID	gene coordinates	Gene name
3:28383003:28383087:-	included	ENSG00000163512	3:28380036:28389968:-	AZI2
3:37090008:37090100:+	included	ENSG00000076242	3:37034822:37092409:+	MLH1
3:43341246:43341307:+	included	ENSG00000163788	3:43328003:43390330:+	SNRK
3:47465423:47465535:-	included	ENSG00000114650	3:47455202:47517603:-	SCAP
3:49129830:49129889:-	included	ENSG00000198218	3:49067139:49131796:-	QRICH1
3:49154492:49154794:-	included	ENSG00000172046	3:49153013:49158251:-	USP19
3:49212495:49212596:+	included	ENSG00000185909	3:49209043:49213917:+	KLHDC8B
3:50331063:50331152:-	included	ENSG00000186792	3:50330261:50336290:-	HYAL3
3:52130585:52130728:-	included	ENSG00000164087	3:52109268:52188428:-	POC1A
3:52716108:52716249:-	included	ENSG00000163939	3:52713597:52719594:-	PBRM1
3:52783708:52783845:-	included	ENSG00000114904	3:52780165:52786086:-	NEK4
3:58127585:58127656:+	included	ENSG00000136068	3:58117804:58157978:+	FLNB
3:78696779:78696805:-	included	ENSG00000169855	3:78655957:78719690:-	ROBO1
3:97510615:97510670:+	included	ENSG00000113966	3:97503860:97517379:+	ARL6
3:100030677:100030721:+	included	ENSG00000036054	3:99979844:100044095:+	TBC1D23
3:113146034:113146186:-	included	ENSG00000243849	3:113122844:113152839:+	WDR52-AS1
3:121438493:121438615:-	included	ENSG00000173230	3:121382045:121468602:-	GOLGB1
3:127294592:127294652:-	included	ENSG00000163870	3:127291911:127299280:-	TPRA1
3:129185733:129185909:+	included	ENSG00000163913	3:129168713:129198760:+	IFT122
3:141724283:141724386:-	included	ENSG00000114126	3:141697429:141809680:-	TFDP2
3:146254327:146254352:-	included	ENSG00000188313	3:146232966:146262651:-	PLSCR1
3:152165514:152165562:+	included	ENSG00000152601	3:152132846:152173366:+	MBNL1
3:195449372:195449689:+	included	ENSG00000176945	3:195447752:195460422:+	MUC20
3:195612284:195612414:-	included	ENSG00000061938	3:195611715:195619472:-	TNK2
3:197417945:197418019:-	included	ENSG00000145016	3:197398263:197476570:-	KIAA0226
4:667701:667755:-	included	ENSG00000169020	4:666224:667910:-	ATP5I
4:1729435:1730514:+	included	ENSG00000013810	4:1723261:1729959:+	TACC3
4:4307865:4307911:+	included	ENSG00000168826	4:4291923:4323512:+	ZBTB49
4:8477499:8481260:+	included	ENSG00000155275	4:8475033:8477692:+	TRMT44
4:13574326:13574466:-	included	ENSG00000038219	4:13571409:13579825:-	BOD1L1
4:38054727:38054846:+	included	ENSG00000065882	4:38051377:38056663:+	TBC1D1
4:89912124:89912301:-	included	ENSG00000138640	4:89647105:89978323:-	FAM13A
4:130030462:130030513:+	included	ENSG00000151470	4:130014471:130037795:+	C4orf33
4:152021637:152021740:+	included	ENSG00000145425	4:152020724:152025804:+	RPS3A
4:159754953:159755042:+	included	ENSG00000052795	4:159690345:159757810:+	FNIP2
5:306717:306875:+	included	ENSG00000249915	5:271735:315089:+	PDCD6
5:311408:311517:+	included	ENSG00000249915	5:271735:315089:+	PDCD6
5:868722:869234:-	included	ENSG00000028310	5:868816:876318:-	BRD9
5:884053:884185:-	included	ENSG00000028310	5:863850:889814:-	BRD9
5:10683641:10683683:-	included	ENSG00000112977	5:10679341:10761384:-	DAP
5:19981169:19981287:-	included	ENSG00000145526	5:19473140:19988339:-	CDH18
5:31521230:31521322:-	included	ENSG00000113360	5:31493315:31526527:-	DROSHA
5:37203426:37203572:-	included	ENSG00000197603	5:37107079:37213724:-	C5orf42
5:38949458:38949529:-	included	ENSG00000164327	5:38938020:39074510:-	RICTOR
5:54456158:54456224:+	included	ENSG00000164294	5:54455994:54459959:+	GPX8
5:56532940:56532999:+	included	ENSG00000062194	5:56527129:56533316:+	GPBP1
5:65451893:65454760:+	included	ENSG00000153914	5:65454527:65458064:+	SREK1
5:68551287:68551355:+	included	ENSG00000134058	5:68530667:68568731:+	CDK7
5:69521435:69521622:-	included	ENSG00000253816	5:69515744:69554757:-	RP11-1415C14.3
5:74026085:74026223:-	included	ENSG00000164347	5:74026084:74029213:-	GFM2
5:76745585:76745685:-	included	ENSG00000164253	5:76726757:76788421:-	WDR41
5:81272012:81272146:+	included	ENSG00000152348	5:81267844:81283498:+	ATG10

Table S1. Exons affected by SWI/SNF ATPase subunits

BRG1-wt

Affected exon coordinates	included/skipped	ENSEMBL gene ID	gene coordinates	Gene name
5:92774409:92774447:-	included	ENSG00000237187	5:92746723:92774443:-	NR2F1-AS1
5:122930733:122930828:+	included	ENSG00000151292	5:122847792:122952465:+	CSNK1G3
5:133912458:133912586:+	included	ENSG00000043143	5:133909446:133915165:+	JADE2
5:135488364:135488447:+	included	ENSG00000113658	5:135483108:135489370:+	SMAD5
5:138268267:138268401:+	included	ENSG00000044115	5:138266326:138269851:+	CTNNA1
5:149927780:149927950:+	included	ENSG00000070614	5:149877339:149933276:+	NDST1
5:154173360:154173560:+	included	ENSG00000155506	5:154172292:154181569:+	LARP1
5:159535552:159535621:-	included	ENSG00000170234	5:159518348:159546430:-	PWWP2A
5:170588411:170588470:+	included	ENSG00000204764	5:170570275:170632611:+	RANBP17
5:176958283:176958522:-	included	ENSG00000146067	5:176952054:176958835:-	FAM193B
5:176974169:176974229:-	included	ENSG00000146067	5:176952197:176981459:-	FAM193B
5:177637133:177637273:+	included	ENSG00000175309	5:177635623:177649477:-	PHYKPL
5:179274978:179275066:-	included	ENSG00000161010	5:179274356:179285808:-	C5orf45
5:180666067:180666177:-	included	ENSG00000204628	5:180663908:180670916:-	GNB2L1
6:7246657:7247454:+	included	ENSG00000124782	6:7107829:7249507:+	RREB1
6:26091138:26091332:+	included	ENSG00000010704	6:26087508:26095445:+	HFE
6:26091542:26091817:+	included	ENSG00000010704	6:26087508:26095445:+	HFE
6:26094414:26094446:+	included	ENSG00000010704	6:26087508:26095445:+	HFE
6:28239632:28240564:+	included	ENSG00000197062	6:28234787:28245974:+	RP5-874C20.3
6:30295424:30295473:+	included	ENSG00000204599	6:30294255:30297528:+	TRIM39
6:30657802:30658020:-	included	ENSG00000137404	6:30655823:30658500:-	NRM
6:32939372:32940704:+	included	ENSG00000204256	6:32936941:32939918:+	BRD2
6:33281471:33281641:-	included	ENSG00000231925	6:33280790:33281732:-	TAPBP
6:41048550:41048636:+	included	ENSG00000124596	6:41034677:41065497:-	OARD1
6:44224079:44224233:-	included	ENSG00000157593	6:44221832:44225291:-	SLC35B2
6:46137790:46137851:-	included	ENSG00000112796	6:46126923:46138708:-	ENPP5
6:54001513:54003084:+	included	ENSG00000146147	6:53964362:54001623:+	MLIP
6:56333780:56333797:-	included	ENSG00000151914	6:56322857:56334754:-	DST
6:58275636:58276289:-	included	ENSG00000215190	6:58272360:58276677:-	LINC00680
6:58276137:58276289:-	included	ENSG00000215190	6:58272360:58276677:-	LINC00680
6:75949993:75950101:-	included	ENSG00000112695	6:75947392:75953644:-	COX7A2
6:97717983:97718159:-	included	ENSG00000146263	6:97678144:97731064:-	MMS22L
6:109466422:109466584:+	included	ENSG00000183137	6:109450506:109485113:+	CEP57L1
6:109797966:109798133:-	included	ENSG00000112365	6:109783796:109804440:-	ZBTB24
6:127652975:127653012:-	included	ENSG00000093144	6:127609856:127664020:-	ECHDC1
6:138763120:138763251:-	included	ENSG00000135540	6:138743179:138893677:-	NHSL1
6:158922710:158925210:+	included	ENSG00000130338	6:158733691:158932860:+	TULP4
6:161574378:161575342:-	included	ENSG00000026652	6:161551010:161695057:-	AGPAT4
7:889560:889670:+	included	ENSG00000164828	7:889437:892544:+	SUN1
7:6052310:6052771:+	included	ENSG00000106305	7:6048920:6054882:+	AIMP2
7:23637586:23637670:+	included	ENSG00000169193	7:23637034:23641839:+	CCDC126
7:25164309:25164463:-	included	ENSG00000172115	7:25159709:25164980:-	CYCS
7:25219281:25219419:-	included	ENSG00000153790	7:25174315:25219973:-	C7orf31
7:45025620:45025696:-	included	ENSG00000232956	7:45023445:45026267:-	SNHG15
7:51240153:51240227:-	included	ENSG00000106078	7:51083908:51261132:-	COBL
7:56146057:56146201:+	included	ENSG00000129103	7:56131694:56147817:+	SUMF2
7:64291318:64291454:+	included	ENSG00000197008	7:64254765:64292580:+	ZNF138
7:72304344:72304508:+	included	ENSG00000225648	7:72300003:72307217:+	SBDSP1
7:72510219:72510331:+	included	ENSG00000174384	7:72507940:72515008:+	RP11-313P13.4
7:73151259:73151550:-	included	ENSG00000106077	7:73150423:73153111:-	ABHD11
7:97598317:97598364:-	included	ENSG00000243554	7:97598316:97601566:-	AC004967.7
7:99117792:99117927:+	included	ENSG00000196652	7:99102273:99131407:+	ZKSCAN5

Table S1. Exons affected by SWI/SNF ATPase subunits

BRG1-wt

Affected exon coordinates	included/skipped	ENSEMBL gene ID	gene coordinates	Gene name
7:99168883:99169002:+	included	ENSG00000197343	7:99156200:99172029:+	ZNF655
7:99747122:99747202:+	included	ENSG00000188186	7:99746529:99751826:+	LAMTOR4
7:99950833:99950893:+	included	ENSG00000272752	7:99948878:99955915:+	STAG3L5P-PVRIG2P-PILRB
7:102036424:102036984:+	included	ENSG00000128563	7:102004318:102045072:+	PRKRIP1
7:102131433:102131570:-	included	ENSG00000170667	7:102122891:102133758:-	RASA4B
7:106897177:106897239:-	included	ENSG00000164597	7:106876999:106899065:-	COG5
7:129736761:129736847:+	included	ENSG00000128607	7:129710389:129760609:+	KLHDC10
8:8647834:8647944:-	included	ENSG00000147324	8:8640863:8751155:-	MFHAS1
8:12045581:12045682:-	included	ENSG00000186523	8:12042657:12051636:-	FAM86B1
8:17100951:17101131:-	included	ENSG00000198791	8:17089078:17104387:-	CNOT7
8:21996152:21996306:-	included	ENSG00000168476	8:21995532:21999464:-	REEP4
8:22396982:22397011:+	included	ENSG00000120910	8:22298595:22398638:+	PPP3CC
8:30941215:30941295:+	included	ENSG00000165392	8:30891316:31031285:+	WRN
8:38287200:38287466:-	included	ENSG00000077782	8:38282200:38287520:-	FGFR1
8:57080587:57080945:-	included	ENSG00000181690	8:57073462:57123838:-	PLAG1
8:101964157:101964536:-	included	ENSG00000164924	8:101960823:101964847:-	YWHAZ
8:141889570:141889736:-	included	ENSG00000169398	8:141856757:141944659:-	PTK2
8:144906483:144906569:-	included	ENSG00000179950	8:144900119:144911214:-	PUF60
9:19026:19252:-	included	ENSG00000181404	9:14510:29739:-	XXyac-YRM2039.2
9:13143465:13143563:-	included	ENSG00000107186	9:13138046:13165506:-	MPDZ
9:16738358:16738483:-	included	ENSG00000173068	9:16409500:16870704:-	BNC2
9:34336226:34336339:+	included	ENSG00000164978	9:34329503:34343709:+	NUDT2
9:34636994:34637086:-	included	ENSG00000147955	9:34635806:34637680:-	SIGMAR1
9:88293250:88293313:-	included	ENSG00000135049	9:88272352:88307707:-	AGTPBP1
9:90585483:90585545:-	included	ENSG00000156345	9:90581355:90589600:-	CDK20
9:94877204:94877348:-	included	ENSG00000090054	9:94870082:94877664:-	SPTLC1
9:103191788:103191944:+	included	ENSG00000066697	9:103189437:103213511:+	MSANTD3
9:116093264:116093396:-	included	ENSG00000148225	9:116075501:116102562:-	WDR31
9:125833691:125833780:+	included	ENSG00000011454	9:125703111:125865547:+	RABGAP1
9:127828269:127828337:-	included	ENSG00000173611	9:127704886:127905775:-	SCAI
9:128414396:128414536:-	included	ENSG00000119487	9:128322061:128419989:-	MAPKAP1
9:129571072:129571194:+	included	ENSG00000169155	9:129567284:129600489:+	ZBTB43
9:134369792:134369873:+	included	ENSG00000130723	9:134366151:134375584:+	PRRC2B
9:140669561:140669704:+	included	ENSG00000181090	9:140648682:140671233:+	EHMT1
X:6995261:6995490:-	included	ENSG00000130021	X:6966960:7066231:-	HDHD1
X:9621585:9621729:+	included	ENSG00000101849	X:9502982:9652110:+	TBL1X
X:14044171:14044340:-	included	ENSG00000046647	X:14026397:14048011:-	GEMIN8
X:14044259:14044340:-	included	ENSG00000046647	X:14026397:14048011:-	GEMIN8
X:19941276:19941525:-	included	ENSG00000173681	X:19930977:19988416:-	CXorf23
X:44965787:44965894:+	included	ENSG00000147050	X:44732756:44971847:+	KDM6A
X:47030427:47030657:+	included	ENSG00000182872	X:47004267:47046210:+	RBM10
X:48930092:48930309:-	included	ENSG00000243279	X:48928812:48931730:-	PRAF2
X:51637365:51637445:+	included	ENSG00000179222	X:51636741:51638848:+	MAGED1
X:53707512:53707647:-	included	ENSG00000086758	X:53611170:53713673:-	HUWE1
X:73749048:73749276:+	included	ENSG00000147100	X:73740954:73752443:+	SLC16A2
X:75396716:75396827:+	included	ENSG00000102390	X:75392770:75398039:+	PBDC1
X:102071920:102072010:+	included	ENSG00000223546	X:102024108:102094892:+	LINC00630
X:102631956:102632034:+	included	ENSG00000166681	X:102631267:102633005:+	NGFRAP1
X:108939373:108939425:-	included	ENSG00000068366	X:108926041:108976554:-	ACSL4
X:129289131:129289261:-	included	ENSG00000156709	X:129263336:129299638:-	AIFM1
X:135961176:135961612:-	included	ENSG00000147274	X:135954433:135961590:-	RBMX
X:148608475:148608607:-	included	ENSG00000010404	X:148564073:148615470:-	IDS

Table S1. Exons affected by SWI/SNF ATPase subunits

BRG1-wt

Affected exon coordinates	included/skipped	ENSEMBL gene ID	gene coordinates	Gene name
X:151885137:151885234:+	included	ENSG00000183305	X:151883081:151885563:+	MAGEA2B
X:152091264:152091353:+	included	ENSG00000147394	X:152082985:152142024:+	ZNF185
X:152097119:152097205:+	included	ENSG00000147394	X:152082985:152142024:+	ZNF185
X:154300602:154300618:+	included	ENSG00000214827	X:154293437:154376212:-	MTCP1

Table S1. Exons affected by SWI/SNF ATPase subunits

BRG1-mut

Affected exon coordinates	included/skipped	ENSEMBL gene ID	gene coordinates	Gene name
1:2124086:2124414:-	skipped	ENSG00000162585	1:2115916:2126180:-	C1orf86
1:2124284:2124414:-	skipped	ENSG00000162585	1:2115916:2126180:-	C1orf86
1:3765172:3765345:-	skipped	ENSG00000116198	1:3728644:3773763:-	CEP104
1:10167280:10167432:+	skipped	ENSG00000130939	1:10167388:10192538:+	UBE4B
1:11715385:11715756:+	skipped	ENSG00000132879	1:11714913:11723384:+	FBXO44
1:11903871:11904130:+	skipped	ENSG00000242349	1:11901073:11907741:+	NPPA-AS1
1:15536986:15537058:+	skipped	ENSG00000171729	1:15479027:15546973:+	TMEM51
1:31452909:31453025:-	skipped	ENSG00000134644	1:31447495:31454992:-	PUM1
1:35761528:35761589:+	skipped	ENSG00000146463	1:35734567:35887545:+	ZMYM4
1:38157131:38157253:-	skipped	ENSG00000116922	1:38147241:38157888:-	C1orf109
1:40313658:40313769:-	skipped	ENSG00000043514	1:40307504:40313769:-	TRIT1
1:41618271:41618413:-	skipped	ENSG00000010803	1:41492871:41627104:-	SCMH1
1:43161870:43161959:+	skipped	ENSG00000065978	1:43148097:43168020:+	YBX1
1:46812593:46812747:+	skipped	ENSG00000117481	1:46805848:46827863:+	NSUN4
1:52924008:52924121:-	skipped	ENSG00000134744	1:52898760:52928584:-	ZCCHC11
1:53370706:53372283:-	skipped	ENSG00000121310	1:53364845:53378502:-	ECHDC2
1:61851543:61851596:+	skipped	ENSG00000162599	1:61330930:61921197:+	NFIA
1:78211106:78211284:-	skipped	ENSG00000077254	1:78161671:78225537:-	USP33
1:93792159:93792219:-	skipped	ENSG00000223745	1:93727742:93811388:-	RP4-717I23.3
1:104120112:104120230:+	skipped	ENSG00000240038	1:104096436:104122152:+	AMY2B
1:110774843:110775219:+	skipped	ENSG00000116396	1:110762337:110776666:+	KCNC4
1:111493910:111495437:-	skipped	ENSG00000232811	1:111486088:111495470:+	RP11-96K19.2
1:143721045:143721165:-	skipped	ENSG00000226637	1:143719662:143721171:-	RP6-206I17.3
1:145290781:145290850:+	skipped	ENSG00000213240	1:145290421:145291972:+	NOTCH2NL
1:146055345:146055447:-	skipped	ENSG00000152042	1:146032646:146082765:-	NBPF11
1:147597182:147597284:-	skipped	ENSG00000203836	1:147596183:147615861:-	NBPF24
1:147908554:147908674:-	skipped	ENSG00000225871	1:147907214:147909036:+	RP11-495P10.10
1:150990943:150991145:+	skipped	ENSG00000143363	1:150980895:151001429:+	PRUNE
1:151214914:151215043:+	skipped	ENSG00000143398	1:151214587:151220959:+	PIP5K1A
1:151257682:151258550:+	skipped	ENSG00000143373	1:151254093:151259181:+	ZNF687
1:154186369:154186422:-	skipped	ENSG00000143612	1:154184932:154186992:-	C1orf43
1:154192312:154192413:-	skipped	ENSG00000143612	1:154179181:154193082:-	C1orf43
1:155110037:155110198:+	skipped	ENSG00000169241	1:155107819:155110844:+	SLC50A1
1:160231075:160231148:-	skipped	ENSG00000132716	1:160185504:160232291:-	DCAF8
1:161130119:161130296:+	skipped	ENSG00000143258	1:161129239:161132158:+	USP21
1:183666538:183666647:+	skipped	ENSG00000143344	1:183605219:183897665:+	RGL1
1:210126400:210126604:+	skipped	ENSG00000143469	1:210111537:210337631:+	SYT14
1:213186435:213186760:-	skipped	ENSG00000174606	1:213179880:213186760:-	ANGEL2
1:222836230:222836373:+	skipped	ENSG00000154305	1:222833280:222837392:+	MIA3
1:225692693:225692755:-	skipped	ENSG00000154380	1:225677501:225700398:-	ENAH
1:225704898:225705692:-	skipped	ENSG00000154380	1:225674536:225840844:-	ENAH
1:227068170:227068425:+	skipped	ENSG00000143801	1:227057884:227069671:+	PSEN2
1:228328825:228329208:+	skipped	ENSG00000143774	1:228327662:228336541:+	GUK1
1:228329334:228329530:+	skipped	ENSG00000143774	1:228327662:228336541:+	GUK1
1:233362972:233363117:-	skipped	ENSG00000135749	1:233334712:233363015:-	PCNXL2
1:241767562:241767881:-	skipped	ENSG00000054277	1:241756425:241803663:-	OPN3
1:244613292:244613473:-	skipped	ENSG00000035687	1:244571795:244615436:-	ADSS
1:247486000:247486107:-	skipped	ENSG00000162714	1:247460713:247495045:-	ZNF496
10:27442156:27442210:-	skipped	ENSG00000136758	10:27399382:27443288:-	YME1L1
10:33242521:33242737:-	skipped	ENSG00000150093	10:33189246:33246822:-	ITGB1
10:46280957:46281079:+	skipped	ENSG00000172661	10:46274463:46281411:+	FAM21C
10:51613218:51613311:-	skipped	ENSG00000138297	10:51592079:51623338:-	TIMM23

Table S1. Exons affected by SWI/SNF ATPase subunits

BRG1-mut

Affected exon coordinates	included/skipped	ENSEMBL gene ID	gene coordinates	Gene name
10:64979638:64979743:-	skipped	ENSG00000171988	10:64926980:65225641:-	JMJD1C
10:75239608:75239726:-	skipped	ENSG00000107758	10:75196185:75255759:-	PPP3CB
10:75898564:75898689:-	skipped	ENSG00000185009	10:75898013:75910515:-	AP3M1
10:103344359:103344676:-	skipped	ENSG00000166169	10:103338638:103347966:-	POLL
10:103344635:103344676:-	skipped	ENSG00000166169	10:103338638:103347966:-	POLL
11:504824:504996:-	skipped	ENSG00000023191	11:494512:506821:-	RNH1
11:2423069:2423377:+	skipped	ENSG00000184281	11:2421985:2424473:+	TSSC4
11:2423206:2423377:+	skipped	ENSG00000184281	11:2421985:2424473:+	TSSC4
11:2970457:2970494:-	skipped	ENSG00000205531	11:2965667:2973999:-	NAP1L4
11:3394807:3394905:-	skipped	ENSG00000005801	11:3392209:3400384:-	ZNF195
11:10828802:10828866:-	skipped	ENSG00000110321	11:10818596:10829520:-	EIF4G2
11:18031618:18031686:-	skipped	ENSG00000129158	11:18029487:18031889:-	SERGEF
11:18418027:18418157:+	skipped	ENSG00000134333	11:18415934:18429972:+	LDHA
11:33369712:33369774:+	skipped	ENSG00000110422	11:33278217:33375114:+	HIPK3
11:33752970:33753014:-	skipped	ENSG00000085063	11:33730245:33757985:-	CD59
11:36619194:36619280:+	skipped	ENSG00000175097	11:36597123:36619782:-	RAG2
11:43918745:43918904:+	skipped	ENSG00000166199	11:43911311:43941816:+	ALKBH3
11:46639810:46639925:+	skipped	ENSG00000175224	11:46639084:46646914:+	ATG13
11:47707445:47707601:-	skipped	ENSG00000165923	11:47681142:47711922:-	AGBL2
11:61101021:61101122:+	skipped	ENSG00000167986	11:61097428:61101247:-	DDB1
11:63523584:63523642:+	skipped	ENSG00000133318	11:63448917:63527354:+	RTN3
11:65112051:65112092:+	skipped	ENSG00000133884	11:65111730:65120450:+	DPF2
11:67374770:67375087:+	skipped	ENSG00000167792	11:67374322:67379977:+	NDUFV1
11:67799000:67799177:+	skipped	ENSG00000110717	11:67798083:67801197:+	NDUFS8
11:68350511:68350597:+	skipped	ENSG00000110075	11:68326109:68380822:+	PPP6R3
11:71191265:71191320:+	skipped	ENSG00000172890	11:71189440:71196694:+	NADSYN1
11:71502782:71502865:+	skipped	ENSG00000158483	11:71498555:71512280:+	FAM86C1
11:71822488:71822542:-	skipped	ENSG00000110200	11:71817423:71823750:-	ANAPC15
11:75274772:75274815:+	skipped	ENSG00000149257	11:75273100:75283828:+	SERPINH1
11:86168408:86168801:-	skipped	ENSG00000151376	11:86168620:86209166:-	ME3
11:102080248:102080295:+	skipped	ENSG00000137693	11:101981191:102104154:+	YAP1
11:111956691:111957034:-	skipped	ENSG00000150779	11:111955523:111957522:-	TIMM8B
11:112050001:112050292:+	skipped	ENSG00000255292	11:111957626:112064528:+	SDHD
11:116820849:116820972:-	skipped	ENSG00000160584	11:116714117:116969137:-	SIK3
11:118897216:118897398:-	skipped	ENSG00000137700	11:118894823:118901616:-	SLC37A4
11:126161320:126161464:+	skipped	ENSG00000150455	11:126160750:126168740:+	TIRAP
12:86496:86722:+	skipped	ENSG00000226210	12:73724:91218:+	ABC7-42389800N19.1
12:90104:90314:+	skipped	ENSG00000226210	12:73724:91218:+	ABC7-42389800N19.1
12:514677:514730:+	skipped	ENSG00000120647	12:510816:518947:+	CCDC77
12:10761697:10761982:-	skipped	ENSG00000111196	12:10758611:10766222:-	MAGOHB
12:26147970:26148063:+	skipped	ENSG00000123094	12:26111990:26173401:+	RASSF8
12:30839255:30839593:-	skipped	ENSG00000133704	12:30837234:30841680:-	IPO8
12:46367973:46369517:-	skipped	ENSG00000139218	12:46315191:46384348:-	SCAF11
12:48543870:48543923:-	skipped	ENSG00000177981	12:48543239:48545169:-	ASB8
12:49210656:49210778:+	skipped	ENSG00000167535	12:49209394:49211095:+	CACNB3
12:50586235:50586347:-	skipped	ENSG00000050405	12:50571556:50589830:-	LIMA1
12:51449618:51449804:+	skipped	ENSG00000050426	12:51449481:51450312:+	LETMD1
12:53343240:53343369:-	skipped	ENSG00000170421	12:53342267:53343615:-	KRT8
12:53679708:53680696:+	skipped	ENSG00000135476	12:53676033:53683079:+	ESPL1
12:53690214:53691708:+	skipped	ENSG00000123349	12:53691396:53693230:+	PFDN5
12:54675579:54675725:+	skipped	ENSG00000135486	12:54673976:54680871:+	HNRNPA1
12:56510973:56510995:+	skipped	ENSG00000229117	12:56510369:56511727:+	RPL41

Table S1. Exons affected by SWI/SNF ATPase subunits

BRG1-mut

Affected exon coordinates	included/skipped	ENSEMBL gene ID	gene coordinates	Gene name
12:56685415:56685577:-	skipped	ENSG00000062485	12:56679974:56685542:-	CS
12:56692393:56692475:-	skipped	ENSG00000062485	12:56665482:56694102:-	CS
12:58145283:58145535:-	skipped	ENSG00000135446	12:58141509:58146304:-	CDK4
12:69018451:69018653:+	skipped	ENSG00000127314	12:69004618:69054372:+	RAP1B
12:69651815:69651925:+	skipped	ENSG00000111605	12:69633316:69668138:+	CPSF6
12:109046814:109046836:-	skipped	ENSG00000110880	12:109038884:109125372:-	CORO1C
12:110937340:110937351:-	skipped	ENSG00000111237	12:110936600:110939912:-	VPS29
12:120648733:120648909:+	skipped	ENSG00000255857	12:120639093:120650722:+	PXN-AS1
12:123460800:123460948:+	skipped	ENSG00000111325	12:123459126:123464589:+	OGFOD2
12:123712010:123712119:-	skipped	ENSG00000051825	12:123638761:123717670:-	MPHOSPH9
12:124440831:124440991:-	skipped	ENSG00000119242	12:124420955:124457371:-	CCDC92
12:132249001:132249231:+	skipped	ENSG00000061936	12:132195633:132284282:+	SFSWAP
13:33109906:33111164:-	skipped	ENSG00000244754	13:33101011:33110520:-	N4BP2L2
13:73309098:73309143:+	skipped	ENSG00000136122	13:73302060:73319317:+	BORA
13:78317151:78317300:+	skipped	ENSG00000139737	13:78315672:78320993:+	SLAIN1
13:98659616:98659841:+	skipped	ENSG00000065150	13:98655262:98659841:+	IPO5
13:111302984:111303447:-	skipped	ENSG00000134905	13:111299207:111303554:-	CARS2
14:21679565:21679722:-	skipped	ENSG00000092199	14:21679095:21680161:-	HNRNPC
14:23469166:23469326:-	skipped	ENSG00000100802	14:23465448:23471978:-	C14orf93
14:23536523:23537880:-	skipped	ENSG00000100813	14:23527772:23540774:-	ACIN1
14:24035771:24035895:-	skipped	ENSG00000213983	14:24035547:24036956:-	AP1G2
14:24459984:24460161:+	skipped	ENSG00000187630	14:24439147:24475610:+	DHRS4L2
14:24470593:24470726:+	skipped	ENSG00000187630	14:24464305:24475157:+	DHRS4L2
14:39627489:39628754:-	skipped	ENSG00000182400	14:39617014:39639736:-	TRAPPC6B
14:50074118:50074839:+	skipped	ENSG00000165501	14:50065414:50081390:+	LRR1
14:53185695:53185756:+	skipped	ENSG00000100519	14:53184948:53194712:+	PSMC6
14:53518562:53518645:-	skipped	ENSG00000100523	14:53515265:53519804:-	DDHD1
14:58686412:58686447:+	skipped	ENSG00000131966	14:58666797:58701478:+	ACTR10
14:69345175:69345240:-	skipped	ENSG00000072110	14:69341039:69345739:-	ACTN1
14:73444845:73444942:-	skipped	ENSG00000165861	14:73441535:73445703:-	ZFYVE1
14:74766251:74766378:-	skipped	ENSG00000119688	14:74762432:74769637:-	ABCD4
14:75528387:75528465:-	skipped	ENSG00000119640	14:75519923:75530752:-	ACYP1
14:90390857:90391021:-	skipped	ENSG00000140025	14:90314776:90398938:-	EFCAB11
14:91751739:91752180:-	skipped	ENSG00000015133	14:91737666:91755675:-	CCDC88C
14:92622823:92622975:+	skipped	ENSG00000165934	14:92621522:92627590:+	CPSF2
14:100341268:100341324:+	skipped	ENSG00000066629	14:100317186:100349883:+	EML1
15:23027801:23027922:-	skipped	ENSG00000140157	15:23019818:23034378:-	NIPA2
15:23196162:23196291:-	skipped	ENSG00000187667	15:23187727:23208417:-	WHAMMP3
15:25300221:25300377:+	skipped	ENSG00000224078	15:25295778:25367623:+	SNHG14
15:42121302:42121490:+	skipped	ENSG00000243708	15:42120282:42140345:+	PLA2G4B
15:42852980:42853068:+	skipped	ENSG00000137814	15:42851581:42853885:+	HAUS2
15:43856301:43856363:-	skipped	ENSG00000168781	15:43825659:43877062:-	PPIP5K1
15:52856387:52856443:-	skipped	ENSG00000128989	15:52839241:52861436:-	ARPP19
15:55724695:55724800:-	skipped	ENSG00000256061	15:55709952:55790546:-	DYX1C1
15:64668942:64669104:-	skipped	ENSG00000166803	15:64657192:64673709:-	KIAA0101
15:65281507:65281611:-	skipped	ENSG00000090487	15:65275270:65282249:-	SPG21
15:68497459:68497489:-	skipped	ENSG00000129007	15:68486140:68498387:-	CALML4
15:69745986:69747626:+	skipped	ENSG00000137818	15:69745122:69748255:+	RPLP1
15:75640762:75640987:+	skipped	ENSG00000140398	15:75639295:75641312:+	NEIL1
15:76587932:76588078:-	skipped	ENSG00000140374	15:76566823:76603754:-	ETFA
15:79606883:79606997:+	skipped	ENSG00000166557	15:79603403:79615189:+	TMED3
15:80390758:80390920:+	skipped	ENSG00000086666	15:80351909:80430735:+	ZFAND6

Table S1. Exons affected by SWI/SNF ATPase subunits

BRG1-mut

Affected exon coordinates	included/skipped	ENSEMBL gene ID	gene coordinates	Gene name
15:82769402:82769439:-	skipped	ENSG00000255769	15:82768638:82770067:-	RP11-152F13.3
15:82969811:82969899:+	skipped	ENSG00000259328	15:82969810:82971216:+	RP11-152F13.7
15:82970439:82970534:+	skipped	ENSG00000259328	15:82969810:82971216:+	RP11-152F13.7
15:83145986:83146023:-	skipped	ENSG00000259472	15:83145206:83146651:-	RP13-996F3.3
15:89422649:89423841:-	skipped	ENSG00000140511	15:89420518:89438857:-	HAPLN3
15:93426815:93427008:+	skipped	ENSG00000272888	15:93425961:93427091:+	AC013394.2
16:167300:167374:-	skipped	ENSG00000103148	16:135384:188348:-	NPRL3
16:448208:448394:+	skipped	ENSG00000103202	16:446724:450746:+	NME4
16:1888017:1888135:+	skipped	ENSG00000162039	16:1887684:1891908:-	MEIOB
16:2012874:2012910:-	skipped	ENSG00000140988	16:2012052:2014861:-	RPS2
16:2013654:2014366:-	skipped	ENSG00000140988	16:2012052:2014861:-	RPS2
16:2314574:2314761:-	skipped	ENSG00000205937	16:2312208:2318115:-	RNPS1
16:3707000:3707112:+	skipped	ENSG00000213918	16:3704667:3713727:+	DNASE1
16:4465716:4465853:-	skipped	ENSG00000262246	16:4457525:4465898:-	CORO7
16:8953962:8954166:-	skipped	ENSG00000153048	16:8948921:8955570:-	CARHSP1
16:15458720:15458789:-	skipped	ENSG00000183793	16:15458701:15463926:-	NPIPA5
16:15793586:15793711:+	skipped	ENSG00000072864	16:15781283:15793787:+	NDE1
16:16417609:16417705:+	skipped	ENSG00000244257	16:16404197:16428047:+	PKD1P1
16:16429718:16429994:+	skipped	ENSG00000183889	16:16416161:16444443:+	NPIPA7
16:16429909:16429994:+	skipped	ENSG00000183889	16:16416161:16444443:+	NPIPA7
16:18453239:18453308:-	skipped	ENSG00000233024	16:18451942:18468926:-	NPIPA7
16:18466435:18466711:-	skipped	ENSG00000233024	16:18451942:18468926:-	NPIPA7
16:23403283:23403555:-	skipped	ENSG00000168434	16:23399961:23404902:-	COG7
16:23568211:23568248:-	skipped	ENSG00000103356	16:23563512:23568676:-	EARS2
16:29983363:29983461:+	skipped	ENSG00000149932	16:29952205:29984371:+	TMEM219
16:30005227:30006164:-	skipped	ENSG00000149929	16:30003644:30007757:-	HIRIP3
16:30012533:30015978:+	skipped	ENSG00000169592	16:30007564:30012896:+	INO80E
16:30078206:30078359:+	skipped	ENSG00000149925	16:30075492:30080278:+	ALDOA
16:30094067:30094168:+	skipped	ENSG00000149923	16:30087298:30096100:+	PPP4C
16:30770975:30771130:-	skipped	ENSG00000156873	16:30759590:30772490:+	PHKG2
16:57168687:57168738:+	skipped	ENSG00000140848	16:57153110:57171160:+	CPNE2
16:57212414:57212764:-	skipped	ENSG00000172775	16:57186377:57219976:-	FAM192A
16:67984557:67984963:-	skipped	ENSG00000124067	16:67983701:67985437:-	SLC12A4
16:69680420:69680481:+	skipped	ENSG00000102908	16:69599928:69719978:+	NFAT5
16:70072808:70073003:-	skipped	ENSG00000196696	16:70010199:70099851:-	PDXDC2P
16:89615388:89615744:+	skipped	ENSG00000197912	16:89611133:89619487:+	SPG7
16:90185153:90185240:+	skipped	ENSG00000261172	16:90182607:90192430:-	RP11-356C4.5
17:6920269:6920333:+	skipped	ENSG00000161939	17:6915953:6920821:+	RNASEK-C17orf49
17:7481438:7481562:+	skipped	ENSG00000161960	17:7476143:7481719:+	EIF4A1
17:8347245:8347494:+	skipped	ENSG00000166579	17:8339968:8351895:+	NDEL1
17:18762653:18762788:+	skipped	ENSG00000141127	17:18761408:18775941:+	PRPSAP2
17:19827721:19827825:-	skipped	ENSG00000108599	17:19807614:19881150:-	AKAP10
17:21114249:21114540:-	skipped	ENSG00000178307	17:21100592:21117937:-	TMEM11
17:21114380:21114540:-	skipped	ENSG00000178307	17:21100592:21117937:-	TMEM11
17:26727626:26727783:-	skipped	ENSG00000076351	17:26722694:26733219:-	SLC46A1
17:36916684:36916861:+	skipped	ENSG00000108294	17:36916637:36920478:+	PSMB3
17:36916761:36916861:+	skipped	ENSG00000108294	17:36916637:36920478:+	PSMB3
17:37813151:37813338:+	skipped	ENSG00000131748	17:37808741:37813305:+	STARD3
17:41610555:41610716:-	skipped	ENSG00000067596	17:41598763:41621831:+	DHX8
17:44067244:44067441:+	skipped	ENSG00000186868	17:44050148:44101797:+	MAPT
17:57814814:57814904:+	skipped	ENSG00000062716	17:57807075:57814878:+	VMP1
17:61803998:61804055:-	skipped	ENSG00000266173	17:61780191:61819143:-	STRADA

Table S1. Exons affected by SWI/SNF ATPase subunits

BRG1-mut

Affected exon coordinates	included/skipped	ENSEMBL gene ID	gene coordinates	Gene name
17:65358892:65359008:-	skipped	ENSG00000197170	17:65334031:65362743:-	PSMD12
17:73225093:73225226:+	skipped	ENSG00000125450	17:73221197:73229047:+	NUP85
17:78252563:78252709:+	skipped	ENSG00000173821	17:78234664:78369112:+	RNF213
17:78391581:78391745:+	skipped	ENSG00000173818	17:78388977:78394333:+	ENDOV
17:78393386:78393520:+	skipped	ENSG00000173818	17:78388977:78394333:+	ENDOV
17:79206115:79206318:-	skipped	ENSG00000167302	17:79202076:79212891:-	ENTHD2
17:80165607:80165924:-	skipped	ENSG00000176155	17:80115323:80170689:-	CCDC57
17:80398349:80398489:+	skipped	ENSG00000169660	17:80397875:80399704:+	HEXDC
18:46619587:46619681:-	skipped	ENSG00000141627	18:46570173:46623863:-	DYM
18:47014870:47014936:-	skipped	ENSG00000215472	18:47008050:47017956:-	RPL17-C18orf32
18:47018106:47018203:-	skipped	ENSG00000265496	18:47013708:47018248:+	MIR1539
18:76856476:76856649:+	skipped	ENSG00000166377	18:76847314:76856535:+	ATP9B
18:77135394:77135426:+	skipped	ENSG00000166377	18:77090079:77138141:+	ATP9B
18:77746602:77746750:-	skipped	ENSG00000141759	18:77732866:77748593:-	TXNL4A
19:1011095:1011194:-	skipped	ENSG00000182087	19:1009649:1012116:-	TMEM259
19:1975108:1976113:+	skipped	ENSG00000133275	19:1969824:1979402:+	CSNK1G2
19:5901346:5901465:-	skipped	ENSG00000266941	19:5899905:5901472:+	AC104532.3
19:9947499:9947551:+	skipped	ENSG00000127445	19:9945998:9960358:+	PIN1
19:10463110:10463227:-	skipped	ENSG00000105397	19:10461208:10463932:-	TYK2
19:11150134:11150229:+	skipped	ENSG00000127616	19:11141488:11152142:+	SMARCA4
19:19144940:19145047:+	skipped	ENSG00000105676	19:19144383:19168987:+	ARMC6
19:19153520:19153686:+	skipped	ENSG00000105676	19:19144383:19168987:+	ARMC6
19:19300068:19300273:-	skipped	ENSG00000254901	19:19287711:19303221:-	MEF2BNB
19:39379894:39379963:-	skipped	ENSG00000068903	19:39379726:39384917:-	SIRT2
19:50183129:50183182:+	skipped	ENSG00000126457	19:50180523:50183376:+	PRMT1
19:50430951:50431105:-	skipped	ENSG00000104951	19:50392910:50432796:-	IL4I1
19:51010831:51010956:-	skipped	ENSG00000161677	19:51009254:51014610:-	JOSD2
19:52703527:52703651:+	skipped	ENSG00000105568	19:52693291:52730687:+	PPP2R1A
19:58515751:58516068:+	skipped	ENSG00000176593	19:58513428:58522600:+	CTD-2368P22.1
2:675758:676238:-	skipped	ENSG00000151353	2:675557:676642:-	TMEM18
2:3607038:3607319:+	skipped	ENSG00000234171	2:3606081:3609324:+	RNASEH1-AS1
2:9554307:9554385:-	skipped	ENSG00000119185	2:9552397:9563216:-	ITGB1BP1
2:9616116:9616168:+	skipped	ENSG00000134330	2:9613786:9624677:+	IAH1
2:27275201:27275411:+	skipped	ENSG00000084693	2:27265231:27276022:+	AGBL5
2:37229449:37229715:-	skipped	ENSG00000008869	2:37208039:37311485:-	HEATR5B
2:62115159:62115392:-	skipped	ENSG00000115484	2:62115082:62115593:-	CCT4
2:70516482:70516504:-	skipped	ENSG00000143977	2:70508493:70520903:-	SNRPG
2:74166037:74166149:+	skipped	ENSG00000114956	2:74153952:74186088:+	DGUOK
2:85241059:85241176:+	skipped	ENSG00000176407	2:85198215:85255179:+	KCMF1
2:97399256:97399338:-	skipped	ENSG00000114988	2:97371665:97405800:-	LMAN2L
2:98124458:98124528:-	skipped	ENSG00000196912	2:98108977:98127712:-	ANKRD36B
2:99689481:99689536:-	skipped	ENSG00000135951	2:99634662:99743639:-	TSGA10
2:101032685:101032987:-	skipped	ENSG00000115526	2:101024134:101034076:-	CHST10
2:102487956:102488147:+	skipped	ENSG00000071054	2:102314205:102507958:+	MAP4K4
2:114351865:114352093:+	skipped	ENSG00000146556	2:114346126:114356611:+	WASH2P
2:122514816:122515010:+	skipped	ENSG00000211460	2:122513235:122515184:+	TSN
2:159214961:159215082:-	skipped	ENSG00000153237	2:159027592:159313265:-	CCDC148
2:170394523:170394651:-	skipped	ENSG00000138399	2:170386258:170430383:-	FASTKD1
2:174223983:174224219:+	skipped	ENSG00000144354	2:174219547:174233632:+	CDCA7
2:174224071:174224219:+	skipped	ENSG00000144354	2:174219547:174233632:+	CDCA7
2:178998588:178998627:+	skipped	ENSG00000155636	2:178988589:179003738:+	RBM45
2:190650072:190650197:+	skipped	ENSG00000064933	2:190649214:190656667:+	PMS1

Table S1. Exons affected by SWI/SNF ATPase subunits

BRG1-mut

Affected exon coordinates	included/skipped	ENSEMBL gene ID	gene coordinates	Gene name
2:191520703:191520879:+	skipped	ENSG00000138386	2:191513620:191524374:+	NAB1
2:192267358:192267444:+	skipped	ENSG00000128641	2:192110001:192290115:+	MYO1B
2:203807463:203807690:+	skipped	ENSG00000138380	2:203776967:203807547:+	CARF
2:204341799:204341879:-	skipped	ENSG00000173166	2:204259067:204399985:-	RAPH1
2:207025611:207025856:+	skipped	ENSG00000114942	2:207024308:207027652:+	EEF1B2
2:210880928:210880981:+	skipped	ENSG00000197713	2:210867288:210886300:+	RPE
2:219525030:219525205:+	skipped	ENSG00000074582	2:219523486:219525940:+	BCS1L
2:220072370:220072496:+	skipped	ENSG00000158552	2:220071505:220073622:+	ZFAND2B
2:223765392:223765498:+	skipped	ENSG00000123983	2:223725651:223809357:+	ACSL3
2:228211942:228212100:+	skipped	ENSG00000168958	2:228205007:228222097:+	MFF
2:233063237:233063486:+	skipped	ENSG00000144535	2:232825954:233209060:+	DIS3L2
2:241535351:241535569:+	skipped	ENSG00000142330	2:241534562:241536119:+	CAPN10
2:242208244:242208710:-	skipped	ENSG00000115677	2:242206226:242212227:-	HDLBP
2:242259618:242259702:+	skipped	ENSG00000168385	2:242255308:242264659:+	SEPT2
2:242621334:242621748:-	skipped	ENSG00000168393	2:242615156:242626406:-	DTYMK
20:363077:363270:+	skipped	ENSG00000101255	20:361940:368814:+	TRIB3
20:10393178:10394579:-	skipped	ENSG00000125863	20:10381656:10414870:-	MKKS
20:18278629:18278706:+	skipped	ENSG00000125846	20:18269120:18297640:+	ZNF133
20:18286963:18287037:+	skipped	ENSG00000125846	20:18285728:18296032:+	ZNF133
20:25201868:25201969:+	skipped	ENSG00000197586	20:25176328:25207365:+	ENTPD6
20:34243124:34243266:-	skipped	ENSG00000244462	20:34242814:34252878:-	RBM12
20:44047492:44047619:+	skipped	ENSG00000124155	20:44044716:44054884:+	PIGT
20:48700666:48700791:-	skipped	ENSG00000244687	20:48697660:48732491:-	UBE2V1
20:49457074:49457467:+	skipped	ENSG00000230043	20:49457151:49457286:-	TMSB4XP6
20:57612246:57612354:-	skipped	ENSG00000101166	20:57608199:57617964:-	SLMO2
20:62367133:62369000:+	skipped	ENSG00000203896	20:62366814:62369794:+	LIME1
20:62507169:62507228:+	skipped	ENSG00000101150	20:62496595:62521777:+	TPD52L2
20:62579578:62579828:-	skipped	ENSG00000198276	20:62571276:62582479:-	UCKL1
21:34809190:34809299:-	skipped	ENSG00000159128	21:34775201:34809828:+	IFNGR2
21:38318309:38318454:-	skipped	ENSG00000159267	21:38309467:38352928:-	HLCS
21:38605663:38605743:-	skipped	ENSG00000157538	21:38602818:38605743:-	DSCR3
21:40717756:40719218:-	skipped	ENSG00000205581	21:40714240:40721025:-	HMGN1
21:45380513:45380660:+	skipped	ENSG00000160216	21:45366810:45404554:+	AGPAT3
21:45482934:45483049:+	skipped	ENSG00000160218	21:45432199:45526433:+	TRAPPC10
21:46194482:46194656:-	skipped	ENSG00000184787	21:46188954:46195351:-	UBE2G2
21:46221199:46221313:-	skipped	ENSG00000184787	21:46219904:46221723:-	UBE2G2
22:17990845:17990928:+	skipped	ENSG00000099954	22:17840836:18018834:+	CECR2
22:18232871:18232940:-	skipped	ENSG00000015475	22:18220823:18257261:-	BID
22:22330277:22330559:-	skipped	ENSG00000100038	22:22328790:22330341:-	TOP3B
22:22899232:22899506:-	skipped	ENSG00000185686	22:22890122:22901768:-	PRAME
22:37419793:37419968:+	skipped	ENSG00000128309	22:37415675:37425842:+	MPST
22:41985056:41985167:-	skipped	ENSG00000100417	22:41979986:41985800:-	PMM1
22:42300539:42300736:+	skipped	ENSG00000198911	22:42294679:42300977:+	SREBF2
3:9731648:9731827:+	skipped	ENSG00000163719	3:9710426:9744071:+	MTMR14
3:9880669:9880855:-	skipped	ENSG00000156990	3:9879565:9882550:-	RPUSD3
3:10360823:10361000:-	skipped	ENSG00000157020	3:10354763:10362796:-	SEC13
3:15273618:15273665:+	skipped	ENSG00000131375	3:15247749:15294425:+	CAPN7
3:15616484:15616589:-	skipped	ENSG00000131373	3:15602210:15643338:-	HACL1
3:47552638:47552716:-	skipped	ENSG00000163832	3:47551445:47555201:-	ELP6
3:47908736:47908828:-	skipped	ENSG00000047849	3:47892181:47951731:-	MAP4
3:48341921:48342124:-	skipped	ENSG00000172113	3:48334753:48342848:-	NME6
3:48540528:48540626:-	skipped	ENSG00000164054	3:48538611:48541573:-	SHISA5

Table S1. Exons affected by SWI/SNF ATPase subunits

BRG1-mut

Affected exon coordinates	included/skipped	ENSEMBL gene ID	gene coordinates	Gene name
3:48731892:48731958:-	skipped	ENSG00000068745	3:48726970:48733021:-	IP6K2
3:48752748:48752960:-	skipped	ENSG00000068745	3:48725435:48754683:-	IP6K2
3:48752822:48752960:-	skipped	ENSG00000068745	3:48725435:48754683:-	IP6K2
3:49514282:49514338:+	skipped	ENSG00000173402	3:49507734:49524848:+	DAG1
3:50141681:50141741:+	skipped	ENSG00000003756	3:50139769:50145688:+	RBM5
3:50603437:50603525:-	skipped	ENSG00000088543	3:50595461:50605096:-	C3orf18
3:51995765:51996104:-	skipped	ENSG00000090097	3:51991469:52001473:-	PCBP4
3:52865424:52865494:-	skipped	ENSG00000055955	3:52863875:52865495:-	ITIH4
3:56594890:56595040:+	skipped	ENSG00000180376	3:56591190:56598167:+	CCDC66
3:58117654:58117746:+	skipped	ENSG00000136068	3:57994126:58157978:+	FLNB
3:69050759:69050865:-	skipped	ENSG00000163378	3:69024364:69063112:-	EOGT
3:73112824:73112898:+	skipped	ENSG00000163605	3:73045935:73118350:+	PPP4R2
3:119222379:119222467:+	skipped	ENSG00000113845	3:119217378:119243937:+	TIMMDC1
3:119222801:119222868:+	skipped	ENSG00000113845	3:119217378:119243937:+	TIMMDC1
3:123451743:123451949:-	skipped	ENSG00000065534	3:123428004:123454617:-	MYLK
3:129174957:129175107:+	skipped	ENSG00000163913	3:129168713:129182465:+	IFT122
3:130887674:130887781:+	skipped	ENSG00000114670	3:130745693:130903595:+	NEK11
3:131220688:131220768:-	skipped	ENSG00000114686	3:131206524:131221795:-	MRPL3
3:138515305:138515409:-	skipped	ENSG00000051382	3:138474598:138553675:-	PIK3CB
3:141461486:141461749:+	skipped	ENSG00000114125	3:141457045:141466402:+	RNF7
3:158306641:158306713:+	skipped	ENSG00000178053	3:158288951:158322981:+	MLF1
3:160083881:160083940:-	skipped	ENSG00000068885	3:160073800:160095242:-	IFT80
3:185649365:185649640:-	skipped	ENSG00000136527	3:185649302:185655812:-	TRA2B
3:196294062:196294178:-	skipped	ENSG00000185798	3:196281055:196295510:-	WDR53
4:466364:466490:-	skipped	ENSG00000251595	4:419223:467918:-	ABCA11P
4:524225:524534:+	skipped	ENSG00000174227	4:515678:524287:+	PIGG
4:3430285:3430438:+	skipped	ENSG00000159788	4:3424261:3432174:+	RGS12
4:20711306:20711396:+	skipped	ENSG00000163138	4:20709424:20711459:+	PACRGL
4:44682458:44682569:+	skipped	ENSG00000151806	4:44680510:44691426:+	GUF1
4:71607344:71607397:+	skipped	ENSG00000018189	4:71570140:71634339:+	RUFY3
4:83803011:83803090:-	skipped	ENSG00000138674	4:83764837:83812306:-	SEC31A
4:99806073:99806212:-	skipped	ENSG00000151247	4:99792834:99851788:-	EIF4E
4:103674980:103675152:-	skipped	ENSG00000109323	4:103552659:103682098:-	MANBA
4:115540579:115540681:+	skipped	ENSG00000174607	4:115519610:115599380:+	UGT8
4:123147862:123147990:+	skipped	ENSG00000138688	4:123107335:123161084:+	KIAA1109
4:129083349:129083472:+	skipped	ENSG00000138709	4:128982466:129100565:+	LARP1B
4:147177978:147178088:-	skipped	ENSG00000120519	4:147175126:147443116:-	SLC10A7
4:152022127:152022314:+	skipped	ENSG00000145425	4:152020724:152025804:+	RPS3A
4:157782581:157782641:-	skipped	ENSG00000145431	4:157693940:157795124:-	PDGFC
4:177017614:177017736:+	skipped	ENSG00000150627	4:176987027:177032444:+	WDR17
5:1630524:1630681:-	skipped	ENSG00000188002	5:1629115:1634099:-	RP11-43F13.1
5:32235068:32235235:-	skipped	ENSG00000150712	5:32227099:32313044:-	MTMR12
5:69206202:69206389:+	skipped	ENSG00000198237	5:69171097:69216694:+	RP11-98J23.2
5:69492705:69492797:-	skipped	ENSG00000254701	5:69435401:69493223:-	RP11-1415C14.4
5:69521435:69521622:-	skipped	ENSG00000253816	5:69515744:69554757:-	RP11-1415C14.3
5:87705890:87706011:+	skipped	ENSG00000247828	5:87688011:87713308:+	TMEM161B-AS1
5:94988821:94988918:+	skipped	ENSG00000175449	5:94982457:94991894:+	RFESD
5:102523015:102523077:+	skipped	ENSG00000145725	5:102519943:102526759:+	PPIP5K2
5:132109615:132109904:-	skipped	ENSG00000164402	5:132086508:132113067:-	SEPT8
5:137354644:137354835:-	skipped	ENSG00000031003	5:137273648:137368720:-	FAM13B
5:139244700:139244758:-	skipped	ENSG00000158458	5:139227501:139283982:-	NRG2
5:140020603:140020805:+	skipped	ENSG00000131495	5:140018324:140026899:-	NDUFA2

Table S1. Exons affected by SWI/SNF ATPase subunits

BRG1-mut

Affected exon coordinates	included/skipped	ENSEMBL gene ID	gene coordinates	Gene name
5:140073520:140073639:+	skipped	ENSG00000112855	5:140071010:140078876:+	HARS2
5:140898511:140898563:-	skipped	ENSG00000131504	5:140894582:140904322:-	DIAPH1
5:145889630:145889723:+	skipped	ENSG00000113649	5:145886933:145891068:+	TCERG1
5:149824042:149824204:-	skipped	ENSG00000164587	5:149822752:149829310:-	RPS14
5:150490188:150490205:-	skipped	ENSG00000197043	5:150480665:150537443:-	ANXA6
5:153832016:153832059:+	skipped	ENSG00000164576	5:153831957:153835709:+	SAP30L
5:169014203:169014376:+	skipped	ENSG00000040275	5:169014262:169015847:+	SPDL1
5:171341347:171341409:-	skipped	ENSG00000072803	5:171326936:171433663:-	FBXW11
6:2969013:2969147:-	skipped	ENSG00000124570	6:2948392:2972090:-	SERPINB6
6:15660614:15660752:-	skipped	ENSG00000047579	6:15523031:15663273:-	DTNBP1
6:28130721:28130878:+	skipped	ENSG00000226314	6:28129569:28131289:+	ZNF192P1
6:30168812:30168915:-	skipped	ENSG00000234127	6:30152231:30172542:-	TRIM26
6:31861139:31861439:-	skipped	ENSG00000204371	6:31847535:31865461:-	EHMT2
6:32629124:32629234:-	skipped	ENSG00000179344	6:32627243:32634415:-	HLA-DQB1
6:33288513:33289751:-	skipped	ENSG00000204209	6:33286334:33290787:-	DAXX
6:41884523:41884677:-	skipped	ENSG00000124641	6:41884593:41888843:-	MED20
6:42984172:42984322:+	skipped	ENSG00000124702	6:42981950:42989036:+	KLHDC3
6:83759428:83759491:-	skipped	ENSG00000118420	6:83602116:83775560:-	UBE3D
6:99864225:99864304:-	skipped	ENSG00000132424	6:99845926:99873184:-	PNISR
6:109421395:109421489:+	skipped	ENSG00000183137	6:109416312:109479691:+	CEP57L1
6:109450507:109450695:+	skipped	ENSG00000183137	6:109450506:109485113:+	CEP57L1
6:109775883:109776093:-	skipped	ENSG00000135596	6:109773548:109776925:-	MICAL1
6:111805947:111806064:+	skipped	ENSG00000231889	6:111804713:111814206:+	TRAF3IP2-AS1
6:119281934:119282028:-	skipped	ENSG00000111879	6:119280993:119285925:-	FAM184A
6:130370901:130370975:+	skipped	ENSG00000198945	6:130339727:130372488:+	L3MBTL3
6:152819877:152819927:-	skipped	ENSG00000131018	6:152442818:152958534:-	SYNE1
6:153312320:153312456:-	skipped	ENSG00000112031	6:153308496:153315777:-	MTRF1L
6:160208775:160208903:-	skipped	ENSG00000120438	6:160205021:160210704:-	TCP1
7:872142:872238:+	skipped	ENSG00000164828	7:870566:881672:+	SUN1
7:889157:889240:+	skipped	ENSG00000164828	7:855527:914557:+	SUN1
7:2584543:2584690:-	skipped	ENSG00000106009	7:2577510:2595361:-	BRAT1
7:12393441:12394113:-	skipped	ENSG00000146530	7:12370510:12443531:-	VWDE
7:44465499:44465878:-	skipped	ENSG00000015676	7:44444111:44530205:-	NUDCD3
7:66233818:66234012:+	skipped	ENSG00000243335	7:66205642:66276446:+	KCTD7
7:72514920:72515008:+	skipped	ENSG00000174384	7:72507940:72515008:+	RP11-313P13.4
7:72957878:72957974:-	skipped	ENSG00000106635	7:72951488:72957931:-	BCL7B
7:73604577:73604636:+	skipped	ENSG00000106682	7:73598151:73609135:+	EIF4H
7:74317004:74317092:+	skipped	ENSG00000123965	7:74306893:74322330:+	PMS2P5
7:87436688:87436828:+	skipped	ENSG00000105784	7:87257728:87461611:+	RUNDC3B
7:89984400:89984544:+	skipped	ENSG00000105793	7:89969684:90006889:+	GTPBP10
7:99923314:99923402:-	skipped	ENSG00000078319	7:99918614:99939531:-	PMS2P1
7:100861600:100865077:+	skipped	ENSG00000106397	7:100860703:100861647:-	PLOD3
7:102228681:102228892:-	skipped	ENSG00000105808	7:102222796:102234438:-	RASA4
7:102724477:102724509:+	skipped	ENSG00000170632	7:102715327:102740205:+	ARMC10
7:107392990:107393062:+	skipped	ENSG00000105879	7:107384589:107396037:+	CBLL1
7:127981172:127981273:-	skipped	ENSG00000106344	7:127975598:127983928:-	RBM28
7:129269919:129270009:+	skipped	ENSG00000106459	7:129251579:129273621:+	NRF1
7:134620439:134620516:+	skipped	ENSG00000122786	7:134429002:134653153:+	CALD1
7:150067803:150067973:+	skipped	ENSG00000214022	7:150065277:150068769:+	REPIN1
7:150817607:150817832:+	skipped	ENSG00000133612	7:150814840:150819965:+	AGAP3
7:150934042:150934258:+	skipped	ENSG00000033100	7:150929574:150935905:+	CHPF2
8:16927197:16927228:+	skipped	ENSG00000155970	8:16921638:16977799:+	MICU3

Table S1. Exons affected by SWI/SNF ATPase subunits

BRG1-mut

Affected exon coordinates	included/skipped	ENSEMBL gene ID	gene coordinates	Gene name
8:17581181:17581342:-	skipped	ENSG00000129422	8:17580749:17658426:-	MTUS1
8:22423339:22423385:+	skipped	ENSG00000120896	8:22418443:22423385:+	SORBS3
8:37963549:37963657:+	skipped	ENSG00000129691	8:37963017:37968329:+	ASH2L
8:59472843:59474354:+	skipped	ENSG00000137575	8:59465482:59484860:+	SDCBP
8:67834849:67834960:-	skipped	ENSG00000245910	8:67833918:67837776:-	SNHG6
8:74881795:74881908:-	skipped	ENSG00000154582	8:74851403:74884421:-	TCEB1
8:87497101:87497190:-	skipped	ENSG00000176623	8:87490988:87500788:-	RMDN1
8:97243454:97243743:-	skipped	ENSG00000156467	8:97242704:97247771:-	UQCRB
8:97243607:97243743:-	skipped	ENSG00000156467	8:97242704:97247771:-	UQCRB
8:117798742:117798888:+	skipped	ENSG00000147679	8:117778741:117798816:+	UTP23
8:130916745:130916831:-	skipped	ENSG00000153310	8:130851838:130952078:-	FAM49B
8:131104219:131104389:-	skipped	ENSG00000153317	8:131088642:131124660:-	ASAP1
8:141930913:141931231:-	skipped	ENSG00000169398	8:141856757:141944659:-	PTK2
8:144676063:144676241:-	skipped	ENSG00000104529	8:144671350:144679612:-	EEF1D
8:144902836:144902886:-	skipped	ENSG00000179950	8:144900119:144911214:-	PUF60
9:2651415:2651498:+	skipped	ENSG00000147852	9:2621833:2660053:+	VLDLR
9:3301546:3301620:-	skipped	ENSG00000080298	9:3218296:3525983:-	RFX3
9:16832242:16832348:-	skipped	ENSG00000173068	9:16409500:16870704:-	BNC2
9:21993881:21994067:-	skipped	ENSG00000147889	9:21989338:21994872:-	CDKN2A
9:33928370:33928610:-	skipped	ENSG00000137073	9:33921856:33928833:-	UBAP2
9:46698075:46698184:+	skipped	ENSG00000227449	9:46687461:46746500:+	RP11-111F5.5
9:70475019:70475078:-	skipped	ENSG00000147996	9:70432003:70490246:-	CBWD5
9:70858099:70858196:+	skipped	ENSG00000196873	9:70856860:70862843:+	CBWD3
9:90584711:90584834:-	skipped	ENSG00000156345	9:90581355:90589600:-	CDK20
9:99277931:99278074:-	skipped	ENSG00000081377	9:99276784:99284885:-	CDC14B
9:100872351:100872516:-	skipped	ENSG00000106785	9:100831556:100881492:-	TRIM14
9:103191788:103191944:+	skipped	ENSG00000066697	9:103189437:103213511:+	MSANTD3
9:114351544:114351662:-	skipped	ENSG00000106853	9:114312001:114362135:-	PTGR1
9:130190831:130190939:+	skipped	ENSG00000196152	9:130186660:130207651:+	ZNF79
9:130924283:130925231:+	skipped	ENSG00000171159	9:130922538:130926207:+	C9orf16
9:134379576:134379727:+	skipped	ENSG00000130714	9:134378288:134399193:+	POMT1
X:3740415:3740621:-	skipped	ENSG00000205664	X:3735568:3746991:-	RP11-706O15.1
X:47057566:47057754:+	skipped	ENSG00000130985	X:47056597:47060361:+	UBA1
X:47081660:47081779:+	skipped	ENSG00000102225	X:47077258:47081779:+	CDK16
X:52986286:52986436:-	skipped	ENSG00000182646	X:52976461:52986462:-	FAM156A
X:72899489:72899548:+	skipped	ENSG00000204116	X:72783035:72906937:+	CHIC1
X:96018099:96018119:+	skipped	ENSG00000147202	X:95939661:96855597:+	DIAPH2
X:149919502:149919712:+	skipped	ENSG00000063601	X:149861434:149931380:+	MTMR1
X:153073352:153073594:-	skipped	ENSG00000067840	X:153070333:153073594:-	PDZD4
X:153279493:153279729:-	skipped	ENSG00000184216	X:153275950:153285431:-	IRAK1
1:1255836:1256473:-	included	ENSG00000127054	1:1246976:1260071:-	CPSF3L
1:1326677:1326955:-	included	ENSG00000221978	1:1322501:1327029:-	CCNL2
1:1622415:1622832:-	included	ENSG00000189339	1:1592938:1624083:-	SLC35E2B
1:1643703:1643866:-	included	ENSG00000008128	1:1634168:1654270:-	CDK11A
1:1688178:1688321:-	included	ENSG00000008130	1:1683909:1690081:-	NADK
1:10197125:10197263:+	included	ENSG00000130939	1:10182099:10205097:+	UBE4B
1:16719723:16719977:+	included	ENSG00000055070	1:16679069:16719850:+	SZRD1
1:16910089:16910191:-	included	ENSG00000219481	1:16888813:16939982:-	NBPF1
1:19669246:19669358:-	included	ENSG00000077549	1:19665272:19746245:-	CAPZB
1:21276496:21276604:-	included	ENSG00000075151	1:21132962:21377487:-	EIF4G3
1:23660011:23660124:-	included	ENSG00000125944	1:23631180:23670829:-	HNRNPR
1:25233750:25233908:-	included	ENSG00000020633	1:25226001:25291501:-	RUNX3

Table S1. Exons affected by SWI/SNF ATPase subunits

BRG1-mut

Affected exon coordinates	included/skipped	ENSEMBL gene ID	gene coordinates	Gene name
1:26152793:26152902:+	included	ENSG00000117640	1:26149486:26156288:+	MTFR1L
1:26623410:26623486:-	included	ENSG00000158062	1:26607818:26633127:-	UBXN11
1:27440317:27440777:-	included	ENSG00000090020	1:27425305:27481401:-	SLC9A1
1:27736186:27736700:-	included	ENSG00000158195	1:27730729:27816669:-	WASF2
1:28208482:28209554:+	included	ENSG00000130775	1:28199054:28213196:+	THEMIS2
1:32100823:32101123:-	included	ENSG00000162517	1:32096389:32110190:-	PEF1
1:36691013:36691064:+	included	ENSG00000054118	1:36690016:36770958:+	THRAP3
1:38262416:38262492:+	included	ENSG00000185090	1:38259473:38266805:+	MANEAL
1:44063419:44063724:+	included	ENSG00000142949	1:44056691:44087954:+	PTPRF
1:44436578:44436861:+	included	ENSG00000132768	1:44435671:44437198:+	DPH2
1:44436638:44436892:+	included	ENSG00000132768	1:44435671:44437198:+	DPH2
1:46151248:46151292:-	included	ENSG00000159592	1:46092975:46152208:-	GPBP1L1
1:46156646:46156782:+	included	ENSG00000159596	1:46153867:46159517:+	TMEM69
1:47027150:47027314:-	included	ENSG00000079277	1:47023904:47030735:-	MKNK1
1:48917224:48917272:-	included	ENSG00000132122	1:48761043:48937845:-	SPATA6
1:53715052:53715228:-	included	ENSG00000157193	1:53711420:53722995:-	LRP8
1:67293495:67293593:-	included	ENSG00000231080	1:67292624:67294241:+	RP11-342H21.2
1:68151708:68151809:+	included	ENSG00000116717	1:68150743:68154021:+	GADD45A
1:68152033:68152270:+	included	ENSG00000116717	1:68150743:68154021:+	GADD45A
1:78390873:78390914:+	included	ENSG00000162614	1:78383812:78409580:+	NEXN
1:86851141:86851273:-	included	ENSG00000122417	1:86847624:86851227:-	ODF2L
1:87333736:87333785:-	included	ENSG00000183291	1:87328131:87380048:-	SEP15
1:110020440:110020631:+	included	ENSG00000143028	1:110009179:110022410:+	SYPL2
1:113247722:113248874:-	included	ENSG00000155366	1:113247261:113249749:-	RHOC
1:144301071:144301536:-	included	ENSG00000235398	1:144299757:144301536:-	LINC00623
1:148344640:148344742:-	included	ENSG00000203832	1:148341795:148346929:-	NBPF20
1:149615765:149616230:+	included	ENSG00000269501	1:149605700:149616657:+	RP11-353N4.6
1:153614719:153614905:+	included	ENSG00000160679	1:153606524:153618782:+	CHTOP
1:153615703:153615840:+	included	ENSG00000160679	1:153606524:153618782:+	CHTOP
1:153923736:153924142:-	included	ENSG00000160741	1:153920150:153927465:-	CRTC2
1:153934696:153934826:-	included	ENSG00000143570	1:153931574:153936048:-	SLC39A1
1:154956318:154956542:+	included	ENSG00000160688	1:154955813:154960608:+	FLAD1
1:154962815:154963004:+	included	ENSG00000160688	1:154960505:154965587:+	FLAD1
1:155033239:155033308:+	included	ENSG00000143537	1:155030714:155035223:+	ADAM15
1:155109304:155109427:+	included	ENSG00000169241	1:155107819:155110844:+	SLC50A1
1:155279544:155279756:+	included	ENSG00000160752	1:155278704:155282770:+	FDPS
1:155292034:155292228:-	included	ENSG00000225855	1:155287736:155293959:-	RUSC1-AS1
1:155746186:155746272:-	included	ENSG00000116580	1:155736326:155746252:-	GON4L
1:156761489:156761584:+	included	ENSG00000143294	1:156720401:156761584:+	PRCC
1:171558508:171558744:+	included	ENSG00000117523	1:171454650:171560986:+	PRRC2C
1:193038164:193038311:+	included	ENSG00000116747	1:193028551:193054011:+	TROVE2
1:205117333:205117467:-	included	ENSG00000133059	1:205111631:205180727:-	DSTYK
1:205633613:205634014:-	included	ENSG00000158715	1:205626978:205649587:-	SLC45A3
1:213061233:213061343:+	included	ENSG00000162769	1:213031596:213072705:+	FLVCR1
1:213299109:213299186:+	included	ENSG00000136643	1:213224588:213446127:+	RPS6KC1
1:227935393:227935934:+	included	ENSG00000143740	1:227935763:227947353:+	SNAP47
1:228681584:228681970:+	included	ENSG00000168159	1:228674761:228683467:+	RNF187
1:249145062:249145146:-	included	ENSG00000171163	1:249144222:249150142:-	ZNF692
10:6268155:6268328:+	included	ENSG00000170525	10:6263366:6275070:+	PFKFB3
10:30315032:30318795:-	included	ENSG00000165757	10:30301728:30348453:-	KIAA1462
10:35426710:35426807:+	included	ENSG00000095794	10:35415718:35501053:+	CREM
10:46923604:46923810:+	included	ENSG00000165874	10:46914471:46923688:+	FAM35BP

Table S1. Exons affected by SWI/SNF ATPase subunits

BRG1-mut

**Affected exon coordinates**	**included/skipped**	**ENSEMBL gene ID**	**gene coordinates**	**Gene name**
10:47405671:47405877:+	included	ENSG00000189014	10:47379726:47420456:+	FAM35DP
10:51729802:51730218:+	included	ENSG00000214982	10:51623416:51732824:+	PARGP1
10:69408498:69408574:-	included	ENSG00000183230	10:69281599:69425410:-	CTNNA3
10:71914844:71914880:-	included	ENSG00000079332	10:71909959:71922787:-	SAR1A
10:72083619:72083786:-	included	ENSG00000172731	10:72058728:72141670:-	LRRC20
10:75279555:75279750:-	included	ENSG00000221817	10:75256044:75279828:+	RP11-137L10.6
10:75280666:75280785:-	included	ENSG00000166348	10:75257295:75301460:-	USP54
10:75871667:75871870:+	included	ENSG00000035403	10:75843233:75879918:+	VCL
10:81070681:81070941:+	included	ENSG00000108175	10:81065974:81072506:+	ZMIZ1
10:102743062:102744114:+	included	ENSG00000055950	10:102729214:102746953:-	MRPL43
10:103190102:103190209:+	included	ENSG00000166167	10:103113819:103317078:+	BTRC
10:103345619:103345913:-	included	ENSG00000166169	10:103338638:103347966:-	POLL
10:103559999:103560157:-	included	ENSG00000198408	10:103556802:103578175:-	MGEA5
10:104230422:104230874:+	included	ENSG00000138111	10:104221169:104232007:+	TMEM180
10:104860509:104860700:-	included	ENSG00000076685	10:104857728:104866407:-	NT5C2
10:105153956:105154151:-	included	ENSG00000221767	10:105154009:105154158:-	MIR1307
10:120101239:120101439:-	included	ENSG00000165669	10:120094769:120101258:-	FAM204A
10:123658356:123658484:-	included	ENSG00000107669	10:123499938:123687551:-	ATE1
10:134155717:134155775:+	included	ENSG00000148814	10:134145613:134195010:+	LRRC27
11:230452:230641:-	included	ENSG00000142082	11:230602:236345:-	SIRT3
11:8724119:8725158:-	included	ENSG00000166444	11:8718011:8724204:-	ST5
11:14541451:14541757:-	included	ENSG00000129084	11:14535005:14541926:-	PSMA1
11:27696844:27697687:+	included	ENSG00000176697	11:27676439:27723180:-	BDNF
11:36248635:36248980:+	included	ENSG00000179241	11:36184848:36248979:+	LDLRAD3
11:46430063:46430256:-	included	ENSG00000110497	11:46418933:46439545:-	AMBRA1
11:47296114:47296710:+	included	ENSG00000110514	11:47290711:47296277:+	MADD
11:47434951:47435058:+	included	ENSG00000165915	11:47433852:47435431:+	SLC39A13
11:57558857:57559145:+	included	ENSG00000198561	11:57559030:57569266:+	CTNND1
11:57558966:57559145:+	included	ENSG00000198561	11:57559030:57569266:+	CTNND1
11:64039085:64039275:-	included	ENSG00000002330	11:64037740:64039340:-	BAD
11:64082473:64082742:+	included	ENSG00000173153	11:64081242:64083362:+	ESRRA
11:64533423:64533627:-	included	ENSG00000168066	11:64532761:64535316:-	SF1
11:64572506:64572670:-	included	ENSG00000133895	11:64570981:64577957:-	MEN1
11:64806044:64806301:+	included	ENSG00000273003	11:64781653:64808042:+	RP11-399J13.3
11:65006133:65006951:+	included	ENSG00000197847	11:64981310:65010228:+	SLC22A20
11:65480819:65480974:+	included	ENSG00000172977	11:65479709:65486835:+	KAT5
11:65661485:65661592:-	included	ENSG00000175592	11:65659519:65667890:-	FOSL1
11:66391685:66393149:+	included	ENSG00000239306	11:66384052:66394818:+	RBM14
11:66410921:66411611:+	included	ENSG00000173933	11:66406087:66413940:+	RBM4
11:67256738:67256926:+	included	ENSG00000110711	11:67256806:67258124:+	AIP
11:68673537:68673852:+	included	ENSG00000132740	11:68671358:68678947:+	IGHMBP2
11:71809335:71809461:-	included	ENSG00000149357	11:71808337:71810304:-	LAMTOR1
11:76165793:76165834:+	included	ENSG00000158636	11:76155966:76208275:+	C11orf30
11:82903470:82903583:-	included	ENSG00000247137	11:82903061:82904611:-	RP11-727A23.5
11:107496123:107496258:+	included	ENSG00000110675	11:107461816:107536210:+	ELMOD1
11:111624167:111624301:-	included	ENSG00000137713	11:111597631:111637106:-	PPP2R1B
11:113688380:113688559:-	included	ENSG00000048028	11:113668595:113746292:-	USP28
11:118971728:118971757:-	included	ENSG00000172269	11:118967212:118979041:-	DPAGT1
11:125526101:125526230:+	included	ENSG00000149554	11:125495868:125546150:+	CHEK1
11:129993507:129993674:+	included	ENSG00000084234	11:129939731:130013417:+	APLP2
12:2994328:2994700:+	included	ENSG00000171792	12:2985423:2997334:+	RHNO1
12:2994449:2994658:+	included	ENSG00000171792	12:2985423:2997334:+	RHNO1

Table S1. Exons affected by SWI/SNF ATPase subunits

BRG1-mut

Affected exon coordinates	included/skipped	ENSEMBL gene ID	gene coordinates	Gene name
12:4716493:4716553:+	included	ENSG00000010219	12:4708790:4716555:+	DYRK4
12:6781516:6781698:-	included	ENSG00000126746	12:6781206:6782561:-	ZNF384
12:6839835:6839986:+	included	ENSG00000111652	12:6832906:6841022:+	COPS7A
12:6959997:6960173:-	included	ENSG00000111665	12:6953956:6960433:-	CDCA3
12:9072352:9072513:+	included	ENSG00000111752	12:9066491:9075287:+	PHC1
12:9845424:9846544:+	included	ENSG00000069493	12:9833520:9847725:+	CLEC2D
12:10862507:10862713:-	included	ENSG00000060138	12:10851682:10875906:-	YBX3
12:12841447:12841866:-	included	ENSG00000183150	12:12813994:12849121:-	GPR19
12:27829997:27830029:+	included	ENSG00000110841	12:27677105:27844717:+	PPFIBP1
12:31237903:31238060:+	included	ENSG00000013573	12:31231453:31242064:+	DDX11
12:31256256:31256329:+	included	ENSG00000013573	12:31255201:31256939:+	DDX11
12:49399526:49399664:-	included	ENSG00000181929	12:49398006:49412547:-	PRKAG1
12:50040422:50040536:-	included	ENSG00000161791	12:50038735:50046025:-	FMNL3
12:50052225:50052377:-	included	ENSG00000161791	12:50031723:50101197:-	FMNL3
12:51634751:51634900:+	included	ENSG00000183283	12:51632075:51637717:+	DAZAP2
12:53416038:53416150:+	included	ENSG00000063046	12:53399941:53435993:+	EIF4B
12:53621146:53621471:-	included	ENSG00000172819	12:53621157:53625998:-	RARG
12:54581603:54581689:-	included	ENSG00000123415	12:54575236:54582724:-	SMUG1
12:56223273:56223420:-	included	ENSG00000135392	12:56214743:56224565:-	DNAJC14
12:56329089:56329188:+	included	ENSG00000065357	12:56321102:56331227:+	DGKA
12:56373156:56373258:+	included	ENSG00000111540	12:56367696:56388490:+	RAB5B
12:56395996:56396504:+	included	ENSG00000139531	12:56395281:56399309:+	SUOX
12:56396327:56396504:+	included	ENSG00000139531	12:56395281:56399309:+	SUOX
12:56396388:56396504:+	included	ENSG00000139531	12:56395281:56399309:+	SUOX
12:56554410:56554454:+	included	ENSG00000092841	12:56551944:56555358:+	MYL6
12:56558087:56558152:-	included	ENSG00000258199	12:56556142:56584068:-	RP11-977G19.5
12:57487190:57487381:+	included	ENSG00000166886	12:57482676:57489259:+	NAB2
12:57882802:57883128:+	included	ENSG00000166986	12:57881838:57884125:+	MARS
12:58197307:58197452:-	included	ENSG00000135407	12:58197078:58200272:-	AVIL
12:68710361:68710390:-	included	ENSG00000111554	12:68688347:68726066:-	MDM1
12:69045775:69045831:+	included	ENSG00000127314	12:69044023:69050193:+	RAP1B
12:70688017:70688074:+	included	ENSG00000111596	12:70671911:70726626:+	CNOT2
12:70726547:70726626:+	included	ENSG00000111596	12:70721286:70729246:+	CNOT2
12:95650326:95650398:+	included	ENSG00000028203	12:95611521:95650957:+	VEZT
12:100647267:100647493:-	included	ENSG00000166153	12:100597446:100660857:-	DEPDC4
12:108936546:108936627:-	included	ENSG00000075856	12:108923986:108954936:-	SART3
12:111953958:111954167:-	included	ENSG00000204842	12:111947722:111958749:-	ATXN2
12:117287119:117287289:+	included	ENSG00000135119	12:117273801:117290750:+	RNFT2
12:122831922:122832026:-	included	ENSG00000130779	12:122817531:122879974:-	CLIP1
12:123842356:123842399:-	included	ENSG00000139697	12:123779076:123849390:-	SBNO1
12:125621208:125621410:+	included	ENSG00000081760	12:125620335:125621286:+	AACS
12:133446205:133446420:-	included	ENSG00000072609	12:133438050:133481480:-	CHFR
13:25460373:25460523:-	included	ENSG00000151849	13:25457170:25497018:-	CENPJ
13:31035776:31035825:-	included	ENSG00000189403	13:31033986:31038447:-	HMGB1
13:42795400:42795530:+	included	ENSG00000102780	13:42614175:42803294:+	DGKH
14:20923737:20923932:+	included	ENSG00000100823	14:20923349:20925927:+	APEX1
14:21990967:21993065:-	included	ENSG00000165821	14:21989672:21994634:-	SALL2
14:23302627:23302698:+	included	ENSG00000172590	14:23299091:23304246:+	MRPL52
14:23769316:23769534:-	included	ENSG00000235194	14:23764851:23772057:-	PPP1R3E
14:24025952:24026513:+	included	ENSG00000157306	14:24025196:24028118:+	RP11-66N24.4
14:45609835:45609912:+	included	ENSG00000187790	14:45605142:45636626:+	FANCM
14:52470958:52470960:+	included	ENSG00000087302	14:52470729:52471410:+	C14orf166

Table S1. Exons affected by SWI/SNF ATPase subunits

BRG1-mut

Affected exon coordinates	included/skipped	ENSEMBL gene ID	gene coordinates	Gene name
14:56068475:56068598:+	included	ENSG00000126777	14:56025789:56078948:+	KTN1
14:58832742:58832786:+	included	ENSG00000032219	14:58765102:58840451:+	ARID4A
14:61285440:61285565:+	included	ENSG00000020426	14:61263037:61285578:+	MNAT1
14:68120154:68120215:-	included	ENSG00000100568	14:68113791:68141548:-	VTI1B
14:69259599:69259787:-	included	ENSG00000185650	14:69255321:69261453:-	ZFP36L1
14:69925080:69925446:+	included	ENSG00000029364	14:69921417:69925260:+	SLC39A9
14:70855187:70855323:-	included	ENSG00000258644	14:70793068:70883701:-	SYNJ2BP-COX16
14:74345799:74346008:+	included	ENSG00000140043	14:74318546:74352159:+	PTGR2
14:74759857:74760013:-	included	ENSG00000119688	14:74752125:74769759:-	ABCD4
14:91044450:91044652:-	included	ENSG00000165914	14:91007529:91084367:-	TTC7B
14:93172828:93172998:-	included	ENSG00000100600	14:93170166:93183804:-	LGMN
14:102375926:102376027:+	included	ENSG00000078304	14:102368193:102378961:+	PPP2R5C
14:102744943:102745031:-	included	ENSG00000080823	14:102692621:102771531:-	MOK
14:102797959:102798183:+	included	ENSG00000022976	14:102797858:102805167:+	ZNF839
14:103420535:103420612:-	included	ENSG00000198752	14:103398715:103523799:-	CDC42BPB
15:20682178:20682309:-	included	ENSG00000180229	15:20667660:20682646:-	HERC2P3
15:23033894:23034006:-	included	ENSG00000140157	15:23019818:23034378:-	NIPA2
15:23200631:23200789:-	included	ENSG00000187667	15:23187727:23208417:-	WHAMMP3
15:25653767:25653831:-	included	ENSG00000114062	15:25582380:25684128:-	UBE3A
15:28878736:28878886:+	included	ENSG00000206149	15:28878244:28887539:+	HERC2P9
15:34517737:34517859:+	included	ENSG00000128463	15:34517218:34520784:+	EMC4
15:41191342:41193212:+	included	ENSG00000104142	15:41186627:41196173:+	VPS18
15:42820460:42820618:+	included	ENSG00000092531	15:42820140:42823849:+	SNAP23
15:43713223:43713372:-	included	ENSG00000067369	15:43699411:43748529:-	TP53BP1
15:45464345:45464516:-	included	ENSG00000138606	15:45459411:45480154:-	SHF
15:51018270:51018323:-	included	ENSG00000138600	15:51014337:51018524:-	SPPL2A
15:52861045:52861099:-	included	ENSG00000128989	15:52839241:52861436:-	ARPP19
15:59737408:59737461:+	included	ENSG00000157470	15:59736188:59752286:+	FAM81A
15:63929187:63929333:-	included	ENSG00000103657	15:63926161:63929749:-	HERC1
15:65243169:65243448:+	included	ENSG00000166839	15:65204100:65251039:+	ANKDD1A
15:66794126:66794250:-	included	ENSG00000174444	15:66790354:66797221:-	RPL4
15:74277000:74277212:-	included	ENSG00000067221	15:74275546:74284689:-	STOML1
15:75134876:75135015:-	included	ENSG00000140474	15:75128456:75135538:-	ULK3
15:75198619:75198706:-	included	ENSG00000178761	15:75192327:75199462:-	FAM219B
15:80367506:80367584:+	included	ENSG00000086666	15:80351909:80430735:+	ZFAND6
15:83087938:83087977:+	included	ENSG00000259429	15:83084256:83088121:+	UBE2Q2P3
15:83145928:83146023:-	included	ENSG00000205271	15:83133436:83145983:+	CSPG4P10
15:84873352:84873443:-	included	ENSG00000225151	15:84869931:84874024:-	GOLGA2P7
15:85752430:85752521:-	included	ENSG00000229212	15:85749008:85753102:-	RP11-561C5.4
15:89645731:89645807:+	included	ENSG00000140526	15:89630689:89698621:+	ABHD2
15:91485667:91485835:+	included	ENSG00000140553	15:91478514:91486314:+	UNC45A
15:91527923:91528055:-	included	ENSG00000198901	15:91509269:91537725:-	PRC1
15:102196070:102196223:-	included	ENSG00000185418	15:102194671:102204645:-	TARSL2
16:258600:258663:-	included	ENSG00000007392	16:239096:259772:-	LUC7L
16:1369703:1369863:+	included	ENSG00000103275	16:1361871:1375351:+	UBE2I
16:1717336:1717401:+	included	ENSG00000007545	16:1712462:1718064:+	CRAMP1L
16:2012737:2012910:-	included	ENSG00000140988	16:2012052:2014861:-	RPS2
16:2013150:2013257:-	included	ENSG00000140988	16:2012052:2014861:-	RPS2
16:2127599:2127727:+	included	ENSG00000103197	16:2126147:2129596:+	TSC2
16:2305586:2305727:-	included	ENSG00000205937	16:2303123:2306867:-	RNPS1
16:2719412:2722571:-	included	ENSG00000260565	16:2722326:2723445:-	ERVK13-1
16:2722708:2722977:-	included	ENSG00000260565	16:2722326:2723445:-	ERVK13-1

Table S1. Exons affected by SWI/SNF ATPase subunits

BRG1-mut

Affected exon coordinates	included/skipped	ENSEMBL gene ID	gene coordinates	Gene name
16:2825452:2825626:-	included	ENSG00000103363	16:2821414:2827298:-	TCEB2
16:3021158:3021379:+	included	ENSG00000127564	16:3018102:3030417:-	PKMYT1
16:3021275:3021379:+	included	ENSG00000127564	16:3018102:3030417:-	PKMYT1
16:3338454:3338570:+	included	ENSG00000006194	16:3332942:3341460:+	ZNF263
16:4393208:4393292:-	included	ENSG00000217930	16:4391652:4393694:-	PAM16
16:4504812:4504928:+	included	ENSG00000103423	16:4475805:4506776:+	DNAJA3
16:4562792:4563065:-	included	ENSG00000089486	16:4560739:4565110:-	CDIP1
16:4851268:4851322:-	included	ENSG00000067836	16:4851029:4852881:-	ROGDI
16:4927386:4927475:+	included	ENSG00000118900	16:4925272:4930446:+	UBN1
16:9023226:9023400:-	included	ENSG00000187555	16:8987652:9030586:-	USP7
16:11647389:11647604:-	included	ENSG00000189067	16:11643215:11650300:-	LITAF
16:12798481:12798906:-	included	ENSG00000103381	16:12756918:12897874:-	CPPED1
16:16165499:16165586:+	included	ENSG00000103222	16:16043433:16236931:+	ABCC1
16:16232221:16232415:+	included	ENSG00000103222	16:16043433:16236931:+	ABCC1
16:18439973:18439996:-	included	ENSG00000205746	16:18437996:18440175:-	RP11-1212A22.1
16:18799385:18799481:-	included	ENSG00000134419	16:18792616:18801656:-	RPS15A
16:20913808:20914039:+	included	ENSG00000102897	16:20911223:20935585:+	LYRM1
16:21430956:21431087:-	included	ENSG00000169246	16:21430989:21436657:-	NPIPB3
16:21865405:21865742:-	included	ENSG00000185864	16:21858802:21869003:-	NPIPB4
16:29095764:29095853:+	included	ENSG00000260908	16:29065052:29118766:-	CTB-134H23.3
16:29499344:29499412:-	included	ENSG00000169203	16:29496953:29506865:-	RP11-231C14.4
16:29511319:29511595:-	included	ENSG00000169203	16:29496010:29517141:-	RP11-231C14.4
16:29576672:29576827:-	included	ENSG00000205534	16:29538888:29606287:-	RP11-345J4.8
16:29820861:29821085:+	included	ENSG00000103495	16:29819095:29822484:+	MAZ
16:30006424:30006784:-	included	ENSG00000149929	16:30003644:30007757:-	HIRIP3
16:30077197:30077248:+	included	ENSG00000149925	16:30075492:30080278:+	ALDOA
16:30251287:30251563:-	included	ENSG00000198064	16:30246819:30257150:-	RP11-347C12.1
16:32163770:32163966:-	included	ENSG00000230267	16:32163039:32164308:-	HERC2P4
16:33263182:33263470:+	included	ENSG00000261509	16:33261514:33264716:+	TP53TG3B
16:48585297:48585393:-	included	ENSG00000102921	16:48580092:48587564:-	N4BP1
16:67211913:67212010:-	included	ENSG00000196123	16:67209506:67217943:-	KIAA0895L
16:69678700:69678759:+	included	ENSG00000102908	16:69599928:69719978:+	NFAT5
16:70165205:70165322:+	included	ENSG00000090857	16:70147528:70195184:+	PDPR
16:71322781:71322840:-	included	ENSG00000180917	16:71319552:71323330:-	CMTR2
16:81121206:81121269:-	included	ENSG00000140905	16:81117850:81129923:-	GCSH
16:84213552:84213689:-	included	ENSG00000103168	16:84211457:84220655:-	TAF1C
16:89627635:89627737:+	included	ENSG00000167526	16:89627064:89630949:+	RPL13
17:5321335:5321436:-	included	ENSG00000108559	17:5312056:5321549:-	NUP88
17:5329291:5331531:+	included	ENSG00000263272	17:5328458:5336196:-	CTC-524C5.2
17:7296920:7297155:-	included	ENSG00000187838	17:7293045:7297889:-	TMEM256-PLSCR3
17:7477578:7477996:+	included	ENSG00000161960	17:7476023:7480433:+	EIF4A1
17:7477584:7477626:+	included	ENSG00000161960	17:7476023:7480433:+	EIF4A1
17:7847794:7847956:+	included	ENSG00000170037	17:7835472:7852896:+	CNTROB
17:8079277:8079344:-	included	ENSG00000179029	17:8077316:8079706:-	TMEM107
17:8113495:8113567:-	included	ENSG00000178999	17:8110559:8113899:-	AURKB
17:16342842:16343017:+	included	ENSG00000175061	17:16342288:16345052:+	FAM211A-AS1
17:16342974:16343017:+	included	ENSG00000175061	17:16342288:16345052:+	FAM211A-AS1
17:18156913:18157033:-	included	ENSG00000177731	17:18154700:18157012:-	FLII
17:19235166:19235381:+	included	ENSG00000072134	17:19140653:19240028:+	EPN2
17:19459109:19459222:+	included	ENSG00000142494	17:19436774:19480910:+	SLC47A1
17:36291899:36292527:+	included	ENSG00000185128	17:36283970:36294912:+	TBC1D3F
17:36339906:36340198:-	included	ENSG00000197681	17:36337710:36347030:-	TBC1D3

Table S1. Exons affected by SWI/SNF ATPase subunits

BRG1-mut

Affected exon coordinates	included/skipped	ENSEMBL gene ID	gene coordinates	Gene name
17:37883548:37883800:+	included	ENSG00000141736	17:37844166:37884614:+	ERBB2
17:44707777:44707816:+	included	ENSG00000073969	17:44668034:44834830:+	NSF
17:48434418:48434613:+	included	ENSG00000015532	17:48433544:48435678:+	XYLT2
17:57043055:57043254:+	included	ENSG00000175175	17:56833229:57058983:+	PPM1E
17:59939271:59939436:-	included	ENSG00000136492	17:59934588:59940705:-	BRIP1
17:61490041:61490070:+	included	ENSG00000170921	17:61086916:61505060:+	TANC2
17:62746712:62746841:-	included	ENSG00000265298	17:62746537:62747429:+	RP13-104F24.3
17:62854897:62855001:-	included	ENSG00000176809	17:62850481:62859703:-	LRRC37A3
17:71223304:71223403:-	included	ENSG00000133193	17:71203491:71228510:-	FAM104A
17:73887894:73887959:-	included	ENSG00000141569	17:73876415:73888384:-	TRIM65
17:73888092:73888266:-	included	ENSG00000141569	17:73876415:73888384:-	TRIM65
17:74086410:74086562:-	included	ENSG00000182473	17:74077086:74099868:-	EXOC7
17:74679929:74680009:-	included	ENSG00000182534	17:74668632:74707037:-	MXRA7
17:79518758:79519078:-	included	ENSG00000185504	17:79516279:79520135:-	C17orf70
17:79892202:79892365:-	included	ENSG00000183010	17:79890259:79895204:-	PYCR1
17:79892529:79892621:-	included	ENSG00000183010	17:79890259:79895204:-	PYCR1
17:80616367:80616589:-	included	ENSG00000141542	17:80614943:80617465:-	RAB40B
18:3450320:3450503:+	included	ENSG00000177426	18:3411605:3457682:+	TGIF1
18:11906202:11906308:-	included	ENSG00000154889	18:11896981:11908307:-	MPPE1
18:19014908:19015036:+	included	ENSG00000141449	18:18943553:19105378:+	GREB1L
18:34340571:34340745:+	included	ENSG00000134775	18:33877676:34360018:+	FHOD3
18:54293393:54293497:-	included	ENSG00000091164	18:54264438:54305876:-	TXNL1
19:4501203:4501329:+	included	ENSG00000167674	19:4496329:4502116:+	HDGFRP2
19:6026174:6026248:-	included	ENSG00000087903	19:6007876:6026710:-	RFX2
19:10290863:10290910:-	included	ENSG00000130816	19:10244021:10305610:-	DNMT1
19:10463602:10463774:-	included	ENSG00000105397	19:10461208:10463932:-	TYK2
19:10657515:10657747:+	included	ENSG00000130734	19:10654570:10663800:+	ATG4D
19:11144443:11144541:+	included	ENSG00000127616	19:11141488:11152142:+	SMARCA4
19:11629890:11630008:-	included	ENSG00000130159	19:11616730:11639989:-	ECSIT
19:14593501:14593818:-	included	ENSG00000123159	19:14592558:14594275:-	GIPC1
19:15540397:15540900:-	included	ENSG00000011451	19:15532333:15544101:-	WIZ
19:19102149:19102362:-	included	ENSG00000064607	19:19101701:19102427:-	SUGP2
19:19166600:19166786:+	included	ENSG00000105676	19:19165039:19168763:+	ARMC6
19:32838151:32838244:+	included	ENSG00000168813	19:32836499:32878573:+	ZNF507
19:35760706:35760906:+	included	ENSG00000105698	19:35760010:35761420:+	USF2
19:36235392:36235441:-	included	ENSG00000267120	19:36230152:36236333:-	AD000671.6
19:41811551:41811772:+	included	ENSG00000105323	19:41768400:41813503:+	HNRNPUL1
19:41811737:41811772:+	included	ENSG00000105323	19:41768400:41813503:+	HNRNPUL1
19:41939177:41939339:-	included	ENSG00000105341	19:41937653:41942344:-	ATP5SL
19:42720832:42721197:-	included	ENSG00000160570	19:42719379:42721833:-	DEDD2
19:44530178:44530238:+	included	ENSG00000159885	19:44529493:44532538:+	ZNF222
19:45176984:45177117:+	included	ENSG00000186567	19:45174723:45187631:+	CEACAM19
19:45973887:45974207:+	included	ENSG00000125740	19:45973522:45975811:+	FOSB
19:46269608:46270413:-	included	ENSG00000177045	19:46268650:46272113:-	SIX5
19:49129325:49129619:+	included	ENSG00000063176	19:49122547:49133030:+	SPHK2
19:49605371:49606844:+	included	ENSG00000104852	19:49588675:49611855:+	SNRNP70
19:49669298:49669472:+	included	ENSG00000130529	19:49660997:49674630:+	TRPM4
19:49699697:49700131:+	included	ENSG00000130529	19:49685782:49715093:+	TRPM4
19:50167931:50168103:-	included	ENSG00000126456	19:50162828:50169132:-	IRF3
19:50727411:50727434:+	included	ENSG00000105357	19:50691442:50813047:+	MYH14
19:54372944:54373092:+	included	ENSG00000179820	19:54369476:54377510:+	MYADM
19:54373008:54373092:+	included	ENSG00000179820	19:54369476:54377510:+	MYADM

Table S1. Exons affected by SWI/SNF ATPase subunits

BRG1-mut

Affected exon coordinates	included/skipped	ENSEMBL gene ID	gene coordinates	Gene name
19:54682482:54682658:-	included	ENSG00000125505	19:54677112:54684507:-	MBOAT7
19:54710144:54710592:+	included	ENSG00000170889	19:54704609:54711515:+	RPS9
19:58196630:58196753:+	included	ENSG00000204519	19:58193336:58199396:+	ZNF551
2:11916212:11916319:+	included	ENSG00000134324	2:11864477:11924071:+	LPIN1
2:27258462:27258596:+	included	ENSG00000119777	2:27258387:27260532:+	TMEM214
2:43787373:43787524:-	included	ENSG00000115970	2:43779206:43819559:-	THADA
2:46818942:46819064:-	included	ENSG00000151665	2:46808075:46844258:-	PIGF
2:47136162:47136316:-	included	ENSG00000180398	2:47132955:47136211:-	MCFD2
2:65543868:65544017:-	included	ENSG00000198369	2:65537984:65659311:-	SPRED2
2:70215874:70215970:-	included	ENSG00000179818	2:70189901:70223973:-	PCBP1-AS1
2:73188260:73188377:-	included	ENSG00000144040	2:73171731:73208246:-	SFXN5
2:74428509:74428606:+	included	ENSG00000065911	2:74425688:74444692:+	MTHFD2
2:74692023:74692114:-	included	ENSG00000115275	2:74688183:74692537:-	MOGS
2:85582199:85582293:+	included	ENSG00000115459	2:85581516:85584148:+	ELMOD3
2:85582678:85583019:+	included	ENSG00000115459	2:85581516:85584148:+	ELMOD3
2:86078387:86078826:-	included	ENSG00000115525	2:86078542:86080236:-	ST3GAL5
2:103373872:103373998:+	included	ENSG00000170417	2:103353366:103414359:+	TMEM182
2:106497784:106498505:+	included	ENSG00000071051	2:106433014:106509632:+	NCK2
2:110350628:110350696:-	included	ENSG00000186522	2:110300558:110371783:-	SEPT10
2:111320189:111320334:-	included	ENSG00000183054	2:111315315:111320847:-	RGPD6
2:113174770:113174915:-	included	ENSG00000169629	2:113127668:113191222:-	RGPD8
2:113518290:113518341:-	included	ENSG00000169607	2:113514351:113522195:-	CKAP2L
2:120925456:120925583:+	included	ENSG00000115109	2:120918142:120925536:+	EPB41L5
2:122166600:122166623:-	included	ENSG00000074054	2:122165094:122185046:-	CLASP1
2:131897740:131897848:+	included	ENSG00000115762	2:131862419:131905370:+	PLEKHB2
2:132290297:132290354:+	included	ENSG00000163040	2:132285405:132291239:+	CCDC74A
2:175347738:175347886:-	included	ENSG00000163328	2:175347600:175351728:-	GPR155
2:179236853:179236960:+	included	ENSG00000079156	2:179184970:179260348:+	OSBPL6
2:179988442:179988556:-	included	ENSG00000187231	2:179974048:179989194:-	SESTD1
2:182785324:182785377:+	included	ENSG00000138434	2:182783587:182794404:+	SSFA2
2:197511065:197511228:+	included	ENSG00000144395	2:197504438:197522153:+	CCDC150
2:203075458:203075529:-	included	ENSG00000116030	2:203070902:203103331:-	SUMO1
2:219516424:219517023:-	included	ENSG00000115568	2:219515223:219521126:-	ZNF142
2:220081374:220081554:-	included	ENSG00000115657	2:220074489:220083712:-	ABCB6
2:220093732:220093896:-	included	ENSG00000198925	2:220089404:220094361:-	ATG9A
2:232655632:232655883:+	included	ENSG00000144524	2:232653398:232660855:+	COPS7B
2:236839409:236839567:+	included	ENSG00000157985	2:236792038:236945341:+	AGAP1
2:241465646:241465862:-	included	ENSG00000144504	2:241461355:241467298:-	ANKMY1
2:242447421:242447550:-	included	ENSG00000115694	2:242434431:242448103:-	STK25
20:330282:330476:+	included	ENSG00000125841	20:327748:334146:+	NRSN2
20:2638019:2638134:+	included	ENSG00000101361	20:2632790:2639039:+	NOP56
20:3095951:3096112:-	included	ENSG00000235958	20:3087558:3131513:+	UBOX5-AS1
20:3740654:3740833:-	included	ENSG00000101220	20:3734154:3748375:-	C20orf27
20:11898426:11898659:+	included	ENSG00000132640	20:11898564:11907243:+	BTBD3
20:20003100:20003124:+	included	ENSG00000173418	20:19998444:20003299:+	NAA20
20:31017704:31017856:+	included	ENSG00000171456	20:30946154:31027122:+	ASXL1
20:33871979:33872064:-	included	ENSG00000242372	20:33866713:33872788:-	EIF6
20:34761686:34761876:+	included	ENSG00000088367	20:34742663:34773257:+	EPB41L1
20:35219313:35219412:+	included	ENSG00000259716	20:35177529:35232932:-	RP5-977B1.11
20:45867502:45867882:-	included	ENSG00000101040	20:45837858:45984401:-	ZMYND8
20:45867640:45867882:-	included	ENSG00000101040	20:45837858:45984401:-	ZMYND8
20:49458303:49458437:+	included	ENSG00000124243	20:49411430:49493714:+	BCAS4

Table S1. Exons affected by SWI/SNF ATPase subunits

BRG1-mut

Affected exon coordinates	included/skipped	ENSEMBL gene ID	gene coordinates	Gene name
20:50407872:50408891:-	included	ENSG00000101115	20:50408606:50409617:-	SALL4
20:61833639:61835159:-	included	ENSG00000149658	20:61826780:61847586:-	YTHDF1
20:62562462:62562535:+	included	ENSG00000101152	20:62526517:62562472:+	DNAJC5
20:62657292:62657411:+	included	ENSG00000196700	20:62588054:62669938:-	ZNF512B
21:18924067:18924271:+	included	ENSG00000154639	21:18884699:18942418:+	CXADR
21:27369675:27369731:-	included	ENSG00000142192	21:27317838:27462377:-	APP
21:38878402:38878657:+	included	ENSG00000157540	21:38861086:38884862:+	DYRK1A
21:46281078:46281186:-	included	ENSG00000183255	21:46271321:46281848:-	PTTG1IP
21:46554630:46554747:+	included	ENSG00000197381	21:46540814:46596041:+	ADARB1
21:46604389:46604508:+	included	ENSG00000197381	21:46591556:46646475:+	ADARB1
22:20050861:20050965:+	included	ENSG00000183597	22:20040957:20052482:+	TANGO2
22:24219572:24220056:+	included	ENSG00000133460	22:24217346:24225139:+	SLC2A11
22:24939810:24940051:-	included	ENSG00000138867	22:24936499:24944000:-	GUCD1
22:28390461:28390641:+	included	ENSG00000100154	22:28379586:28392227:-	TTC28
22:28394592:28394826:+	included	ENSG00000235954	22:28393564:28394770:+	TTC28-AS1
22:29706817:29706988:+	included	ENSG00000185340	22:29706268:29707097:+	GAS2L1
22:29946717:29946832:-	included	ENSG00000100296	22:29935432:29949669:-	THOC5
22:30823109:30823390:+	included	ENSG00000242114	22:30821517:30825045:+	MTFP1
22:31368034:31368158:+	included	ENSG00000253352	22:31366662:31372049:+	TUG1
22:38609830:38609896:+	included	ENSG00000185022	22:38609541:38612512:+	MAFF
22:45255560:45255714:+	included	ENSG00000241484	22:45255540:45258343:+	ARHGAP8
22:51012237:51012350:-	included	ENSG00000205560	22:51007289:51016449:-	CPT1B
3:9510203:9510259:+	included	ENSG00000168137	3:9439402:9519838:+	SETD5
3:9785908:9786192:+	included	ENSG00000156983	3:9773412:9789557:+	BRPF1
3:9796388:9796569:+	included	ENSG00000114026	3:9791627:9808421:+	OGG1
3:9876365:9876591:+	included	ENSG00000214021	3:9870846:9877087:+	TTLL3
3:13524964:13525064:+	included	ENSG00000163517	3:13521223:13546834:+	HDAC11
3:15123874:15124115:-	included	ENSG00000131381	3:15123984:15127619:-	ZFYVE20
3:15132846:15133000:-	included	ENSG00000131381	3:15111579:15140655:-	ZFYVE20
3:15282960:15283095:+	included	ENSG00000131375	3:15247749:15294425:+	CAPN7
3:37860301:37860421:-	included	ENSG00000235257	3:37847319:37862591:-	AC093415.2
3:44544457:44544548:-	included	ENSG00000178917	3:44541067:44544602:-	ZNF852
3:47894653:47894842:-	included	ENSG00000047849	3:47892181:47951731:-	MAP4
3:49212495:49212596:+	included	ENSG00000185909	3:49209043:49213917:+	KLHDC8B
3:52130585:52130728:-	included	ENSG00000164087	3:52109268:52188428:-	POC1A
3:52456238:52456354:+	included	ENSG00000010318	3:52445212:52457656:+	PHF7
3:52588740:52588895:-	included	ENSG00000168273	3:52570620:52613253:+	SMIM4
3:57041210:57041364:-	included	ENSG00000163947	3:56761445:57113336:-	ARHGEF3
3:71823596:71823658:-	included	ENSG00000163421	3:71820806:71834357:-	PROK2
3:98240497:98240562:-	included	ENSG00000080822	3:98239562:98241746:-	CLDND1
3:124733557:124733856:-	included	ENSG00000173706	3:124732315:124738340:-	HEG1
3:129115060:129115263:-	included	ENSG00000251474	3:129109997:129118262:-	RPL32P3
3:136237090:136237257:-	included	ENSG00000118007	3:136055076:136471210:-	STAG1
3:142455221:142455375:+	included	ENSG00000144935	3:142442915:142525270:+	TRPC1
3:146254327:146254352:-	included	ENSG00000188313	3:146232966:146262651:-	PLSCR1
3:152173331:152173366:+	included	ENSG00000152601	3:152132846:152173366:+	MBNL1
3:160118388:160118753:+	included	ENSG00000113810	3:160117425:160120655:+	SMC4
3:167452002:167452133:-	included	ENSG00000114209	3:167401085:167452614:-	PDCD10
3:195609157:195609199:-	included	ENSG00000061938	3:195590234:195635955:-	TNK2
3:197417945:197418019:-	included	ENSG00000145016	3:197398263:197476570:-	KIAA0226
4:667701:667755:-	included	ENSG00000169020	4:666224:667910:-	ATP5I
4:1719243:1719430:-	included	ENSG00000168936	4:1718327:1719962:-	TMEM129

Table S1. Exons affected by SWI/SNF ATPase subunits

BRG1-mut

Affected exon coordinates	included/skipped	ENSEMBL gene ID	gene coordinates	Gene name
4:1729435:1730514:+	included	ENSG00000013810	4:1723261:1729959:+	TACC3
4:1953726:1953958:+	included	ENSG00000109685	4:1953831:1957885:+	WHSC1
4:20702294:20702410:+	included	ENSG00000163138	4:20702060:20704317:+	PACRGL
4:20703764:20703844:+	included	ENSG00000163138	4:20702060:20704317:+	PACRGL
4:76465069:76465169:+	included	ENSG00000174796	4:76439653:76469650:+	THAP6
4:83819142:83819215:-	included	ENSG00000138674	4:83800040:83822319:-	SEC31A
4:89658623:89658706:-	included	ENSG00000138640	4:89653272:89658790:-	FAM13A
4:99308587:99308658:+	included	ENSG00000138698	4:99273663:99313231:+	RAP1GDS1
4:100000971:100001142:-	included	ENSG00000197894	4:99993336:100006367:-	ADH5
4:103748749:103748898:-	included	ENSG00000109332	4:103715539:103748969:-	UBE2D3
4:106624805:106624966:-	included	ENSG00000138785	4:106603783:106629238:-	INTS12
4:124318766:124319011:+	included	ENSG00000164056	4:124317965:124319492:+	SPRY1
4:153456093:153456185:-	included	ENSG00000109670	4:153451424:153456362:-	FBXW7
4:160272211:160272285:+	included	ENSG00000109756	4:160271379:160275197:+	RAPGEF2
4:169927036:169927065:-	included	ENSG00000145439	4:169922319:169931405:-	CBR4
4:170926731:170927161:-	included	ENSG00000198948	4:170926809:170928126:-	MFAP3L
5:306717:306875:+	included	ENSG00000249915	5:271735:315089:+	PDCD6
5:884053:884185:-	included	ENSG00000028310	5:863850:889814:-	BRD9
5:10683641:10683683:-	included	ENSG00000112977	5:10679341:10761384:-	DAP
5:31515561:31515671:-	included	ENSG00000113360	5:31493315:31526527:-	DROSHA
5:34004679:34004839:-	included	ENSG00000242110	5:33997111:34008189:-	AMACR
5:68588015:68588189:-	included	ENSG00000183323	5:68576001:68628543:-	CCDC125
5:69364853:69364969:+	included	ENSG00000205571	5:69359237:69366582:+	SMN2
5:70240276:70240392:+	included	ENSG00000172062	5:70238298:70242105:+	SMN1
5:74026085:74026223:-	included	ENSG00000164347	5:74026084:74029213:-	GFM2
5:76441942:76442987:+	included	ENSG00000250802	5:76435073:76442447:+	ZBED3-AS1
5:122930733:122930828:+	included	ENSG00000151292	5:122847792:122952465:+	CSNK1G3
5:127412639:127412785:-	included	ENSG00000245937	5:127276117:127418792:-	CTC-228N24.3
5:130517922:130517992:+	included	ENSG00000186687	5:130506502:130541119:+	LYRM7
5:138268267:138268401:+	included	ENSG00000044115	5:138266326:138269851:+	CTNNA1
5:145560575:145560750:-	included	ENSG00000133706	5:145492600:145562223:-	LARS
5:149771107:149771358:+	included	ENSG00000070814	5:149769509:149772454:+	TCOF1
5:154173360:154173560:+	included	ENSG00000155506	5:154172292:154181569:+	LARP1
5:173359453:173359503:+	included	ENSG00000113742	5:173315282:173388979:+	CPEB4
5:177558238:177558377:+	included	ENSG00000145916	5:177558035:177569614:+	RMND5B
5:177637133:177637273:+	included	ENSG00000175309	5:177635623:177649477:-	PHYKPL
5:179044052:179044111:-	included	ENSG00000169045	5:179041178:179050711:-	HNRNPH1
5:179274978:179275066:-	included	ENSG00000161010	5:179274356:179285808:-	C5orf45
5:180666067:180666177:-	included	ENSG00000204628	5:180663908:180670916:-	GNB2L1
6:7229230:7232140:+	included	ENSG00000124782	6:7107829:7249507:+	RREB1
6:7246657:7247454:+	included	ENSG00000124782	6:7107829:7249507:+	RREB1
6:10751366:10751467:+	included	ENSG00000137210	6:10747991:10757214:+	TMEM14B
6:13974441:13974595:+	included	ENSG00000180537	6:13924676:13977503:+	RNF182
6:22174227:22174291:+	included	ENSG00000272168	6:22146882:22197448:+	CASC15
6:26091542:26091817:+	included	ENSG00000010704	6:26087508:26095445:+	HFE
6:26368203:26368278:+	included	ENSG00000186470	6:26365386:26370831:+	BTN3A2
6:28112109:28112202:+	included	ENSG00000198315	6:28109687:28121889:+	ZKSCAN8
6:30525927:30525989:+	included	ENSG00000204576	6:30524662:30531500:+	PRR3
6:30657802:30658020:-	included	ENSG00000137404	6:30655823:30658500:-	NRM
6:31607277:31607423:-	included	ENSG00000204463	6:31606804:31620482:-	BAG6
6:31726310:31726397:+	included	ENSG00000204410	6:31721059:31727278:+	MSH5
6:31940398:31940696:+	included	ENSG00000204344	6:31939607:31941153:+	STK19

Table S1. Exons affected by SWI/SNF ATPase subunits

BRG1-mut

Affected exon coordinates	included/skipped	ENSEMBL gene ID	gene coordinates	Gene name
6:32939372:32940704:+	included	ENSG00000204256	6:32936941:32939918:+	BRD2
6:32974857:32974992:-	included	ENSG00000204252	6:32971954:32977389:-	HLA-DOA
6:33280994:33281254:-	included	ENSG00000231925	6:33280790:33281732:-	TAPBP
6:33281471:33281641:-	included	ENSG00000231925	6:33280790:33281732:-	TAPBP
6:34204981:34205094:+	included	ENSG00000137309	6:34204649:34214007:+	HMGA1
6:35261528:35261692:+	included	ENSG00000065029	6:35260099:35262329:+	ZNF76
6:41048550:41048636:+	included	ENSG00000124596	6:41034677:41065497:-	OARD1
6:41904297:41904433:-	included	ENSG00000112576	6:41902670:41909586:-	CCND3
6:42851596:42851679:+	included	ENSG00000146223	6:42847355:42855039:+	RPL7L1
6:44224079:44224233:-	included	ENSG00000157593	6:44221832:44225291:-	SLC35B2
6:44224422:44224615:-	included	ENSG00000157593	6:44221832:44225291:-	SLC35B2
6:54001513:54003084:+	included	ENSG00000146147	6:53964362:54001623:+	MLIP
6:56333780:56333797:-	included	ENSG00000151914	6:56322857:56334754:-	DST
6:56482784:56485513:-	included	ENSG00000151914	6:56322784:56507794:-	DST
6:75949993:75950101:-	included	ENSG00000112695	6:75947392:75953644:-	COX7A2
6:83806450:83806542:+	included	ENSG00000083097	6:83777384:83878127:+	DOPEY1
6:99365250:99365595:-	included	ENSG00000112234	6:99316419:99395802:-	FBXL4
6:99863445:99863623:-	included	ENSG00000132424	6:99845926:99873184:-	PNISR
6:109466422:109466584:+	included	ENSG00000183137	6:109450506:109485113:+	CEP57L1
6:109797966:109798133:-	included	ENSG00000112365	6:109783796:109804440:-	ZBTB24
6:127652975:127653012:-	included	ENSG00000093144	6:127609856:127664020:-	ECHDC1
6:138763120:138763251:-	included	ENSG00000135540	6:138743179:138893677:-	NHSL1
6:158922710:158925210:+	included	ENSG00000130338	6:158733691:158932860:+	TULP4
6:161574378:161575342:-	included	ENSG00000026652	6:161551010:161695057:-	AGPAT4
7:889560:889670:+	included	ENSG00000164828	7:889437:892544:+	SUN1
7:12727260:12727353:+	included	ENSG00000122644	7:12726480:12728804:+	ARL4A
7:25164309:25164463:-	included	ENSG00000172115	7:25159709:25164980:-	CYCS
7:30590934:30591095:-	included	ENSG00000263683	7:30589832:30591185:+	RP4-777O23.1
7:33392471:33392485:+	included	ENSG00000122507	7:33169143:33645680:+	BBS9
7:35919206:35919329:+	included	ENSG00000122545	7:35840541:35944917:+	SEPT7
7:45025620:45025696:-	included	ENSG00000232956	7:45023445:45026267:-	SNHG15
7:51150755:51150925:-	included	ENSG00000106078	7:51083908:51261132:-	COBL
7:56146057:56146201:+	included	ENSG00000129103	7:56131694:56147817:+	SUMF2
7:65226633:65226735:+	included	ENSG00000228409	7:65216128:65228323:+	CCT6P1
7:72482243:72482355:+	included	ENSG00000229018	7:72476063:72491629:+	RP11-313P13.3
7:72952264:72952568:-	included	ENSG00000106635	7:72950685:72954898:-	BCL7B
7:73151259:73151550:-	included	ENSG00000106077	7:73150423:73153111:-	ABHD11
7:74995221:74995328:+	included	ENSG00000205583	7:74990643:74996355:+	STAG3L1
7:91874741:91874909:-	included	ENSG00000001631	7:91865724:91875228:-	KRIT1
7:97598317:97598364:-	included	ENSG00000243554	7:97598316:97601566:-	AC004967.7
7:99096339:99096465:-	included	ENSG00000160908	7:99090862:99097920:-	ZNF394
7:99159511:99160117:+	included	ENSG00000197343	7:99156028:99162328:+	ZNF655
7:99710972:99711028:-	included	ENSG00000106290	7:99704700:99712181:-	TAF6
7:99820220:99820343:-	included	ENSG00000239521	7:99798275:99869606:-	GATS
7:100454490:100454798:+	included	ENSG00000146828	7:100450340:100462980:+	SLC12A9
7:102036424:102036984:+	included	ENSG00000128563	7:102004318:102045072:+	PRKRIP1
7:102131433:102131570:-	included	ENSG00000170667	7:102122891:102133758:-	RASA4B
7:102143610:102143691:-	included	ENSG00000170667	7:102123589:102158157:-	RASA4B
7:102242771:102242852:-	included	ENSG00000105808	7:102240306:102246624:-	RASA4
7:102866661:102866826:-	included	ENSG00000170629	7:102815579:102920725:-	DPY19L2P2
7:106897177:106897239:-	included	ENSG00000164597	7:106876999:106899065:-	COG5
7:128587284:128587589:+	included	ENSG00000128604	7:128577993:128588886:+	IRF5

Table S1. Exons affected by SWI/SNF ATPase subunits

BRG1-mut

Affected exon coordinates	included/skipped	ENSEMBL gene ID	gene coordinates	Gene name
7:141413989:141414174:-	included	ENSG00000228775	7:141404137:141438030:-	WEE2-AS1
7:148724941:148725084:-	included	ENSG00000155660	7:148700153:148725733:-	PDIA4
7:149575767:149575879:+	included	ENSG00000171130	7:149570056:149577782:+	ATP6V0E2
7:150942546:150942757:-	included	ENSG00000082014	7:150937507:150942599:-	SMARCD3
7:156751300:156751550:+	included	ENSG00000146909	7:156746805:156752812:+	NOM1
7:156799173:156799333:-	included	ENSG00000130675	7:156797546:156802129:-	MNX1
8:12043861:12044126:-	included	ENSG00000186523	8:12042657:12051636:-	FAM86B1
8:12286142:12286407:-	included	ENSG00000145002	8:12282912:12293915:-	FAM86B2
8:12287859:12287960:-	included	ENSG00000145002	8:12282912:12293915:-	FAM86B2
8:21996152:21996306:-	included	ENSG00000168476	8:21995532:21999464:-	REEP4
8:22396982:22397011:+	included	ENSG00000120910	8:22298595:22398638:+	PPP3CC
8:22460050:22460156:+	included	ENSG00000241852	8:22457113:22461646:+	C8orf58
8:25246325:25246462:+	included	ENSG00000147459	8:25182941:25269772:+	DOCK5
8:38287200:38287466:-	included	ENSG00000077782	8:38282200:38287520:-	FGFR1
8:48641495:48641664:+	included	ENSG00000164808	8:48641281:48642266:+	SPIDR
8:56986254:56986327:-	included	ENSG00000008988	8:56985505:56987065:-	RPS20
8:67589877:67590189:+	included	ENSG00000213865	8:67588453:67593235:+	C8orf44
8:86127150:86127266:-	included	ENSG00000133740	8:86118397:86128438:+	E2F5
8:91643780:91643935:-	included	ENSG00000180694	8:91634222:91658311:-	TMEM64
8:96046236:96046350:+	included	ENSG00000156170	8:95970235:96070938:+	NDUFAF6
8:96064402:96064458:+	included	ENSG00000156170	8:95970235:96070938:+	NDUFAF6
8:101964157:101964536:-	included	ENSG00000164924	8:101960823:101964847:-	YWHAZ
8:133837280:133837402:+	included	ENSG00000129292	8:133829652:133859825:+	PHF20L1
8:141436714:141436740:-	included	ENSG00000167632	8:140742585:141468678:-	TRAPPC9
8:144671161:144672251:-	included	ENSG00000104529	8:144671350:144679612:-	EEF1D
8:144917990:144918045:-	included	ENSG00000185189	8:144917187:144919304:-	NRBP2
8:145735743:145735890:+	included	ENSG00000167700	8:145734456:145736596:+	MFSD3
9:4583038:4583172:+	included	ENSG00000106688	9:4572334:4585557:+	SLC1A1
9:5381863:5382031:-	included	ENSG00000107020	9:5357972:5437878:-	PLGRKT
9:34636994:34637086:-	included	ENSG00000147955	9:34635806:34637680:-	SIGMAR1
9:42471302:42471463:+	included	ENSG00000223839	9:42468588:42474236:+	FAM95B1
9:43030440:43030601:-	included	ENSG00000233244	9:43027747:43032877:-	CYP4F59P
9:90585483:90585545:-	included	ENSG00000156345	9:90581355:90589600:-	CDK20
9:91033693:91033866:+	included	ENSG00000106723	9:91003333:91093609:+	SPIN1
9:98690433:98690559:+	included	ENSG00000182150	9:98685634:98691399:+	ERCC6L2
9:117143340:117143726:-	included	ENSG00000106948	9:117096435:117156685:-	AKNA
9:130952608:130952723:-	included	ENSG00000148337	9:130941329:130953767:-	CIZ1
9:131219477:131219716:+	included	ENSG00000136811	9:131217464:131233625:+	ODF2
9:134369792:134369873:+	included	ENSG00000130723	9:134366151:134375584:+	PRRC2B
9:139562693:139562814:+	included	ENSG00000172889	9:139560239:139566445:+	EGFL7
X:6995261:6995490:-	included	ENSG00000130021	X:6966960:7066231:-	HDHD1
X:9621585:9621729:+	included	ENSG00000101849	X:9502982:9652110:+	TBL1X
X:13754227:13754415:+	included	ENSG00000046651	X:13752875:13754666:+	OFD1
X:14044259:14044340:-	included	ENSG00000046647	X:14026397:14048011:-	GEMIN8
X:47030427:47030657:+	included	ENSG00000182872	X:47004267:47046210:+	RBM10
X:48930092:48930309:-	included	ENSG00000243279	X:48928812:48931730:-	PRAF2
X:51637365:51637445:+	included	ENSG00000179222	X:51636741:51638848:+	MAGED1
X:64138922:64139116:-	included	ENSG00000126970	X:64137279:64140007:-	ZC4H2
X:102002761:102002839:+	included	ENSG00000198908	X:101975615:102003809:+	BHLHB9
X:102071920:102072010:+	included	ENSG00000223546	X:102024108:102094892:+	LINC00630
X:102631956:102632034:+	included	ENSG00000166681	X:102631267:102633005:+	NGFRAP1
X:102840787:102841219:+	included	ENSG00000133142	X:102840432:102841427:+	TCEAL4

Table S1. Exons affected by SWI/SNF ATPase subunits

BRG1-mut

Affected exon coordinates	included/skipped	ENSEMBL gene ID	gene coordinates	Gene name
X:117750165:117750203:+	included	ENSG00000147251	X:117629860:117820126:+	DOCK11
X:129484620:129484705:+	included	ENSG00000102078	X:129483221:129499010:+	SLC25A14
X:135961176:135961612:-	included	ENSG00000147274	X:135954433:135961590:-	RBMX
X:148608475:148608607:-	included	ENSG00000010404	X:148564073:148615470:-	IDS
X:151883336:151883646:+	included	ENSG00000183305	X:151883081:151885563:+	MAGEA2B
X:151883568:151883646:+	included	ENSG00000183305	X:151883081:151885563:+	MAGEA2B
X:151885385:151885450:+	included	ENSG00000183305	X:151883081:151885563:+	MAGEA2B
X:152857964:152858191:-	included	ENSG00000147382	X:152853512:152861521:-	FAM58A
X:153277980:153278129:-	included	ENSG00000184216	X:153275950:153285431:-	IRAK1
X:153627828:153627935:+	included	ENSG00000147403	X:153618314:153628507:+	RPL10
X:153631283:153631531:-	included	ENSG00000147403	X:153627706:153632038:+	RPL10
X:154300602:154300618:+	included	ENSG00000214827	X:154293437:154376212:-	MTCP1

Table S1. Exons affected by SWI/SNF ATPase subunits

BRM-wt

Affected exon coordinates	included/skipped	ENSEMBL gene ID	gene coordinates	Gene name
1:2124086:2124414:-	skipped	ENSG00000162585	1:2115916:2126180:-	C1orf86
1:3811859:3812747:-	skipped	ENSG00000198912	1:3805688:3816849:-	C1orf174
1:6078695:6078895:+	skipped	ENSG00000069424	1:6051525:6160235:+	KCNAB2
1:6293534:6293703:-	skipped	ENSG00000116237	1:6281252:6296000:-	ICMT
1:9165545:9165739:-	skipped	ENSG00000180758	1:9164590:9171429:-	GPR157
1:19452097:19452193:-	skipped	ENSG00000127481	1:19450970:19454195:-	UBR4
1:21548240:21548335:-	skipped	ENSG00000117298	1:21546306:21548574:-	ECE1
1:23667345:23667513:-	skipped	ENSG00000125944	1:23664762:23670810:-	HNRNPR
1:26153106:26153317:+	skipped	ENSG00000117640	1:26149486:26156288:+	MTFR1L
1:32542765:32542919:+	skipped	ENSG00000121775	1:32537631:32568464:+	TMEM39B
1:32683038:32683178:-	skipped	ENSG00000160055	1:32680072:32686797:-	TMEM234
1:40313658:40313769:-	skipped	ENSG00000043514	1:40307504:40313769:-	TRIT1
1:41232590:41232646:+	skipped	ENSG00000066136	1:41231761:41232603:+	NFYC
1:41618271:41618413:-	skipped	ENSG00000010803	1:41492871:41627104:-	SCMH1
1:43161870:43161959:+	skipped	ENSG00000065978	1:43148097:43168020:+	YBX1
1:44119334:44119470:+	skipped	ENSG00000066135	1:44115828:44171186:+	KDM4A
1:46812593:46812747:+	skipped	ENSG00000117481	1:46805848:46827863:+	NSUN4
1:52924008:52924121:-	skipped	ENSG00000134744	1:52898760:52928584:-	ZCCHC11
1:53370706:53372283:-	skipped	ENSG00000121310	1:53364845:53378502:-	ECHDC2
1:53722912:53723136:-	skipped	ENSG00000157193	1:53711420:53722995:-	LRP8
1:53723681:53723746:-	skipped	ENSG00000157193	1:53716436:53734270:-	LRP8
1:62619030:62619153:+	skipped	ENSG00000132849	1:62208148:62629592:+	INADL
1:85724618:85724744:-	skipped	ENSG00000162642	1:85723125:85725316:-	C1orf52
1:93621593:93621641:-	skipped	ENSG00000117500	1:93619752:93628767:-	TMED5
1:110774843:110775219:+	skipped	ENSG00000116396	1:110762337:110776666:+	KCNC4
1:114963002:114963073:-	skipped	ENSG00000197323	1:114935398:115053781:-	TRIM33
1:118420398:118420463:-	skipped	ENSG00000196505	1:118406106:118472253:-	GDAP2
1:146055345:146055447:-	skipped	ENSG00000152042	1:146032646:146082765:-	NBPF11
1:146065567:146065690:-	skipped	ENSG00000152042	1:146032646:146082765:-	NBPF11
1:146397359:146397461:+	skipped	ENSG00000186275	1:146373545:146467638:+	NBPF12
1:147607403:147607526:-	skipped	ENSG00000203836	1:147596183:147615861:-	NBPF24
1:151029127:151029262:-	skipped	ENSG00000197622	1:151028058:151032249:-	CDC42SE1
1:153599959:153600074:-	skipped	ENSG00000189171	1:153591262:153600724:-	S100A13
1:154192312:154192413:-	skipped	ENSG00000143612	1:154179181:154193082:-	C1orf43
1:156169626:156170263:+	skipped	ENSG00000160785	1:156169408:156169977:+	SLC25A44
1:156714293:156714362:-	skipped	ENSG00000143321	1:156713136:156722240:-	HDGF
1:163292657:163292885:+	skipped	ENSG00000143228	1:163291775:163298351:+	NUF2
1:171558508:171558744:+	skipped	ENSG00000117523	1:171454650:171560986:+	PRRC2C
1:199998161:199998856:+	skipped	ENSG00000116833	1:199996784:200013663:+	NR5A2
1:201804082:201804192:+	skipped	ENSG00000198700	1:201798276:201822201:+	IPO9
1:207243625:207243754:+	skipped	ENSG00000123836	1:207242768:207253020:+	PFKFB2
1:211485697:211485829:+	skipped	ENSG00000117625	1:211485578:211487181:+	RCOR3
1:213186435:213186760:-	skipped	ENSG00000174606	1:213179880:213186760:-	ANGEL2
1:227068170:227068425:+	skipped	ENSG00000143801	1:227057884:227069671:+	PSEN2
1:227838657:227838777:+	skipped	ENSG00000181450	1:227751243:227843529:+	ZNF678
1:228296138:228296209:-	skipped	ENSG00000162910	1:228295367:228297001:-	MRPL55
1:228328825:228329208:+	skipped	ENSG00000143774	1:228327662:228336541:+	GUK1
10:3822298:3822421:-	skipped	ENSG00000067082	10:3818187:3827467:-	KLF6
10:13364835:13365047:-	skipped	ENSG00000086475	10:13359423:13390297:-	SEPHS1
10:18947148:18947426:-	skipped	ENSG00000152487	10:18940194:18948196:-	ARL5B-AS1
10:27442156:27442210:-	skipped	ENSG00000136758	10:27399382:27443288:-	YME1L1
10:27462047:27462188:+	skipped	ENSG00000120539	10:27443752:27475848:+	MASTL

Table S1. Exons affected by SWI/SNF ATPase subunits

BRM-wt

Affected exon coordinates	included/skipped	ENSEMBL gene ID	gene coordinates	Gene name
10:35437296:35437419:+	skipped	ENSG00000095794	10:35415718:35501053:+	CREM
10:48235122:48235173:+	skipped	ENSG00000198035	10:48189611:48237508:+	AGAP9
10:49516138:49516331:+	skipped	ENSG00000107643	10:49514697:49618189:+	MAPK8
10:51338277:51338363:-	skipped	ENSG00000244393	10:51253925:51371300:-	RP11-592B15.3
10:51613218:51613311:-	skipped	ENSG00000138297	10:51592079:51623338:-	TIMM23
10:63974788:63974850:-	skipped	ENSG00000182010	10:63942793:63996022:-	RTKN2
10:64979638:64979743:-	skipped	ENSG00000171988	10:64926980:65225641:-	JMJD1C
10:74139222:74139341:-	skipped	ENSG00000107745	10:74127097:74310956:-	MICU1
10:74776604:74776657:-	skipped	ENSG00000122884	10:74769397:74776619:-	P4HA1
10:75898564:75898689:-	skipped	ENSG00000185009	10:75898013:75910515:-	AP3M1
10:88935646:88935852:+	skipped	ENSG00000122376	10:88854952:88951225:+	FAM35A
10:102016019:102016233:-	skipped	ENSG00000095485	10:101992054:102027437:-	CWF19L1
10:103364897:103364969:+	skipped	ENSG00000166171	10:103348044:103369425:+	DPCD
10:103368592:103368694:+	skipped	ENSG00000166171	10:103348044:103369425:+	DPCD
10:111892063:111892158:+	skipped	ENSG00000148700	10:111885512:111893106:+	ADD3
10:114849156:114849299:+	skipped	ENSG00000148737	10:114710008:114920420:+	TCF7L2
10:120864823:120865007:+	skipped	ENSG00000119979	10:120863597:120865147:+	FAM45A
10:120901765:120901885:-	skipped	ENSG00000183605	10:120900278:120916268:-	SFXN4
10:123903087:123903221:+	skipped	ENSG00000138162	10:123872544:123970084:+	TACC2
10:126501893:126502133:+	skipped	ENSG00000165660	10:126490353:126525239:+	FAM175B
10:127477852:127478097:-	skipped	ENSG00000188690	10:127477145:127483796:-	UROS
10:135193503:135193989:+	skipped	ENSG00000148832	10:135193042:135193877:+	PAOX
11:230452:230641:-	skipped	ENSG00000142082	11:230602:236345:-	SIRT3
11:504824:504996:-	skipped	ENSG00000023191	11:494512:506821:-	RNH1
11:908542:908645:-	skipped	ENSG00000177830	11:902196:915058:-	CHID1
11:1466201:1466232:+	skipped	ENSG00000174672	11:1463674:1466823:+	BRSK2
11:2423206:2423377:+	skipped	ENSG00000184281	11:2421985:2424473:+	TSSC4
11:2970457:2970494:-	skipped	ENSG00000205531	11:2965667:2973999:-	NAP1L4
11:3394807:3394905:-	skipped	ENSG00000005801	11:3392209:3400384:-	ZNF195
11:3838583:3838765:+	skipped	ENSG00000148985	11:3837418:3845402:+	PGAP2
11:10828802:10828866:-	skipped	ENSG00000110321	11:10818596:10829520:-	EIF4G2
11:14529202:14529312:-	skipped	ENSG00000129084	11:14526419:14532577:-	PSMA1
11:31491287:31491344:-	skipped	ENSG00000148950	11:31470576:31531139:-	IMMP1L
11:43356828:43356904:+	skipped	ENSG00000166181	11:43356828:43360923:+	API5
11:62401782:62401847:-	skipped	ENSG00000089597	11:62392297:62414087:-	GANAB
11:63486174:63488504:+	skipped	ENSG00000133318	11:63448917:63527354:+	RTN3
11:63523584:63523642:+	skipped	ENSG00000133318	11:63448917:63527354:+	RTN3
11:64082473:64082742:+	skipped	ENSG00000173153	11:64081242:64083362:+	ESRRA
11:65112051:65112092:+	skipped	ENSG00000133884	11:65111730:65120450:+	DPF2
11:66436086:66436762:-	skipped	ENSG00000173914	11:66433018:66436156:-	RBM4B
11:69489319:69489534:-	skipped	ENSG00000149716	11:69486145:69490184:-	ORAOV1
11:71809335:71809461:-	skipped	ENSG00000149357	11:71808337:71810304:-	LAMTOR1
11:74563034:74563127:-	skipped	ENSG00000166435	11:74559169:74563109:-	XRRA1
11:76075382:76075476:-	skipped	ENSG00000137492	11:76060999:76091986:-	PRKRIR
11:76250643:76250684:+	skipped	ENSG00000158636	11:76156068:76261387:+	C11orf30
11:82624329:82624428:+	skipped	ENSG00000165490	11:82611016:82639931:+	C11orf82
11:82624999:82625212:+	skipped	ENSG00000165490	11:82611016:82639931:+	C11orf82
11:82989769:82989872:-	skipped	ENSG00000137500	11:82985219:82997007:-	CCDC90B
11:94010123:94010275:+	skipped	ENSG00000250519	11:93971315:94012372:+	RP11-680H20.2
11:94528177:94528326:+	skipped	ENSG00000166025	11:94439596:94532705:+	AMOTL1
11:102219329:102219495:+	skipped	ENSG00000110330	11:102217941:102219744:+	BIRC2
11:111742075:111742213:-	skipped	ENSG00000258529	11:111741017:111747568:-	ALG9

Table S1. Exons affected by SWI/SNF ATPase subunits

BRM-wt

Affected exon coordinates	included/skipped	ENSEMBL gene ID	gene coordinates	Gene name
11:111956691:111957034:-	skipped	ENSG00000150779	11:111955523:111957522:-	TIMM8B
11:113705929:113706049:-	skipped	ENSG00000048028	11:113668595:113746292:-	USP28
11:114314578:114314655:+	skipped	ENSG00000076043	11:114311392:114320983:+	REXO2
11:117070349:117070545:+	skipped	ENSG00000149591	11:117070036:117075273:+	TAGLN
11:118897216:118897398:-	skipped	ENSG00000137700	11:118894823:118901616:-	SLC37A4
12:87881:88017:+	skipped	ENSG00000226210	12:73724:91218:+	ABC7-42389800N19.1
12:90104:90314:+	skipped	ENSG00000226210	12:73724:91218:+	ABC7-42389800N19.1
12:1748850:1749142:+	skipped	ENSG00000111186	12:1726221:1756377:+	WNT5B
12:2994328:2994700:+	skipped	ENSG00000171792	12:2985423:2997334:+	RHNO1
12:7092163:7092367:+	skipped	ENSG00000111684	12:7090720:7092626:-	LPCAT3
12:7260847:7261075:-	skipped	ENSG00000205885	12:7260647:7264202:+	C1RL-AS1
12:7354837:7354947:+	skipped	ENSG00000139197	12:7341319:7354939:+	PEX5
12:9845630:9845711:+	skipped	ENSG00000069493	12:9833520:9847725:+	CLEC2D
12:10761697:10761982:-	skipped	ENSG00000111196	12:10758611:10766222:-	MAGOHB
12:32891198:32891230:+	skipped	ENSG00000087470	12:32884419:32891230:+	DNM1L
12:39724044:39724064:-	skipped	ENSG00000139116	12:39723853:39726129:-	KIF21A
12:42604157:42604256:-	skipped	ENSG00000015153	12:42550905:42632050:-	YAF2
12:49349881:49349980:-	skipped	ENSG00000134287	12:49349934:49351245:-	ARF3
12:49498502:49498668:-	skipped	ENSG00000139636	12:49496135:49504633:-	LMBR1L
12:50481146:50481268:+	skipped	ENSG00000066117	12:50479107:50484109:+	SMARCD1
12:52437586:52437695:+	skipped	ENSG00000123358	12:52431025:52439352:+	NR4A1
12:53895799:53895968:+	skipped	ENSG00000139546	12:53894704:53900215:+	TARBP2
12:65700643:65700806:+	skipped	ENSG00000174099	12:65672483:65710097:+	MSRB3
12:69651815:69651925:+	skipped	ENSG00000111605	12:69633316:69668138:+	CPSF6
12:76467983:76468019:-	skipped	ENSG00000187109	12:76467584:76478463:-	NAP1L1
12:89744365:89744802:-	skipped	ENSG00000139318	12:89741008:89747048:-	DUSP6
12:100635421:100635623:-	skipped	ENSG00000166153	12:100631577:100646057:-	DEPDC4
12:109046814:109046836:-	skipped	ENSG00000110880	12:109038884:109125372:-	CORO1C
12:110937340:110937351:-	skipped	ENSG00000111237	12:110936600:110939912:-	VPS29
12:111082772:111082934:+	skipped	ENSG00000204852	12:111051831:111087235:+	TCTN1
12:118455495:118455657:+	skipped	ENSG00000111445	12:118451392:118462786:+	RFC5
12:120648733:120648909:+	skipped	ENSG00000255857	12:120639093:120650722:+	PXN-AS1
12:121881483:121881596:-	skipped	ENSG00000089094	12:121866899:122018364:-	KDM2B
12:123718287:123718471:+	skipped	ENSG00000051825	12:123712014:123728561:-	MPHOSPH9
12:123873980:123874101:+	skipped	ENSG00000183955	12:123874029:123875851:+	SETD8
12:124440831:124440991:-	skipped	ENSG00000119242	12:124420955:124457371:-	CCDC92
13:24159595:24159684:+	skipped	ENSG00000127863	13:24144802:24247638:+	TNFRSF19
13:33109906:33111164:-	skipped	ENSG00000244754	13:33101011:33110520:-	N4BP2L2
13:98659616:98659841:+	skipped	ENSG00000065150	13:98655262:98659841:+	IPO5
14:23421750:23421892:-	skipped	ENSG00000092036	14:23415436:23426363:-	HAUS4
14:23469166:23469326:-	skipped	ENSG00000100802	14:23465448:23471978:-	C14orf93
14:24036320:24036559:-	skipped	ENSG00000213983	14:24035547:24036956:-	AP1G2
14:24459984:24460161:+	skipped	ENSG00000187630	14:24439147:24475610:+	DHRS4L2
14:24629073:24629169:+	skipped	ENSG00000092098	14:24626499:24629814:+	RNF31
14:24707452:24707611:+	skipped	ENSG00000100938	14:24707393:24708045:+	GMPR2
14:35564254:35564390:-	skipped	ENSG00000092020	14:35554681:35564391:-	PPP2R3C
14:35782087:35782127:+	skipped	ENSG00000100902	14:35761648:35782105:+	PSMA6
14:37688758:37688896:+	skipped	ENSG00000151338	14:37667117:37737923:+	MIPOL1
14:50074118:50074839:+	skipped	ENSG00000165501	14:50065414:50081390:+	LRR1
14:50580088:50580230:-	skipped	ENSG00000100483	14:50575349:50583195:-	VCPKMT
14:58759784:58759856:-	skipped	ENSG00000257621	14:58732082:58764847:-	RP11-349A22.5
14:69919958:69920026:+	skipped	ENSG00000029364	14:69864731:69920017:+	SLC39A9

Table S1. Exons affected by SWI/SNF ATPase subunits

BRM-wt

Affected exon coordinates	included/skipped	ENSEMBL gene ID	gene coordinates	Gene name
14:73749067:73749213:-	skipped	ENSG00000133961	14:73748121:73753965:-	NUMB
14:75374138:75374233:-	skipped	ENSG00000198208	14:75372345:75374160:-	RPS6KL1
14:75517555:75517726:-	skipped	ENSG00000119684	14:75515871:75518207:-	MLH3
14:90390857:90391021:-	skipped	ENSG00000140025	14:90314776:90398938:-	EFCAB11
14:91611565:91611657:+	skipped	ENSG00000133943	14:91526676:91636545:+	C14orf159
14:96846025:96846125:+	skipped	ENSG00000100744	14:96846021:96853561:+	GSKIP
14:100341268:100341324:+	skipped	ENSG00000066629	14:100317186:100349883:+	EML1
14:100835424:100835595:-	skipped	ENSG00000140105	14:100828039:100841810:-	WARS
14:100840473:100840516:-	skipped	ENSG00000140105	14:100828039:100841810:-	WARS
14:104179212:104179268:-	skipped	ENSG00000126215	14:104170732:104181820:-	XRCC3
14:105455969:105456044:+	skipped	ENSG00000140104	14:105452111:105461227:+	C14orf79
15:32403991:32404100:+	skipped	ENSG00000175344	15:32322690:32461049:+	CHRNA7
15:34517737:34517859:+	skipped	ENSG00000128463	15:34517218:34520784:+	EMC4
15:34519894:34520047:+	skipped	ENSG00000128463	15:34517218:34520784:+	EMC4
15:40845794:40846326:-	skipped	ENSG00000128891	15:40846140:40857179:-	C15orf57
15:42852980:42853068:+	skipped	ENSG00000137814	15:42851581:42853885:+	HAUS2
15:43724392:43724875:-	skipped	ENSG00000067369	15:43699411:43748529:-	TP53BP1
15:45895298:45895385:+	skipped	ENSG00000104164	15:45879320:45901909:+	BLOC1S6
15:60688350:60688626:-	skipped	ENSG00000182718	15:60687845:60690185:-	ANXA2
15:60689457:60689537:-	skipped	ENSG00000182718	15:60687845:60690185:-	ANXA2
15:60812165:60812468:+	skipped	ENSG00000245534	15:60771376:60922836:+	RP11-219B17.1
15:65281507:65281611:-	skipped	ENSG00000090487	15:65275270:65282249:-	SPG21
15:65944008:65944097:+	skipped	ENSG00000074621	15:65935242:65946529:+	SLC24A1
15:75336727:75336855:+	skipped	ENSG00000138621	15:75335615:75342079:+	PPCDC
15:76587932:76588078:-	skipped	ENSG00000140374	15:76566823:76603754:-	ETFA
15:82970439:82970534:+	skipped	ENSG00000259328	15:82969810:82971216:+	RP11-152F13.7
15:84278022:84278218:+	skipped	ENSG00000140600	15:84115979:84287495:+	SH3GL3
15:84872929:84873083:-	skipped	ENSG00000225151	15:84869632:84873277:-	GOLGA2P7
15:91446461:91446964:+	skipped	ENSG00000196547	15:91445447:91448566:+	MAN2A2
15:91527136:91527370:-	skipped	ENSG00000198901	15:91509269:91537725:-	PRC1
15:99874128:99874334:+	skipped	ENSG00000168904	15:99791590:99874335:+	LRRC28
15:99874223:99874334:+	skipped	ENSG00000168904	15:99791590:99874335:+	LRRC28
16:448208:448394:+	skipped	ENSG00000103202	16:446724:450746:+	NME4
16:983991:984145:-	skipped	ENSG00000103227	16:921286:997644:-	LMF1
16:1369703:1369863:+	skipped	ENSG00000103275	16:1361871:1375351:+	UBE2I
16:2012874:2012910:-	skipped	ENSG00000140988	16:2012052:2014861:-	RPS2
16:3182851:3183195:-	skipped	ENSG00000263072	16:3178672:3184883:-	RP11-473M20.14
16:3336023:3336149:+	skipped	ENSG00000006194	16:3332942:3341460:+	ZNF263
16:4465716:4465853:-	skipped	ENSG00000262246	16:4457525:4465898:-	CORO7
16:14743661:14743830:+	skipped	ENSG00000103429	16:14726671:14763093:+	BFAR
16:15793586:15793711:+	skipped	ENSG00000072864	16:15781283:15793787:+	NDE1
16:18466580:18466711:-	skipped	ENSG00000233024	16:18451942:18468926:-	NPIPA7
16:21433127:21433464:-	skipped	ENSG00000169246	16:21430989:21436657:-	NPIPB3
16:21852124:21852332:-	skipped	ENSG00000185864	16:21848794:21854733:-	NPIPB4
16:23403283:23403555:-	skipped	ENSG00000168434	16:23399961:23404902:-	COG7
16:23568211:23568248:-	skipped	ENSG00000103356	16:23563512:23568676:-	EARS2
16:24789665:24789789:+	skipped	ENSG00000090905	16:24788568:24837548:+	TNRC6A
16:27279484:27279695:-	skipped	ENSG00000169189	16:27268662:27280045:-	NSMCE1
16:28847647:28847811:+	skipped	ENSG00000168488	16:28837090:28848558:+	ATXN2L
16:28884491:28884590:+	skipped	ENSG00000178188	16:28874879:28884948:+	SH2B1
16:29983363:29983461:+	skipped	ENSG00000149932	16:29952205:29984371:+	TMEM219
16:30094067:30094168:+	skipped	ENSG00000149923	16:30087298:30096100:+	PPP4C

Table S1. Exons affected by SWI/SNF ATPase subunits

BRM-wt

Affected exon coordinates	included/skipped	ENSEMBL gene ID	gene coordinates	Gene name
16:32265715:32266004:+	skipped	ENSG00000260575	16:32261940:32265743:-	RP11-56L13.7
16:56973807:56974000:+	skipped	ENSG00000051108	16:56969289:56976114:+	HERPUD1
16:57168687:57168738:+	skipped	ENSG00000140848	16:57153110:57171160:+	CPNE2
16:57208532:57208600:-	skipped	ENSG00000172775	16:57207692:57208798:-	FAM192A
16:68573661:68573728:+	skipped	ENSG00000184939	16:68563992:68592009:+	ZFP90
16:70434599:70434997:-	skipped	ENSG00000157350	16:70413337:70472991:-	ST3GAL2
16:75335655:75335803:-	skipped	ENSG00000153774	16:75327595:75340443:-	CFDP1
16:84531025:84531253:-	skipped	ENSG00000261243	16:84529204:84531101:+	RP11-517C16.4
16:88925753:88925851:+	skipped	ENSG00000167515	16:88922627:88927823:+	TRAPPC2L
16:89180747:89180895:+	skipped	ENSG00000261546	16:89179582:89181687:-	CTD-2555A7.3
16:89288500:89288591:+	skipped	ENSG00000170100	16:89284117:89289690:+	ZNF778
16:89645265:89645404:+	skipped	ENSG00000178773	16:89642189:89650122:+	CPNE7
17:641086:641287:+	skipped	ENSG00000167695	17:635651:646206:+	FAM57A
17:6916638:6916854:+	skipped	ENSG00000219200	17:6915735:6917851:+	RNASEK
17:8347245:8347494:+	skipped	ENSG00000166579	17:8339968:8351895:+	NDEL1
17:15554405:15555260:-	skipped	ENSG00000266261	17:15554903:15557803:+	RP11-640I15.1
17:17078607:17078726:+	skipped	ENSG00000133030	17:17075033:17088290:+	MPRIP
17:17924736:17924977:-	skipped	ENSG00000171953	17:17880722:17942482:-	ATPAF2
17:18156913:18157033:-	skipped	ENSG00000177731	17:18154700:18157012:-	FLII
17:18236480:18236602:-	skipped	ENSG00000176974	17:18231186:18266801:-	SHMT1
17:18607932:18608078:+	skipped	ENSG00000108448	17:18604669:18608078:+	TRIM16L
17:18775896:18775962:+	skipped	ENSG00000141127	17:18761408:18775941:+	PRPSAP2
17:20107646:20109225:+	skipped	ENSG00000128487	17:20058650:20108051:+	SPECC1
17:20790905:20791162:+	skipped	ENSG00000205212	17:20766707:20799453:-	CCDC144NL
17:25904518:25904665:+	skipped	ENSG00000141068	17:25904481:25912986:+	KSR1
17:26727626:26727783:-	skipped	ENSG00000076351	17:26722694:26733219:-	SLC46A1
17:29101110:29101362:-	skipped	ENSG00000176390	17:29099380:29113011:-	CRLF3
17:33445520:33445638:-	skipped	ENSG00000185379	17:33443962:33446888:-	RAD51D
17:36872620:36873234:+	skipped	ENSG00000263975	17:36871580:36876525:-	CTB-58E17.9
17:38065211:38065295:-	skipped	ENSG00000073605	17:38060847:38073569:-	GSDMB
17:38287790:38287837:+	skipped	ENSG00000188895	17:38282645:38288295:+	MSL1
17:41566817:41566902:+	skipped	ENSG00000067596	17:41561232:41569660:+	DHX8
17:42260298:42260461:-	skipped	ENSG00000267080	17:42255064:42264085:-	ASB16-AS1
17:42265275:42265377:+	skipped	ENSG00000168591	17:42264337:42268594:+	TMUB2
17:42266792:42266956:+	skipped	ENSG00000168591	17:42264337:42268594:+	TMUB2
17:42289712:42289822:-	skipped	ENSG00000108312	17:42289547:42290724:-	UBTF
17:42786672:42786734:+	skipped	ENSG00000161692	17:42785975:42829632:+	DBF4B
17:44067244:44067441:+	skipped	ENSG00000186868	17:44050148:44101797:+	MAPT
17:47784705:47784806:-	skipped	ENSG00000121073	17:47778304:47785317:-	SLC35B1
17:49053224:49053262:-	skipped	ENSG00000008294	17:49042435:49124239:-	SPAG9
17:53031022:53031120:-	skipped	ENSG00000166260	17:53029266:53032510:-	COX11
17:56424890:56424945:-	skipped	ENSG00000213246	17:56423272:56424972:-	SUPT4H1
17:61512447:61512604:-	skipped	ENSG00000008283	17:61509664:61518227:-	CYB561
17:65358892:65359008:-	skipped	ENSG00000197170	17:65334031:65362743:-	PSMD12
17:74725772:74725876:+	skipped	ENSG00000181038	17:74722911:74729598:+	METTL23
17:76392389:76392466:+	skipped	ENSG00000087157	17:76374729:76394349:+	PGS1
17:78252563:78252709:+	skipped	ENSG00000173821	17:78234664:78369112:+	RNF213
17:80007794:80007882:-	skipped	ENSG00000169733	17:80005777:80008390:-	RFNG
17:80398349:80398489:+	skipped	ENSG00000169660	17:80397875:80399704:+	HEXDC
18:13671861:13671972:-	skipped	ENSG00000177150	18:13666229:13671938:-	FAM210A
18:23658975:23659110:-	skipped	ENSG00000141380	18:23596577:23670589:-	SS18
18:33718758:33718835:+	skipped	ENSG00000134759	18:33718769:33725321:+	ELP2

Table S1. Exons affected by SWI/SNF ATPase subunits

BRM-wt

Affected exon coordinates	included/skipped	ENSEMBL gene ID	gene coordinates	Gene name
18:47017775:47017815:-	skipped	ENSG00000215472	18:47008050:47017956:-	RPL17-C18orf32
18:77488907:77489069:+	skipped	ENSG00000060069	18:77439800:77514510:+	CTDP1
18:77693969:77694022:-	skipped	ENSG00000122490	18:77662419:77711664:-	PQLC1
19:3542774:3542975:-	skipped	ENSG00000183397	19:3539151:3544028:+	C19orf71
19:5216731:5216778:-	skipped	ENSG00000105426	19:5215377:5220027:-	PTPRS
19:9947499:9947551:+	skipped	ENSG00000127445	19:9945998:9960358:+	PIN1
19:15540397:15540900:-	skipped	ENSG00000011451	19:15532333:15544101:-	WIZ
19:16623825:16623920:+	skipped	ENSG00000105072	19:16623879:16628931:+	C19orf44
19:16665963:16666208:-	skipped	ENSG00000127526	19:16664199:16675066:-	SLC35E1
19:17396490:17396628:+	skipped	ENSG00000160117	19:17392453:17397314:+	ANKLE1
19:19166600:19166786:+	skipped	ENSG00000105676	19:19165039:19168763:+	ARMC6
19:19291495:19291570:-	skipped	ENSG00000254901	19:19287740:19296907:-	MEF2BNB
19:21510762:21510943:-	skipped	ENSG00000182141	19:21473961:21512212:-	ZNF708
19:37316282:37316424:+	skipped	ENSG00000267254	19:37288464:37319003:+	CTD-2162K18.5
19:45176984:45177117:+	skipped	ENSG00000186567	19:45174723:45187631:+	CEACAM19
19:45996418:45996636:-	skipped	ENSG00000213889	19:45992034:46005557:+	PPM1N
19:48863329:48863433:-	skipped	ENSG00000161558	19:48863132:48867195:-	TMEM143
19:50315704:50315993:-	skipped	ENSG00000010361	19:50315536:50316541:-	FUZ
19:52503599:52503631:-	skipped	ENSG00000197619	19:52494584:52511334:-	ZNF615
19:52703527:52703651:+	skipped	ENSG00000105568	19:52693291:52730687:+	PPP2R1A
19:52817406:52817532:+	skipped	ENSG00000198464	19:52800429:52829172:+	ZNF480
19:55967003:55967212:-	skipped	ENSG00000063241	19:55964351:55973710:-	ISOC2
19:56880648:56880706:+	skipped	ENSG00000240225	19:56879704:56885126:+	ZNF542
19:58869318:58869652:-	skipped	ENSG00000174586	19:58868804:58874191:-	ZNF497
2:10950369:10950546:-	skipped	ENSG00000143870	2:10923516:10952970:-	PDIA6
2:11597305:11597359:-	skipped	ENSG00000169016	2:11584500:11606272:-	E2F6
2:26999240:26999350:+	skipped	ENSG00000213699	2:26987151:27004008:+	SLC35F6
2:27275201:27275411:+	skipped	ENSG00000084693	2:27265231:27276022:+	AGBL5
2:27354540:27354699:-	skipped	ENSG00000138073	2:27353623:27357543:-	PREB
2:30371111:30371407:+	skipped	ENSG00000119801	2:30370426:30371200:+	YPEL5
2:44586636:44587177:-	skipped	ENSG00000138078	2:44570943:44588952:-	PREPL
2:55455168:55455340:-	skipped	ENSG00000162994	2:55445069:55459490:-	CLHC1
2:55805383:55805478:-	skipped	ENSG00000138041	2:55774427:55844749:-	SMEK2
2:61237791:61238023:-	skipped	ENSG00000162927	2:61235915:61244566:-	PUS10
2:62107421:62107529:-	skipped	ENSG00000115484	2:62095223:62115939:-	CCT4
2:63486446:63486544:-	skipped	ENSG00000143951	2:63348517:63815933:-	WDPCP
2:73635750:73635875:+	skipped	ENSG00000116127	2:73612885:73836842:+	ALMS1
2:74783021:74783205:+	skipped	ENSG00000115325	2:74781212:74784681:+	DOK1
2:85787938:85788108:-	skipped	ENSG00000115486	2:85771845:85788632:-	GGCX
2:86380683:86380697:-	skipped	ENSG00000132305	2:86371054:86422893:-	IMMT
2:86398331:86398432:-	skipped	ENSG00000132305	2:86397913:86398700:-	IMMT
2:97399256:97399338:-	skipped	ENSG00000114988	2:97371665:97405800:-	LMAN2L
2:112237484:112237638:-	skipped	ENSG00000172965	2:112227214:112237838:-	MIR4435-1HG
2:120125135:120125220:+	skipped	ENSG00000155368	2:120124510:120126421:+	DBI
2:160104883:160105023:-	skipped	ENSG00000196151	2:160092303:160143310:-	WDSUB1
2:170870351:170870482:+	skipped	ENSG00000144357	2:170683967:170940641:+	UBR3
2:172563743:172563887:+	skipped	ENSG00000268821	2:172560843:172567314:-	AC068039.1
2:172951382:172951581:+	skipped	ENSG00000144355	2:172949953:172954405:+	DLX1
2:175263003:175263170:+	skipped	ENSG00000144306	2:175260468:175268938:+	SCRN3
2:175263094:175263170:+	skipped	ENSG00000144306	2:175260468:175268938:+	SCRN3
2:179396041:179396305:+	skipped	ENSG00000237298	2:179388630:179396307:+	TTN-AS1
2:186664424:186673860:+	skipped	ENSG00000231646	2:186648417:186664877:-	AC008174.3

Table S1. Exons affected by SWI/SNF ATPase subunits

BRM-wt

Affected exon coordinates	included/skipped	ENSEMBL gene ID	gene coordinates	Gene name
2:201852088:201852255:-	skipped	ENSG00000183308	2:201827985:201873825:+	AC005037.3
2:202141550:202141827:+	skipped	ENSG00000064012	2:202122702:202141627:+	CASP8
2:203084755:203084829:-	skipped	ENSG00000116030	2:203070902:203103331:-	SUMO1
2:204143296:204143438:+	skipped	ENSG00000119004	2:204103662:204162068:+	CYP20A1
2:207025611:207025856:+	skipped	ENSG00000114942	2:207024308:207027652:+	EEF1B2
2:219146663:219146904:-	skipped	ENSG00000135926	2:219138923:219150657:-	TMBIM1
2:219492752:219494386:+	skipped	ENSG00000115556	2:219472487:219501904:+	PLCD4
2:241535351:241535569:+	skipped	ENSG00000142330	2:241534562:241536119:+	CAPN10
2:242047882:242048222:-	skipped	ENSG00000115687	2:242045709:242049195:-	PASK
2:242259618:242259702:+	skipped	ENSG00000168385	2:242255308:242264659:+	SEPT2
20:2638019:2638134:+	skipped	ENSG00000101361	20:2632790:2639039:+	NOP56
20:13782464:13782519:+	skipped	ENSG00000101247	20:13782213:13784067:+	NDUFAF5
20:23350221:23350378:+	skipped	ENSG00000125812	20:23349507:23351058:+	GZF1
20:34262967:34263124:-	skipped	ENSG00000244005	20:34262452:34270713:-	NFS1
20:34328447:34328519:-	skipped	ENSG00000131051	20:34327024:34330149:-	RBM39
20:44047492:44047619:+	skipped	ENSG00000124155	20:44044716:44054884:+	PIGT
20:44050023:44050223:+	skipped	ENSG00000124155	20:44044716:44054884:+	PIGT
20:48767749:48767852:-	skipped	ENSG00000124208	20:48697660:48770174:-	TMEM189-UBE2V1
20:55968335:55968389:+	skipped	ENSG00000132819	20:55966462:55984389:+	RBM38
20:60879517:60879633:+	skipped	ENSG00000130706	20:60877148:60879597:+	ADRM1
20:62367133:62369000:+	skipped	ENSG00000203896	20:62366814:62369794:+	LIME1
20:62579578:62579828:-	skipped	ENSG00000198276	20:62571276:62582479:-	UCKL1
21:34648901:34649058:+	skipped	ENSG00000249624	21:34619078:34655517:+	AP000295.9
21:34809190:34809299:-	skipped	ENSG00000159128	21:34775201:34809828:+	IFNGR2
21:35199122:35199167:+	skipped	ENSG00000205726	21:35190329:35201333:+	ITSN1
21:45380513:45380660:+	skipped	ENSG00000160216	21:45366810:45404554:+	AGPAT3
21:45482934:45483049:+	skipped	ENSG00000160218	21:45432199:45526433:+	TRAPPC10
21:45516486:45516594:+	skipped	ENSG00000160218	21:45516460:45524932:+	TRAPPC10
21:46221199:46221313:-	skipped	ENSG00000184787	21:46219904:46221723:-	UBE2G2
22:17990845:17990928:+	skipped	ENSG00000099954	22:17840836:18018834:+	CECR2
22:20040960:20041074:+	skipped	ENSG00000183597	22:20040957:20052482:+	TANGO2
22:20843272:20843531:-	skipped	ENSG00000099910	22:20842817:20846340:-	KLHL22
22:21829500:21829589:-	skipped	ENSG00000183506	22:21827288:21838423:-	PI4KAP2
22:22330277:22330559:-	skipped	ENSG00000100038	22:22328790:22330341:-	TOP3B
22:24225959:24226616:+	skipped	ENSG00000133460	22:24220044:24226971:+	SLC2A11
22:24672668:24672771:+	skipped	ENSG00000100014	22:24666789:24726369:+	SPECC1L
22:32016539:32016585:-	skipped	ENSG00000241878	22:32014476:32024207:-	PISD
22:32023989:32024196:-	skipped	ENSG00000241878	22:32014476:32024207:-	PISD
22:34252728:34252790:-	skipped	ENSG00000133424	22:33668846:34316409:-	LARGE
22:39052961:39053148:+	skipped	ENSG00000100211	22:39052640:39064472:+	CBY1
22:41985056:41985167:-	skipped	ENSG00000100417	22:41979986:41985800:-	PMM1
22:42300539:42300736:+	skipped	ENSG00000198911	22:42294679:42300977:+	SREBF2
3:9731648:9731827:+	skipped	ENSG00000163719	3:9710426:9744071:+	MTMR14
3:9796388:9796569:+	skipped	ENSG00000114026	3:9791627:9808421:+	OGG1
3:9798155:9798305:+	skipped	ENSG00000114026	3:9791627:9808421:+	OGG1
3:10360823:10361000:-	skipped	ENSG00000157020	3:10354763:10362796:-	SEC13
3:14163417:14163586:-	skipped	ENSG00000163528	3:14153579:14166370:-	CHCHD4
3:48341921:48342124:-	skipped	ENSG00000172113	3:48334753:48342848:-	NME6
3:48502760:48502844:+	skipped	ENSG00000164053	3:48488136:48507054:+	ATRIP
3:48581013:48581079:-	skipped	ENSG00000114268	3:48555116:48594323:-	PFKFB4
3:48731892:48731958:-	skipped	ENSG00000068745	3:48726970:48733021:-	IP6K2
3:49948959:49949444:-	skipped	ENSG00000228008	3:49941419:49954370:+	CTD-2330K9.3

Table S1. Exons affected by SWI/SNF ATPase subunits

BRM-wt

Affected exon coordinates	included/skipped	ENSEMBL gene ID	gene coordinates	Gene name
3:50599804:50600019:-	skipped	ENSG00000088543	3:50595461:50605096:-	C3orf18
3:51995765:51996104:-	skipped	ENSG00000090097	3:51991469:52001473:-	PCBP4
3:52005476:52005908:-	skipped	ENSG00000114779	3:52003099:52008037:-	ABHD14B
3:52737520:52737714:-	skipped	ENSG00000016864	3:52728504:52739807:-	GLT8D1
3:56606365:56606456:+	skipped	ENSG00000180376	3:56600688:56606938:+	CCDC66
3:58117654:58117746:+	skipped	ENSG00000136068	3:57994126:58157978:+	FLNB
3:58419358:58419411:-	skipped	ENSG00000168291	3:58413356:58419584:-	PDHB
3:111709547:111709598:+	skipped	ENSG00000144827	3:111697856:111711118:+	ABHD10
3:119222801:119222868:+	skipped	ENSG00000113845	3:119217378:119243937:+	TIMMDC1
3:119232488:119232566:+	skipped	ENSG00000113845	3:119217378:119243937:+	TIMMDC1
3:130887674:130887781:+	skipped	ENSG00000114670	3:130745693:130903595:+	NEK11
3:131220688:131220768:-	skipped	ENSG00000114686	3:131206524:131221795:-	MRPL3
3:152164493:152164546:+	skipped	ENSG00000152601	3:152132846:152173366:+	MBNL1
3:172469940:172470262:+	skipped	ENSG00000114346	3:172468748:172472375:+	ECT2
3:184640217:184640271:+	skipped	ENSG00000156931	3:184633170:184644434:+	VPS8
4:2243247:2243564:-	skipped	ENSG00000214367	4:2229190:2243848:-	HAUS3
4:40101656:40101746:+	skipped	ENSG00000078177	4:40058555:40104226:+	N4BP2
4:48712536:48712715:-	skipped	ENSG00000075539	4:48499379:48782316:-	FRYL
4:48835418:48835498:+	skipped	ENSG00000109180	4:48833079:48835446:+	OCIAD1
4:53524341:53524448:-	skipped	ENSG00000109189	4:53457137:53525502:-	USP46
4:54306749:54306775:+	skipped	ENSG00000145216	4:54292002:54325788:+	FIP1L1
4:57211293:57211456:-	skipped	ENSG00000157426	4:57204455:57253666:-	AASDH
4:83763293:83763634:-	skipped	ENSG00000138674	4:83739813:83812419:-	SEC31A
4:84028957:84029103:-	skipped	ENSG00000145287	4:84011200:84035955:-	PLAC8
4:89199688:89199794:-	skipped	ENSG00000163644	4:89196075:89200852:-	PPM1K
4:103747642:103747776:-	skipped	ENSG00000109332	4:103715539:103748969:-	UBE2D3
4:120379126:120379325:+	skipped	ENSG00000245958	4:120375945:120381399:+	RP11-33B1.1
4:123818779:123818833:-	skipped	ENSG00000138685	4:123747862:123819391:+	FGF2
4:177017614:177017736:+	skipped	ENSG00000150627	4:176987027:177032444:+	WDR17
4:183837826:183838010:-	skipped	ENSG00000129187	4:183836075:183838498:-	DCTD
4:185569619:185569785:-	skipped	ENSG00000164305	4:185548849:185570629:-	CASP3
5:304292:304336:+	skipped	ENSG00000249915	5:271735:315089:+	PDCD6
5:1293428:1294781:-	skipped	ENSG00000164362	5:1294672:1295184:-	TERT
5:32235068:32235235:-	skipped	ENSG00000150712	5:32227099:32313044:-	MTMR12
5:52095719:52096954:+	skipped	ENSG00000152684	5:52083773:52099880:+	PELO
5:54998567:54998722:-	skipped	ENSG00000177058	5:54921672:55008554:-	SLC38A9
5:68590620:68590727:-	skipped	ENSG00000183323	5:68576001:68628543:-	CCDC125
5:69216979:69217071:+	skipped	ENSG00000198237	5:69216552:69274436:+	RP11-98J23.2
5:70092028:70092120:+	skipped	ENSG00000253366	5:70091601:70149508:+	RP11-589F5.4
5:87517491:87517577:-	skipped	ENSG00000164180	5:87485449:87564647:-	TMEM161B
5:89692310:89692381:-	skipped	ENSG00000153140	5:89688077:89705603:-	CETN3
5:94916596:94916646:+	skipped	ENSG00000164291	5:94890777:94940768:+	ARSK
5:94988821:94988918:+	skipped	ENSG00000175449	5:94982457:94991894:+	RFESD
5:123976945:123977098:-	skipped	ENSG00000168916	5:123972607:123980030:-	ZNF608
5:132202561:132202727:+	skipped	ENSG00000164404	5:132198157:132202576:-	GDF9
5:137853250:137853389:-	skipped	ENSG00000120705	5:137846792:137853351:-	ETF1
5:141980267:141980370:-	skipped	ENSG00000231185	5:141961412:142006755:+	AC005592.2
5:147806776:147807510:+	skipped	ENSG00000145868	5:147803311:147806833:+	FBXO38
5:149824042:149824204:-	skipped	ENSG00000164587	5:149822752:149829310:-	RPS14
5:153417853:153417994:-	skipped	ENSG00000055147	5:153406854:153418481:-	FAM114A2
5:154239255:154239433:+	skipped	ENSG00000155508	5:154239226:154247293:+	CNOT8
5:180235648:180236076:-	skipped	ENSG00000131446	5:180235525:180237920:-	MGAT1

Table S1. Exons affected by SWI/SNF ATPase subunits

BRM-wt

Affected exon coordinates	included/skipped	ENSEMBL gene ID	gene coordinates	Gene name
6:2969013:2969147:-	skipped	ENSG00000124570	6:2948392:2972090:-	SERPINB6
6:3003903:3004023:+	skipped	ENSG00000124588	6:2988220:3012795:+	NQO2
6:7240671:7240835:+	skipped	ENSG00000124782	6:7107829:7249507:+	RREB1
6:10687713:10687794:-	skipped	ENSG00000137434	6:10671650:10694766:-	C6orf52
6:15660614:15660752:-	skipped	ENSG00000047579	6:15523031:15663273:-	DTNBP1
6:22063249:22063469:+	skipped	ENSG00000272168	6:21666674:22194616:+	CASC15
6:26094414:26094446:+	skipped	ENSG00000010704	6:26087508:26095445:+	HFE
6:26368218:26368495:+	skipped	ENSG00000186470	6:26365386:26370831:+	BTN3A2
6:26368406:26368495:+	skipped	ENSG00000186470	6:26365386:26370831:+	BTN3A2
6:26384020:26384143:+	skipped	ENSG00000124508	6:26383328:26392950:+	BTN2A2
6:26443798:26443887:+	skipped	ENSG00000111801	6:26440699:26453642:+	BTN3A3
6:27219529:27219819:+	skipped	ENSG00000112812	6:27215479:27224232:+	PRSS16
6:28318049:28318165:+	skipped	ENSG00000189298	6:28317690:28334523:+	ZKSCAN3
6:31696642:31697002:-	skipped	ENSG00000213722	6:31694814:31697569:-	DDAH2
6:33289496:33289785:-	skipped	ENSG00000204209	6:33286334:33290787:-	DAXX
6:36193048:36193141:+	skipped	ENSG00000096070	6:36179224:36200564:+	BRPF3
6:41037815:41037873:-	skipped	ENSG00000124596	6:41032516:41040330:-	OARD1
6:41086980:41087202:+	skipped	ENSG00000161912	6:41068760:41106465:+	ADCY10P1
6:41884523:41884677:-	skipped	ENSG00000124641	6:41884593:41888843:-	MED20
6:43154698:43154833:+	skipped	ENSG00000112659	6:43149912:43192325:+	CUL9
6:44193798:44193904:+	skipped	ENSG00000112759	6:44191301:44201878:+	SLC29A1
6:53986245:53986400:+	skipped	ENSG00000146147	6:53948222:53989415:+	MLIP
6:90962232:90962309:-	skipped	ENSG00000112182	6:90636247:91006461:-	BACH2
6:109427238:109427293:+	skipped	ENSG00000183137	6:109416312:109479691:+	CEP57L1
6:109690125:109690220:-	skipped	ENSG00000135535	6:109687716:109703660:-	CD164
6:109691614:109691670:-	skipped	ENSG00000135535	6:109687716:109703660:-	CD164
6:111805947:111806064:+	skipped	ENSG00000231889	6:111804713:111814206:+	TRAF3IP2-AS1
6:116981396:116981532:-	skipped	ENSG00000153975	6:116956780:116989957:-	ZUFSP
6:119281934:119282028:-	skipped	ENSG00000111879	6:119280993:119285925:-	FAM184A
6:130370796:130370975:+	skipped	ENSG00000198945	6:130339727:130372488:+	L3MBTL3
6:152621776:152621916:-	skipped	ENSG00000131018	6:152598636:152623404:-	SYNE1
7:2265045:2265185:-	skipped	ENSG00000002822	7:2262209:2272580:-	MAD1L1
7:2584543:2584690:-	skipped	ENSG00000106009	7:2577510:2595361:-	BRAT1
7:12253827:12254020:+	skipped	ENSG00000106460	7:12250948:12254511:+	TMEM106B
7:14028893:14029089:-	skipped	ENSG00000006468	7:14027783:14029291:-	ETV1
7:16643174:16643285:-	skipped	ENSG00000106524	7:16639400:16685442:-	ANKMY2
7:23157453:23157583:+	skipped	ENSG00000122550	7:23145376:23183520:+	KLHL7
7:23570800:23570889:-	skipped	ENSG00000164548	7:23544398:23571660:-	TRA2A
7:30540152:30540297:-	skipped	ENSG00000006625	7:30536236:30544460:-	GGCT
7:42966982:42967058:-	skipped	ENSG00000256646	7:42948871:42971773:-	PSMA2
7:44092466:44092550:+	skipped	ENSG00000136279	7:44089482:44101236:+	DBNL
7:44161881:44162058:-	skipped	ENSG00000106628	7:44155715:44163136:-	POLD2
7:44880498:44880611:-	skipped	ENSG00000105968	7:44866389:44887676:-	H2AFV
7:64150777:64150903:+	skipped	ENSG00000196247	7:64126510:64166957:+	ZNF107
7:64275954:64276031:+	skipped	ENSG00000197008	7:64254765:64292580:+	ZNF138
7:65445211:65445396:-	skipped	ENSG00000169919	7:65439343:65445398:-	GUSB
7:66233818:66234012:+	skipped	ENSG00000243335	7:66205642:66276446:+	KCTD7
7:72470881:72471004:-	skipped	ENSG00000174353	7:72467581:72476441:-	STAG3L3
7:72514920:72515008:+	skipped	ENSG00000174384	7:72507940:72515008:+	RP11-313P13.4
7:74158478:74158589:+	skipped	ENSG00000077809	7:74072010:74175022:+	GTF2I
7:74317004:74317092:+	skipped	ENSG00000123965	7:74306893:74322330:+	PMS2P5
7:74712252:74712364:-	skipped	ENSG00000189011	7:74702427:74712565:-	AC138783.12

Table S1. Exons affected by SWI/SNF ATPase subunits

BRM-wt

Affected exon coordinates	included/skipped	ENSEMBL gene ID	gene coordinates	Gene name
7:86974359:86974549:-	skipped	ENSG00000182165	7:86974512:86974802:-	TP53TG1
7:91873316:91873463:-	skipped	ENSG00000001631	7:91865724:91875228:-	KRIT1
7:92214612:92214710:-	skipped	ENSG00000234545	7:92210819:92219344:-	FAM133B
7:99075897:99075979:+	skipped	ENSG00000198556	7:99070463:99085217:+	ZNF789
7:99773195:99773517:-	skipped	ENSG00000213420	7:99767228:99774990:-	GPC2
7:99923314:99923402:-	skipped	ENSG00000078319	7:99918614:99939531:-	PMS2P1
7:99987511:99987729:+	skipped	ENSG00000085514	7:99965152:99996925:+	PILRA
7:100402788:100402943:-	skipped	ENSG00000196411	7:100400186:100425121:-	EPHB4
7:100860447:100860699:-	skipped	ENSG00000106397	7:100849257:100860954:-	PLOD3
7:102228681:102228892:-	skipped	ENSG00000105808	7:102222796:102234438:-	RASA4
7:102724477:102724509:+	skipped	ENSG00000170632	7:102715327:102740205:+	ARMC10
7:104748991:104749105:+	skipped	ENSG00000005483	7:104748346:104751003:+	KMT2E
7:128040871:128040945:-	skipped	ENSG00000106348	7:128032330:128045919:-	IMPDH1
7:130652148:130652276:-	skipped	ENSG00000231721	7:130628926:130668868:-	LINC-PINT
7:135926898:135927106:+	skipped	ENSG00000232053	7:135777672:136121911:+	AC009784.3
7:139737515:139737656:-	skipped	ENSG00000059378	7:139723543:139763521:-	PARP12
7:140045669:140045770:-	skipped	ENSG00000157800	7:140034669:140045711:-	SLC37A3
7:143078650:143078872:+	skipped	ENSG00000159840	7:143078387:143080018:+	ZYX
7:143530340:143530976:-	skipped	ENSG00000253882	7:143530337:143530962:-	RP11-61L23.2
7:150066831:150066957:+	skipped	ENSG00000214022	7:150065277:150068769:+	REPIN1
7:150067803:150067973:+	skipped	ENSG00000214022	7:150065277:150068769:+	REPIN1
7:150729931:150730148:+	skipped	ENSG00000197150	7:150725509:150730929:+	ABCB8
8:12286142:12286407:-	skipped	ENSG00000145002	8:12282912:12293915:-	FAM86B2
8:22449064:22449181:+	skipped	ENSG00000248235	8:22446786:22459597:+	AC037459.4
8:27155981:27156076:-	skipped	ENSG00000104228	8:27142403:27168836:-	TRIM35
8:27954736:27954835:+	skipped	ENSG00000134014	8:27947189:27965481:+	ELP3
8:33364747:33364839:-	skipped	ENSG00000129696	8:33330903:33367303:-	TTI2
8:37963549:37963657:+	skipped	ENSG00000129691	8:37963017:37968329:+	ASH2L
8:38251616:38251759:+	skipped	ENSG00000165046	8:38243820:38251952:+	LETM2
8:48947426:48947621:+	skipped	ENSG00000169139	8:48920959:48977268:+	UBE2V2
8:59472843:59474354:+	skipped	ENSG00000137575	8:59465482:59484860:+	SDCBP
8:67929854:67930066:-	skipped	ENSG00000178125	8:67900365:67940786:-	PPP1R42
8:74881795:74881908:-	skipped	ENSG00000154582	8:74851403:74884421:-	TCEB1
8:77900543:77900574:-	skipped	ENSG00000164751	8:77892493:77913280:-	PEX2
8:82597998:82598198:-	skipped	ENSG00000133731	8:82583173:82598553:-	IMPA1
8:93933731:93933888:-	skipped	ENSG00000253197	8:93895211:93977873:+	CTD-3239E11.2
8:95503806:95504137:-	skipped	ENSG00000164944	8:95503965:95508664:-	KIAA1429
8:128951754:128951922:+	skipped	ENSG00000249859	8:128806802:128952700:+	PVT1
8:144674885:144675063:-	skipped	ENSG00000104529	8:144671350:144679612:-	EEF1D
8:144902836:144902886:-	skipped	ENSG00000179950	8:144900119:144911214:-	PUF60
8:146002843:146002962:-	skipped	ENSG00000196378	8:145998498:146012710:-	ZNF34
8:146054812:146054989:+	skipped	ENSG00000147789	8:146052961:146055362:+	ZNF7
8:146054908:146054989:+	skipped	ENSG00000147789	8:146052961:146055362:+	ZNF7
9:3301546:3301620:-	skipped	ENSG00000080298	9:3218296:3525983:-	RFX3
9:33928370:33928610:-	skipped	ENSG00000137073	9:33921856:33928833:-	UBAP2
9:70858099:70858196:+	skipped	ENSG00000196873	9:70856860:70862843:+	CBWD3
9:72001475:72002599:+	skipped	ENSG00000135063	9:72000735:72006629:+	FAM189A2
9:80880775:80880822:+	skipped	ENSG00000148019	9:80878965:80886799:+	CEP78
9:90584711:90584834:-	skipped	ENSG00000156345	9:90581355:90589600:-	CDK20
9:97218522:97218619:+	skipped	ENSG00000148110	9:97136832:97223324:+	HIATL1
9:97767440:97767502:+	skipped	ENSG00000148120	9:97718227:97768482:+	C9orf3
9:100872351:100872516:-	skipped	ENSG00000106785	9:100831556:100881492:-	TRIM14

Table S1. Exons affected by SWI/SNF ATPase subunits

BRM-wt

Affected exon coordinates	included/skipped	ENSEMBL gene ID	gene coordinates	Gene name
9:114814679:114814718:-	skipped	ENSG00000106868	9:114803064:114937465:-	SUSD1
9:115380151:115380234:+	skipped	ENSG00000165185	9:115249126:115431677:+	KIAA1958
9:123842584:123842756:+	skipped	ENSG00000119397	9:123837140:123880849:+	CNTRL
9:128031215:128031263:+	skipped	ENSG00000165219	9:128024233:128031333:+	GAPVD1
9:128268589:128268696:-	skipped	ENSG00000119487	9:128201091:128268645:-	MAPKAP1
9:129812322:129812411:+	skipped	ENSG00000136828	9:129812334:129842132:+	RALGPS1
9:131586026:131586173:-	skipped	ENSG00000198917	9:131581929:131592100:-	C9orf114
9:132394929:132395144:+	skipped	ENSG00000148335	9:132388451:132398209:+	NTMT1
9:134349841:134351922:+	skipped	ENSG00000130723	9:134348980:134350234:+	PRRC2B
9:136905156:136905391:-	skipped	ENSG00000169925	9:136895426:136933139:-	BRD3
X:2795241:2795348:+	skipped	ENSG00000056998	X:2761038:2799254:+	GYG2
X:13769368:13769487:+	skipped	ENSG00000046651	X:13752831:13787480:+	OFD1
X:17762191:17762339:+	skipped	ENSG00000047634	X:17761988:17773105:+	SCML1
X:44919267:44919401:+	skipped	ENSG00000147050	X:44918526:44922921:+	KDM6A
X:47081660:47081779:+	skipped	ENSG00000102225	X:47077258:47081779:+	CDK16
X:73749048:73749276:+	skipped	ENSG00000147100	X:73740954:73752443:+	SLC16A2
X:100306633:100306722:-	skipped	ENSG00000188917	X:100278574:100307071:-	TRMT2B
X:102840787:102841219:+	skipped	ENSG00000133142	X:102840432:102841427:+	TCEAL4
X:102841147:102841219:+	skipped	ENSG00000133142	X:102840432:102841427:+	TCEAL4
X:102939609:102939657:-	skipped	ENSG00000123562	X:102931843:102940433:-	MORF4L2
X:123155217:123155281:+	skipped	ENSG00000101972	X:123094061:123164898:+	STAG2
X:129312967:129314017:+	skipped	ENSG00000134594	X:129305622:129318844:+	RAB33A
X:129917521:129917664:-	skipped	ENSG00000165675	X:129757349:130037208:-	ENOX2
X:135290614:135290800:+	skipped	ENSG00000022267	X:135286879:135292113:+	FHL1
X:151883336:151883646:+	skipped	ENSG00000183305	X:151883081:151885563:+	MAGEA2B
X:151883568:151883646:+	skipped	ENSG00000183305	X:151883081:151885563:+	MAGEA2B
X:153277980:153278129:-	skipped	ENSG00000184216	X:153275950:153285431:-	IRAK1
X:153631283:153631531:-	skipped	ENSG00000147403	X:153627706:153632038:+	RPL10
1:17259:17368:-	included	ENSG00000227232	1:14362:29370:-	WASH7P
1:1206890:1207017:-	included	ENSG00000160087	1:1198232:1209217:-	UBE2J2
1:1255836:1256473:-	included	ENSG00000127054	1:1246976:1260071:-	CPSF3L
1:1622415:1622832:-	included	ENSG00000189339	1:1592938:1624083:-	SLC35E2B
1:1643703:1643866:-	included	ENSG00000008128	1:1634168:1654270:-	CDK11A
1:2076777:2076950:+	included	ENSG00000067606	1:2065076:2080363:+	PRKCZ
1:6946300:6946406:+	included	ENSG00000171735	1:6845523:6948261:+	CAMTA1
1:19669246:19669358:-	included	ENSG00000077549	1:19665272:19746245:-	CAPZB
1:22408215:22408287:+	included	ENSG00000070831	1:22379789:22413283:+	CDC42
1:23435515:23435620:-	included	ENSG00000169641	1:23410515:23435623:-	LUZP1
1:23436078:23436143:-	included	ENSG00000169641	1:23420647:23460172:-	LUZP1
1:26152793:26152902:+	included	ENSG00000117640	1:26149486:26156288:+	MTFR1L
1:28843237:28843365:+	included	ENSG00000180198	1:28836588:28862538:+	RCC1
1:28857035:28857085:+	included	ENSG00000180198	1:28836588:28862538:+	RCC1
1:32100823:32101123:-	included	ENSG00000162517	1:32096389:32110190:-	PEF1
1:33486968:33487062:-	included	ENSG00000004455	1:33479965:33487291:-	AK2
1:33791396:33791562:-	included	ENSG00000225313	1:33772966:33791499:+	RP11-415J8.3
1:38262416:38262492:+	included	ENSG00000185090	1:38259473:38266805:+	MANEAL
1:40029508:40029594:-	included	ENSG00000090621	1:40026487:40042423:-	PABPC4
1:40211047:40214439:+	included	ENSG00000084072	1:40157853:40211075:+	PPIE
1:40919875:40919960:+	included	ENSG00000187801	1:40915773:40929386:+	ZFP69B
1:44297327:44297466:+	included	ENSG00000126091	1:44171494:44303918:+	ST3GAL3
1:44436578:44436861:+	included	ENSG00000132768	1:44435671:44437198:+	DPH2
1:44436638:44436892:+	included	ENSG00000132768	1:44435671:44437198:+	DPH2

Table S1. Exons affected by SWI/SNF ATPase subunits

BRM-wt

Affected exon coordinates	included/skipped	ENSEMBL gene ID	gene coordinates	Gene name
1:44441337:44441520:+	included	ENSG00000117410	1:44440158:44442495:+	ATP6V0B
1:46156646:46156782:+	included	ENSG00000159596	1:46153867:46159517:+	TMEM69
1:68947729:68948580:-	included	ENSG00000233589	1:68944811:68949222:+	RP4-694A7.2
1:70831829:70832011:+	included	ENSG00000197568	1:70820487:70833594:+	HHLA3
1:78390873:78390914:+	included	ENSG00000162614	1:78383812:78409580:+	NEXN
1:85136872:85136991:-	included	ENSG00000117155	1:85109389:85156228:-	SSX2IP
1:89453935:89454034:-	included	ENSG00000137944	1:89401455:89458459:-	CCBL2
1:93676359:93676483:+	included	ENSG00000122483	1:93646325:93682236:+	CCDC18
1:109504931:109505091:-	included	ENSG00000121940	1:109472129:109506111:-	CLCC1
1:110020440:110020631:+	included	ENSG00000143028	1:110009179:110022410:+	SYPL2
1:113033633:113033703:+	included	ENSG00000134245	1:113009162:113063905:+	WNT2B
1:113247675:113247790:-	included	ENSG00000155366	1:113247261:113249749:-	RHOC
1:113247722:113248874:-	included	ENSG00000155366	1:113247261:113249749:-	RHOC
1:114267381:114267515:-	included	ENSG00000116793	1:114239823:114301777:-	PHTF1
1:144619347:144619419:+	included	ENSG00000225241	1:144614531:144619419:+	RP11-640M9.2
1:146057587:146057734:-	included	ENSG00000152042	1:146032646:146082765:-	NBPF11
1:147597182:147597284:-	included	ENSG00000203836	1:147596183:147615861:-	NBPF24
1:147599424:147599571:-	included	ENSG00000203836	1:147596183:147615861:-	NBPF24
1:148344640:148344742:-	included	ENSG00000203832	1:148341795:148346929:-	NBPF20
1:151297221:151297393:-	included	ENSG00000143393	1:151264272:151300191:-	PI4KB
1:155224191:155224247:-	included	ENSG00000160767	1:155222539:155225274:-	FAM189B
1:155292034:155292228:-	included	ENSG00000225855	1:155287736:155293959:-	RUSC1-AS1
1:155746186:155746272:-	included	ENSG00000116580	1:155736326:155746252:-	GON4L
1:156177653:156177804:+	included	ENSG00000160785	1:156163879:156182587:+	SLC25A44
1:156450435:156450569:-	included	ENSG00000116604	1:156433518:156470620:-	MEF2D
1:183775509:183775619:+	included	ENSG00000143344	1:183605219:183897665:+	RGL1
1:192989437:192989586:-	included	ENSG00000116750	1:192987751:192998439:-	UCHL5
1:198190129:198190240:+	included	ENSG00000151414	1:198189928:198231753:+	NEK7
1:202923369:202923435:-	included	ENSG00000159346	1:202909950:202927517:-	ADIPOR1
1:204971724:204971876:+	included	ENSG00000163531	1:204889759:204991950:+	NFASC
1:205633613:205634014:-	included	ENSG00000158715	1:205626978:205649587:-	SLC45A3
1:213061233:213061343:+	included	ENSG00000162769	1:213031596:213072705:+	FLVCR1
1:225976736:225976843:+	included	ENSG00000143742	1:225965530:225978168:+	SRP9
1:226035911:226036085:-	included	ENSG00000196187	1:226034839:226036689:-	TMEM63A
1:233248545:233248712:-	included	ENSG00000135749	1:233225596:233270934:-	PCNXL2
1:235377084:235377341:-	included	ENSG00000054267	1:235341114:235377231:-	ARID4B
10:286833:286910:+	included	ENSG00000015171	10:285901:292939:+	ZMYND11
10:11308561:11308640:+	included	ENSG00000048740	10:11047258:11378666:+	CELF2
10:13150138:13150289:+	included	ENSG00000123240	10:13142158:13151662:+	OPTN
10:14595321:14595386:-	included	ENSG00000065809	10:14561157:14646388:-	FAM107B
10:28884662:28884970:+	included	ENSG00000095787	10:28884855:28897522:+	WAC
10:31661947:31662102:+	included	ENSG00000148516	10:31608140:31676198:+	ZEB1
10:32561942:32562010:-	included	ENSG00000120616	10:32560641:32562145:-	EPC1
10:35378824:35378929:-	included	ENSG00000108094	10:35297478:35379570:-	CUL2
10:35426710:35426807:+	included	ENSG00000095794	10:35415718:35501053:+	CREM
10:35490379:35490414:+	included	ENSG00000095794	10:35415718:35501053:+	CREM
10:45958599:45959444:-	included	ENSG00000165406	10:45950034:46030842:-	MARCH8
10:46923604:46923810:+	included	ENSG00000165874	10:46914471:46923688:+	FAM35BP
10:51245749:51245817:-	included	ENSG00000174194	10:51224687:51246535:-	AGAP8
10:51729802:51730218:+	included	ENSG00000214982	10:51623416:51732824:+	PARGP1
10:81070681:81070941:+	included	ENSG00000108175	10:81065974:81072506:+	ZMIZ1
10:98714710:98715591:+	included	ENSG00000196233	10:98708898:98740800:+	LCOR

Table S1. Exons affected by SWI/SNF ATPase subunits

BRM-wt

Affected exon coordinates	included/skipped	ENSEMBL gene ID	gene coordinates	Gene name
10:103345619:103345913:-	included	ENSG00000166169	10:103338638:103347966:-	POLL
10:105153956:105154151:-	included	ENSG00000221767	10:105154009:105154158:-	MIR1307
10:111765722:111765909:+	included	ENSG00000148700	10:111756125:111878366:+	ADD3
10:114208140:114208247:+	included	ENSG00000151532	10:114206755:114210484:+	VTI1A
10:121339983:121340050:-	included	ENSG00000151923	10:121334198:121356007:-	TIAL1
11:1316875:1317024:-	included	ENSG00000078902	11:1311324:1330820:-	TOLLIP
11:6431857:6432591:-	included	ENSG00000166313	11:6424548:6440319:-	APBB1
11:6501553:6501583:-	included	ENSG00000132254	11:6496909:6502542:-	ARFIP2
11:8751497:8752756:-	included	ENSG00000166444	11:8752421:8778699:-	ST5
11:12270731:12270793:+	included	ENSG00000133816	11:12229271:12285332:+	MICAL2
11:18314446:18314523:-	included	ENSG00000110756	11:18300222:18343721:-	HPS5
11:27696844:27697687:+	included	ENSG00000176697	11:27676439:27723180:-	BDNF
11:31823419:31823460:-	included	ENSG00000007372	11:31822254:31825573:-	PAX6
11:43391629:43391935:+	included	ENSG00000052841	11:43380481:43468269:+	TTC17
11:47434951:47435058:+	included	ENSG00000165915	11:47433852:47435431:+	SLC39A13
11:58331628:58331674:-	included	ENSG00000110031	11:58294343:58345639:-	LPXN
11:61103676:61103862:+	included	ENSG00000149476	11:61100692:61106617:+	DAK
11:63714197:63714475:+	included	ENSG00000110583	11:63706430:63724791:+	NAA40
11:64039085:64039275:-	included	ENSG00000002330	11:64037740:64039340:-	BAD
11:64787891:64787971:+	included	ENSG00000213465	11:64786053:64788019:+	ARL2
11:65480819:65480974:+	included	ENSG00000172977	11:65479709:65486835:+	KAT5
11:67256738:67256926:+	included	ENSG00000110711	11:67256806:67258124:+	AIP
11:68673537:68673852:+	included	ENSG00000132740	11:68671358:68678947:+	IGHMBP2
11:76165793:76165834:+	included	ENSG00000158636	11:76155966:76208275:+	C11orf30
11:85372681:85372793:-	included	ENSG00000137504	11:85370751:85376146:-	CREBZF
11:102080248:102080295:+	included	ENSG00000137693	11:101981191:102104154:+	YAP1
11:112100931:112100953:+	included	ENSG00000150787	11:112100816:112104121:+	PTS
11:113688380:113688559:-	included	ENSG00000048028	11:113668595:113746292:-	USP28
11:118274639:118274700:+	included	ENSG00000167283	11:118271868:118280560:+	ATP5L
11:118428453:118428605:-	included	ENSG00000118096	11:118427708:118436726:-	IFT46
11:120197831:120198349:+	included	ENSG00000181264	11:120195837:120204388:+	TMEM136
11:124980540:124980642:-	included	ENSG00000150433	11:124966440:124981604:-	TMEM218
11:126076149:126076448:-	included	ENSG00000165526	11:126075361:126081587:-	RPUSD4
11:126158338:126158468:+	included	ENSG00000150455	11:126152959:126161464:+	TIRAP
11:126159560:126159728:+	included	ENSG00000150455	11:126152959:126161464:+	TIRAP
12:86496:86722:+	included	ENSG00000226210	12:73724:91218:+	ABC7-42389800N19.1
12:6959997:6960173:-	included	ENSG00000111665	12:6953956:6960433:-	CDCA3
12:7021894:7022191:+	included	ENSG00000010626	12:7013896:7023407:+	LRRC23
12:7075074:7075079:-	included	ENSG00000215021	12:7074489:7076738:-	PHB2
12:8801083:8801151:-	included	ENSG00000197614	12:8800697:8811174:-	MFAP5
12:9072352:9072513:+	included	ENSG00000111752	12:9066491:9075287:+	PHC1
12:9845424:9846544:+	included	ENSG00000069493	12:9833520:9847725:+	CLEC2D
12:10862507:10862713:-	included	ENSG00000060138	12:10851682:10875906:-	YBX3
12:12841447:12841866:-	included	ENSG00000183150	12:12813994:12849121:-	GPR19
12:27829997:27830029:+	included	ENSG00000110841	12:27677105:27844717:+	PPFIBP1
12:30872013:30872159:-	included	ENSG00000110888	12:30862485:30906593:-	CAPRIN2
12:31237903:31238060:+	included	ENSG00000013573	12:31231453:31242064:+	DDX11
12:50052225:50052377:-	included	ENSG00000161791	12:50031723:50101197:-	FMNL3
12:50526745:50527851:-	included	ENSG00000139624	12:50523640:50526948:-	CERS5
12:50796458:50796596:+	included	ENSG00000161813	12:50786165:50824334:+	LARP4
12:53621146:53621471:-	included	ENSG00000172819	12:53621157:53625998:-	RARG
12:54675579:54675725:+	included	ENSG00000135486	12:54673976:54680871:+	HNRNPA1

Table S1. Exons affected by SWI/SNF ATPase subunits

BRM-wt

Affected exon coordinates	included/skipped	ENSEMBL gene ID	gene coordinates	Gene name
12:54676863:54677018:+	included	ENSG00000135486	12:54673976:54680871:+	HNRNPA1
12:56223273:56223420:-	included	ENSG00000135392	12:56214743:56224565:-	DNAJC14
12:56231339:56231466:-	included	ENSG00000123342	12:56230209:56233818:-	MMP19
12:56320860:56321051:-	included	ENSG00000170473	12:56295196:56321697:-	WIBG
12:56329089:56329188:+	included	ENSG00000065357	12:56321102:56331227:+	DGKA
12:56396327:56396504:+	included	ENSG00000139531	12:56395281:56399309:+	SUOX
12:56510973:56510995:+	included	ENSG00000229117	12:56510369:56511727:+	RPL41
12:56558087:56558152:-	included	ENSG00000258199	12:56556142:56584068:-	RP11-977G19.5
12:56685415:56685577:-	included	ENSG00000062485	12:56679974:56685542:-	CS
12:57882802:57883128:+	included	ENSG00000166986	12:57881838:57884125:+	MARS
12:58089326:58089460:+	included	ENSG00000135506	12:58087914:58090061:+	OS9
12:58197307:58197452:-	included	ENSG00000135407	12:58197078:58200272:-	AVIL
12:69279572:69279665:-	included	ENSG00000135678	12:69244954:69326979:-	CPM
12:108961434:108961533:+	included	ENSG00000136003	12:108956357:108963117:+	ISCU
12:109895797:109895989:-	included	ENSG00000110906	12:109887765:109896790:-	KCTD10
12:109907320:109909182:-	included	ENSG00000110906	12:109906866:109915105:-	KCTD10
12:111953958:111954167:-	included	ENSG00000204842	12:111947722:111958749:-	ATXN2
12:117287119:117287289:+	included	ENSG00000135119	12:117273801:117290750:+	RNFT2
12:120512247:120512390:+	included	ENSG00000135127	12:120502440:120531451:+	CCDC64
12:120529051:120529205:+	included	ENSG00000135127	12:120528558:120531145:+	CCDC64
12:120636357:120636434:-	included	ENSG00000089157	12:120636160:120638635:-	RPLP0
12:121465379:121465620:-	included	ENSG00000135114	12:121458094:121477045:-	OASL
12:121840545:121840610:+	included	ENSG00000170633	12:121837843:121862154:+	RNF34
12:121870821:121871010:-	included	ENSG00000089094	12:121866899:122018364:-	KDM2B
12:122616839:122616930:+	included	ENSG00000175727	12:122612434:122618711:+	MLXIP
12:122831922:122832026:-	included	ENSG00000130779	12:122817531:122879974:-	CLIP1
12:123010717:123010840:-	included	ENSG00000111011	12:122989189:123011546:-	RSRC2
12:123462528:123462746:+	included	ENSG00000111325	12:123459126:123464589:+	OGFOD2
12:123687797:123687922:-	included	ENSG00000051825	12:123637079:123706441:-	MPHOSPH9
13:37628272:37628433:-	included	ENSG00000102710	13:37625678:37633605:-	SUPT20H
13:42795400:42795530:+	included	ENSG00000102780	13:42614175:42803294:+	DGKH
13:111296412:111296529:-	included	ENSG00000134905	13:111293758:111316208:-	CARS2
14:19919492:19919629:-	included	ENSG00000244306	14:19918475:19919581:-	CTD-2314B22.3
14:21161706:21161809:+	included	ENSG00000214274	14:21152335:21162345:+	ANG
14:21679565:21679722:-	included	ENSG00000092199	14:21679095:21680161:-	HNRNPC
14:21969055:21969271:-	included	ENSG00000165819	14:21966276:21971972:-	METTL3
14:23417077:23417222:-	included	ENSG00000092036	14:23415436:23426363:-	HAUS4
14:23521179:23521292:-	included	ENSG00000139880	14:23516270:23526747:-	CDH24
14:24470072:24470293:+	included	ENSG00000187630	14:24464305:24475157:+	DHRS4L2
14:24775153:24775343:-	included	ENSG00000136305	14:24774301:24777469:-	CIDEB
14:24911384:24911466:-	included	ENSG00000100445	14:24908971:24911982:-	SDR39U1
14:35777200:35777294:+	included	ENSG00000100902	14:35761593:35780258:+	PSMA6
14:45579097:45579196:+	included	ENSG00000185246	14:45566108:45579379:+	PRPF39
14:52470958:52470960:+	included	ENSG00000087302	14:52470729:52471410:+	C14orf166
14:58755436:58755531:-	included	ENSG00000268466	14:58754750:58755865:+	AL132989.1
14:62212409:62212535:+	included	ENSG00000100644	14:62212322:62214190:+	HIF1A
14:64682004:64682072:+	included	ENSG00000054654	14:64628831:64693150:+	SYNE2
14:65544046:65544146:-	included	ENSG00000125952	14:65541841:65569222:-	MAX
14:68129746:68129907:-	included	ENSG00000100568	14:68113791:68141548:-	VTI1B
14:69345175:69345240:-	included	ENSG00000072110	14:69341039:69345739:-	ACTN1
14:72043252:72043304:+	included	ENSG00000197555	14:71787165:72054482:+	SIPA1L1
14:74345799:74346008:+	included	ENSG00000140043	14:74318546:74352159:+	PTGR2

Table S1. Exons affected by SWI/SNF ATPase subunits

BRM-wt

Affected exon coordinates	included/skipped	ENSEMBL gene ID	gene coordinates	Gene name
14:93172828:93172998:-	included	ENSG00000100600	14:93170166:93183804:-	LGMN
14:100841620:100841883:-	included	ENSG00000140105	14:100828039:100841810:-	WARS
14:103958114:103958371:+	included	ENSG00000075413	14:103957875:103969408:+	MARK3
14:103966493:103966537:+	included	ENSG00000075413	14:103957875:103969408:+	MARK3
15:23200631:23200789:-	included	ENSG00000187667	15:23187727:23208417:-	WHAMMP3
15:23285446:23285600:-	included	ENSG00000140181	15:23285219:23293417:-	HERC2P2
15:25653767:25653831:-	included	ENSG00000114062	15:25582380:25684128:-	UBE3A
15:32816672:32816801:-	included	ENSG00000223509	15:32812048:32819092:-	RP11-632K20.7
15:42453672:42453788:-	included	ENSG00000166887	15:42452628:42454250:-	VPS39
15:42820460:42820618:+	included	ENSG00000092531	15:42820140:42823849:+	SNAP23
15:43713223:43713372:-	included	ENSG00000067369	15:43699411:43748529:-	TP53BP1
15:49912331:49912387:-	included	ENSG00000166262	15:49906988:49913013:-	FAM227B
15:69652305:69652470:+	included	ENSG00000137819	15:69606741:69696843:+	PAQR5
15:70969447:70969479:-	included	ENSG00000137831	15:70949140:70994647:-	UACA
15:75134876:75135015:-	included	ENSG00000140474	15:75128456:75135538:-	ULK3
15:75308934:75309090:+	included	ENSG00000198794	15:75303998:75310808:+	SCAMP5
15:82769402:82769439:-	included	ENSG00000255769	15:82768638:82770067:-	RP11-152F13.3
15:93426815:93427008:+	included	ENSG00000272888	15:93425961:93427091:+	AC013394.2
15:101833498:101833598:-	included	ENSG00000131876	15:101826814:101835418:-	SNRPA1
16:732953:733087:-	included	ENSG00000161999	16:731670:734528:-	JMJD8
16:841851:841948:+	included	ENSG00000127586	16:838045:848072:+	CHTF18
16:1876508:1876603:-	included	ENSG00000063854	16:1868515:1876803:-	HAGH
16:2719412:2722571:-	included	ENSG00000260565	16:2722326:2723445:-	ERVK13-1
16:2825452:2825626:-	included	ENSG00000103363	16:2821414:2827298:-	TCEB2
16:3338454:3338570:+	included	ENSG00000006194	16:3332942:3341460:+	ZNF263
16:4504812:4504928:+	included	ENSG00000103423	16:4475805:4506776:+	DNAJA3
16:4851268:4851322:-	included	ENSG00000067836	16:4851029:4852881:-	ROGDI
16:5141795:5141896:-	included	ENSG00000118894	16:5134304:5147789:-	FAM86A
16:18438678:18438772:-	included	ENSG00000205746	16:18437996:18440175:-	RP11-1212A22.1
16:22530459:22530735:+	included	ENSG00000243716	16:22516171:22538951:+	NPIPB5
16:24950685:24950918:-	included	ENSG00000140750	16:24930705:25026652:-	ARHGAP17
16:28502803:28502881:-	included	ENSG00000188603	16:28493470:28503180:-	CLN3
16:29095764:29095853:+	included	ENSG00000260908	16:29065052:29118766:-	CTB-134H23.3
16:29820861:29821085:+	included	ENSG00000103495	16:29819095:29822484:+	MAZ
16:30900035:30900279:+	included	ENSG00000099385	16:30844946:30905623:-	BCL7C
16:32776759:32776866:+	included	ENSG00000260644	16:32772263:32782919:+	HERC2P5
16:57209451:57209502:-	included	ENSG00000172775	16:57186377:57219976:-	FAM192A
16:67201206:67201305:+	included	ENSG00000102878	16:67197287:67203848:+	HSF4
16:67296115:67296192:+	included	ENSG00000262691	16:67295010:67297687:+	CTC-277H1.7
16:67858486:67858682:+	included	ENSG00000102904	16:67840667:67861917:+	TSNAXIP1
16:71322781:71322840:-	included	ENSG00000180917	16:71319552:71323330:-	CMTR2
16:75129496:75129648:+	included	ENSG00000186187	16:75129041:75139234:+	ZNRF1
16:84213552:84213689:-	included	ENSG00000103168	16:84211457:84220655:-	TAF1C
16:89627635:89627737:+	included	ENSG00000167526	16:89627064:89630949:+	RPL13
16:90057637:90057791:+	included	ENSG00000223959	16:90046652:90063031:+	AFG3L1P
17:65445:65593:-	included	ENSG00000181031	17:63114:65724:-	RPH3AL
17:653054:653149:-	included	ENSG00000179409	17:651083:655278:-	GEMIN4
17:708308:708487:-	included	ENSG00000167693	17:702552:767351:-	NXN
17:5329291:5331531:+	included	ENSG00000263272	17:5328458:5336196:-	CTC-524C5.2
17:5391516:5391909:+	included	ENSG00000167842	17:5389604:5392557:+	MIS12
17:7095212:7095321:-	included	ENSG00000132535	17:7093208:7123021:-	DLG4
17:7160176:7160357:+	included	ENSG00000262302	17:7150147:7165408:-	RP1-4G17.5

Table S1. Exons affected by SWI/SNF ATPase subunits

BRM-wt

Affected exon coordinates	included/skipped	ENSEMBL gene ID	gene coordinates	Gene name
17:8079277:8079344:-	included	ENSG00000179029	17:8077316:8079706:-	TMEM107
17:15457079:15457143:-	included	ENSG00000259024	17:15339337:15466875:-	TVP23C-CDRT4
17:15586168:15586278:-	included	ENSG00000221926	17:15584401:15587613:-	TRIM16
17:16351159:16351274:-	included	ENSG00000175061	17:16342135:16373767:+	FAM211A-AS1
17:17716682:17716793:-	included	ENSG00000072310	17:17713712:17717083:-	SREBF1
17:17726832:17726921:-	included	ENSG00000072310	17:17722340:17726932:-	SREBF1
17:17786019:17786177:-	included	ENSG00000175662	17:17746827:17875724:-	TOM1L2
17:17787948:17788082:-	included	ENSG00000175662	17:17746827:17875724:-	TOM1L2
17:18770595:18770647:+	included	ENSG00000141127	17:18761408:18775941:+	PRPSAP2
17:19575034:19575269:+	included	ENSG00000072210	17:19551838:19580909:+	ALDH3A2
17:26659172:26659408:-	included	ENSG00000109083	17:26657216:26662481:-	IFT20
17:29253827:29253936:+	included	ENSG00000184060	17:29248697:29285757:+	ADAP2
17:30536369:30536464:+	included	ENSG00000126858	17:30529842:30538214:+	RHOT1
17:30538135:30538257:+	included	ENSG00000126858	17:30529842:30538214:+	RHOT1
17:36291899:36292527:+	included	ENSG00000185128	17:36283970:36294912:+	TBC1D3F
17:36339906:36340198:-	included	ENSG00000197681	17:36337710:36347030:-	TBC1D3
17:37855790:37855840:+	included	ENSG00000141736	17:37844371:37873910:+	ERBB2
17:43545575:43545959:-	included	ENSG00000225190	17:43517528:43565224:-	PLEKHM1
17:44707777:44707816:+	included	ENSG00000073969	17:44668034:44834830:+	NSF
17:45112410:45112492:-	included	ENSG00000263142	17:45094558:45131935:+	LRRC37A17P
17:48541887:48541960:+	included	ENSG00000136457	17:48541856:48542786:-	CHAD
17:48626647:48626848:+	included	ENSG00000006282	17:48624513:48626690:+	SPATA20
17:48628359:48628583:+	included	ENSG00000006282	17:48627760:48628469:+	SPATA20
17:49231586:49231805:+	included	ENSG00000239672	17:49230932:49233907:+	NME1
17:57208642:57208728:-	included	ENSG00000182628	17:57187311:57232630:-	SKA2
17:57971157:57971285:+	included	ENSG00000108443	17:57970446:58024822:+	RPS6KB1
17:59104227:59104271:+	included	ENSG00000141376	17:58755212:59470192:+	BCAS3
17:60601597:60601692:+	included	ENSG00000146872	17:60555426:60637395:+	TLK2
17:60631024:60631116:+	included	ENSG00000146872	17:60555426:60637395:+	TLK2
17:71208817:71208879:-	included	ENSG00000133193	17:71203491:71228510:-	FAM104A
17:72770005:72770194:-	included	ENSG00000109065	17:72766685:72772470:-	NAT9
17:73969706:73969866:-	included	ENSG00000161533	17:73937587:73975444:-	ACOX1
17:79084714:79084759:+	included	ENSG00000175866	17:79058894:79090024:+	BAIAP2
17:79847659:79847845:-	included	ENSG00000183684	17:79845712:79849462:-	ALYREF
17:79977517:79977570:-	included	ENSG00000169689	17:79976577:79981983:-	STRA13
17:80136903:80137065:-	included	ENSG00000176155	17:80111485:80143048:-	CCDC57
17:80616367:80616589:-	included	ENSG00000141542	17:80614943:80617465:-	RAB40B
18:2778167:2778237:+	included	ENSG00000101596	18:2655736:2805015:+	SMCHD1
18:3450320:3450503:+	included	ENSG00000177426	18:3411605:3457682:+	TGIF1
18:33620770:33620828:-	included	ENSG00000141425	18:33569786:33647539:-	RPRD1A
18:34267091:34267141:+	included	ENSG00000134775	18:33877676:34360018:+	FHOD3
18:34340571:34340745:+	included	ENSG00000134775	18:33877676:34360018:+	FHOD3
18:43819971:43820153:+	included	ENSG00000152242	18:43753518:43820018:+	C18orf25
18:45396846:45396935:-	included	ENSG00000175387	18:45357921:45457512:-	SMAD2
18:77746602:77746750:-	included	ENSG00000141759	18:77732866:77748593:-	TXNL4A
19:1433738:1433826:+	included	ENSG00000071626	19:1426347:1435680:+	DAZAP1
19:3456548:3456633:+	included	ENSG00000141905	19:3435043:3463253:+	NFIC
19:5901346:5901465:-	included	ENSG00000266941	19:5899905:5901472:+	AC104532.3
19:8495542:8495751:+	included	ENSG00000099785	19:8478153:8503888:+	MARCH2
19:10217499:10217681:+	included	ENSG00000130810	19:10216964:10221970:+	PPAN
19:11144443:11144541:+	included	ENSG00000127616	19:11141488:11152142:+	SMARCA4
19:14602468:14602558:-	included	ENSG00000123159	19:14588571:14606944:-	GIPC1

Table S1. Exons affected by SWI/SNF ATPase subunits

BRM-wt

Affected exon coordinates	included/skipped	ENSEMBL gene ID	gene coordinates	Gene name
19:19102149:19102362:-	included	ENSG00000064607	19:19101701:19102427:-	SUGP2
19:30199161:30199330:-	included	ENSG00000131943	19:30191720:30206161:-	C19orf12
19:35760706:35760906:+	included	ENSG00000105698	19:35760010:35761420:+	USF2
19:37957641:37957706:-	included	ENSG00000196437	19:37902056:37958339:-	ZNF569
19:39663558:39664016:+	included	ENSG00000130669	19:39616409:39664443:+	PAK4
19:40771129:40771258:-	included	ENSG00000105221	19:40736223:40791302:-	AKT2
19:41939177:41939339:-	included	ENSG00000105341	19:41937653:41942344:-	ATP5SL
19:45583165:45583287:+	included	ENSG00000142252	19:45582452:45594127:+	GEMIN7
19:45973887:45974207:+	included	ENSG00000125740	19:45973522:45975811:+	FOSB
19:46269608:46270413:-	included	ENSG00000177045	19:46268650:46272113:-	SIX5
19:48745797:48745901:-	included	ENSG00000105483	19:48706888:48753085:-	CARD8
19:49129148:49129619:+	included	ENSG00000063176	19:49122547:49133030:+	SPHK2
19:50166600:50166771:-	included	ENSG00000126456	19:50165631:50167780:-	IRF3
19:50167931:50168103:-	included	ENSG00000126456	19:50162828:50169132:-	IRF3
19:50430951:50431105:-	included	ENSG00000104951	19:50392910:50432796:-	IL4I1
19:55853583:55853748:+	included	ENSG00000133247	19:55853208:55858099:+	SUV420H2
19:57866639:57866779:+	included	ENSG00000131845	19:57862674:57871266:+	ZNF304
2:263564:263762:-	included	ENSG00000035115	2:218135:264032:-	SH3YL1
2:9560119:9560229:-	included	ENSG00000119185	2:9552397:9563216:-	ITGB1BP1
2:25016721:25016834:+	included	ENSG00000138092	2:25016004:25022663:+	CENPO
2:25642384:25642404:-	included	ENSG00000138101	2:25606551:25650885:-	DTNB
2:27997291:27997397:+	included	ENSG00000243147	2:27994583:28002608:+	MRPL33
2:32118311:32118390:-	included	ENSG00000162959	2:32117121:32235486:-	MEMO1
2:38976040:38976488:-	included	ENSG00000115875	2:38971858:38976706:-	SRSF7
2:58285487:58285623:+	included	ENSG00000028116	2:58274009:58312062:+	VRK2
2:58421364:58421408:-	included	ENSG00000115392	2:58386377:58468458:-	FANCL
2:65543868:65544017:-	included	ENSG00000198369	2:65537984:65659311:-	SPRED2
2:73198699:73198814:-	included	ENSG00000144040	2:73171731:73208246:-	SFXN5
2:73961655:73961718:-	included	ENSG00000144034	2:73957390:73964467:-	TPRKB
2:74433109:74433215:+	included	ENSG00000065911	2:74425688:74444692:+	MTHFD2
2:74692023:74692114:-	included	ENSG00000115275	2:74688183:74692537:-	MOGS
2:75907319:75907440:-	included	ENSG00000005436	2:75891544:75938018:-	GCFC2
2:87779742:87779942:+	included	ENSG00000222041	2:87769468:87780211:+	LINC00152
2:102477287:102477448:+	included	ENSG00000071054	2:102472438:102481465:+	MAP4K4
2:160284452:160284553:-	included	ENSG00000123636	2:160260882:160292064:-	BAZ2B
2:171938150:171938218:-	included	ENSG00000198586	2:171910226:171948320:-	TLK1
2:173427469:173427528:+	included	ENSG00000152256	2:173420100:173489823:+	PDK1
2:177134944:177135147:+	included	ENSG00000128654	2:177134122:177202753:+	MTX2
2:179095153:179095240:+	included	ENSG00000079156	2:179059215:179149765:+	OSBPL6
2:179988442:179988556:-	included	ENSG00000187231	2:179974048:179989194:-	SESTD1
2:182785324:182785377:+	included	ENSG00000138434	2:182783587:182794404:+	SSFA2
2:192265475:192265561:+	included	ENSG00000128641	2:192110001:192290115:+	MYO1B
2:197537069:197537161:+	included	ENSG00000144395	2:197504277:197597524:+	CCDC150
2:200709103:200709255:-	included	ENSG00000226124	2:200677234:200715866:-	FTCDNL1
2:201724848:201724938:-	included	ENSG00000013441	2:201717731:201729422:-	CLK1
2:203075458:203075529:-	included	ENSG00000116030	2:203070902:203103331:-	SUMO1
2:203777757:203777837:+	included	ENSG00000138380	2:203776967:203807547:+	CARF
2:208013706:208013924:-	included	ENSG00000118263	2:207938860:208030669:-	KLF7
2:210880928:210880981:+	included	ENSG00000197713	2:210867288:210886300:+	RPE
2:219524760:219524968:+	included	ENSG00000074582	2:219523486:219525940:+	BCS1L
2:219525030:219525205:+	included	ENSG00000074582	2:219523486:219525940:+	BCS1L
2:220081374:220081554:-	included	ENSG00000115657	2:220074489:220083712:-	ABCB6

Table S1. Exons affected by SWI/SNF ATPase subunits

BRM-wt

Affected exon coordinates	included/skipped	ENSEMBL gene ID	gene coordinates	Gene name
2:220164422:220164508:-	included	ENSG00000054356	2:220154344:220174295:-	PTPRN
2:223752548:223752606:+	included	ENSG00000123983	2:223725651:223809357:+	ACSL3
2:230725122:230725247:-	included	ENSG00000153827	2:230695747:230786668:-	TRIP12
2:236839409:236839567:+	included	ENSG00000157985	2:236792038:236945341:+	AGAP1
20:1355128:1355342:+	included	ENSG00000088832	20:1349621:1356247:-	FKBP1A
20:3095951:3096112:-	included	ENSG00000235958	20:3087558:3131513:+	UBOX5-AS1
20:3306231:3306406:-	included	ENSG00000088854	20:3229950:3388272:-	C20orf194
20:10393178:10394579:-	included	ENSG00000125863	20:10381656:10414870:-	MKKS
20:11898426:11898659:+	included	ENSG00000132640	20:11898564:11907243:+	BTBD3
20:18285652:18285822:+	included	ENSG00000125846	20:18285728:18296032:+	ZNF133
20:20003100:20003124:+	included	ENSG00000173418	20:19998444:20003299:+	NAA20
20:30155881:30156083:+	included	ENSG00000101294	20:30153859:30157055:+	HM13
20:33868457:33868632:-	included	ENSG00000242372	20:33866713:33872788:-	EIF6
20:33871979:33872064:-	included	ENSG00000242372	20:33866713:33872788:-	EIF6
20:34087878:34087998:+	included	ENSG00000126001	20:34085637:34090632:+	CEP250
20:43227257:43227531:+	included	ENSG00000168734	20:43160425:43247678:+	PKIG
20:45867502:45867882:-	included	ENSG00000101040	20:45837858:45984401:-	ZMYND8
20:48700666:48700791:-	included	ENSG00000244687	20:48697660:48732491:-	UBE2V1
20:50407872:50408891:-	included	ENSG00000101115	20:50408606:50409617:-	SALL4
20:60702641:60702757:+	included	ENSG00000149657	20:60697516:60710434:+	LSM14B
20:61908557:61908657:+	included	ENSG00000101199	20:61904176:61909905:+	ARFGAP1
21:40719173:40719218:-	included	ENSG00000205581	21:40714240:40721025:-	HMGN1
21:45175358:45175645:+	included	ENSG00000160209	21:45173463:45176043:+	PDXK
21:46604389:46604508:+	included	ENSG00000197381	21:46591556:46646475:+	ADARB1
21:46685937:46686142:-	included	ENSG00000186866	21:46685288:46686290:-	POFUT2
22:19808751:19808898:-	included	ENSG00000185838	22:19799817:19842366:-	GNB1L
22:22899232:22899506:-	included	ENSG00000185686	22:22890122:22901768:-	PRAME
22:24572079:24572243:+	included	ENSG00000099991	22:24571746:24573506:+	CABIN1
22:24939810:24940051:-	included	ENSG00000138867	22:24936499:24944000:-	GUCD1
22:29684595:29687588:+	included	ENSG00000182944	22:29663997:29696514:+	EWSR1
22:29706817:29706988:+	included	ENSG00000185340	22:29706268:29707097:+	GAS2L1
22:31062794:31062925:-	included	ENSG00000167065	22:31048037:31063830:-	DUSP18
22:31724773:31724910:-	included	ENSG00000100105	22:31721789:31742218:-	PATZ1
22:35797659:35797807:+	included	ENSG00000100297	22:35796055:35821423:+	MCM5
22:38609830:38609896:+	included	ENSG00000185022	22:38609541:38612512:+	MAFF
22:41609634:41610707:-	included	ENSG00000100395	22:41601305:41613177:+	L3MBTL2
22:42078360:42078591:-	included	ENSG00000100138	22:42076247:42078460:-	NHP2L1
22:44223394:44223520:-	included	ENSG00000130540	22:44220388:44258383:-	SULT4A1
22:50181225:50181535:-	included	ENSG00000100425	22:50166930:50216861:-	BRD1
3:9739395:9739550:+	included	ENSG00000163719	3:9710426:9744071:+	MTMR14
3:9845220:9845391:+	included	ENSG00000241553	3:9834778:9845898:+	ARPC4
3:14170355:14170585:+	included	ENSG00000170876	3:14166439:14185179:+	TMEM43
3:15252309:15252429:+	included	ENSG00000131375	3:15247658:15259036:+	CAPN7
3:17627845:17628049:-	included	ENSG00000131374	3:17198653:17782399:-	TBC1D5
3:17664140:17664257:-	included	ENSG00000131374	3:17198653:17782399:-	TBC1D5
3:28383003:28383087:-	included	ENSG00000163512	3:28380036:28389968:-	AZI2
3:42835649:42835791:-	included	ENSG00000181061	3:42798668:42846010:-	HIGD1A
3:48337609:48337648:-	included	ENSG00000172113	3:48334753:48342848:-	NME6
3:49154492:49154794:-	included	ENSG00000172046	3:49153013:49158251:-	USP19
3:52028789:52028873:-	included	ENSG00000162244	3:52027615:52029958:-	RPL29
3:52242099:52242263:+	included	ENSG00000023330	3:52240647:52248241:+	ALAS1
3:52456238:52456354:+	included	ENSG00000010318	3:52445212:52457656:+	PHF7

Table S1. Exons affected by SWI/SNF ATPase subunits

BRM-wt

Affected exon coordinates	included/skipped	ENSEMBL gene ID	gene coordinates	Gene name
3:52592265:52592429:-	included	ENSG00000168273	3:52570620:52613253:+	SMIM4
3:52783708:52783845:-	included	ENSG00000114904	3:52780165:52786086:-	NEK4
3:57041210:57041364:-	included	ENSG00000163947	3:56761445:57113336:-	ARHGEF3
3:98240497:98240562:-	included	ENSG00000080822	3:98239562:98241746:-	CLDND1
3:100289453:100293503:+	included	ENSG00000181458	3:100287665:100289758:+	TMEM45A
3:107768466:107768498:-	included	ENSG00000196776	3:107762144:107773586:-	CD47
3:113146034:113146186:-	included	ENSG00000243849	3:113122844:113152839:+	WDR52-AS1
3:129009522:129009647:+	included	ENSG00000183624	3:128997670:129023594:+	HMCES
3:129185733:129185909:+	included	ENSG00000163913	3:129168713:129198760:+	IFT122
3:137790485:137790677:-	included	ENSG00000158163	3:137780831:137834451:-	DZIP1L
3:141461486:141461749:+	included	ENSG00000114125	3:141457045:141466402:+	RNF7
3:146254327:146254352:-	included	ENSG00000188313	3:146232966:146262651:-	PLSCR1
3:167758574:167758657:-	included	ENSG00000173905	3:167726464:167813763:-	GOLIM4
3:170824972:170824995:-	included	ENSG00000154310	3:170779127:171178197:-	TNIK
3:182743543:182743592:-	included	ENSG00000078070	3:182733005:182817375:-	MCCC1
3:195612284:195612414:-	included	ENSG00000061938	3:195611715:195619472:-	TNK2
4:520828:521019:+	included	ENSG00000174227	4:515678:524287:+	PIGG
4:667701:667755:-	included	ENSG00000169020	4:666224:667910:-	ATP5I
4:1291744:1291903:+	included	ENSG00000090316	4:1291857:1293757:+	MAEA
4:1953726:1953958:+	included	ENSG00000109685	4:1953831:1957885:+	WHSC1
4:3949817:3949947:-	included	ENSG00000251669	4:3949225:3954903:-	FAM86EP
4:13574326:13574466:-	included	ENSG00000038219	4:13571409:13579825:-	BOD1L1
4:20709426:20709493:+	included	ENSG00000163138	4:20709424:20711459:+	PACRGL
4:27009605:27009628:+	included	ENSG00000109689	4:27004621:27024462:+	STIM2
4:38053520:38053681:+	included	ENSG00000065882	4:38051377:38056663:+	TBC1D1
4:39065192:39065417:+	included	ENSG00000109790	4:39063851:39122825:+	KLHL5
4:44716613:44716676:-	included	ENSG00000163281	4:44703884:44728587:-	GNPDA2
4:52714371:52714533:+	included	ENSG00000109184	4:52709165:52780460:+	DCUN1D4
4:77066708:77066844:-	included	ENSG00000138750	4:77055328:77069530:-	NUP54
4:77916934:77917091:+	included	ENSG00000138758	4:77870855:77959767:+	SEPT11
4:79843983:79844137:-	included	ENSG00000163291	4:79831991:79860379:-	PAQR3
4:88106403:88106951:-	included	ENSG00000145332	4:88081254:88141760:-	KLHL8
4:128843015:128843118:-	included	ENSG00000164073	4:128842511:128843144:-	MFSD8
4:128886227:128886637:-	included	ENSG00000164073	4:128851860:128887099:-	MFSD8
4:147177978:147178088:-	included	ENSG00000120519	4:147175126:147443116:-	SLC10A7
4:148984299:148984451:+	included	ENSG00000071205	4:148944204:148984428:+	ARHGAP10
4:152021637:152021740:+	included	ENSG00000145425	4:152020724:152025804:+	RPS3A
4:158282162:158282276:+	included	ENSG00000120251	4:158281851:158284086:+	GRIA2
4:159133213:159133372:+	included	ENSG00000164124	4:159122755:159136465:+	TMEM144
4:159754953:159755042:+	included	ENSG00000052795	4:159690345:159757810:+	FNIP2
4:169927036:169927065:-	included	ENSG00000145439	4:169922319:169931405:-	CBR4
4:183837439:183837692:-	included	ENSG00000129187	4:183836075:183838498:-	DCTD
4:185691409:185691486:-	included	ENSG00000151726	4:185689481:185695027:-	ACSL1
5:868722:869234:-	included	ENSG00000028310	5:868816:876318:-	BRD9
5:6631986:6632090:-	included	ENSG00000037474	5:6599351:6633404:-	NSUN2
5:38949458:38949529:-	included	ENSG00000164327	5:38938020:39074510:-	RICTOR
5:68551287:68551355:+	included	ENSG00000134058	5:68530667:68568731:+	CDK7
5:69206202:69206389:+	included	ENSG00000198237	5:69171097:69216694:+	RP11-98J23.2
5:69372348:69372401:+	included	ENSG00000205571	5:69345349:69373421:+	SMN2
5:74034327:74034467:-	included	ENSG00000164347	5:74017028:74063196:-	GFM2
5:92774409:92774447:-	included	ENSG00000237187	5:92746723:92774443:-	NR2F1-AS1
5:126113053:126113559:+	included	ENSG00000113368	5:126112314:126172706:+	LMNB1

Table S1. Exons affected by SWI/SNF ATPase subunits

BRM-wt

Affected exon coordinates	included/skipped	ENSEMBL gene ID	gene coordinates	Gene name
5:132335824:132335893:-	included	ENSG00000155329	5:132332676:132362296:-	ZCCHC10
5:133642300:133642393:-	included	ENSG00000006837	5:133541304:133706734:-	CDKL3
5:133912458:133912586:+	included	ENSG00000043143	5:133909446:133915165:+	JADE2
5:138941829:138941992:+	included	ENSG00000131508	5:138940750:139008016:+	UBE2D2
5:177577889:177577994:-	included	ENSG00000145912	5:177576460:177580968:-	NHP2
5:177658670:177658867:-	included	ENSG00000175309	5:177653466:177659542:-	PHYKPL
5:179035286:179035466:+	included	ENSG00000176783	5:179033903:179036473:+	RUFY1
5:179706796:179707036:-	included	ENSG00000050748	5:179660142:179719099:-	MAPK9
5:180666067:180666177:-	included	ENSG00000204628	5:180663908:180670916:-	GNB2L1
6:3004729:3004885:+	included	ENSG00000124588	6:3004400:3010423:+	NQO2
6:7246657:7247454:+	included	ENSG00000124782	6:7107829:7249507:+	RREB1
6:10751366:10751467:+	included	ENSG00000137210	6:10747991:10757214:+	TMEM14B
6:10755375:10755465:+	included	ENSG00000137210	6:10747991:10757214:+	TMEM14B
6:19804402:19804508:-	included	ENSG00000228412	6:19802394:19804981:-	RP4-625H18.2
6:26091542:26091817:+	included	ENSG00000010704	6:26087508:26095445:+	HFE
6:26463025:26463125:+	included	ENSG00000112763	6:26458149:26469830:+	BTN2A1
6:28239632:28240564:+	included	ENSG00000197062	6:28234787:28245974:+	RP5-874C20.3
6:30525927:30525989:+	included	ENSG00000204576	6:30524662:30531500:+	PRR3
6:30529105:30529285:+	included	ENSG00000204576	6:30524662:30531500:+	PRR3
6:31611859:31611971:-	included	ENSG00000204463	6:31606804:31620482:-	BAG6
6:31669851:31669907:-	included	ENSG00000204427	6:31654725:31671221:-	ABHD16A
6:31726310:31726397:+	included	ENSG00000204410	6:31721059:31727278:+	MSH5
6:31730567:31730945:+	included	ENSG00000255152	6:31727221:31732627:+	MSH5-SAPCD1
6:32122807:32122960:+	included	ENSG00000221988	6:32121217:32123755:+	PPT2
6:32942798:32942889:+	included	ENSG00000204256	6:32937095:32944395:+	BRD2
6:35261528:35261692:+	included	ENSG00000065029	6:35260099:35262329:+	ZNF76
6:36567598:36568053:+	included	ENSG00000112081	6:36562144:36568658:+	SRSF3
6:39876816:39876878:-	included	ENSG00000124615	6:39867353:39900171:-	MOCS1
6:44224422:44224615:-	included	ENSG00000157593	6:44221832:44225291:-	SLC35B2
6:46137790:46137851:-	included	ENSG00000112796	6:46126923:46138708:-	ENPP5
6:58276137:58276289:-	included	ENSG00000215190	6:58272360:58276677:-	LINC00680
6:75949993:75950101:-	included	ENSG00000112695	6:75947392:75953644:-	COX7A2
6:99365250:99365595:-	included	ENSG00000112234	6:99316419:99395802:-	FBXL4
6:109461624:109461878:+	included	ENSG00000183137	6:109450506:109485113:+	CEP57L1
6:109466422:109466584:+	included	ENSG00000183137	6:109450506:109485113:+	CEP57L1
6:135518099:135518461:+	included	ENSG00000118513	6:135516885:135518461:+	MYB
6:135818326:135818387:-	included	ENSG00000135541	6:135811054:135818787:-	AHI1
6:136597606:136597646:-	included	ENSG00000029363	6:136596692:136599241:-	BCLAF1
6:138763120:138763251:-	included	ENSG00000135540	6:138743179:138893677:-	NHSL1
6:139247538:139247618:-	included	ENSG00000135597	6:139224629:139309394:-	REPS1
6:157495981:157496139:+	included	ENSG00000049618	6:157469923:157502332:+	ARID1B
7:872142:872238:+	included	ENSG00000164828	7:870566:881672:+	SUN1
7:889560:889670:+	included	ENSG00000164828	7:889437:892544:+	SUN1
7:2376751:2376916:-	included	ENSG00000106266	7:2304074:2393944:-	SNX8
7:5028694:5028808:+	included	ENSG00000196204	7:5024538:5028999:+	RNF216P1
7:6052310:6052771:+	included	ENSG00000106305	7:6048920:6054882:+	AIMP2
7:12727260:12727353:+	included	ENSG00000122644	7:12726480:12728804:+	ARL4A
7:16725530:16725665:+	included	ENSG00000136261	7:16685755:16746148:+	BZW2
7:23561740:23562051:-	included	ENSG00000164548	7:23544398:23571660:-	TRA2A
7:23637586:23637670:+	included	ENSG00000169193	7:23637034:23641839:+	CCDC126
7:25219281:25219419:-	included	ENSG00000153790	7:25174315:25219973:-	C7orf31
7:30537357:30537512:-	included	ENSG00000006625	7:30536236:30544460:-	GGCT

Table S1. Exons affected by SWI/SNF ATPase subunits

BRM-wt

Affected exon coordinates	included/skipped	ENSEMBL gene ID	gene coordinates	Gene name
7:33392471:33392485:+	included	ENSG00000122507	7:33169143:33645680:+	BBS9
7:36456689:36456799:+	included	ENSG00000011426	7:36446159:36456757:+	ANLN
7:43919267:43919385:-	included	ENSG00000106608	7:43918542:43921495:-	URGCP
7:44799750:44799827:+	included	ENSG00000122515	7:44795777:44809477:+	ZMIZ2
7:64021324:64021651:-	included	ENSG00000173041	7:63980261:64023484:-	ZNF680
7:64291318:64291454:+	included	ENSG00000197008	7:64254765:64292580:+	ZNF138
7:72510219:72510331:+	included	ENSG00000174384	7:72507940:72515008:+	RP11-313P13.4
7:72952264:72952568:-	included	ENSG00000106635	7:72950685:72954898:-	BCL7B
7:73152206:73152472:-	included	ENSG00000106077	7:73150423:73153111:-	ABHD11
7:77567027:77567155:+	included	ENSG00000006576	7:77469446:77586818:+	PHTF2
7:91506192:91506292:-	included	ENSG00000127989	7:91506208:91509753:-	MTERF
7:96607659:96608903:-	included	ENSG00000231764	7:96607779:96641361:-	DLX6-AS1
7:99926459:99926642:-	included	ENSG00000078319	7:99918614:99939531:-	PMS2P1
7:102131433:102131570:-	included	ENSG00000170667	7:102122891:102133758:-	RASA4B
7:102230606:102230743:-	included	ENSG00000105808	7:102222796:102234438:-	RASA4
7:102732924:102733100:+	included	ENSG00000170632	7:102715327:102740205:+	ARMC10
7:106814893:106815190:+	included	ENSG00000105856	7:106809434:106822860:+	HBP1
7:106897177:106897239:-	included	ENSG00000164597	7:106876999:106899065:-	COG5
7:128681611:128681771:-	included	ENSG00000064419	7:128594947:128695198:-	TNPO3
7:149567331:149567440:-	included	ENSG00000204934	7:149564785:149570931:-	ATP6V0E2-AS1
7:155477627:155477746:+	included	ENSG00000184863	7:155465573:155538414:+	RBM33
8:21996152:21996306:-	included	ENSG00000168476	8:21995532:21999464:-	REEP4
8:22460050:22460156:+	included	ENSG00000241852	8:22457113:22461646:+	C8orf58
8:28906807:28906911:+	included	ENSG00000147421	8:28903816:28908171:+	HMBOX1
8:30942682:30942762:+	included	ENSG00000165392	8:30891316:31031285:+	WRN
8:33347803:33347857:+	included	ENSG00000198042	8:33342717:33356164:+	MAK16
8:38286765:38286908:-	included	ENSG00000077782	8:38282200:38287520:-	FGFR1
8:38287200:38287466:-	included	ENSG00000077782	8:38282200:38287520:-	FGFR1
8:38318614:38318773:-	included	ENSG00000077782	8:38314959:38320918:-	FGFR1
8:41442292:41442436:+	included	ENSG00000158669	8:41434705:41456133:+	AGPAT6
8:74872000:74872053:-	included	ENSG00000154582	8:74851403:74884421:-	TCEB1
8:90775057:90775210:+	included	ENSG00000104312	8:90770044:90775086:+	RIPK2
8:94752857:94752924:-	included	ENSG00000212998	8:94752348:94753001:+	RBM12B-AS1
8:101964157:101964536:-	included	ENSG00000164924	8:101960823:101964847:-	YWHAZ
8:136653839:136653969:+	included	ENSG00000131773	8:136605573:136659427:+	KHDRBS3
8:141958765:141958822:-	included	ENSG00000169398	8:141667998:142011332:-	PTK2
8:141993941:141994254:-	included	ENSG00000169398	8:141667998:142011332:-	PTK2
8:145735743:145735890:+	included	ENSG00000167700	8:145734456:145736596:+	MFSD3
8:146221893:146221993:-	included	ENSG00000196922	8:146198974:146228280:-	ZNF252P
9:177723:177820:-	included	ENSG00000172785	9:171952:179058:-	CBWD1
9:16435032:16435097:-	included	ENSG00000173068	9:16418540:16436370:-	BNC2
9:33956077:33956144:-	included	ENSG00000137073	9:33948379:33963780:-	UBAP2
9:35662509:35662738:-	included	ENSG00000137135	9:35658873:35665189:-	ARHGEF39
9:37763626:37763862:+	included	ENSG00000165275	9:37753813:37763754:+	TRMT10B
9:69243508:69243652:-	included	ENSG00000204790	9:69229611:69247581:-	CBWD6
9:70432923:70433098:-	included	ENSG00000147996	9:70432370:70433352:-	CBWD5
9:70470996:70471140:-	included	ENSG00000147996	9:70432003:70490246:-	CBWD5
9:71865951:71866280:+	included	ENSG00000119139	9:71736223:71870124:+	TJP2
9:79898271:79898387:+	included	ENSG00000197969	9:79894761:79934586:+	VPS13A
9:91033693:91033866:+	included	ENSG00000106723	9:91003333:91093609:+	SPIN1
9:94877204:94877348:-	included	ENSG00000090054	9:94870082:94877664:-	SPTLC1
9:95591220:95591445:+	included	ENSG00000187984	9:95584503:95592045:+	ANKRD19P

Table S1. Exons affected by SWI/SNF ATPase subunits

BRM-wt

Affected exon coordinates	included/skipped	ENSEMBL gene ID	gene coordinates	Gene name
9:103191788:103191944:+	included	ENSG00000066697	9:103189437:103213511:+	MSANTD3
9:108336479:108336613:+	included	ENSG00000106692	9:108320421:108359011:+	FKTN
9:116760539:116760634:+	included	ENSG00000157657	9:116638561:116812501:+	ZNF618
9:119459652:119459712:+	included	ENSG00000119401	9:119449580:119463577:+	TRIM32
9:128025830:128025979:+	included	ENSG00000165219	9:128024233:128031333:+	GAPVD1
9:134369792:134369873:+	included	ENSG00000130723	9:134366151:134375584:+	PRRC2B
9:139303451:139303519:-	included	ENSG00000165689	9:139301596:139305045:-	SDCCAG3
X:3740415:3740621:-	included	ENSG00000205664	X:3735568:3746991:-	RP11-706O15.1
X:13738547:13738616:-	included	ENSG00000196459	X:13733615:13740359:-	TRAPPC2
X:20068942:20069064:-	included	ENSG00000184368	X:20024830:20135016:-	MAP7D2
X:38013201:38013285:-	included	ENSG00000101955	X:38008588:38080177:-	SRPX
X:44965787:44965894:+	included	ENSG00000147050	X:44732756:44971847:+	KDM6A
X:47030427:47030657:+	included	ENSG00000182872	X:47004267:47046210:+	RBM10
X:48930092:48930309:-	included	ENSG00000243279	X:48928812:48931730:-	PRAF2
X:62736790:62736962:-	included	ENSG00000235437	X:62729821:62780929:-	RP11-357C3.3
X:102002761:102002839:+	included	ENSG00000198908	X:101975615:102003809:+	BHLHB9
X:102025280:102025348:+	included	ENSG00000223546	X:102024108:102094892:+	LINC00630
X:102071920:102072010:+	included	ENSG00000223546	X:102024108:102094892:+	LINC00630
X:102631956:102632034:+	included	ENSG00000166681	X:102631267:102633005:+	NGFRAP1
X:107370023:107370324:+	included	ENSG00000101844	X:107369306:107395088:+	ATG4A
X:129289131:129289261:-	included	ENSG00000156709	X:129263336:129299638:-	AIFM1
X:148608475:148608607:-	included	ENSG00000010404	X:148564073:148615470:-	IDS
X:151304534:151304552:-	included	ENSG00000266560	X:151283465:151306965:-	RP11-1007I13.4
X:151885137:151885234:+	included	ENSG00000183305	X:151883081:151885563:+	MAGEA2B

Table S1. Exons affected by SWI/SNF ATPase subunits

BRM-mut

**Affected exon coordinates**	**included/skipped**	**ENSEMBL gene ID**	**gene coordinates**	**Gene name**
1:16719723:16719977:+	skipped	ENSG00000055070	1:16679069:16719850:+	SZRD1
1:19452097:19452193:-	skipped	ENSG00000127481	1:19450970:19454195:-	UBR4
1:24857708:24857881:+	skipped	ENSG00000117602	1:24829386:24867530:+	RCAN3
1:25233750:25233908:-	skipped	ENSG00000020633	1:25226001:25291501:-	RUNX3
1:26153106:26153317:+	skipped	ENSG00000117640	1:26149486:26156288:+	MTFR1L
1:27736186:27736700:-	skipped	ENSG00000158195	1:27730729:27816669:-	WASF2
1:28167525:28167745:+	skipped	ENSG00000117751	1:28157288:28178187:+	PPP1R8
1:41618271:41618413:-	skipped	ENSG00000010803	1:41492871:41627104:-	SCMH1
1:46812593:46812747:+	skipped	ENSG00000117481	1:46805848:46827863:+	NSUN4
1:52526100:52526331:+	skipped	ENSG00000134717	1:52521796:52556388:+	BTF3L4
1:52924008:52924121:-	skipped	ENSG00000134744	1:52898760:52928584:-	ZCCHC11
1:54354917:54355136:-	skipped	ENSG00000058799	1:54317391:54355406:-	YIPF1
1:95705333:95705465:+	skipped	ENSG00000122481	1:95699710:95712781:+	RWDD3
1:109202486:109202584:-	skipped	ENSG00000162639	1:109190911:109203744:-	HENMT1
1:109635512:109635643:+	skipped	ENSG00000215717	1:109632424:109639556:+	TMEM167B
1:110172422:110172538:+	skipped	ENSG00000116337	1:110166435:110173839:+	AMPD2
1:113159435:113159517:-	skipped	ENSG00000007341	1:113149725:113161721:-	ST7L
1:113229588:113229728:+	skipped	ENSG00000155363	1:113217312:113229621:+	MOV10
1:114224834:114224924:+	skipped	ENSG00000081026	1:113933370:114225056:+	MAGI3
1:144619347:144619419:+	skipped	ENSG00000225241	1:144614531:144619419:+	RP11-640M9.2
1:146055345:146055447:-	skipped	ENSG00000152042	1:146032646:146082765:-	NBPF11
1:147607403:147607526:-	skipped	ENSG00000203836	1:147596183:147615861:-	NBPF24
1:149577608:149577662:+	skipped	ENSG00000269501	1:149576148:149577802:+	RP11-353N4.6
1:150598955:150599002:-	skipped	ENSG00000143420	1:150594916:150601616:-	ENSA
1:153934696:153934826:-	skipped	ENSG00000143570	1:153931574:153936048:-	SLC39A1
1:153963390:153963510:+	skipped	ENSG00000177954	1:153963234:153964626:+	RPS27
1:154241838:154241888:+	skipped	ENSG00000143569	1:154193324:154243328:+	UBAP2L
1:155110037:155110198:+	skipped	ENSG00000169241	1:155107819:155110844:+	SLC50A1
1:155644801:155644887:-	skipped	ENSG00000163374	1:155629236:155658548:-	YY1AP1
1:155740858:155740948:-	skipped	ENSG00000116580	1:155735239:155743211:-	GON4L
1:161136890:161137024:+	skipped	ENSG00000143224	1:161136199:161141008:+	PPOX
1:171558508:171558744:+	skipped	ENSG00000117523	1:171454650:171560986:+	PRRC2C
1:172546679:172546699:+	skipped	ENSG00000094975	1:172501488:172580971:+	SUCO
1:179846374:179846508:-	skipped	ENSG00000169905	1:179834199:179846925:-	TOR1AIP2
1:197069561:197074315:-	skipped	ENSG00000066279	1:197053257:197115824:-	ASPM
1:205743961:205744214:-	skipped	ENSG00000117280	1:205737113:205744574:-	RAB7L1
1:211485697:211485829:+	skipped	ENSG00000117625	1:211485578:211487181:+	RCOR3
1:221912276:221913129:-	skipped	ENSG00000143507	1:221874765:221915518:-	DUSP10
1:225541505:225541590:+	skipped	ENSG00000185842	1:225083963:225586971:+	DNAH14
1:227935393:227935934:+	skipped	ENSG00000143740	1:227935763:227947353:+	SNAP47
1:228681584:228681970:+	skipped	ENSG00000168159	1:228674761:228683467:+	RNF187
1:231135369:231135547:+	skipped	ENSG00000173409	1:231131505:231136341:+	ARV1
10:6268155:6268328:+	skipped	ENSG00000170525	10:6263366:6275070:+	PFKFB3
10:13364835:13365047:-	skipped	ENSG00000086475	10:13359423:13390297:-	SEPHS1
10:27442156:27442210:-	skipped	ENSG00000136758	10:27399382:27443288:-	YME1L1
10:32128565:32128639:-	skipped	ENSG00000165322	10:32094364:32197802:-	ARHGAP12
10:63974788:63974850:-	skipped	ENSG00000182010	10:63942793:63996022:-	RTKN2
10:64979638:64979743:-	skipped	ENSG00000171988	10:64926980:65225641:-	JMJD1C
10:75160561:75160615:-	skipped	ENSG00000138279	10:75156277:75168165:-	ANXA7
10:75239608:75239726:-	skipped	ENSG00000107758	10:75196185:75255759:-	PPP3CB
10:75898564:75898689:-	skipped	ENSG00000185009	10:75898013:75910515:-	AP3M1
10:76977691:76977749:+	skipped	ENSG00000165637	10:76969911:76991202:+	VDAC2

Table S1. Exons affected by SWI/SNF ATPase subunits

BRM-mut

Affected exon coordinates	included/skipped	ENSEMBL gene ID	gene coordinates	Gene name
10:105155503:105155789:-	skipped	ENSG00000173915	10:105148797:105155780:-	USMG5
10:120081817:120081923:-	skipped	ENSG00000165669	10:120065400:120101832:-	FAM204A
10:124187792:124187936:+	skipped	ENSG00000107679	10:124134211:124191867:+	PLEKHA1
11:908542:908645:-	skipped	ENSG00000177830	11:902196:915058:-	CHID1
11:3838583:3838765:+	skipped	ENSG00000148985	11:3837418:3845402:+	PGAP2
11:14901682:14902314:-	skipped	ENSG00000186104	11:14899552:14908880:-	CYP2R1
11:18339298:18339454:-	skipped	ENSG00000110756	11:18300222:18343721:-	HPS5
11:32949508:32949669:+	skipped	ENSG00000060749	11:32948254:32953383:+	QSER1
11:47281342:47281530:+	skipped	ENSG00000025434	11:47269850:47283525:+	NR1H3
11:47493550:47493654:-	skipped	ENSG00000149187	11:47491845:47497009:-	CELF1
11:57558857:57559145:+	skipped	ENSG00000198561	11:57559030:57569266:+	CTNND1
11:57558966:57559145:+	skipped	ENSG00000198561	11:57559030:57569266:+	CTNND1
11:66391685:66393149:+	skipped	ENSG00000239306	11:66384052:66394818:+	RBM14
11:66410921:66411611:+	skipped	ENSG00000173933	11:66406087:66413940:+	RBM4
11:70267576:70267686:+	skipped	ENSG00000085733	11:70266328:70268023:+	CTTN
11:71502782:71502865:+	skipped	ENSG00000158483	11:71498555:71512280:+	FAM86C1
11:74651830:74651927:-	skipped	ENSG00000166435	11:74617397:74660065:-	XRRA1
11:95555035:95555142:+	skipped	ENSG00000166037	11:95523128:95564431:+	CEP57
11:107686516:107686691:-	skipped	ENSG00000110660	11:107661716:107729914:-	SLC35F2
11:111959591:111959735:+	skipped	ENSG00000204370	11:111957496:111966518:+	SDHD
11:118897216:118897398:-	skipped	ENSG00000137700	11:118894823:118901616:-	SLC37A4
11:118897313:118897398:-	skipped	ENSG00000137700	11:118894823:118901616:-	SLC37A4
11:120925761:120926017:+	skipped	ENSG00000154114	11:120894780:120957805:+	TBCEL
11:129993507:129993674:+	skipped	ENSG00000084234	11:129939731:130013417:+	APLP2
12:1013619:1013660:+	skipped	ENSG00000060237	12:1009763:1013653:+	WNK1
12:3973002:3973123:-	skipped	ENSG00000111224	12:3938139:3982608:-	PARP11
12:6991360:6991491:+	skipped	ENSG00000111671	12:6981818:6991451:-	SPSB2
12:8340328:8340821:+	skipped	ENSG00000196946	12:8328452:8341133:+	ZNF705A
12:9147715:9147884:+	skipped	ENSG00000139187	12:9102639:9162044:+	KLRG1
12:10761697:10761982:-	skipped	ENSG00000111196	12:10758611:10766222:-	MAGOHB
12:27529279:27529320:+	skipped	ENSG00000029153	12:27485786:27576241:+	ARNTL2
12:30839255:30839593:-	skipped	ENSG00000133704	12:30837234:30841680:-	IPO8
12:31632523:31633189:-	skipped	ENSG00000170456	12:31604635:31648836:-	DENND5B
12:32866143:32866305:+	skipped	ENSG00000087470	12:32832133:32898486:+	DNM1L
12:32890799:32890876:+	skipped	ENSG00000087470	12:32884402:32891085:+	DNM1L
12:42606246:42606286:-	skipped	ENSG00000015153	12:42550905:42632050:-	YAF2
12:42629416:42629557:-	skipped	ENSG00000015153	12:42625025:42632050:-	YAF2
12:49397341:49397378:-	skipped	ENSG00000181929	12:49396056:49397376:-	PRKAG1
12:57348690:57348948:-	skipped	ENSG00000139547	12:57345218:57352103:-	RDH16
12:58022831:58022957:-	skipped	ENSG00000135454	12:58017192:58026934:-	B4GALNT1
12:76467983:76468019:-	skipped	ENSG00000187109	12:76467584:76478463:-	NAP1L1
12:80199946:80200077:-	skipped	ENSG00000058272	12:80190696:80211223:-	PPP1R12A
12:89744365:89744802:-	skipped	ENSG00000139318	12:89741008:89747048:-	DUSP6
12:96370207:96370276:-	skipped	ENSG00000084110	12:96366439:96390143:-	HAL
12:96640718:96641512:+	skipped	ENSG00000111145	12:96640908:96661135:+	ELK3
12:100655508:100655546:-	skipped	ENSG00000166153	12:100649650:100660833:-	DEPDC4
12:104682709:104682818:+	skipped	ENSG00000198431	12:104680809:104683181:+	TXNRD1
12:107264601:107264720:+	skipped	ENSG00000111785	12:107264600:107280138:+	RIC8B
12:109046814:109046836:-	skipped	ENSG00000110880	12:109038884:109125372:-	CORO1C
12:110937340:110937351:-	skipped	ENSG00000111237	12:110936600:110939912:-	VPS29
12:131357129:131357174:+	skipped	ENSG00000132341	12:131356423:131362223:+	RAN
13:33109906:33111164:-	skipped	ENSG00000244754	13:33101011:33110520:-	N4BP2L2

Table S1. Exons affected by SWI/SNF ATPase subunits

BRM-mut

Affected exon coordinates	included/skipped	ENSEMBL gene ID	gene coordinates	Gene name
13:76111800:76111811:-	skipped	ENSG00000188243	13:76103844:76111980:-	COMMD6
13:98659616:98659841:+	skipped	ENSG00000065150	13:98655262:98659841:+	IPO5
14:21731826:21731988:-	skipped	ENSG00000092199	14:21730761:21737601:-	HNRNPC
14:21990967:21993065:-	skipped	ENSG00000165821	14:21989672:21994634:-	SALL2
14:23469166:23469326:-	skipped	ENSG00000100802	14:23465448:23471978:-	C14orf93
14:33525111:33525200:+	skipped	ENSG00000151322	14:33404138:34270315:+	NPAS3
14:39516571:39516642:-	skipped	ENSG00000100934	14:39501122:39557927:-	SEC23A
14:39594196:39594240:+	skipped	ENSG00000092208	14:39583426:39606177:+	GEMIN2
14:55251058:55251165:+	skipped	ENSG00000020577	14:55250983:55255846:+	SAMD4A
14:59951708:59951834:+	skipped	ENSG00000050130	14:59951209:59961870:+	JKAMP
14:69259599:69259787:-	skipped	ENSG00000185650	14:69255321:69261453:-	ZFP36L1
14:69925080:69925446:+	skipped	ENSG00000029364	14:69921417:69925260:+	SLC39A9
14:75374138:75374233:-	skipped	ENSG00000198208	14:75372345:75374160:-	RPS6KL1
14:93730131:93730339:-	skipped	ENSG00000011114	14:93721855:93760963:-	BTBD7
14:100835424:100835595:-	skipped	ENSG00000140105	14:100828039:100841810:-	WARS
14:102568212:102568422:-	skipped	ENSG00000080824	14:102547385:102606023:-	HSP90AA1
14:102797959:102798183:+	skipped	ENSG00000022976	14:102797858:102805167:+	ZNF839
15:41104897:41105100:+	skipped	ENSG00000166140	15:41099283:41106543:+	ZFYVE19
15:41191342:41193212:+	skipped	ENSG00000104142	15:41186627:41196173:+	VPS18
15:41556363:41556433:+	skipped	ENSG00000187446	15:41523036:41571633:+	CHP1
15:43857065:43857190:-	skipped	ENSG00000168781	15:43825659:43877062:-	PPIP5K1
15:52856387:52856443:-	skipped	ENSG00000128989	15:52839241:52861436:-	ARPP19
15:60689457:60689537:-	skipped	ENSG00000182718	15:60687845:60690185:-	ANXA2
15:63356263:63356389:+	skipped	ENSG00000140416	15:63334830:63358292:+	TPM1
15:63826002:63826117:+	skipped	ENSG00000140455	15:63820031:63829255:+	USP3
15:64981283:64981368:-	skipped	ENSG00000180304	15:64979774:64988502:-	OAZ2
15:65243169:65243448:+	skipped	ENSG00000166839	15:65204100:65251039:+	ANKDD1A
15:65899640:65899780:-	skipped	ENSG00000138614	15:65897520:65903461:-	VWA9
15:83087938:83087977:+	skipped	ENSG00000259429	15:83084256:83088121:+	UBE2Q2P3
15:86311248:86313051:-	skipped	ENSG00000183655	15:86302553:86338261:-	KLHL25
15:90421497:90421532:-	skipped	ENSG00000157823	15:90373830:90437574:-	AP3S2
15:91446461:91446964:+	skipped	ENSG00000196547	15:91445447:91448566:+	MAN2A2
15:99874128:99874334:+	skipped	ENSG00000168904	15:99791590:99874335:+	LRRC28
15:99874223:99874334:+	skipped	ENSG00000168904	15:99791590:99874335:+	LRRC28
16:684889:684956:-	skipped	ENSG00000130731	16:684428:686342:-	C16orf13
16:2012498:2012657:-	skipped	ENSG00000140988	16:2012052:2014861:-	RPS2
16:3447192:3447744:-	skipped	ENSG00000262621	16:3415111:3536953:+	NAA60
16:4927386:4927475:+	skipped	ENSG00000118900	16:4925272:4930446:+	UBN1
16:15719229:15719657:-	skipped	ENSG00000166783	16:15688242:15737023:-	KIAA0430
16:16232221:16232415:+	skipped	ENSG00000103222	16:16043433:16236931:+	ABCC1
16:18799385:18799481:-	skipped	ENSG00000134419	16:18792616:18801656:-	RPS15A
16:22437534:22437687:-	skipped	ENSG00000257122	16:22430089:22449036:-	RRN3P3
16:22528086:22528423:+	skipped	ENSG00000243716	16:22516171:22538951:+	NPIPB5
16:27231714:27231806:+	skipped	ENSG00000155666	16:27231638:27232223:+	KDM8
16:28993222:28993377:+	skipped	ENSG00000169682	16:28986092:28995869:+	SPNS1
16:29870763:29870844:-	skipped	ENSG00000103502	16:29869677:29875057:-	CDIPT
16:57212414:57212764:-	skipped	ENSG00000172775	16:57186377:57219976:-	FAM192A
16:68573661:68573728:+	skipped	ENSG00000184939	16:68563992:68592009:+	ZFP90
16:69155339:69155396:-	skipped	ENSG00000168802	16:69152036:69155395:-	CHTF8
16:69680420:69680481:+	skipped	ENSG00000102908	16:69599928:69719978:+	NFAT5
16:71678654:71678758:-	skipped	ENSG00000040199	16:71671737:71715757:-	PHLPP2
16:89755660:89755732:+	skipped	ENSG00000185324	16:89753075:89762772:+	CDK10

Table S1. Exons affected by SWI/SNF ATPase subunits

BRM-mut

Affected exon coordinates	included/skipped	ENSEMBL gene ID	gene coordinates	Gene name
17:1617665:1617747:-	skipped	ENSG00000186594	17:1614804:1619545:-	MIR22HG
17:3727354:3727386:-	skipped	ENSG00000074356	17:3725797:3729411:-	C17orf85
17:6913115:6913208:-	skipped	ENSG00000215067	17:6888427:6915653:-	AC027763.2
17:7490217:7490335:+	skipped	ENSG00000129255	17:7489786:7491135:+	MPDU1
17:16691249:16691449:+	skipped	ENSG00000266129	17:16690548:16691455:-	SRP68P1
17:18762653:18762788:+	skipped	ENSG00000141127	17:18761408:18775941:+	PRPSAP2
17:28958773:28958945:+	skipped	ENSG00000250462	17:28956697:28964320:+	LRRC37BP1
17:34800103:34800168:-	skipped	ENSG00000161583	17:34745935:34808103:-	TBC1D3G
17:35939228:35939530:-	skipped	ENSG00000006114	17:35926950:35961035:-	SYNRG
17:37883548:37883800:+	skipped	ENSG00000141736	17:37844166:37884614:+	ERBB2
17:38251697:38252340:-	skipped	ENSG00000126368	17:38249039:38256978:-	NR1D1
17:39973310:39973455:+	skipped	ENSG00000141756	17:39968931:39974748:+	FKBP10
17:42786672:42786734:+	skipped	ENSG00000161692	17:42785975:42829632:+	DBF4B
17:42971785:42971893:-	skipped	ENSG00000108883	17:42927310:42977030:-	EFTUD2
17:47784705:47784806:-	skipped	ENSG00000121073	17:47778304:47785317:-	SLC35B1
17:56725353:56725473:-	skipped	ENSG00000121101	17:56634038:56769416:-	TEX14
17:57943970:57944110:-	skipped	ENSG00000108423	17:57936850:57970296:-	TUBD1
17:61803998:61804055:-	skipped	ENSG00000266173	17:61780191:61819143:-	STRADA
17:65882244:65882432:+	skipped	ENSG00000171634	17:65871035:65888078:+	BPTF
17:73888092:73888266:-	skipped	ENSG00000141569	17:73876415:73888384:-	TRIM65
17:78252563:78252709:+	skipped	ENSG00000173821	17:78234664:78369112:+	RNF213
18:11884878:11885042:-	skipped	ENSG00000154889	18:11884164:11885120:-	MPPE1
18:12485958:12485999:-	skipped	ENSG00000134278	18:12449664:12493148:-	SPIRE1
18:19014908:19015036:+	skipped	ENSG00000141449	18:18943553:19105378:+	GREB1L
18:19029471:19029797:+	skipped	ENSG00000141449	18:19029595:19032394:+	GREB1L
18:23658975:23659110:-	skipped	ENSG00000141380	18:23596577:23670589:-	SS18
18:47017775:47017815:-	skipped	ENSG00000215472	18:47008050:47017956:-	RPL17-C18orf32
19:4179101:4179283:-	skipped	ENSG00000077463	19:4174105:4182601:-	SIRT6
19:12787922:12788025:-	skipped	ENSG00000095059	19:12786530:12790334:-	DHPS
19:16665963:16666208:-	skipped	ENSG00000127526	19:16664199:16675066:-	SLC35E1
19:19788673:19788795:+	skipped	ENSG00000181896	19:19779604:19790332:+	ZNF101
19:32838151:32838244:+	skipped	ENSG00000168813	19:32836499:32878573:+	ZNF507
19:36235527:36235639:-	skipped	ENSG00000267120	19:36230152:36236333:-	AD000671.6
19:37316282:37316424:+	skipped	ENSG00000267254	19:37288464:37319003:+	CTD-2162K18.5
19:47589641:47589795:-	skipped	ENSG00000130749	19:47567443:47617009:-	ZC3H4
19:48735710:48736046:-	skipped	ENSG00000105483	19:48706888:48753085:-	CARD8
19:50203941:50204112:+	skipped	ENSG00000169169	19:50194366:50216988:+	CPT1C
19:57986392:57986514:-	skipped	ENSG00000197128	19:57984412:57988900:-	ZNF772
19:58003481:58003579:+	skipped	ENSG00000105136	19:57999078:58005458:+	ZNF419
2:9616116:9616168:+	skipped	ENSG00000134330	2:9613786:9624677:+	IAH1
2:20113798:20114038:-	skipped	ENSG00000118965	2:20113002:20175397:-	WDR35
2:26999240:26999350:+	skipped	ENSG00000213699	2:26987151:27004008:+	SLC35F6
2:27354540:27354699:-	skipped	ENSG00000138073	2:27353623:27357543:-	PREB
2:39984388:39984508:-	skipped	ENSG00000138050	2:39963199:39997153:-	THUMPD2
2:47136162:47136316:-	skipped	ENSG00000180398	2:47132955:47136211:-	MCFD2
2:63486446:63486544:-	skipped	ENSG00000143951	2:63348517:63815933:-	WDPCP
2:73635750:73635875:+	skipped	ENSG00000116127	2:73612885:73836842:+	ALMS1
2:86769375:86769435:-	skipped	ENSG00000115561	2:86754170:86769436:-	CHMP3
2:110350628:110350696:-	skipped	ENSG00000186522	2:110300558:110371783:-	SEPT10
2:114351865:114352093:+	skipped	ENSG00000146556	2:114346126:114356611:+	WASH2P
2:130858249:130858293:-	skipped	ENSG00000196604	2:130831107:130878182:-	POTEF
2:131897740:131897848:+	skipped	ENSG00000115762	2:131862419:131905370:+	PLEKHB2

Table S1. Exons affected by SWI/SNF ATPase subunits

BRM-mut

Affected exon coordinates	included/skipped	ENSEMBL gene ID	gene coordinates	Gene name
2:160287374:160287667:-	skipped	ENSG00000123636	2:160260882:160292064:-	BAZ2B
2:172563743:172563887:+	skipped	ENSG00000268821	2:172560843:172567314:-	AC068039.1
2:177498093:177498468:-	skipped	ENSG00000163364	2:177494316:177502659:-	LINC01116
2:178998588:178998627:+	skipped	ENSG00000155636	2:178988589:179003738:+	RBM45
2:179396041:179396305:+	skipped	ENSG00000237298	2:179388630:179396307:+	TTN-AS1
2:202141550:202141827:+	skipped	ENSG00000064012	2:202122702:202141627:+	CASP8
2:204143296:204143438:+	skipped	ENSG00000119004	2:204103662:204162068:+	CYP20A1
2:207025611:207025856:+	skipped	ENSG00000114942	2:207024308:207027652:+	EEF1B2
2:207625625:207625737:-	skipped	ENSG00000138400	2:207621342:207630025:-	MDH1B
2:208486530:208486641:-	skipped	ENSG00000144401	2:208445354:208489845:-	METTL21A
2:219492752:219494386:+	skipped	ENSG00000115556	2:219472487:219501904:+	PLCD4
2:220072370:220072496:+	skipped	ENSG00000158552	2:220071505:220073622:+	ZFAND2B
2:228211942:228212100:+	skipped	ENSG00000168958	2:228205007:228222097:+	MFF
2:232655632:232655883:+	skipped	ENSG00000144524	2:232653398:232660855:+	COPS7B
2:240961589:240961757:-	skipped	ENSG00000130414	2:240960533:240964741:-	NDUFA10
2:242208244:242208710:-	skipped	ENSG00000115677	2:242206226:242212227:-	HDLBP
2:242259618:242259702:+	skipped	ENSG00000168385	2:242255308:242264659:+	SEPT2
20:1356135:1356247:-	skipped	ENSG00000088832	20:1349621:1356247:-	FKBP1A
20:23350221:23350378:+	skipped	ENSG00000125812	20:23349507:23351058:+	GZF1
20:32161780:32162067:+	skipped	ENSG00000078699	20:32150139:32211760:+	CBFA2T2
20:32211579:32211660:+	skipped	ENSG00000078699	20:32150139:32211760:+	CBFA2T2
20:33117656:33118038:+	skipped	ENSG00000125971	20:33104213:33128762:+	DYNLRB1
20:35219313:35219412:+	skipped	ENSG00000259716	20:35177529:35232932:-	RP5-977B1.11
20:45891029:45891172:-	skipped	ENSG00000101040	20:45837858:45984401:-	ZMYND8
20:48467347:48467381:+	skipped	ENSG00000197818	20:48429249:48508772:+	SLC9A8
20:48524692:48525129:-	skipped	ENSG00000158480	20:48519927:48532045:-	SPATA2
20:48713020:48713071:-	skipped	ENSG00000244687	20:48697660:48732491:-	UBE2V1
20:48713209:48713348:-	skipped	ENSG00000244687	20:48697660:48732491:-	UBE2V1
20:49458303:49458437:+	skipped	ENSG00000124243	20:49411430:49493714:+	BCAS4
20:52188260:52188392:-	skipped	ENSG00000171940	20:52185358:52192566:-	ZNF217
20:55045656:55045807:+	skipped	ENSG00000022277	20:55043713:55048416:+	RTFDC1
20:55968335:55968389:+	skipped	ENSG00000132819	20:55966462:55984389:+	RBM38
20:61833639:61835159:-	skipped	ENSG00000149658	20:61826780:61847586:-	YTHDF1
21:15591874:15592046:+	skipped	ENSG00000185272	21:15588474:15593774:+	RBM11
21:27484296:27484463:-	skipped	ENSG00000142192	21:27461826:27484400:-	APP
21:46281078:46281186:-	skipped	ENSG00000183255	21:46271321:46281848:-	PTTG1IP
22:18232871:18232940:-	skipped	ENSG00000015475	22:18220823:18257261:-	BID
22:24029018:24029182:-	skipped	ENSG00000272578	22:24020796:24032484:-	AP000347.2
22:26875231:26875321:-	skipped	ENSG00000100099	22:26871335:26875377:-	HPS4
22:29130391:29130715:-	skipped	ENSG00000183765	22:29107896:29137771:-	CHEK2
22:29706817:29706988:+	skipped	ENSG00000185340	22:29706268:29707097:+	GAS2L1
22:30419446:30419472:+	skipped	ENSG00000100330	22:30418451:30421952:+	MTMR3
22:30640744:30640922:-	skipped	ENSG00000128342	22:30639521:30642728:-	LIF
22:31685301:31685379:-	skipped	ENSG00000100100	22:31677578:31688514:-	PIK3IP1
22:35658316:35658414:+	skipped	ENSG00000100281	22:35653444:35661563:+	HMGXB4
22:35717952:35718012:+	skipped	ENSG00000100284	22:35695267:35743985:+	TOM1
22:39052961:39053148:+	skipped	ENSG00000100211	22:39052640:39064472:+	CBY1
22:42914619:42914734:-	skipped	ENSG00000189306	22:42910682:42915800:-	RRP7A
22:45571775:45571961:+	skipped	ENSG00000093000	22:45564046:45574781:+	NUP50
3:17627845:17628049:-	skipped	ENSG00000131374	3:17198653:17782399:-	TBC1D5
3:33450182:33450289:-	skipped	ENSG00000153560	3:33442453:33454248:-	UBP1
3:47544615:47544939:-	skipped	ENSG00000163832	3:47537129:47555251:-	ELP6

Table S1. Exons affected by SWI/SNF ATPase subunits

BRM-mut

Affected exon coordinates	included/skipped	ENSEMBL gene ID	gene coordinates	Gene name
3:47894653:47894842:-	skipped	ENSG00000047849	3:47892181:47951731:-	MAP4
3:48341921:48342124:-	skipped	ENSG00000172113	3:48334753:48342848:-	NME6
3:48731892:48731958:-	skipped	ENSG00000068745	3:48726970:48733021:-	IP6K2
3:48982415:48982614:+	skipped	ENSG00000177479	3:48956253:48999112:+	ARIH2
3:49212495:49212596:+	skipped	ENSG00000185909	3:49209043:49213917:+	KLHDC8B
3:52588740:52588895:-	skipped	ENSG00000168273	3:52570620:52613253:+	SMIM4
3:73112824:73112898:+	skipped	ENSG00000163605	3:73045935:73118350:+	PPP4R2
3:93782656:93782968:+	skipped	ENSG00000178694	3:93781759:93847389:+	NSUN3
3:98240497:98240562:-	skipped	ENSG00000080822	3:98239562:98241746:-	CLDND1
3:101498578:101498744:+	skipped	ENSG00000144815	3:101498045:101504450:+	NXPE3
3:119197181:119197321:+	skipped	ENSG00000163389	3:119196969:119198826:+	POGLUT1
3:124527869:124528003:-	skipped	ENSG00000082781	3:124480794:124606144:-	ITGB5
3:126334198:126334305:-	skipped	ENSG00000197763	3:126325894:126373998:-	TXNRD3
3:131220688:131220768:-	skipped	ENSG00000114686	3:131206524:131221795:-	MRPL3
3:132379743:132379892:+	skipped	ENSG00000081307	3:132378761:132379789:+	UBA5
3:132418197:132418294:-	skipped	ENSG00000113971	3:132399453:132441276:-	NPHP3
3:141712380:141712427:-	skipped	ENSG00000114126	3:141707908:141712427:-	TFDP2
3:150140836:150140907:+	skipped	ENSG00000196428	3:150140825:150141238:+	TSC22D2
3:183354009:183354099:+	skipped	ENSG00000114796	3:183353397:183368526:+	KLHL24
4:466364:466490:-	skipped	ENSG00000251595	4:419223:467918:-	ABCA11P
4:1729435:1730514:+	skipped	ENSG00000013810	4:1723261:1729959:+	TACC3
4:2243247:2243564:-	skipped	ENSG00000214367	4:2229190:2243848:-	HAUS3
4:13371495:13371589:-	skipped	ENSG00000157869	4:13370124:13383218:-	RAB28
4:39779302:39779430:+	skipped	ENSG00000078140	4:39699663:39784412:+	UBE2K
4:40101656:40101746:+	skipped	ENSG00000078177	4:40058555:40104226:+	N4BP2
4:52777236:52777340:+	skipped	ENSG00000109184	4:52775565:52780268:+	DCUN1D4
4:71607344:71607397:+	skipped	ENSG00000018189	4:71570140:71634339:+	RUFY3
4:71891533:71891648:+	skipped	ENSG00000156136	4:71891600:71892516:+	DCK
4:74012958:74013149:-	skipped	ENSG00000132466	4:73968191:74027065:-	ANKRD17
4:83292681:83292737:-	skipped	ENSG00000138668	4:83279910:83295314:-	HNRNPD
4:89199296:89199794:-	skipped	ENSG00000163644	4:89196075:89200852:-	PPM1K
4:89199688:89199794:-	skipped	ENSG00000163644	4:89196075:89200852:-	PPM1K
4:99969884:99970027:+	skipped	ENSG00000164024	4:99916770:99983964:+	METAP1
4:100849699:100849774:-	skipped	ENSG00000164031	4:100844196:100851746:-	DNAJB14
4:103674980:103675152:-	skipped	ENSG00000109323	4:103552659:103682098:-	MANBA
4:119687684:119687978:-	skipped	ENSG00000150961	4:119685567:119689402:-	SEC24D
4:123147862:123147990:+	skipped	ENSG00000138688	4:123107335:123161084:+	KIAA1109
4:123247010:123247072:+	skipped	ENSG00000138688	4:123230519:123283905:+	KIAA1109
4:123818779:123818833:-	skipped	ENSG00000138685	4:123747862:123819391:+	FGF2
4:124318766:124319011:+	skipped	ENSG00000164056	4:124317965:124319492:+	SPRY1
4:152022127:152022314:+	skipped	ENSG00000145425	4:152020724:152025804:+	RPS3A
4:152403676:152403800:+	skipped	ENSG00000164142	4:152330367:152487410:+	FAM160A1
4:158283563:158283636:+	skipped	ENSG00000120251	4:158281851:158284086:+	GRIA2
4:185691553:185691630:-	skipped	ENSG00000151726	4:185689481:185695027:-	ACSL1
5:31535066:31535158:+	skipped	ENSG00000082213	5:31532401:31538793:+	C5orf22
5:43453760:43454145:-	skipped	ENSG00000151881	5:43444353:43483938:-	C5orf28
5:68667281:68667384:+	skipped	ENSG00000152942	5:68665644:68667296:+	RAD17
5:69206202:69206389:+	skipped	ENSG00000198237	5:69171097:69216694:+	RP11-98J23.2
5:69492705:69492797:-	skipped	ENSG00000254701	5:69435401:69493223:-	RP11-1415C14.4
5:81272012:81272146:+	skipped	ENSG00000152348	5:81267844:81283498:+	ATG10
5:82357696:82357730:-	skipped	ENSG00000174695	5:82352353:82357794:-	TMEM167A
5:87517491:87517577:-	skipped	ENSG00000164180	5:87485449:87564647:-	TMEM161B

Table S1. Exons affected by SWI/SNF ATPase subunits

BRM-mut

Affected exon coordinates	included/skipped	ENSEMBL gene ID	gene coordinates	Gene name
5:137292166:137292231:-	skipped	ENSG00000031003	5:137273648:137368720:-	FAM13B
5:137495244:137495288:-	skipped	ENSG00000112983	5:137495263:137496924:-	BRD8
5:138463633:138463741:-	skipped	ENSG00000120725	5:138443695:138532217:-	SIL1
5:138614254:138614429:+	skipped	ENSG00000015479	5:138609790:138615528:+	MATR3
5:138615624:138615747:+	skipped	ENSG00000015479	5:138609791:138618678:+	MATR3
5:147806776:147807510:+	skipped	ENSG00000145868	5:147803311:147806833:+	FBXO38
5:153832016:153832059:+	skipped	ENSG00000164576	5:153831957:153835709:+	SAP30L
5:171341347:171341409:-	skipped	ENSG00000072803	5:171326936:171433663:-	FBXW11
5:179048844:179048977:-	skipped	ENSG00000169045	5:179044817:179050672:-	HNRNPH1
5:179278243:179278424:-	skipped	ENSG00000161010	5:179274356:179285808:-	C5orf45
6:2397455:2397860:+	skipped	ENSG00000250903	6:2391779:2413825:+	GMDS-AS1
6:26368218:26368495:+	skipped	ENSG00000186470	6:26365386:26370831:+	BTN3A2
6:30657053:30657247:-	skipped	ENSG00000137404	6:30655823:30658500:-	NRM
6:30853402:30853457:+	skipped	ENSG00000204580	6:30851221:30857046:+	DDR1
6:31856746:31856847:-	skipped	ENSG00000204371	6:31847535:31865461:-	EHMT2
6:32939372:32940704:+	skipped	ENSG00000204256	6:32936941:32939918:+	BRD2
6:33289496:33289785:-	skipped	ENSG00000204209	6:33286334:33290787:-	DAXX
6:35387904:35388058:+	skipped	ENSG00000112033	6:35310334:35395968:+	PPARD
6:36567598:36568053:+	skipped	ENSG00000112081	6:36562144:36568658:+	SRSF3
6:41884523:41884677:-	skipped	ENSG00000124641	6:41884593:41888843:-	MED20
6:41904297:41904433:-	skipped	ENSG00000112576	6:41902670:41909586:-	CCND3
6:44224079:44224233:-	skipped	ENSG00000157593	6:44221832:44225291:-	SLC35B2
6:90981568:90981660:-	skipped	ENSG00000112182	6:90636247:91006461:-	BACH2
6:99863445:99863623:-	skipped	ENSG00000132424	6:99845926:99873184:-	PNISR
6:99864225:99864304:-	skipped	ENSG00000132424	6:99845926:99873184:-	PNISR
6:99930629:99930759:-	skipped	ENSG00000123552	6:99880189:99963252:-	USP45
6:109461624:109461878:+	skipped	ENSG00000183137	6:109450506:109485113:+	CEP57L1
6:111805947:111806064:+	skipped	ENSG00000231889	6:111804713:111814206:+	TRAF3IP2-AS1
6:117864287:117864374:+	skipped	ENSG00000164465	6:117859937:117869091:+	DCBLD1
6:127652975:127653012:-	skipped	ENSG00000093144	6:127609856:127664020:-	ECHDC1
6:130370796:130370975:+	skipped	ENSG00000198945	6:130339727:130372488:+	L3MBTL3
6:130370901:130370975:+	skipped	ENSG00000198945	6:130339727:130372488:+	L3MBTL3
6:144269122:144269597:-	skipped	ENSG00000118495	6:144261436:144290115:-	PLAGL1
6:167426996:167427055:+	skipped	ENSG00000213066	6:167412669:167466201:+	FGFR1OP
7:2584543:2584690:-	skipped	ENSG00000106009	7:2577510:2595361:-	BRAT1
7:5568792:5569294:-	skipped	ENSG00000075624	7:5566781:5570340:-	ACTB
7:7607657:7607754:+	skipped	ENSG00000164654	7:7606591:7612556:+	MIOS
7:14028893:14029089:-	skipped	ENSG00000006468	7:14027783:14029291:-	ETV1
7:16643174:16643285:-	skipped	ENSG00000106524	7:16639400:16685442:-	ANKMY2
7:30503225:30503314:-	skipped	ENSG00000106100	7:30464142:30518396:-	NOD1
7:35872408:35872510:+	skipped	ENSG00000122545	7:35840828:35872455:+	SEPT7
7:44161881:44162058:-	skipped	ENSG00000106628	7:44155715:44163136:-	POLD2
7:44880498:44880611:-	skipped	ENSG00000105968	7:44866389:44887676:-	H2AFV
7:56146057:56146201:+	skipped	ENSG00000129103	7:56131694:56147817:+	SUMF2
7:65444821:65444898:-	skipped	ENSG00000169919	7:65439343:65445398:-	GUSB
7:72514920:72515008:+	skipped	ENSG00000174384	7:72507940:72515008:+	RP11-313P13.4
7:73663342:73663550:-	skipped	ENSG00000049541	7:73645828:73668719:-	RFC2
7:73969768:73969824:+	skipped	ENSG00000006704	7:73960091:74015433:+	GTF2IRD1
7:74317004:74317092:+	skipped	ENSG00000123965	7:74306893:74322330:+	PMS2P5
7:75039889:75040009:+	skipped	ENSG00000223705	7:75039604:75040822:+	NSUN5P1
7:91671360:91671500:+	skipped	ENSG00000127914	7:91670017:91672457:+	AKAP9
7:99081362:99081476:+	skipped	ENSG00000198556	7:99081277:99084627:+	ZNF789

Table S1. Exons affected by SWI/SNF ATPase subunits

BRM-mut

Affected exon coordinates	included/skipped	ENSEMBL gene ID	gene coordinates	Gene name
7:99661411:99661602:+	skipped	ENSG00000166529	7:99654533:99661878:+	ZSCAN21
7:99700298:99700369:+	skipped	ENSG00000221838	7:99699171:99701271:+	AP4M1
7:99950996:99951635:+	skipped	ENSG00000272752	7:99948878:99955915:+	STAG3L5P-PVRIG2P-PILRB
7:100402788:100402943:-	skipped	ENSG00000196411	7:100400186:100425121:-	EPHB4
7:101843351:101843452:+	skipped	ENSG00000257923	7:101458958:101927249:+	CUX1
7:102076649:102076780:+	skipped	ENSG00000160991	7:102073552:102079494:+	ORAI2
7:102079391:102079628:+	skipped	ENSG00000160991	7:102073552:102079494:+	ORAI2
7:102228681:102228892:-	skipped	ENSG00000105808	7:102222796:102234438:-	RASA4
7:102724477:102724509:+	skipped	ENSG00000170632	7:102715327:102740205:+	ARMC10
7:112126407:112126494:+	skipped	ENSG00000181016	7:112120907:112130203:+	LSMEM1
7:130039880:130040095:-	skipped	ENSG00000106477	7:130033611:130081078:-	CEP41
7:130737184:130737314:-	skipped	ENSG00000231721	7:130727594:130737309:-	LINC-PINT
7:139737515:139737656:-	skipped	ENSG00000059378	7:139723543:139763521:-	PARP12
7:140508037:140508116:-	skipped	ENSG00000157764	7:140434278:140624564:-	BRAF
7:143079341:143079778:+	skipped	ENSG00000159840	7:143078387:143080018:+	ZYX
7:148987029:148987174:+	skipped	ENSG00000244560	7:148982371:148990853:+	RP4-800G7.2
7:149575767:149575879:+	skipped	ENSG00000171130	7:149570056:149577782:+	ATP6V0E2
7:151854846:151855010:-	skipped	ENSG00000055609	7:151849983:151859249:-	KMT2C
8:16927197:16927228:+	skipped	ENSG00000155970	8:16921638:16977799:+	MICU3
8:17838100:17838264:+	skipped	ENSG00000078674	8:17837729:17840790:+	PCM1
8:26197419:26197489:+	skipped	ENSG00000221914	8:26149043:26212146:+	PPP2R2A
8:27147673:27147695:-	skipped	ENSG00000104228	8:27142403:27168836:-	TRIM35
8:27155981:27156076:-	skipped	ENSG00000104228	8:27142403:27168836:-	TRIM35
8:27954736:27954835:+	skipped	ENSG00000134014	8:27947189:27965481:+	ELP3
8:30612182:30612266:+	skipped	ENSG00000104691	8:30608913:30620839:+	UBXN8
8:53598497:53598716:-	skipped	ENSG00000023287	8:53535015:53626992:-	RB1CC1
8:57879669:57879777:-	skipped	ENSG00000104331	8:57870491:57906403:-	IMPAD1
8:91018264:91018505:+	skipped	ENSG00000104325	8:91013704:91018461:+	DECR1
8:92088820:92088959:+	skipped	ENSG00000155100	8:92082589:92091435:+	OTUD6B
8:101315388:101315593:-	skipped	ENSG00000034677	8:101269287:101315487:-	RNF19A
8:125530983:125531122:-	skipped	ENSG00000147687	8:125500725:125551319:-	TATDN1
8:128808140:128808254:+	skipped	ENSG00000249859	8:128808207:128808274:+	PVT1
8:145153984:145154108:-	skipped	ENSG00000179526	8:145153535:145154225:-	SHARPIN
9:2651415:2651498:+	skipped	ENSG00000147852	9:2621833:2660053:+	VLDLR
9:3301546:3301620:-	skipped	ENSG00000080298	9:3218296:3525983:-	RFX3
9:16429298:16429997:-	skipped	ENSG00000173068	9:16418540:16436370:-	BNC2
9:34636994:34637086:-	skipped	ENSG00000147955	9:34635806:34637680:-	SIGMAR1
9:67984833:67985114:+	skipped	ENSG00000155282	9:67977437:67987998:+	RP11-195B21.3
9:77693242:77693498:-	skipped	ENSG00000106733	9:77675488:77703106:-	NMRK1
9:99271955:99272071:-	skipped	ENSG00000081377	9:99264893:99329536:-	CDC14B
9:116770624:116770659:+	skipped	ENSG00000157657	9:116638561:116812501:+	ZNF618
9:128268589:128268696:-	skipped	ENSG00000119487	9:128201091:128268645:-	MAPKAP1
9:136905156:136905391:-	skipped	ENSG00000169925	9:136895426:136933139:-	BRD3
9:140470761:140470854:-	skipped	ENSG00000148399	9:140468658:140472992:-	DPH7
X:23892520:23892574:-	skipped	ENSG00000184831	X:23851469:23926051:-	APOO
X:44919267:44919401:+	skipped	ENSG00000147050	X:44918526:44922921:+	KDM6A
X:51637365:51637445:+	skipped	ENSG00000179222	X:51636741:51638848:+	MAGED1
X:80374229:80374258:-	skipped	ENSG00000198157	X:80370333:80377187:-	HMGN5
X:80450617:80450796:-	skipped	ENSG00000198157	X:80369199:80457441:-	HMGN5
X:100416517:100416558:+	skipped	ENSG00000102384	X:100353177:100418425:+	CENPI
X:110952193:110952276:+	skipped	ENSG00000101901	X:110909042:110956525:+	ALG13
X:122994017:122994143:+	skipped	ENSG00000101966	X:122993573:123019561:+	XIAP

Table S1. Exons affected by SWI/SNF ATPase subunits

BRM-mut

Affected exon coordinates	included/skipped	ENSEMBL gene ID	gene coordinates	Gene name
X:151884446:151884551:+	skipped	ENSG00000183305	X:151883081:151885563:+	MAGEA2B
X:151885385:151885450:+	skipped	ENSG00000183305	X:151883081:151885563:+	MAGEA2B
X:153073352:153073594:-	skipped	ENSG00000067840	X:153070333:153073594:-	PDZD4
X:153277980:153278129:-	skipped	ENSG00000184216	X:153275950:153285431:-	IRAK1
X:154294181:154294332:-	skipped	ENSG00000182712	X:154289896:154299637:-	CMC4
1:6946300:6946406:+	included	ENSG00000171735	1:6845523:6948261:+	CAMTA1
1:23660011:23660124:-	included	ENSG00000125944	1:23631180:23670829:-	HNRNPR
1:31791014:31791083:+	included	ENSG00000121766	1:31769841:31837783:+	ZCCHC17
1:32561261:32561363:+	included	ENSG00000121775	1:32537631:32568464:+	TMEM39B
1:33290914:33291086:+	included	ENSG00000116497	1:33282367:33292090:+	S100PBP
1:33797226:33797300:-	included	ENSG00000134686	1:33789226:33815412:-	PHC2
1:43314962:43315413:+	included	ENSG00000164011	1:43312279:43318148:+	ZNF691
1:43831939:43832082:-	included	ENSG00000066322	1:43829067:43833689:-	ELOVL1
1:46156646:46156782:+	included	ENSG00000159596	1:46153867:46159517:+	TMEM69
1:67293495:67293593:-	included	ENSG00000231080	1:67292624:67294241:+	RP11-342H21.2
1:85035614:85035822:-	included	ENSG00000117151	1:85015288:85040147:-	CTBS
1:93594835:93595005:+	included	ENSG00000143033	1:93586355:93600016:+	MTF2
1:93676359:93676483:+	included	ENSG00000122483	1:93646325:93682236:+	CCDC18
1:101458193:101458296:-	included	ENSG00000117543	1:101455619:101460706:-	DPH5
1:109939829:109939962:-	included	ENSG00000134243	1:109852191:109940573:-	SORT1
1:114255901:114256060:-	included	ENSG00000116793	1:114248386:114255950:-	PHTF1
1:117149114:117149173:-	included	ENSG00000143061	1:117117030:117210375:-	IGSF3
1:144339564:144339618:-	included	ENSG00000235398	1:144325551:144340593:-	LINC00623
1:144606838:144607061:+	included	ENSG00000225241	1:144593362:144621656:+	RP11-640M9.2
1:144617150:144617252:+	included	ENSG00000225241	1:144614531:144619419:+	RP11-640M9.2
1:144955216:144955292:-	included	ENSG00000178104	1:144952051:144955352:-	PDE4DIP
1:145457935:145458003:-	included	ENSG00000121851	1:145456235:145470387:-	POLR3GL
1:148342470:148342542:-	included	ENSG00000203832	1:148341795:148346929:-	NBPF20
1:148344640:148344742:-	included	ENSG00000203832	1:148341795:148346929:-	NBPF20
1:149671772:149671860:+	included	ENSG00000252925	1:149671722:149671868:-	RNU1-68P
1:151257682:151258550:+	included	ENSG00000143373	1:151254093:151259181:+	ZNF687
1:151298648:151298849:-	included	ENSG00000143393	1:151264272:151300191:-	PI4KB
1:155109304:155109427:+	included	ENSG00000169241	1:155107819:155110844:+	SLC50A1
1:155181918:155182010:+	included	ENSG00000173171	1:155178489:155183614:+	MTX1
1:155650173:155650247:-	included	ENSG00000163374	1:155629236:155658548:-	YY1AP1
1:155650207:155650247:-	included	ENSG00000163374	1:155629236:155658548:-	YY1AP1
1:155746186:155746272:-	included	ENSG00000116580	1:155736326:155746252:-	GON4L
1:155990057:155990137:-	included	ENSG00000163479	1:155978838:155990750:-	SSR2
1:156761489:156761584:+	included	ENSG00000143294	1:156720401:156761584:+	PRCC
1:160194852:160194909:-	included	ENSG00000132716	1:160185504:160232291:-	DCAF8
1:160231075:160231148:-	included	ENSG00000132716	1:160185504:160232291:-	DCAF8
1:179102447:179102509:-	included	ENSG00000143322	1:179068462:179112179:-	ABL2
1:201114237:201114463:-	included	ENSG00000116857	1:201103899:201140702:-	TMEM9
1:222763600:222763749:+	included	ENSG00000225265	1:222763166:222765828:+	RP11-378J18.3
1:225976736:225976843:+	included	ENSG00000143742	1:225965530:225978168:+	SRP9
1:242012411:242012518:+	included	ENSG00000174371	1:242011268:242013762:+	EXO1
1:245165423:245165534:+	included	ENSG00000203666	1:245165454:245194133:+	EFCAB2
10:13150138:13150289:+	included	ENSG00000123240	10:13142158:13151662:+	OPTN
10:30726105:30726335:+	included	ENSG00000107968	10:30723172:30728410:+	MAP3K8
10:35426710:35426807:+	included	ENSG00000095794	10:35415718:35501053:+	CREM
10:38383859:38384152:+	included	ENSG00000075407	10:38383263:38384487:+	ZNF37A
10:46923604:46923810:+	included	ENSG00000165874	10:46914471:46923688:+	FAM35BP

Table S1. Exons affected by SWI/SNF ATPase subunits

BRM-mut

Affected exon coordinates	included/skipped	ENSEMBL gene ID	gene coordinates	Gene name
10:51364705:51364771:-	included	ENSG00000244393	10:51253925:51371300:-	RP11-592B15.3
10:70893288:70893363:+	included	ENSG00000122958	10:70883267:70932617:+	VPS26A
10:70916763:70916919:+	included	ENSG00000122958	10:70883267:70932617:+	VPS26A
10:102743705:102743831:-	included	ENSG00000055950	10:102729214:102746953:-	MRPL43
10:123658356:123658484:-	included	ENSG00000107669	10:123499938:123687551:-	ATE1
10:124746850:124747020:+	included	ENSG00000179988	10:124713896:124749906:+	PSTK
10:126100542:126100769:-	included	ENSG00000065154	10:126095924:126106762:-	OAT
10:134155717:134155775:+	included	ENSG00000148814	10:134145613:134195010:+	LRRC27
10:134179803:134180181:+	included	ENSG00000148814	10:134178974:134180505:+	LRRC27
11:225673:226081:-	included	ENSG00000142082	11:215457:236431:-	SIRT3
11:504824:504996:-	included	ENSG00000023191	11:494512:506821:-	RNH1
11:14529202:14529312:-	included	ENSG00000129084	11:14526419:14532577:-	PSMA1
11:17308181:17308260:+	included	ENSG00000070081	11:17229699:17316971:+	NUCB2
11:18312989:18313566:-	included	ENSG00000110756	11:18305339:18313489:-	HPS5
11:18314446:18314523:-	included	ENSG00000110756	11:18300222:18343721:-	HPS5
11:18347494:18347700:+	included	ENSG00000110768	11:18343841:18387808:+	GTF2H1
11:33612783:33612967:+	included	ENSG00000110427	11:33563617:33631438:+	KIAA1549L
11:43356828:43356904:+	included	ENSG00000166181	11:43356828:43360923:+	API5
11:57505258:57505498:+	included	ENSG00000213593	11:57480071:57506653:+	TMX2
11:57556509:57556627:+	included	ENSG00000198561	11:57549074:57564056:+	CTNND1
11:62388527:62388765:-	included	ENSG00000149541	11:62382767:62389647:-	B3GAT3
11:66433743:66433964:-	included	ENSG00000173914	11:66432924:66433966:-	RBM4B
11:70197100:70197129:+	included	ENSG00000131626	11:70192020:70201818:+	PPFIA1
11:71809335:71809461:-	included	ENSG00000149357	11:71808337:71810304:-	LAMTOR1
11:74570203:74570328:-	included	ENSG00000166435	11:74554014:74575159:-	XRRA1
11:75112684:75113490:+	included	ENSG00000149273	11:75110529:75117957:+	RPS3
11:76798988:76799107:+	included	ENSG00000149260	11:76778017:76804853:+	CAPN5
11:102080248:102080295:+	included	ENSG00000137693	11:101981191:102104154:+	YAP1
11:103270391:103270600:+	included	ENSG00000187240	11:102980159:103350591:+	DYNC2H1
11:105961284:105961405:+	included	ENSG00000149313	11:105946227:105962186:+	AASDHPPT
11:111624167:111624301:-	included	ENSG00000137713	11:111597631:111637106:-	PPP2R1B
11:112041346:112041397:+	included	ENSG00000254638	11:112035919:112043329:-	RP11-356J5.4
11:112100931:112100953:+	included	ENSG00000150787	11:112100816:112104121:+	PTS
11:113678745:113678858:-	included	ENSG00000048028	11:113675649:113679897:-	USP28
11:125526101:125526230:+	included	ENSG00000149554	11:125495868:125546150:+	CHEK1
11:126159560:126159728:+	included	ENSG00000150455	11:126152959:126161464:+	TIRAP
11:126161168:126161464:+	included	ENSG00000150455	11:126160750:126168740:+	TIRAP
11:130104051:130104168:-	included	ENSG00000196323	11:130100873:130131784:-	ZBTB44
12:7075074:7075079:-	included	ENSG00000215021	12:7074489:7076738:-	PHB2
12:9072352:9072513:+	included	ENSG00000111752	12:9066491:9075287:+	PHC1
12:12841447:12841866:-	included	ENSG00000183150	12:12813994:12849121:-	GPR19
12:24366277:24366351:-	included	ENSG00000255864	12:24366189:24715524:-	RP11-444D3.1
12:27829997:27830029:+	included	ENSG00000110841	12:27677105:27844717:+	PPFIBP1
12:27950180:27950282:+	included	ENSG00000087448	12:27932952:27955973:+	KLHL42
12:31237517:31237603:+	included	ENSG00000013573	12:31231453:31242064:+	DDX11
12:31256256:31256329:+	included	ENSG00000013573	12:31255201:31256939:+	DDX11
12:48359065:48359128:+	included	ENSG00000134291	12:48357351:48362046:+	TMEM106C
12:49580093:49580197:-	included	ENSG00000167552	12:49578578:49583107:-	TUBA1A
12:49741427:49741888:+	included	ENSG00000178401	12:49740699:49745958:+	DNAJC22
12:49835513:49835599:+	included	ENSG00000123352	12:49760366:49888554:+	SPATS2
12:51447595:51447643:+	included	ENSG00000050426	12:51441744:51451870:+	LETMD1
12:51449618:51449804:+	included	ENSG00000050426	12:51449481:51450312:+	LETMD1

Table S1. Exons affected by SWI/SNF ATPase subunits

BRM-mut

Affected exon coordinates	included/skipped	ENSEMBL gene ID	gene coordinates	Gene name
12:53402381:53402427:+	included	ENSG00000063046	12:53399941:53435993:+	EIF4B
12:54675579:54675725:+	included	ENSG00000135486	12:54673976:54680871:+	HNRNPA1
12:56676644:56676775:-	included	ENSG00000062485	12:56676120:56677605:-	CS
12:65081996:65082233:+	included	ENSG00000153179	12:65004292:65088962:+	RASSF3
12:69044180:69044248:+	included	ENSG00000127314	12:69044023:69050193:+	RAP1B
12:69279572:69279665:-	included	ENSG00000135678	12:69244954:69326979:-	CPM
12:70687851:70688074:+	included	ENSG00000111596	12:70671911:70726626:+	CNOT2
12:70726547:70726626:+	included	ENSG00000111596	12:70721286:70729246:+	CNOT2
12:94829051:94829103:-	included	ENSG00000173588	12:94806259:94853764:-	CCDC41
12:95456626:95456663:-	included	ENSG00000120798	12:95453695:95467391:-	NR2C1
12:95650326:95650398:+	included	ENSG00000028203	12:95611521:95650957:+	VEZT
12:95650926:95651015:+	included	ENSG00000028203	12:95611521:95650957:+	VEZT
12:97311399:97311515:+	included	ENSG00000139350	12:97301000:97347129:+	NEDD1
12:111079347:111079432:+	included	ENSG00000204852	12:111073471:111079391:+	TCTN1
12:120636357:120636434:-	included	ENSG00000089157	12:120636160:120638635:-	RPLP0
12:120653363:120653464:-	included	ENSG00000089159	12:120652638:120653809:-	PXN
12:123462528:123462746:+	included	ENSG00000111325	12:123459126:123464589:+	OGFOD2
12:123842356:123842399:-	included	ENSG00000139697	12:123779076:123849390:-	SBNO1
13:41376271:41376436:+	included	ENSG00000102743	13:41363547:41382606:+	SLC25A15
13:52709865:52709999:-	included	ENSG00000136098	13:52706774:52733695:-	NEK3
13:77692475:77692654:-	included	ENSG00000005810	13:77672202:77700651:-	MYCBP2
13:111296412:111296529:-	included	ENSG00000134905	13:111293758:111316208:-	CARS2
14:21491023:21491064:-	included	ENSG00000165795	14:21484921:21493982:-	NDRG2
14:21969055:21969271:-	included	ENSG00000165819	14:21966276:21971972:-	METTL3
14:23377542:23377608:-	included	ENSG00000100461	14:23369853:23388393:-	RBM23
14:24656873:24657019:-	included	ENSG00000196497	14:24656696:24657275:-	IPO4
14:31388172:31388312:-	included	ENSG00000196792	14:31381271:31401604:-	STRN3
14:50300141:50300214:-	included	ENSG00000165525	14:50249996:50319921:-	NEMF
14:53327732:53327752:-	included	ENSG00000073712	14:53323989:53328021:-	FERMT2
14:53518562:53518645:-	included	ENSG00000100523	14:53515265:53519804:-	DDHD1
14:55657787:55657894:-	included	ENSG00000126787	14:55614829:55658262:-	DLGAP5
14:58755436:58755531:-	included	ENSG00000268466	14:58754750:58755865:+	AL132989.1
14:63920407:63920603:-	included	ENSG00000154001	14:63851248:63920494:-	PPP2R5E
14:65544046:65544146:-	included	ENSG00000125952	14:65541841:65569222:-	MAX
14:68271934:68272022:-	included	ENSG00000072121	14:68213236:68283306:-	ZFYVE26
14:74361062:74361112:+	included	ENSG00000119725	14:74353436:74364803:+	ZNF410
14:74759857:74760013:-	included	ENSG00000119688	14:74752125:74769759:-	ABCD4
14:77566227:77566304:+	included	ENSG00000198894	14:77564600:77572433:+	CIPC
14:95600698:95600840:-	included	ENSG00000100697	14:95552564:95624347:-	DICER1
14:102698872:102699008:-	included	ENSG00000080823	14:102695163:102699062:-	MOK
14:104196130:104196183:+	included	ENSG00000100711	14:104190845:104200001:+	ZFYVE21
15:23029840:23029966:-	included	ENSG00000140157	15:23019818:23034378:-	NIPA2
15:28878736:28878886:+	included	ENSG00000206149	15:28878244:28887539:+	HERC2P9
15:42121302:42121490:+	included	ENSG00000243708	15:42120282:42140345:+	PLA2G4B
15:43164809:43164956:-	included	ENSG00000128881	15:43147316:43211986:-	TTBK2
15:49912331:49912387:-	included	ENSG00000166262	15:49906988:49913013:-	FAM227B
15:52861045:52861099:-	included	ENSG00000128989	15:52839241:52861436:-	ARPP19
15:60770767:60770859:-	included	ENSG00000128915	15:60711807:60771344:-	NARG2
15:63516047:63516178:+	included	ENSG00000166128	15:63481667:63559981:+	RAB8B
15:69745986:69747626:+	included	ENSG00000137818	15:69745122:69748255:+	RPLP1
15:74979432:74979520:-	included	ENSG00000179151	15:74979470:74988042:-	EDC3
15:82769344:82769439:-	included	ENSG00000188384	15:82756850:82769399:+	CSPG4P8

Table S1. Exons affected by SWI/SNF ATPase subunits

BRM-mut

Affected exon coordinates	included/skipped	ENSEMBL gene ID	gene coordinates	Gene name
15:82769402:82769439:-	included	ENSG00000255769	15:82768638:82770067:-	RP11-152F13.3
15:91485667:91485835:+	included	ENSG00000140553	15:91478514:91486314:+	UNC45A
15:101827861:101827907:-	included	ENSG00000131876	15:101826814:101835418:-	SNRPA1
16:258600:258663:-	included	ENSG00000007392	16:239096:259772:-	LUC7L
16:1876508:1876603:-	included	ENSG00000063854	16:1868515:1876803:-	HAGH
16:2013654:2014366:-	included	ENSG00000140988	16:2012052:2014861:-	RPS2
16:18466435:18466711:-	included	ENSG00000233024	16:18451942:18468926:-	NPIPA7
16:21863091:21863367:-	included	ENSG00000185864	16:21858802:21869003:-	NPIPB4
16:21863236:21863367:-	included	ENSG00000185864	16:21858802:21869003:-	NPIPB4
16:22530459:22530735:+	included	ENSG00000243716	16:22516171:22538951:+	NPIPB5
16:23619185:23619333:-	included	ENSG00000083093	16:23614487:23632710:-	PALB2
16:28903636:28903980:+	included	ENSG00000196296	16:28889725:28915785:+	ATP2A1
16:29095764:29095853:+	included	ENSG00000260908	16:29065052:29118766:-	CTB-134H23.3
16:29511319:29511595:-	included	ENSG00000169203	16:29496010:29517141:-	RP11-231C14.4
16:29511464:29511595:-	included	ENSG00000169203	16:29496010:29517141:-	RP11-231C14.4
16:30005227:30006164:-	included	ENSG00000149929	16:30003644:30007757:-	HIRIP3
16:30005969:30006164:-	included	ENSG00000149929	16:30003644:30007757:-	HIRIP3
16:30012533:30015978:+	included	ENSG00000169592	16:30007564:30012896:+	INO80E
16:32776759:32776866:+	included	ENSG00000260644	16:32772263:32782919:+	HERC2P5
16:48585297:48585393:-	included	ENSG00000102921	16:48580092:48587564:-	N4BP1
16:66860571:66860683:-	included	ENSG00000159593	16:66850516:66864871:-	NAE1
16:66861883:66862045:-	included	ENSG00000159593	16:66850516:66864871:-	NAE1
16:67291434:67291504:+	included	ENSG00000135740	16:67271585:67306093:+	SLC9A5
16:67858486:67858682:+	included	ENSG00000102904	16:67840667:67861917:+	TSNAXIP1
16:70165205:70165322:+	included	ENSG00000090857	16:70147528:70195184:+	PDPR
16:74383640:74383757:-	included	ENSG00000214331	16:74366299:74402059:-	RP11-252A24.2
16:81121206:81121269:-	included	ENSG00000140905	16:81117850:81129923:-	GCSH
17:5329291:5331531:+	included	ENSG00000263272	17:5328458:5336196:-	CTC-524C5.2
17:5391516:5391909:+	included	ENSG00000167842	17:5389604:5392557:+	MIS12
17:16342842:16343017:+	included	ENSG00000175061	17:16342288:16345052:+	FAM211A-AS1
17:16342974:16343017:+	included	ENSG00000175061	17:16342288:16345052:+	FAM211A-AS1
17:17924437:17924552:-	included	ENSG00000171953	17:17880722:17942482:-	ATPAF2
17:19835118:19835291:-	included	ENSG00000108599	17:19807614:19881150:-	AKAP10
17:27043011:27043079:-	included	ENSG00000109113	17:27041298:27045286:-	RAB34
17:27253800:27253891:-	included	ENSG00000109118	17:27248812:27255308:-	PHF12
17:29253827:29253936:+	included	ENSG00000184060	17:29248697:29285757:+	ADAP2
17:30678484:30678648:+	included	ENSG00000010244	17:30677926:30685653:+	ZNF207
17:37855790:37855840:+	included	ENSG00000141736	17:37844371:37873910:+	ERBB2
17:40814991:40815070:+	included	ENSG00000037042	17:40811322:40819024:+	TUBG2
17:42265044:42265111:+	included	ENSG00000168591	17:42264337:42268594:+	TMUB2
17:46926614:46926739:+	included	ENSG00000136436	17:46925570:46930397:+	CALCOCO2
17:49231586:49231805:+	included	ENSG00000239672	17:49230932:49233907:+	NME1
17:57208642:57208728:-	included	ENSG00000182628	17:57187311:57232630:-	SKA2
17:57764362:57764382:+	included	ENSG00000141367	17:57762477:57764508:+	CLTC
17:58037429:58037529:-	included	ENSG00000189050	17:58033848:58039960:-	RNFT1
17:71087963:71088255:-	included	ENSG00000133195	17:71080973:71088851:-	SLC39A11
17:79530439:79530594:-	included	ENSG00000182446	17:79525814:79533628:-	NPLOC4
17:80430440:80430574:+	included	ENSG00000141562	17:80416060:80446131:+	NARF
18:9204474:9204542:+	included	ENSG00000101745	18:9182379:9211619:+	ANKRD12
18:23615795:23615887:-	included	ENSG00000141380	18:23596577:23670589:-	SS18
18:23664016:23664139:-	included	ENSG00000141380	18:23660334:23670064:-	SS18
18:47014870:47014936:-	included	ENSG00000215472	18:47008050:47017956:-	RPL17-C18orf32

Table S1. Exons affected by SWI/SNF ATPase subunits

BRM-mut

Affected exon coordinates	included/skipped	ENSEMBL gene ID	gene coordinates	Gene name
19:3542774:3542975:-	included	ENSG00000183397	19:3539151:3544028:+	C19orf71
19:15540397:15540900:-	included	ENSG00000011451	19:15532333:15544101:-	WIZ
19:45162010:45162033:+	included	ENSG00000073008	19:45147097:45166850:+	PVR
19:47880172:47880246:+	included	ENSG00000134815	19:47876053:47885536:+	DHX34
19:49129148:49129619:+	included	ENSG00000063176	19:49122547:49133030:+	SPHK2
19:50430951:50431105:-	included	ENSG00000104951	19:50392910:50432796:-	IL4I1
19:54042470:54042536:+	included	ENSG00000130844	19:54041398:54042778:+	ZNF331
19:57864023:57864103:+	included	ENSG00000131845	19:57862674:57871266:+	ZNF304
19:57987028:57987154:-	included	ENSG00000197128	19:57984412:57988900:-	ZNF772
2:25061716:25061781:-	included	ENSG00000138031	2:25057794:25063472:-	ADCY3
2:27258462:27258596:+	included	ENSG00000119777	2:27258387:27260532:+	TMEM214
2:37469778:37469836:+	included	ENSG00000003509	2:37458773:37474719:+	NDUFAF7
2:62115159:62115392:-	included	ENSG00000115484	2:62115082:62115593:-	CCT4
2:64069673:64069733:+	included	ENSG00000169764	2:64068087:64083567:+	UGP2
2:70215874:70215970:-	included	ENSG00000179818	2:70189901:70223973:-	PCBP1-AS1
2:73198699:73198814:-	included	ENSG00000144040	2:73171731:73208246:-	SFXN5
2:73961655:73961718:-	included	ENSG00000144034	2:73957390:73964467:-	TPRKB
2:74617710:74617855:-	included	ENSG00000237737	2:74612844:74621009:+	DCTN1-AS1
2:80846210:80846353:+	included	ENSG00000066032	2:79412356:80875904:+	CTNNA2
2:85571122:85571288:-	included	ENSG00000042445	2:85570815:85573476:-	RETSAT
2:86078387:86078826:-	included	ENSG00000115525	2:86078542:86080236:-	ST3GAL5
2:86380683:86380697:-	included	ENSG00000132305	2:86371054:86422893:-	IMMT
2:122159444:122159446:-	included	ENSG00000074054	2:122095351:122407163:-	CLASP1
2:135010666:135010789:+	included	ENSG00000152127	2:135010674:135012085:+	MGAT5
2:170425694:170425762:-	included	ENSG00000138399	2:170417039:170427749:-	FASTKD1
2:174223983:174224219:+	included	ENSG00000144354	2:174219547:174233632:+	CDCA7
2:179095153:179095240:+	included	ENSG00000079156	2:179059215:179149765:+	OSBPL6
2:179988442:179988556:-	included	ENSG00000187231	2:179974048:179989194:-	SESTD1
2:190575775:190575879:+	included	ENSG00000151687	2:190539015:190625919:+	ANKAR
2:203075458:203075529:-	included	ENSG00000116030	2:203070902:203103331:-	SUMO1
2:204137369:204137471:+	included	ENSG00000119004	2:204103662:204162068:+	CYP20A1
2:210561266:210561472:+	included	ENSG00000078018	2:210517894:210595121:+	MAP2
2:216975190:216975274:+	included	ENSG00000079246	2:216974053:216983862:+	XRCC5
2:219524760:219524968:+	included	ENSG00000074582	2:219523486:219525940:+	BCS1L
2:233124551:233125395:+	included	ENSG00000144535	2:233114049:233172488:+	DIS3L2
20:25490306:25490425:-	included	ENSG00000101004	20:25433340:25566153:-	NINL
20:25655533:25655714:+	included	ENSG00000213742	20:25604680:25658710:+	ZNF337-AS1
20:30507667:30507735:+	included	ENSG00000131044	20:30458504:30532764:+	TTLL9
20:32652850:32652966:+	included	ENSG00000125970	20:32581451:32668077:+	RALY
20:34087878:34087998:+	included	ENSG00000126001	20:34085637:34090632:+	CEP250
20:35930683:35930726:+	included	ENSG00000101363	20:35918040:35945663:+	MANBAL
20:42087793:42088060:+	included	ENSG00000124193	20:42086567:42089974:+	SRSF6
20:42159438:42159529:+	included	ENSG00000185513	20:42157296:42163043:+	L3MBTL1
20:48700666:48700791:-	included	ENSG00000244687	20:48697660:48732491:-	UBE2V1
20:49457074:49457467:+	included	ENSG00000230043	20:49457151:49457286:-	TMSB4XP6
20:57234679:57234690:+	included	ENSG00000124222	20:57226327:57254582:+	STX16
21:18924067:18924271:+	included	ENSG00000154639	21:18884699:18942418:+	CXADR
21:34955794:34955972:-	included	ENSG00000159147	21:34947782:34960976:-	DONSON
21:38466305:38466390:+	included	ENSG00000182670	21:38463626:38469185:+	TTC3
22:19868379:19868468:-	included	ENSG00000184470	22:19863044:19872027:-	TXNRD2
22:28336008:28336159:+	included	ENSG00000235954	22:28315363:28389280:+	TTC28-AS1
22:29099493:29099554:-	included	ENSG00000183765	22:29083730:29107919:-	CHEK2

Table S1. Exons affected by SWI/SNF ATPase subunits

BRM-mut

Affected exon coordinates	included/skipped	ENSEMBL gene ID	gene coordinates	Gene name
22:29684595:29687588:+	included	ENSG00000182944	22:29663997:29696514:+	EWSR1
22:30078968:30079053:+	included	ENSG00000186575	22:29999544:30094583:+	NF2
22:30229530:30229603:-	included	ENSG00000100325	22:30184596:30234263:-	ASCC2
22:32758959:32759045:-	included	ENSG00000205853	22:32755892:32766972:-	RFPL3S
22:38463711:38463777:+	included	ENSG00000100151	22:38453401:38464121:+	PICK1
22:41213207:41213381:-	included	ENSG00000100372	22:41165633:41215386:-	SLC25A17
22:41609634:41610707:-	included	ENSG00000100395	22:41601305:41613177:+	L3MBTL2
22:44168772:44168983:-	included	ENSG00000186976	22:44073870:44208117:-	EFCAB6
22:45567690:45567918:+	included	ENSG00000093000	22:45564046:45574781:+	NUP50
22:46677868:46678048:+	included	ENSG00000075234	22:46663860:46689905:+	TTC38
3:4354582:4355445:+	included	ENSG00000170364	3:4345044:4356086:+	SETMAR
3:9807443:9807574:-	included	ENSG00000134072	3:9799025:9811661:-	CAMK1
3:9985586:9985779:+	included	ENSG00000163703	3:9982807:9986107:+	CRELD1
3:14160645:14160813:-	included	ENSG00000163528	3:14153579:14166370:-	CHCHD4
3:15252309:15252429:+	included	ENSG00000131375	3:15247658:15259036:+	CAPN7
3:17603511:17603634:-	included	ENSG00000131374	3:17198653:17782399:-	TBC1D5
3:17617906:17618041:-	included	ENSG00000131374	3:17198653:17782399:-	TBC1D5
3:20216497:20216547:-	included	ENSG00000129810	3:20202084:20227683:-	SGOL1
3:29941196:29941246:+	included	ENSG00000144642	3:29804413:29944729:+	RBMS3
3:31871532:31871723:-	included	ENSG00000144645	3:31699381:32022794:-	OSBPL10
3:32027740:32027901:+	included	ENSG00000197385	3:32023262:32030417:+	ZNF860
3:37089010:37089174:+	included	ENSG00000076242	3:37034822:37092409:+	MLH1
3:49524687:49524848:+	included	ENSG00000173402	3:49507734:49524848:+	DAG1
3:52028789:52028873:-	included	ENSG00000162244	3:52027615:52029958:-	RPL29
3:52456238:52456354:+	included	ENSG00000010318	3:52445212:52457656:+	PHF7
3:52783708:52783845:-	included	ENSG00000114904	3:52780165:52786086:-	NEK4
3:71823596:71823658:-	included	ENSG00000163421	3:71820806:71834357:-	PROK2
3:78696779:78696805:-	included	ENSG00000169855	3:78655957:78719690:-	ROBO1
3:100289453:100293503:+	included	ENSG00000181458	3:100287665:100289758:+	TMEM45A
3:107768466:107768498:-	included	ENSG00000196776	3:107762144:107773586:-	CD47
3:113146034:113146186:-	included	ENSG00000243849	3:113122844:113152839:+	WDR52-AS1
3:113801810:113801850:+	included	ENSG00000151576	3:113775581:113807196:+	QTRTD1
3:119176865:119177031:-	included	ENSG00000176142	3:119148346:119182444:-	TMEM39A
3:137790485:137790677:-	included	ENSG00000158163	3:137780831:137834451:-	DZIP1L
3:138488893:138488959:-	included	ENSG00000051382	3:138478016:138489397:-	PIK3CB
3:140685190:140685276:+	included	ENSG00000114120	3:140660671:140698775:+	SLC25A36
3:141461486:141461749:+	included	ENSG00000114125	3:141457045:141466402:+	RNF7
3:169501518:169501646:+	included	ENSG00000085274	3:169497076:169502464:+	MYNN
3:181417386:181417671:+	included	ENSG00000242808	3:181417381:181417888:+	SOX2-OT
3:184640217:184640271:+	included	ENSG00000156931	3:184633170:184644434:+	VPS8
3:185155235:185155418:+	included	ENSG00000073803	3:185081029:185200389:+	MAP3K13
3:185649365:185649640:-	included	ENSG00000136527	3:185649302:185655812:-	TRA2B
3:195449372:195449689:+	included	ENSG00000176945	3:195447752:195460422:+	MUC20
3:195612284:195612414:-	included	ENSG00000061938	3:195611715:195619472:-	TNK2
3:197417945:197418019:-	included	ENSG00000145016	3:197398263:197476570:-	KIAA0226
4:366453:366796:+	included	ENSG00000131127	4:331602:366815:+	ZNF141
4:1956264:1956412:+	included	ENSG00000109685	4:1953831:1957885:+	WHSC1
4:2656785:2656856:+	included	ENSG00000125386	4:2627158:2734279:+	FAM193A
4:3430285:3430438:+	included	ENSG00000159788	4:3424261:3432174:+	RGS12
4:3956687:3956760:-	included	ENSG00000251669	4:3949631:3956770:-	FAM86EP
4:20702294:20702410:+	included	ENSG00000163138	4:20702060:20704317:+	PACRGL
4:20703133:20703290:+	included	ENSG00000163138	4:20702060:20704317:+	PACRGL

Table S1. Exons affected by SWI/SNF ATPase subunits

BRM-mut

Affected exon coordinates	included/skipped	ENSEMBL gene ID	gene coordinates	Gene name
4:20703764:20703844:+	included	ENSG00000163138	4:20702060:20704317:+	PACRGL
4:27009605:27009628:+	included	ENSG00000109689	4:27004621:27024462:+	STIM2
4:39528673:39528739:-	included	ENSG00000109814	4:39522971:39529218:-	UGDH
4:52714371:52714533:+	included	ENSG00000109184	4:52709165:52780460:+	DCUN1D4
4:54257597:54257665:+	included	ENSG00000145216	4:54243811:54325835:+	FIP1L1
4:76716489:76716509:+	included	ENSG00000138768	4:76678649:76735366:+	USO1
4:89597369:89597392:+	included	ENSG00000138641	4:89526944:89629686:+	HERC3
4:89912124:89912301:-	included	ENSG00000138640	4:89647105:89978323:-	FAM13A
4:121652773:121652945:-	included	ENSG00000138738	4:121613069:121844004:-	PRDM5
4:152021637:152021740:+	included	ENSG00000145425	4:152020724:152025804:+	RPS3A
4:152065372:152065440:-	included	ENSG00000109686	4:152058884:152065556:-	SH3D19
4:159133213:159133372:+	included	ENSG00000164124	4:159122755:159136465:+	TMEM144
4:159601680:159601759:+	included	ENSG00000171503	4:159593276:159630775:+	ETFDH
4:159754953:159755042:+	included	ENSG00000052795	4:159690345:159757810:+	FNIP2
4:170926731:170927161:-	included	ENSG00000198948	4:170926809:170928126:-	MFAP3L
4:186357438:186357513:+	included	ENSG00000205129	4:186347402:186357513:+	C4orf47
5:884053:884185:-	included	ENSG00000028310	5:863850:889814:-	BRD9
5:65451893:65454760:+	included	ENSG00000153914	5:65454527:65458064:+	SREK1
5:70092028:70092120:+	included	ENSG00000253366	5:70091601:70149508:+	RP11-589F5.4
5:70240485:70240580:+	included	ENSG00000172062	5:70238298:70242105:+	SMN1
5:74650881:74651039:+	included	ENSG00000113161	5:74650132:74650991:+	HMGCR
5:133642300:133642393:-	included	ENSG00000006837	5:133541304:133706734:-	CDKL3
5:133644265:133644418:-	included	ENSG00000006837	5:133643691:133655080:-	CDKL3
5:133912458:133912586:+	included	ENSG00000043143	5:133909446:133915165:+	JADE2
5:138655023:138655190:+	included	ENSG00000015479	5:138643783:138657706:+	MATR3
5:139851824:139851880:+	included	ENSG00000131503	5:139781438:139852062:+	ANKHD1
5:151171606:151171762:+	included	ENSG00000145907	5:151151470:151192346:+	G3BP1
5:159535552:159535621:-	included	ENSG00000170234	5:159518348:159546430:-	PWWP2A
5:175786465:175786570:-	included	ENSG00000122203	5:175786522:175788767:-	KIAA1191
5:176958954:176959201:-	included	ENSG00000146067	5:176958338:176959613:-	FAM193B
5:177658670:177658867:-	included	ENSG00000175309	5:177653466:177659542:-	PHYKPL
5:179035286:179035466:+	included	ENSG00000176783	5:179033903:179036473:+	RUFY1
5:179274978:179275066:-	included	ENSG00000161010	5:179274356:179285808:-	C5orf45
6:10755375:10755465:+	included	ENSG00000137210	6:10747991:10757214:+	TMEM14B
6:13974441:13974595:+	included	ENSG00000180537	6:13924676:13977503:+	RNF182
6:17790103:17790141:-	included	ENSG00000137177	6:17759484:17794928:-	KIF13A
6:20535318:20535430:+	included	ENSG00000145996	6:20534687:21232635:+	CDKAL1
6:26091542:26091817:+	included	ENSG00000010704	6:26087508:26095445:+	HFE
6:28239632:28240564:+	included	ENSG00000197062	6:28234787:28245974:+	RP5-874C20.3
6:28297080:28297555:-	included	ENSG00000235109	6:28295224:28303228:-	ZSCAN31
6:35389597:35390160:+	included	ENSG00000112033	6:35310334:35395968:+	PPARD
6:39077090:39081496:-	included	ENSG00000217165	6:39078096:39080728:+	ANKRD18EP
6:54001513:54003084:+	included	ENSG00000146147	6:53964362:54001623:+	MLIP
6:56333780:56333797:-	included	ENSG00000151914	6:56322857:56334754:-	DST
6:56482784:56485513:-	included	ENSG00000151914	6:56322784:56507794:-	DST
6:58276137:58276289:-	included	ENSG00000215190	6:58272360:58276677:-	LINC00680
6:83806450:83806542:+	included	ENSG00000083097	6:83777384:83878127:+	DOPEY1
6:88304245:88304331:+	included	ENSG00000135336	6:88299847:88305155:+	ORC3
6:99365250:99365595:-	included	ENSG00000112234	6:99316419:99395802:-	FBXL4
6:135371720:135371785:-	included	ENSG00000112339	6:135281515:135376036:-	HBS1L
6:138763120:138763251:-	included	ENSG00000135540	6:138743179:138893677:-	NHSL1
7:6052310:6052771:+	included	ENSG00000106305	7:6048920:6054882:+	AIMP2

Table S1. Exons affected by SWI/SNF ATPase subunits

BRM-mut

Affected exon coordinates	included/skipped	ENSEMBL gene ID	gene coordinates	Gene name
7:12727260:12727353:+	included	ENSG00000122644	7:12726480:12728804:+	ARL4A
7:42966982:42967058:-	included	ENSG00000256646	7:42948871:42971773:-	PSMA2
7:45765783:45765835:-	included	ENSG00000214765	7:45763378:45767909:-	SEPT7P2
7:64532523:64532625:+	included	ENSG00000234585	7:64525376:64534226:+	CCT6P3
7:65226633:65226735:+	included	ENSG00000228409	7:65216128:65228323:+	CCT6P1
7:72510219:72510331:+	included	ENSG00000174384	7:72507940:72515008:+	RP11-313P13.4
7:73101329:73101425:+	included	ENSG00000071462	7:73097898:73101425:+	WBSCR22
7:74982537:74982649:-	included	ENSG00000174368	7:74981056:74984931:-	PMS2P2
7:77567027:77567155:+	included	ENSG00000006576	7:77469446:77586818:+	PHTF2
7:86540938:86541011:-	included	ENSG00000164659	7:86509331:86574362:-	KIAA1324L
7:86783706:86783844:+	included	ENSG00000135164	7:86783772:86784875:+	DMTF1
7:87815930:87816016:+	included	ENSG00000008277	7:87563457:87832204:+	ADAM22
7:90041321:90041363:+	included	ENSG00000157224	7:90032666:90042725:+	CLDN12
7:91506192:91506292:-	included	ENSG00000127989	7:91506208:91509753:-	MTERF
7:100451778:100452000:+	included	ENSG00000146828	7:100450340:100462980:+	SLC12A9
7:100861600:100865077:+	included	ENSG00000106397	7:100860703:100861647:-	PLOD3
7:100887288:100887420:-	included	ENSG00000214253	7:100882738:100888356:-	FIS1
7:100962237:100962344:-	included	ENSG00000128581	7:100958352:100965104:-	RABL5
7:102207029:102207183:-	included	ENSG00000168255	7:102206322:102213030:-	POLR2J3
7:106897177:106897239:-	included	ENSG00000164597	7:106876999:106899065:-	COG5
7:108161920:108161965:-	included	ENSG00000135241	7:108155357:108166567:-	PNPLA8
7:110739937:110740018:+	included	ENSG00000184903	7:110303356:111030806:-	IMMP2L
8:180804:180907:-	included	ENSG00000223508	8:163185:182211:-	RPL23AP53
8:11660831:11661027:+	included	ENSG00000079459	8:11660276:11661341:+	FDFT1
8:22396982:22397011:+	included	ENSG00000120910	8:22298595:22398638:+	PPP3CC
8:28719557:28719729:-	included	ENSG00000104299	8:28707525:28747495:-	INTS9
8:28906807:28906911:+	included	ENSG00000147421	8:28903816:28908171:+	HMBOX1
8:67478299:67478478:-	included	ENSG00000185697	8:67477367:67479193:-	MYBL1
8:74872000:74872053:-	included	ENSG00000154582	8:74851403:74884421:-	TCEB1
8:91643780:91643935:-	included	ENSG00000180694	8:91634222:91658311:-	TMEM64
8:95746858:95746982:+	included	ENSG00000156162	8:95731930:95752007:+	DPY19L4
8:96046236:96046350:+	included	ENSG00000156170	8:95970235:96070938:+	NDUFAF6
8:117746516:117746563:-	included	ENSG00000147677	8:117738293:117779164:-	EIF3H
8:128867401:128867565:+	included	ENSG00000249859	8:128866973:128902987:+	PVT1
8:146221893:146221993:-	included	ENSG00000196922	8:146198974:146228280:-	ZNF252P
9:177723:177820:-	included	ENSG00000172785	9:171952:179058:-	CBWD1
9:13115247:13115333:-	included	ENSG00000107186	9:13115319:13123270:-	MPDZ
9:32988081:32988127:-	included	ENSG00000137074	9:32985986:32988132:-	APTX
9:36581641:36581736:+	included	ENSG00000165304	9:36572858:36677503:+	MELK
9:94877204:94877348:-	included	ENSG00000090054	9:94870082:94877664:-	SPTLC1
9:100673200:100675257:-	included	ENSG00000136932	9:100666815:100674753:-	C9orf156
9:103191788:103191944:+	included	ENSG00000066697	9:103189437:103213511:+	MSANTD3
9:108336479:108336613:+	included	ENSG00000106692	9:108320421:108359011:+	FKTN
9:116093264:116093396:-	included	ENSG00000148225	9:116075501:116102562:-	WDR31
9:130924283:130925231:+	included	ENSG00000171159	9:130922538:130926207:+	C9orf16
9:133506548:133506680:+	included	ENSG00000107164	9:133454992:133513739:+	FUBP3
X:47509320:47509425:-	included	ENSG00000126767	X:47508285:47510003:-	ELK1
X:80457936:80458075:+	included	ENSG00000131171	X:80457441:80554046:+	SH3BGRL
X:83415296:83415443:-	included	ENSG00000072133	X:83411082:83442906:-	RPS6KA6
X:96018099:96018119:+	included	ENSG00000147202	X:95939661:96855597:+	DIAPH2
X:107370023:107370324:+	included	ENSG00000101844	X:107369306:107395088:+	ATG4A
X:129484620:129484705:+	included	ENSG00000102078	X:129483221:129499010:+	SLC25A14

Table S1. Exons affected by SWI/SNF ATPase subunits

BRM-mut

**Affected exon coordinates**	**included/skipped**	**ENSEMBL gene ID**	**gene coordinates**	**Gene name**
X:133906173:133906313:-	included	ENSG00000156504	X:133903595:133931164:-	FAM122B
X:148608475:148608607:-	included	ENSG00000010404	X:148564073:148615470:-	IDS
X:149102595:149102791:-	included	ENSG00000197021	X:149097744:149106653:-	CXorf40B
X:151304462:151304552:-	included	ENSG00000266560	X:151283465:151306965:-	RP11-1007I13.4
X:151883336:151883646:+	included	ENSG00000183305	X:151883081:151885563:+	MAGEA2B
X:151883568:151883646:+	included	ENSG00000183305	X:151883081:151885563:+	MAGEA2B
X:151885137:151885234:+	included	ENSG00000183305	X:151883081:151885563:+	MAGEA2B
X:152862880:152863164:-	included	ENSG00000147382	X:152853376:152864641:-	FAM58A
X:154300602:154300618:+	included	ENSG00000214827	X:154293437:154376212:-	MTCP1

Table S2. Genes affected by expression of SWI/SNF ATPase subunits at gene expression level (DESeq2)

		BRG1-wt		BRG1-mut		BRM-wt		BRM-mut	
	Number of genes affected ->	255		658		101		2	
**Name**	**GeneID**	**log2 (FC)**	**P-adj**	**log2 (FC)**	**P-adj**	**log2 (FC)**	**P-adj**	**log2 (FC)**	**P-adj**
KRT80	ENSG00000167767	3.186481	1.0697E-61			2.45202982	2.0566E-14		
SMARCA4	ENSG00000127616	2.77036159	2.1401E-57	3.64350764	3.0167E-50				
UCA1	ENSG00000214049	2.64649374	6.66E-53			2.5169836	1.8081E-17		
CPA4	ENSG00000128510	2.79549106	2.977E-52			2.28084434	1.8869E-12		
S100A16	ENSG00000188643	2.79700225	5.5447E-51			1.7214803	7.3482E-07		
SERPINE1	ENSG00000106366	1.83184617	4.9305E-31	0.70756553	0.03957835	1.49564234	1.2473E-05		
SPARC	ENSG00000113140	2.25586079	2.341E-29			2.65033978	1.8452E-17		
HSPA6	ENSG00000173110	2.19328793	6.3785E-28	2.8377109	1.517E-13			0.6176402	0.0132892
KRT8	ENSG00000170421	2.0334101	2.4836E-27			1.17979127	0.00679978		
MMP1	ENSG00000196611	1.95778238	6.4456E-27			1.84497656	4.306E-08		
MATN2	ENSG00000132561	1.65879861	6.3055E-25			1.61359915	3.3674E-08		
F3	ENSG00000117525	1.77668771	4.2057E-23			1.84469202	2.0304E-08		
TAGLN	ENSG00000149591	1.7104314	1.6559E-21						
IFITM1	ENSG00000185885	1.8472324	4.6509E-20			1.39570732	9.1277E-05		
PRSS23	ENSG00000150687	1.43336586	1.4299E-19			1.5202992	7.3482E-07		
ANXA3	ENSG00000138772	1.80916884	3.9308E-19			1.89001696	2.257E-08		
PLSCR1	ENSG00000188313	1.81664424	3.9728E-19			1.22050238	0.00331311		
CD44	ENSG00000026508	1.73765331	2.015E-17			1.7868886	1.5317E-07		
DDX60	ENSG00000137628	1.70648572	1.6386E-16			1.1536306	0.00679978		
PHLDA1	ENSG00000139289	1.44609615	2.5746E-16			1.25814354	0.00266257		
RBP1	ENSG00000114115	1.63630821	4.9307E-16			1.41936285	0.00013082		
HAPLN3	ENSG00000140511	1.61328787	7.126E-16	1.11827759	0.01921132	1.12311249	0.01423905		
SLC7A8	ENSG00000092068	1.64692098	8.5068E-16			1.7021842	6.5899E-07		
MYOF	ENSG00000138119	1.15482062	1.3556E-15			1.18277646	0.00135388		
KLHDC8A	ENSG00000162873	1.64813959	2.2588E-15			1.88770442	1.7958E-08		
REC8	ENSG00000100918	1.52622811	3.8134E-15			1.07164159	0.01857928		
NIPAL1	ENSG00000163293	1.43988514	6.2382E-15			1.33743557	0.00059712		
ALPK2	ENSG00000198796	1.58931785	2.9782E-14			2.17133845	1.233E-11		
MYO10	ENSG00000145555	1.48959482	6.8497E-14			1.92801067	1.0098E-09		
HAS3	ENSG00000103044	1.31056344	1.4039E-13			1.37001619	2.4785E-05		
STRA6	ENSG00000137868	1.47379206	8.2056E-13			1.67144833	1.5553E-06		
MYO1B	ENSG00000128641	1.15443559	1.0554E-12			1.09333422	0.01123969		
S100A4	ENSG00000196154	1.48955955	1.0554E-12			1.62229772	1.0784E-06		
DDX58	ENSG00000107201	1.48137096	1.2117E-12			1.01426431	0.04305065		
LOXL2	ENSG00000134013	1.32320012	8.7853E-12						
PLEKHA4	ENSG00000105559	1.43845886	1.2136E-11	1.07875115	0.01921132	1.68459719	4.2074E-07		
STAT1	ENSG00000115415	1.36985913	1.2136E-11						
DHX58	ENSG00000108771	1.40581107	2.1046E-11			1.69930461	8.6629E-07		
CNN1	ENSG00000130176	1.42145073	2.6684E-11			1.21427263	0.00360621		
AHNAK2	ENSG00000185567	1.23053066	7.1388E-11			1.23617949	0.00030114		
IFI27	ENSG00000165949	1.37325984	7.8799E-11			1.30788926	0.00133729		
SYNM	ENSG00000182253	1.19225556	1.2203E-10			1.15180568	0.00214		
KRT18	ENSG00000111057	1.37169271	1.3594E-10			1.24949723	0.0028902		
MFGE8	ENSG00000140545	1.21260793	3.2047E-10	0.81876247	0.02919797				
PARP12	ENSG00000059378	1.3478416	3.2249E-10			1.18451982	0.00662842		
CDKN2B	ENSG00000147883	1.11117973	1.0606E-09			1.25210159	0.0001552		
CACNG4	ENSG00000075461	1.31382395	1.1797E-09			1.09253023	0.01334149		
ZNF114	ENSG00000178150	1.0945447	1.4809E-09			1.14220958	0.003466		
IGFBP4	ENSG00000141753	1.23415176	1.7211E-09			1.15990945	0.00271376		
IFIT5	ENSG00000152778	1.2389251	1.9608E-09						

Table S2. Genes affected by expression of SWI/SNF ATPase subunits at gene expression level (DESeq2)

		BRG1-wt		BRG1-mut		BRM-wt		BRM-mut	
	Number of genes affected ->	255		658		101		2	
**Name**	**GeneID**	**log2 (FC)**	**P-adj**	**log2 (FC)**	**P-adj**	**log2 (FC)**	**P-adj**	**log2 (FC)**	**P-adj**
FRMD4B	ENSG00000114541	1.23876209	2.0603E-09			1.58801253	5.3654E-06		
OLFML2A	ENSG00000185585	1.17159101	4.7341E-09						
IFI35	ENSG00000068079	1.25838553	4.9319E-09			1.6210781	1.5413E-06		
SP110	ENSG00000135899	1.26181108	6.5452E-09						
ATP8B1	ENSG00000081923	1.1231087	8.4584E-09			1.24720305	0.0020715		
RHOBTB3	ENSG00000164292	0.9324018	1.1131E-08			1.04926827	0.00919426		
HERC6	ENSG00000138642	1.19551122	2.6792E-08			1.08413925	0.02288591		
AHNAK	ENSG00000124942	0.86793931	3.3009E-08			0.97446822	0.01730171		
PHLDB2	ENSG00000144824	0.94402207	3.6157E-08						
BST2	ENSG00000130303	1.10010105	3.8538E-08			1.56064806	7.5504E-06		
LAMB1	ENSG00000091136	1.14521005	4.2291E-08			1.11415302	0.00662842		
DUSP10	ENSG00000143507	1.07466385	4.5712E-08			1.18905041	0.0011516		
ANK1	ENSG00000029534	1.15563581	5.8777E-08			1.51214979	3.3773E-05		
SH3BP5	ENSG00000131370	0.98713218	5.9917E-08			0.95758142	0.01901204		
AKAP12	ENSG00000131016	0.88084857	7.461E-08						
USP18	ENSG00000184979	1.19433528	7.461E-08			1.17597011	0.00707063		
HKDC1	ENSG00000156510	1.11649975	1.5355E-07			1.22021603	0.00266257		
PDP1	ENSG00000164951	0.84002655	1.7884E-07						
EIF2AK2	ENSG00000055332	1.00938528	1.9689E-07						
IQGAP2	ENSG00000145703	1.03550701	1.9689E-07						
LGALS3	ENSG00000131981	1.15938414	2.2263E-07			1.21702705	0.00326898		
TNIK	ENSG00000154310	0.95145719	2.5864E-07						
ARHGAP29	ENSG00000137962	1.07590001	3.3249E-07						
NLRC5	ENSG00000140853	1.10932967	3.5718E-07			1.43204158	0.00012472		
PLXND1	ENSG00000004399	0.97125886	8.4746E-07	0.99604469	0.00815618				
SHFL	ENSG00000130813	1.10304541	9.9227E-07			1.07605834	0.00953094		
TPM1	ENSG00000140416	0.87418312	1.0719E-06						
RIPOR2	ENSG00000111913	1.0257741	1.2201E-06			1.61716273	5.3947E-06		
ATP2B4	ENSG00000058668	0.87497415	1.9577E-06						
FSTL3	ENSG00000070404	1.03632201	1.9577E-06						
MCAM	ENSG00000076706	1.07296303	2.2924E-06						
SAMD9	ENSG00000205413	1.00511396	2.4446E-06						
CALB1	ENSG00000104327	1.07673191	2.6694E-06			1.18199567	0.00679978		
TRIM22	ENSG00000132274	0.9542257	2.8731E-06						
AXL	ENSG00000167601	1.06006799	4.5089E-06			1.03919158	0.03830203		
TNS3	ENSG00000136205	1.03978315	5.095E-06			1.23429763	0.00351963		
UBE2L6	ENSG00000156587	1.04389813	6.4672E-06						
TRIM21	ENSG00000132109	1.03425198	6.5112E-06						
NLRP1	ENSG00000091592	0.80931113	6.5415E-06	0.78354737	0.02939149				
IFITM3	ENSG00000142089	0.90138469	6.5611E-06			2.03126249	1.0098E-09		
APOBEC3C	ENSG00000244509	1.03648119	6.9208E-06			1.09213684	0.02114176		
TRIM25	ENSG00000121060	0.9225144	1.1182E-05						
GLIPR1	ENSG00000139278	0.9383465	1.1182E-05			1.20984144	0.00249356		
KRT8P3	ENSG00000254285	0.99367171	1.2929E-05						
STAT2	ENSG00000170581	0.96988417	1.7195E-05						
	ENSG00000260336	0.81948514	1.8474E-05						
TGFBI	ENSG00000120708	0.92726362	2.0176E-05			1.72370508	8.6629E-07		
ZC3HAV1	ENSG00000105939	0.86334726	2.0307E-05						
PDLIM1	ENSG00000107438	0.87444377	2.633E-05						
VSIR	ENSG00000107738	0.87541589	3.9828E-05						

Table S2. Genes affected by expression of SWI/SNF ATPase subunits at gene expression level (DESeq2)

		BRG1-wt		BRG1-mut		BRM-wt		BRM-mut	
	Number of genes affected ->	255		658		101		2	
**Name**	**GeneID**	**log2 (FC)**	**P-adj**	**log2 (FC)**	**P-adj**	**log2 (FC)**	**P-adj**	**log2 (FC)**	**P-adj**
IRF9	ENSG00000213928	0.96684327	4.4765E-05			1.07081478	0.01271427		
TDRD7	ENSG00000196116	0.87901947	5.0623E-05						
IFIT1	ENSG00000185745	0.88728812	5.4399E-05			1.2034373	0.00344815		
APOL6	ENSG00000221963	0.87325837	6.0752E-05			1.05986766	0.0193845		
NEDD9	ENSG00000111859	0.83840126	6.2547E-05			1.00563369	0.01583793		
S100A14	ENSG00000189334	0.83397503	7.4876E-05			0.98675129	0.02602778		
S100A2	ENSG00000196754	0.93147431	7.9986E-05						
HLA-B	ENSG00000234745	0.92785746	8.0663E-05						
CSF1	ENSG00000184371	0.94703326	9.2539E-05						
CHST2	ENSG00000175040	0.90260143	9.6446E-05						
TXK	ENSG00000074966	0.90626093	0.00012835			1.29793126	0.00151491		
APOL1	ENSG00000100342	0.78285264	0.00013568						
MMP24	ENSG00000125966	0.92414424	0.00014341						
NAV2	ENSG00000166833	0.92065957	0.00017706						
NMI	ENSG00000123609	0.90798315	0.00017895						
LMNA	ENSG00000160789	0.89460215	0.0001841	1.18204762	0.00910958				
LGALS1	ENSG00000100097	0.90611089	0.00020368	1.1076236	0.02471334				
LINC02167	ENSG00000261122	0.78996565	0.00022406			1.61522204	4.6781E-06		
PGM2L1	ENSG00000165434	0.72558162	0.00023065						
SLCO2A1	ENSG00000174640	0.81564186	0.00026501			1.4078713	0.00024225		
IFI44	ENSG00000137965	0.82626771	0.0003336						
APOL2	ENSG00000128335	0.82737186	0.00035084						
BATF2	ENSG00000168062	0.74711804	0.00036043						
RBM24	ENSG00000112183	0.88438478	0.00038254						
PLAUR	ENSG00000011422	0.88023522	0.00039882			1.08637379	0.02002492		
S100A13	ENSG00000189171	0.88300584	0.00039882						
TMSB4X	ENSG00000205542	0.82297958	0.00041708						
TRIM69	ENSG00000185880	0.81553826	0.00042657						
THBS1	ENSG00000137801	0.57878181	0.00045675						
PARP9	ENSG00000138496	0.77604566	0.00052969			1.27819146	0.00168263		
SCARA3	ENSG00000168077	0.7601733	0.00053604						
CABLES1	ENSG00000134508	0.68957189	0.00056314						
PSME1	ENSG00000092010	0.60950792	0.00071541						
OXTR	ENSG00000180914	0.73372194	0.00091199						
PARP10	ENSG00000178685	0.71198414	0.00098503						
EFEMP1	ENSG00000115380	0.8254874	0.0009872						
TRIM14	ENSG00000106785	0.78055088	0.00104353						
FLNB	ENSG00000136068	0.65332421	0.00104353						
DTX3L	ENSG00000163840	0.75369067	0.0010824			1.25476929	0.00249356		
TRANK1	ENSG00000168016	0.76291043	0.00112813						
PSTPIP2	ENSG00000152229	0.7271548	0.00126784						
IFI6	ENSG00000126709	0.67877133	0.00134295	1.11870588	0.03238562	2.38150115	2.0566E-14		
MMP2	ENSG00000087245	0.79990835	0.00139387						
GBP1	ENSG00000117228	0.70703255	0.00141797						
KCNN4	ENSG00000104783	0.75030904	0.00142723						
TNNT2	ENSG00000118194	0.66011119	0.00147803						
ARL4C	ENSG00000188042	0.81647937	0.00185374						
UBA7	ENSG00000182179	0.75883333	0.0018794						
PLAC8	ENSG00000145287	0.70287512	0.00204974						
TRIM56	ENSG00000169871	0.79188945	0.00271206						

Table S2. Genes affected by expression of SWI/SNF ATPase subunits at gene expression level (DESeq2)

		BRG1-wt		BRG1-mut		BRM-wt		BRM-mut	
	Number of genes affected ->	255		658		101		2	
**Name**	**GeneID**	**log2 (FC)**	**P-adj**	**log2 (FC)**	**P-adj**	**log2 (FC)**	**P-adj**	**log2 (FC)**	**P-adj**
IFFO2	ENSG00000169991	0.65062039	0.00271673						
PPL	ENSG00000118898	0.79132441	0.00304358						
NFASC	ENSG00000163531	0.66466278	0.00305482						
ABTB2	ENSG00000166016	0.79522472	0.00305482						
ENPP1	ENSG00000197594	0.62839509	0.00305482						
ZBED6CL	ENSG00000188707	0.69691865	0.00317945						
SMAD7	ENSG00000101665	0.74945186	0.0031799						
OAS1	ENSG00000089127	0.6085839	0.00347318			1.51660531	1.5748E-05		
FRAS1	ENSG00000138759	0.65470353	0.00352515						
FILIP1L	ENSG00000168386	0.78706511	0.00352515			1.15483971	0.00662842		
TAP1	ENSG00000168394	0.77930701	0.00352515						
ROR1	ENSG00000185483	0.71219654	0.00358109						
NPR1	ENSG00000169418	0.7586367	0.00396227						
LRP2	ENSG00000081479	0.72834135	0.00401197						
DKK3	ENSG00000050165	0.75138871	0.00437319						
METTL7A	ENSG00000185432	0.76672302	0.00475827			1.0512975	0.03142511		
PNPT1	ENSG00000138035	0.66851131	0.00488512						
FEZ1	ENSG00000149557	0.70295535	0.00512189						
APOBEC3B	ENSG00000179750	0.69833795	0.00534291						
UCP2	ENSG00000175567	0.57594297	0.00549902						
SVIL	ENSG00000197321	0.66415465	0.00551174						
LGALS3BP	ENSG00000108679	0.61909305	0.00557967	0.94258693	0.04082972	1.97828312	1.0098E-09		
EGF	ENSG00000138798	0.69460682	0.00562404						
SAMHD1	ENSG00000101347	0.65909789	0.00562855						
OAS3	ENSG00000111331	0.65076435	0.0056865			1.55955853	1.5748E-05		
TMSB4XP8	ENSG00000187653	0.68496907	0.00612669						
DOCK5	ENSG00000147459	0.59502844	0.00651098						
NID1	ENSG00000116962	0.53624574	0.00757644						
GEM	ENSG00000164949	0.70003832	0.00757644						
NDRG1	ENSG00000104419	0.74169347	0.008489						
CDA	ENSG00000158825	0.56319045	0.0085471						
PTPRH	ENSG00000080031	0.73585619	0.00985022						
OSR2	ENSG00000164920	0.71472679	0.00998092						
ZNFX1	ENSG00000124201	0.63049274	0.0101547						
MALT1	ENSG00000172175	0.50449305	0.01017191						
SGK1	ENSG00000118515	0.5135487	0.0104065						
SEL1L3	ENSG00000091490	0.53626843	0.01165083						
FLNC	ENSG00000128591	0.71275784	0.01196132						
S100A10	ENSG00000197747	0.49779288	0.01260052						
TRIM2	ENSG00000109654	0.49563997	0.01261674						
KCNH1	ENSG00000143473	0.71655745	0.01274507						
TIMP2	ENSG00000035862	0.5791462	0.01327331						
FGF1	ENSG00000113578	0.54286801	0.01327331						
ARHGEF3	ENSG00000163947	0.56590866	0.01327331						
VLDLR	ENSG00000147852	0.69429092	0.01340351						
IL11	ENSG00000095752	0.71276811	0.01392123						
	ENSG00000117289	0.70084225	0.01435895						
TLE3	ENSG00000140332	0.69301395	0.01435895						
PSMB9	ENSG00000240065	0.7037212	0.01498179						
LAP3	ENSG00000002549	0.57825392	0.01503879						

Table S2. Genes affected by expression of SWI/SNF ATPase subunits at gene expression level (DESeq2)

		BRG1-wt		BRG1-mut		BRM-wt		BRM-mut	
	Number of genes affected ->	255		658		101		2	
**Name**	**GeneID**	**log2 (FC)**	**P-adj**	**log2 (FC)**	**P-adj**	**log2 (FC)**	**P-adj**	**log2 (FC)**	**P-adj**
GAP43	ENSG00000172020	0.56932027	0.01638439						
IFI44L	ENSG00000137959	0.53071371	0.01765909						
EMP3	ENSG00000142227	0.54846336	0.01775355						
CCND1	ENSG00000110092	0.57298921	0.01781836						
CAPN2	ENSG00000162909	0.4744627	0.01781836						
HMGCR	ENSG00000113161	0.49030065	0.01927476						
NUAK2	ENSG00000163545	0.68105413	0.01942205						
CLU	ENSG00000120885	0.6303335	0.01955196						
SYNJ2	ENSG00000078269	0.48795191	0.02017573						
B2M	ENSG00000166710	0.49841781	0.0205488						
NPTXR	ENSG00000221890	0.68246663	0.02148048						
	ENSG00000161570	0.62085394	0.02169823						
IGFL2-AS1	ENSG00000268621	0.50219115	0.02239424			1.14570488	0.00343724		
VCAN	ENSG00000038427	0.60622982	0.02287168						
SECTM1	ENSG00000141574	0.64588127	0.02325978	1.19179889	0.0348338				
LPIN3	ENSG00000132793	0.64985419	0.0245956						
ZNF365	ENSG00000138311	0.66629662	0.02627221						
TAGLN2	ENSG00000158710	0.58467132	0.02659968						
NRP2	ENSG00000118257	0.59061802	0.02694916						
LIF	ENSG00000128342	0.63116172	0.02784079	0.86593604	0.02381964				
NPPB	ENSG00000120937	0.60173613	0.02936969						
FRMD5	ENSG00000171877	0.59194674	0.03060976						
	ENSG00000182319	0.671118	0.03146963	0.95662372	0.04607184				
PSME2	ENSG00000100911	0.50488235	0.03212298						
EHD2	ENSG00000024422	0.55108656	0.0340959						
MFAP5	ENSG00000197614	0.51507698	0.03582538						
PLAT	ENSG00000104368	0.48440863	0.03680439						
TNFRSF19	ENSG00000127863	0.57385722	0.03915552						
ITPR3	ENSG00000096433	0.64085894	0.03960393						
APOBEC3G	ENSG00000239713	0.48350907	0.03976636						
CXCL10	ENSG00000169245	0.52195442	0.03980591						
MYL9	ENSG00000101335	0.52074717	0.04029914						
DDX60L	ENSG00000181381	0.48877372	0.04052636			0.99751503	0.02288591		
RAB17	ENSG00000124839	0.49989898	0.04065787						
PMP22	ENSG00000109099	0.50824341	0.04072556						
C6orf141	ENSG00000197261	0.61798104	0.04155142						
CEACAM1	ENSG00000079385	0.48724755	0.04226572						
SP140L	ENSG00000185404	0.57534723	0.04226572						
CACNG8	ENSG00000142408	0.65026962	0.04317152						
TLR3	ENSG00000164342	0.60449257	0.04320015						
SLC12A3	ENSG00000070915	0.44802949	0.04368963						
LRRC61	ENSG00000127399	0.6321529	0.04368963						
EHD4	ENSG00000103966	0.57333792	0.04382724						
GBP4	ENSG00000162654	0.57714184	0.04382724						
SLC2A12	ENSG00000146411	0.59194058	0.04393219						
NEAT1	ENSG00000245532	0.46499223	0.04393219						
IRF1	ENSG00000125347	0.63937958	0.04461211						
GJD3	ENSG00000183153	0.45600509	0.04544128						
NR3C1	ENSG00000113580	0.64089544	0.04636798						
TINAGL1	ENSG00000142910	0.45421395	0.0465399						

Table S2. Genes affected by expression of SWI/SNF ATPase subunits at gene expression level (DESeq2)

		BRG1-wt		BRG1-mut		BRM-wt		BRM-mut	
	Number of genes affected ->	255		658		101		2	
**Name**	**GeneID**	**log2 (FC)**	**P-adj**	**log2 (FC)**	**P-adj**	**log2 (FC)**	**P-adj**	**log2 (FC)**	**P-adj**
GPRC5A	ENSG00000013588	0.63785931	0.04654439						
TOM1L2	ENSG00000175662	0.51048949	0.04665244						
SAT1	ENSG00000130066	0.48607388	0.04829538						
DCBLD2	ENSG00000057019	0.51439079	0.04859306						
CAVIN1	ENSG00000177469	0.46593688	0.04926057						
SREBF1	ENSG00000072310			1.21814726	0.00207417				
PPDPF	ENSG00000125534			1.48062837	0.00690329				
ID1	ENSG00000125968			1.13973512	0.00690329				
COL6A2	ENSG00000142173			1.42666301	0.00690329				
MLST8	ENSG00000167965			1.36272093	0.00690329				
MZT2B	ENSG00000152082			1.60528698	0.00790096				
MPG	ENSG00000103152			1.47767527	0.00815618				
CORO1B	ENSG00000172725			1.45055591	0.00815618				
ATP5F1D	ENSG00000099624			1.46883548	0.00861935				
TTYH3	ENSG00000136295			1.24369219	0.00861935				
LTBP4	ENSG00000090006			1.34167323	0.00910958				
PGGHG	ENSG00000142102			1.22824963	0.00910958				
SLC25A10	ENSG00000183048			1.51058748	0.00910958				
KLHL17	ENSG00000187961			1.40676819	0.00910958				
GPC1	ENSG00000063660			1.36472926	0.01032239				
TNRC18	ENSG00000182095			1.1054077	0.01032239				
GPAA1	ENSG00000197858			1.3242583	0.01032239				
GADD45B	ENSG00000099860			1.08923446	0.01109345				
SELENOO	ENSG00000073169			1.21615421	0.01192806				
CTBP1	ENSG00000159692			1.13930135	0.0124038				
PIGQ	ENSG00000007541			1.31932474	0.01322917				
HMG20B	ENSG00000064961			1.11715244	0.01322917				
ZDHHC8	ENSG00000099904			1.34312206	0.01322917				
ARSA	ENSG00000100299			1.24899495	0.01322917				
NDUFS7	ENSG00000115286			1.23455075	0.01322917				
HELZ2	ENSG00000130589			1.24121624	0.01322917	1.12033819	0.01568673		
LAMA5	ENSG00000130702			1.36602139	0.01322917				
BTBD2	ENSG00000133243			1.34320641	0.01322917				
TMC6	ENSG00000141524			1.27820268	0.01322917				
VPS28	ENSG00000160948			1.04174611	0.01322917				
TP53I13	ENSG00000167543			1.22056545	0.01322917				
E4F1	ENSG00000167967			1.2630677	0.01322917				
SCAND1	ENSG00000171222			1.54005793	0.01322917				
RRS1	ENSG00000179041			1.00749843	0.01322917				
DPM3	ENSG00000179085			1.47731445	0.01322917				
SCRIB	ENSG00000180900			1.40314756	0.01322917				
	ENSG00000185928			1.25200418	0.01322917				
EMID1	ENSG00000186998			1.31033842	0.01322917				
ATAD3A	ENSG00000197785			1.31284179	0.01322917				
OXLD1	ENSG00000204237			0.98714872	0.01322917				
TSPAN4	ENSG00000214063			1.34853147	0.01322917				
SLC9A3-AS1	ENSG00000225138			1.29969482	0.01322917				
RPL35P5	ENSG00000225573			1.01386983	0.01322917				
PITX1	ENSG00000069011			1.30017557	0.01419922				
NUBP2	ENSG00000095906			1.3625842	0.01419922				

Table S2. Genes affected by expression of SWI/SNF ATPase subunits at gene expression level (DESeq2)

		BRG1-wt		BRG1-mut		BRM-wt		BRM-mut	
	Number of genes affected ->	255		658		101		2	
**Name**	**GeneID**	**log2 (FC)**	**P-adj**	**log2 (FC)**	**P-adj**	**log2 (FC)**	**P-adj**	**log2 (FC)**	**P-adj**
ECI1	ENSG00000167969			1.15562074	0.01419922				
FSCN1	ENSG00000075618			1.28964853	0.01455295				
BRF1	ENSG00000185024			0.9934869	0.01455295				
CACTIN	ENSG00000105298			1.26073908	0.01571656				
PDZD4	ENSG00000067840			1.02302307	0.01596853				
RAI1	ENSG00000108557			1.18737704	0.01596853				
FBXO2	ENSG00000116661			1.07165323	0.01596853				
SLC66A2	ENSG00000122490			1.2854791	0.01596853				
JOSD2	ENSG00000161677			1.44480455	0.01596853				
MFSD3	ENSG00000167700			1.37691808	0.01596853				
IRF2BP1	ENSG00000170604			1.13772539	0.01596853				
PLXNB2	ENSG00000196576			1.17875532	0.01596853				
FAAP20	ENSG00000162585			0.88802673	0.01604818				
KIFC2	ENSG00000167702			1.05313453	0.01651961				
LMF2	ENSG00000100258			1.35620345	0.01653008				
AGRN	ENSG00000188157			1.22903517	0.01653008				
CIC	ENSG00000079432			1.20924398	0.01688906				
RPL36	ENSG00000130255			1.2655665	0.01688906				
SLC9A3R2	ENSG00000065054			1.39059458	0.0173469				
C12orf57	ENSG00000111678			1.18491207	0.0173469				
WDR34	ENSG00000119333			1.04443361	0.0173469				
NR1H2	ENSG00000131408			1.23711626	0.0173469				
ARHGDIA	ENSG00000141522			1.20299856	0.0173469				
CBS	ENSG00000160200			1.01365658	0.0173469				
SEMA6B	ENSG00000167680			1.18583698	0.0173469				
GTPBP6	ENSG00000178605			1.10871192	0.0173469				
COL18A1	ENSG00000182871			1.25965516	0.0173469				
LLGL2	ENSG00000073350			1.16680558	0.01878446				
TICAM1	ENSG00000127666			1.25484908	0.01878446				
H1-10	ENSG00000184897			1.28137892	0.01878446				
FAM83H	ENSG00000180921			0.84807781	0.01893281				
PKD1	ENSG00000008710			1.2962528	0.01915793				
ARHGEF18	ENSG00000104880			1.02611912	0.01915793				
DBN1	ENSG00000113758			1.07105694	0.01915793				
SLC2A4RG	ENSG00000125520			1.1978553	0.01915793				
CEP131	ENSG00000141577			1.2470976	0.01915793				
MTA1	ENSG00000182979			1.01338969	0.01915793				
ZNF358	ENSG00000198816			1.37459901	0.01915793				
SMOX	ENSG00000088826			1.07123376	0.01921132				
TSPO	ENSG00000100300			1.31619362	0.01921132				
BRAT1	ENSG00000106009			1.21259749	0.01921132				
NPDC1	ENSG00000107281			1.25472054	0.01921132				
APOE	ENSG00000130203			1.3610872	0.01921132				
SSBP4	ENSG00000130511			1.30348396	0.01921132				
CSNK1G2	ENSG00000133275			1.22474942	0.01921132				
ZNF668	ENSG00000167394			1.05797585	0.01921132				
SLC25A6	ENSG00000169100			1.11926405	0.01921132				
DUS1L	ENSG00000169718			1.06459396	0.01921132				
NCOR2	ENSG00000196498			1.08557346	0.01921132				
CACNA1H	ENSG00000196557			1.21672157	0.01921132				

Table S2. Genes affected by expression of SWI/SNF ATPase subunits at gene expression level (DESeq2)

		BRG1-wt		BRG1-mut		BRM-wt		BRM-mut	
	Number of genes affected ->	255		658		101		2	
**Name**	**GeneID**	**log2 (FC)**	**P-adj**	**log2 (FC)**	**P-adj**	**log2 (FC)**	**P-adj**	**log2 (FC)**	**P-adj**
INF2	ENSG00000203485			1.2308239	0.01921132				
MCRIP1	ENSG00000225663			1.43962381	0.01921132				
ASMTL	ENSG00000169093			0.8839767	0.01960999				
RPUSD1	ENSG00000007376			1.3150487	0.02049448				
WDR18	ENSG00000065268			1.13315227	0.02049448				
PALM	ENSG00000099864			1.19126328	0.02049448				
NME3	ENSG00000103024			1.37002131	0.02049448				
MIIP	ENSG00000116691			1.15399958	0.02049448				
RHOT2	ENSG00000140983			1.18917231	0.02049448				
TRABD	ENSG00000170638			1.26641406	0.02049448				
CAPN10	ENSG00000142330			1.07606627	0.02060819				
CKB	ENSG00000166165			1.17216807	0.02070686				
AGPAT2	ENSG00000169692			1.22854386	0.02080935				
FAAP100	ENSG00000185504			1.17884102	0.02080935				
DOT1L	ENSG00000104885			1.0729893	0.02100837				
0	ENSG00000256663			1.12652072	0.02116526				
ASPHD1	ENSG00000174939			1.26734468	0.02125284				
RAB11B	ENSG00000185236			1.05843023	0.02138384				
MRPL41	ENSG00000182154			1.31819878	0.0223016				
DVL1	ENSG00000107404			1.30345442	0.02232635				
TEDC1	ENSG00000185347			1.24280195	0.02232635				
RPLP1	ENSG00000137818			1.12843248	0.02299022				
RNH1	ENSG00000023191			1.14863137	0.02309792				
SCAF1	ENSG00000126461			1.17894887	0.02309792				
THEM6	ENSG00000130193			1.17500697	0.02309792				
SLC19A1	ENSG00000173638			1.07396005	0.02317798				
CTU2	ENSG00000174177			1.15102539	0.02317798				
SIVA1	ENSG00000184990			0.99171455	0.02331516				
H2AX	ENSG00000188486			1.05045407	0.02375895				
MRC2	ENSG00000011028			1.07801175	0.02386919				
PHRF1	ENSG00000070047			1.07092994	0.02386919				
COL26A1	ENSG00000160963			0.94099336	0.02386919				
INTS1	ENSG00000164880			1.21345757	0.02386919				
PUS1	ENSG00000177192			0.901004	0.02386919				
CCDC106	ENSG00000173581			1.08014703	0.02407102				
SBF1	ENSG00000100241			1.20280395	0.02412051				
NOP53	ENSG00000105373			1.06821824	0.02417028				
C7orf50	ENSG00000146540			1.18244666	0.02417028				
JPH3	ENSG00000154118			0.79762639	0.02417028				
RAC3	ENSG00000169750			1.15709559	0.02417028				
RPS2P46	ENSG00000189343			1.08508642	0.02417028				
AMH	ENSG00000104899			1.28910022	0.02421183				
ROMO1	ENSG00000125995			1.20526397	0.02421183				
IGFBP2	ENSG00000115457			1.07774306	0.02456532				
ISYNA1	ENSG00000105655			1.10946905	0.02466184				
PGAP6	ENSG00000129925			1.14182578	0.02466184				
	ENSG00000130489			1.13551252	0.02466184				
IMPA2	ENSG00000141401			0.8577894	0.02466184				
COL6A1	ENSG00000142156			1.19819546	0.02466184				
UBXN6	ENSG00000167671			1.03374379	0.02466184				

Table S2. Genes affected by expression of SWI/SNF ATPase subunits at gene expression level (DESeq2)

		BRG1-wt		BRG1-mut		BRM-wt		BRM-mut	
	Number of genes affected ->	255		658		101		2	
**Name**	**GeneID**	**log2 (FC)**	**P-adj**	**log2 (FC)**	**P-adj**	**log2 (FC)**	**P-adj**	**log2 (FC)**	**P-adj**
PACS2	ENSG00000179364			1.02870973	0.02466184				
TMEM179	ENSG00000258986			1.17411388	0.02466184				
BAD	ENSG00000002330			1.16260233	0.02471334				
BCAR1	ENSG00000050820			1.12337005	0.02471334				
RNF126	ENSG00000070423			1.21232865	0.02471334				
CAPN15	ENSG00000103326			1.18008493	0.02471334				
SGTA	ENSG00000104969			1.03150124	0.02471334				
ILVBL	ENSG00000105135			1.0054137	0.02471334				
FZR1	ENSG00000105325			1.1107272	0.02471334				
MYH14	ENSG00000105357			0.88594373	0.02471334				
CCDC92	ENSG00000119242			0.66511572	0.02471334				
LRP3	ENSG00000130881			1.28826512	0.02471334				
EPHA2	ENSG00000142627			1.0030296	0.02471334				
ATP13A2	ENSG00000159363			1.10805718	0.02471334				
HDGFL2	ENSG00000167674			0.95144136	0.02471334				
GAS2L1	ENSG00000185340			1.10026874	0.02471334				
BCAM	ENSG00000187244			0.92489416	0.02471334				
SAMD11	ENSG00000187634			1.16846646	0.02471334				
QTRT1	ENSG00000213339			0.94567782	0.02471334				
FNDC10	ENSG00000228594			1.29616719	0.02471334				
PHPT1	ENSG00000054148			1.12989204	0.02474813				
MRPL28	ENSG00000086504			0.98669244	0.02474813				
VPS51	ENSG00000149823			0.99075827	0.02474813				
FBXW5	ENSG00000159069			1.18926558	0.02474813				
0	ENSG00000231686			1.32333357	0.02474813				
ARPC1B	ENSG00000130429			1.00465032	0.02486799				
TMEM259	ENSG00000182087			1.14683531	0.02486799				
ZNF512B	ENSG00000196700			0.9918311	0.02486799				
SHANK3	ENSG00000251322			1.10875993	0.02486799				
ADRM1	ENSG00000130706			1.02432897	0.02493278				
HEXD	ENSG00000169660			0.95557058	0.02522779				
CRIP2	ENSG00000182809			1.30766834	0.02522779				
OSBPL5	ENSG00000021762			1.06060886	0.02573325				
PBXIP1	ENSG00000163346			0.72352434	0.02573325				
CCDC124	ENSG00000007080			1.21950837	0.02589784				
ASPSCR1	ENSG00000169696			1.07277838	0.02602194				
ZGPAT	ENSG00000197114			1.05711088	0.02602194				
RPL18AP3	ENSG00000213442			1.05004094	0.02602194				
	ENSG00000267896			1.22886879	0.02602194				
SEMA3B	ENSG00000012171			1.05457441	0.02611163				
CPSF1	ENSG00000071894			1.10863086	0.02611455				
ARFRP1	ENSG00000101246			0.88423671	0.02611455				
BCL7C	ENSG00000099385			0.90241884	0.02633023				
SLC7A5	ENSG00000103257			0.90881752	0.02633023				
NOTCH3	ENSG00000074181			0.9483874	0.02648307				
EPN1	ENSG00000063245			1.07464175	0.02657736				
MAPK12	ENSG00000188130			1.01938243	0.02657736				
WIZ	ENSG00000011451			1.03128391	0.02677352				
POLRMT	ENSG00000099821			1.10223247	0.02677352				
ZNF646	ENSG00000167395			0.77442002	0.02677352				

Table S2. Genes affected by expression of SWI/SNF ATPase subunits at gene expression level (DESeq2)

		BRG1-wt		BRG1-mut		BRM-wt		BRM-mut	
	Number of genes affected ->	255		658		101		2	
**Name**	**GeneID**	**log2 (FC)**	**P-adj**	**log2 (FC)**	**P-adj**	**log2 (FC)**	**P-adj**	**log2 (FC)**	**P-adj**
BRD1	ENSG00000100425			0.79019155	0.02689883				
NME4	ENSG00000103202			0.86239794	0.02689883				
TYK2	ENSG00000105397			0.92286862	0.02689883				
PRR12	ENSG00000126464			1.14342458	0.02689883				
CHTF18	ENSG00000127586			1.20539082	0.02689883				
TUBGCP6	ENSG00000128159			0.93994189	0.02689883				
SLC38A10	ENSG00000157637			1.04323848	0.02689883				
WDR90	ENSG00000161996			1.08240611	0.02689883				
GAA	ENSG00000171298			1.12559662	0.02689883				
NELFA	ENSG00000185049			1.04019547	0.02689883				
0	ENSG00000243679			1.31353119	0.02689883				
DDAH2	ENSG00000213722			1.16504035	0.02697578				
FCGRT	ENSG00000104870			0.98463016	0.02722868				
LRFN4	ENSG00000173621			1.28244414	0.02722868				
PNPLA6	ENSG00000032444			0.99627486	0.02768098				
CROCC	ENSG00000058453			1.12087868	0.02768098				
POLD1	ENSG00000062822			1.14306269	0.02768098				
VPS9D1	ENSG00000075399			1.16059494	0.02768098				
OLFM2	ENSG00000105088			0.97815452	0.02768098				
MRPL4	ENSG00000105364			1.04516283	0.02768098				
C19orf25	ENSG00000119559			1.10913511	0.02768098				
TRPM4	ENSG00000130529			1.00460638	0.02768098				
LLGL1	ENSG00000131899			0.98476906	0.02768098				
HSPBP1	ENSG00000133265			1.13891188	0.02768098				
RNPEPL1	ENSG00000142327			1.14591368	0.02768098				
AMDHD2	ENSG00000162066			1.13151164	0.02768098				
SLC4A2	ENSG00000164889			1.10399507	0.02768098				
POLR2L	ENSG00000177700			1.16841691	0.02768098				
NELFB	ENSG00000188986			1.00970634	0.02768098				
ACOT7	ENSG00000097021			0.99027975	0.02793919				
CACNG7	ENSG00000105605			0.87016643	0.02793919				
NDUFA11	ENSG00000174886			1.10775505	0.02793919				
MED16	ENSG00000175221			1.19624654	0.02793919				
EPHB4	ENSG00000196411			0.83491406	0.02793919				
STOML1	ENSG00000067221			1.0705696	0.02798796				
NTN1	ENSG00000065320			0.81517212	0.02848125				
TMUB1	ENSG00000164897			1.11437688	0.02848125				
SF3A2	ENSG00000104897			1.15264918	0.02865201				
ULK1	ENSG00000177169			1.07740191	0.02869778				
KCNQ2	ENSG00000075043			1.06454851	0.02901405				
GPS1	ENSG00000169727			1.01159949	0.02901405				
PPP1R14B	ENSG00000173457			1.10602887	0.02901405				
MAMDC4	ENSG00000177943			1.23231385	0.02902956				
POLR2I	ENSG00000105258			1.02282263	0.0290328				
RECQL4	ENSG00000160957			1.13708684	0.0290328				
ACTB	ENSG00000075624			0.69663606	0.02919595				
PAFAH1B3	ENSG00000079462			0.94866147	0.02919595				
COMT	ENSG00000093010			1.06900457	0.02919595				
PLXNA1	ENSG00000114554			0.97614257	0.02919595				
PFKL	ENSG00000141959			1.15397874	0.02919595				

Table S2. Genes affected by expression of SWI/SNF ATPase subunits at gene expression level (DESeq2)

		BRG1-wt		BRG1-mut		BRM-wt		BRM-mut	
	Number of genes affected ->	255		658		101		2	
**Name**	**GeneID**	**log2 (FC)**	**P-adj**	**log2 (FC)**	**P-adj**	**log2 (FC)**	**P-adj**	**log2 (FC)**	**P-adj**
ZDHHC12	ENSG00000160446			1.21522892	0.02919595				
BAK1P1	ENSG00000175730			1.01430006	0.02919595				
RPLP2	ENSG00000177600			0.94354476	0.02919595				
D2HGDH	ENSG00000180902			1.11279843	0.02919797				
PCNX3	ENSG00000197136			1.02837637	0.02919797				
BCAS4	ENSG00000124243			0.78511135	0.02932022				
PLPPR3	ENSG00000129951			1.24912961	0.02942554				
ROGDI	ENSG00000067836			0.83793396	0.02947531				
C20orf27	ENSG00000101220			1.10151039	0.02947531				
MAP3K10	ENSG00000130758			0.99542457	0.02947531				
C21orf58	ENSG00000160298			0.65015826	0.02947531				
PLEKHH3	ENSG00000068137			1.15235805	0.02948861				
UNC5A	ENSG00000113763			1.24114469	0.02948861				
RPS15	ENSG00000115268			1.06239058	0.02948861				
VPS37D	ENSG00000176428			1.25505591	0.02948861				
RABEP2	ENSG00000177548			1.10597599	0.02948861				
CPTP	ENSG00000224051			1.13589257	0.02948861				
TRMT61A	ENSG00000166166			1.14826105	0.02949069				
KCNH2	ENSG00000055118			1.01083063	0.02957746				
KLF16	ENSG00000129911			1.04941146	0.02957746				
EEF2	ENSG00000167658			1.02152008	0.02957746				
DRAP1	ENSG00000175550			1.1089065	0.02957746				
TECPR1	ENSG00000205356			1.03961994	0.02957746				
CLUH	ENSG00000132361			1.04581524	0.02960728				
FASTK	ENSG00000164896			0.89817387	0.02960728				
RABL6	ENSG00000196642			0.89047523	0.02964731				
GRAMD1A	ENSG00000089351			0.81023196	0.02985634				
SPSB2	ENSG00000111671			1.16910959	0.02985634				
NACC1	ENSG00000160877			1.01316197	0.02985634				
DIPK1B	ENSG00000165716			1.03741772	0.02985634				
DECR2	ENSG00000242612			0.9082013	0.02985634				
MRPS34	ENSG00000074071			0.93952384	0.03015426				
MICALL2	ENSG00000164877			1.15251173	0.03015426				
SEMA4B	ENSG00000185033			0.8359822	0.03039433				
SLC66A1	ENSG00000040487			0.78993301	0.03076998				
PIEZO1	ENSG00000103335			0.90739552	0.03094301				
WDR81	ENSG00000167716			1.12363213	0.03094301				
VWA1	ENSG00000179403			1.22073514	0.03094301				
SLC52A2	ENSG00000185803			1.16081175	0.03094301				
NDOR1	ENSG00000188566			1.1063702	0.03094301				
HCFC1R1	ENSG00000103145			0.97074503	0.03095747				
B3GAT3	ENSG00000149541			1.05690475	0.03095747				
NFKBIL1	ENSG00000204498			0.97220907	0.03095747				
SPPL2B	ENSG00000005206			1.03678683	0.03103887				
CTSD	ENSG00000117984			1.14713133	0.03109779				
LMNB2	ENSG00000176619			0.85652848	0.03109779				
PAQR4	ENSG00000162073			0.79340486	0.03110394				
PACSIN3	ENSG00000165912			1.03707983	0.03110394				
ANAPC2	ENSG00000176248			0.97722132	0.03110394				
0	ENSG00000230715			1.24633028	0.03110394				

Table S2. Genes affected by expression of SWI/SNF ATPase subunits at gene expression level (DESeq2)

		BRG1-wt		BRG1-mut		BRM-wt		BRM-mut	
	Number of genes affected ->	255		658		101		2	
**Name**	**GeneID**	**log2 (FC)**	**P-adj**	**log2 (FC)**	**P-adj**	**log2 (FC)**	**P-adj**	**log2 (FC)**	**P-adj**
TP73	ENSG00000078900			0.96683873	0.03112217				
NCLN	ENSG00000125912			1.08655451	0.03112217				
ATG4D	ENSG00000130734			1.10273295	0.03112217				
CASKIN2	ENSG00000177303			1.10040485	0.03112217				
LRP5	ENSG00000162337			1.09961608	0.03113684				
RPTOR	ENSG00000141564			0.83710136	0.03120346				
ALG12	ENSG00000182858			0.9825225	0.03120346				
EVI5L	ENSG00000142459			0.99261196	0.03160801				
RPL29	ENSG00000162244			0.95643706	0.03160801				
SMTN	ENSG00000183963			0.86881474	0.03160801				
0	ENSG00000218175			0.93926276	0.03160801				
UBE2SP2	ENSG00000224126			1.13587767	0.03160801				
MAPK8IP2	ENSG00000008735			0.88672703	0.03177154				
SHARPIN	ENSG00000179526			1.10637301	0.03179579				
ISOC2	ENSG00000063241			1.06626779	0.03184225				
TELO2	ENSG00000100726			1.19549398	0.03184225				
AHDC1	ENSG00000126705			1.08692107	0.03184225				
OGFR	ENSG00000060491			0.99223369	0.03193383				
NTHL1	ENSG00000065057			1.13342435	0.03193383				
ARVCF	ENSG00000099889			1.09028117	0.03207764				
TMEM175	ENSG00000127419			1.01924724	0.03207764				
GSN	ENSG00000148180			0.8777176	0.03207764				
GPSM1	ENSG00000160360			1.16006304	0.03207764				
TONSL	ENSG00000160949			1.09287142	0.03207764				
BRSK1	ENSG00000160469			0.95830185	0.03209621				
JUNB	ENSG00000171223			1.24268055	0.03209621				
NDUFS8	ENSG00000110717			1.01101489	0.03230276				
DDX54	ENSG00000123064			0.98896044	0.03230276				
RRAS	ENSG00000126458			1.11849995	0.03230276				
GLIS2	ENSG00000126603			1.15703598	0.03230276				
CTIF	ENSG00000134030			0.67933156	0.03230276				
TMEM201	ENSG00000188807			0.99934226	0.03230276				
TMEM250	ENSG00000238227			0.89647656	0.03230276				
GRN	ENSG00000030582			1.00145396	0.03236959				
MNT	ENSG00000070444			0.83010979	0.03236959				
REXO1	ENSG00000079313			1.04148001	0.03236959				
ZNF213	ENSG00000085644			1.04752191	0.03236959				
TGM1	ENSG00000092295			1.19027001	0.03236959				
ELOB	ENSG00000103363			1.06337375	0.03236959				
TMEM59L	ENSG00000105696			1.06888841	0.03236959				
PLEKHM2	ENSG00000116786			0.82487426	0.03236959				
DOHH	ENSG00000129932			1.22062572	0.03236959				
ABHD17A	ENSG00000129968			1.05984585	0.03236959				
ACAP3	ENSG00000131584			1.08005762	0.03236959				
FBXW9	ENSG00000132004			1.07901011	0.03236959				
RFX1	ENSG00000132005			1.1250606	0.03236959				
AGAP2	ENSG00000135439			0.92039852	0.03236959				
SH3GL1	ENSG00000141985			1.07350105	0.03236959				
RHPN1	ENSG00000158106			1.19765647	0.03236959				
ATAD3B	ENSG00000160072			0.96809987	0.03236959				

Table S2. Genes affected by expression of SWI/SNF ATPase subunits at gene expression level (DESeq2)

		BRG1-wt		BRG1-mut		BRM-wt		BRM-mut	
	Number of genes affected ->	255		658		101		2	
**Name**	**GeneID**	**log2 (FC)**	**P-adj**	**log2 (FC)**	**P-adj**	**log2 (FC)**	**P-adj**	**log2 (FC)**	**P-adj**
EMC10	ENSG00000161671			0.88198655	0.03236959				
NDUFV1	ENSG00000167792			0.93448842	0.03236959				
SSNA1	ENSG00000176101			1.14052958	0.03236959				
CRACR2B	ENSG00000177685			1.2553217	0.03236959				
FLNA	ENSG00000196924			0.97935498	0.03236959				
PIM3	ENSG00000198355			0.96870642	0.03236959				
SELENOM	ENSG00000198832			1.07871576	0.03236959				
MXD3	ENSG00000213347			1.14192121	0.03236959				
INAFM1	ENSG00000257704			1.07862847	0.03236959				
0	ENSG00000266962			0.96387172	0.03236959				
RHBDL1	ENSG00000103269			1.24711412	0.03238562				
SLC27A1	ENSG00000130304			0.93975465	0.03240173				
MTSS2	ENSG00000132613			0.9590897	0.03261224				
EME2	ENSG00000197774			0.89330409	0.03291922				
TMEM189	ENSG00000240849			0.72561227	0.03291922				
TLE2	ENSG00000065717			0.98136637	0.03389689				
ARHGEF1	ENSG00000076928			1.03974283	0.03389689				
POLR2E	ENSG00000099817			1.01688603	0.03389689				
ARFGAP1	ENSG00000101199			0.93555101	0.03389689				
ECH1	ENSG00000104823			1.0977237	0.03389689				
GALK1	ENSG00000108479			1.15562138	0.03389689				
PITPNM1	ENSG00000110697			1.0856699	0.03389689				
STK11	ENSG00000118046			0.91713214	0.03389689				
COQ8B	ENSG00000123815			0.82660895	0.03389689				
PPP1R12C	ENSG00000125503			1.09361777	0.03389689				
HIP1R	ENSG00000130787			0.97646595	0.03389689				
FOXP4	ENSG00000137166			1.00130995	0.03389689				
SLC29A4	ENSG00000164638			1.07832008	0.03389689				
SAC3D1	ENSG00000168061			1.05779986	0.03389689				
PC	ENSG00000173599			1.10282671	0.03389689				
RAD23A	ENSG00000179262			0.83797157	0.03389689				
PTP4A3	ENSG00000184489			1.1782309	0.03389689				
TPM2	ENSG00000198467			0.66363144	0.03389689				
NUDT1	ENSG00000106268			0.92909849	0.03419322				
MIF	ENSG00000240972			1.24447235	0.0345154				
PAXX	ENSG00000148362			1.11899739	0.03472462				
HAGHL	ENSG00000103253			1.17815725	0.03473688				
GIPC1	ENSG00000123159			0.97231428	0.03473688				
TSNARE1	ENSG00000171045			1.11012977	0.03473688				
CTDP1	ENSG00000060069			0.89644033	0.03479356				
CHERP	ENSG00000085872			0.92964757	0.03479356				
ABCA2	ENSG00000107331			1.0145389	0.03479356				
HPCAL1	ENSG00000115756			0.98555814	0.03479356				
YIPF2	ENSG00000130733			0.9891067	0.03479356				
0	ENSG00000218426			0.79134614	0.03479356				
FLYWCH1	ENSG00000059122			0.91872478	0.0348338				
DMPK	ENSG00000104936			0.9973872	0.0348338				
SURF6	ENSG00000148296			0.7167135	0.0348338				
YDJC	ENSG00000161179			0.89869077	0.0348338				
BSG	ENSG00000172270			1.05192164	0.0348338				

Table S2. Genes affected by expression of SWI/SNF ATPase subunits at gene expression level (DESeq2)

		BRG1-wt		BRG1-mut		BRM-wt		BRM-mut	
	Number of genes affected ->	255		658		101		2	
**Name**	**GeneID**	**log2 (FC)**	**P-adj**	**log2 (FC)**	**P-adj**	**log2 (FC)**	**P-adj**	**log2 (FC)**	**P-adj**
AURKAIP1	ENSG00000175756			1.05705447	0.0348338				
CD151	ENSG00000177697			0.92710938	0.0348338				
TBL3	ENSG00000183751			1.06991863	0.0348338				
HSF1	ENSG00000185122			0.97785824	0.0348338				
SBK1	ENSG00000188322			0.87800698	0.0348338				
ARL2	ENSG00000213465			0.998173	0.0348338				
SCAMP4	ENSG00000227500			1.08569253	0.0348338				
VPS9D1-AS1	ENSG00000261373			0.96577115	0.0348338				
DNPH1	ENSG00000112667			1.02918341	0.03508554				
SOX12	ENSG00000177732			0.77768168	0.03510653				
CEP170B	ENSG00000099814			1.04085647	0.03511276				
PIN1	ENSG00000127445			0.88030439	0.03513726				
DCAF15	ENSG00000132017			0.88852754	0.03513726				
CUEDC1	ENSG00000180891			0.76098181	0.03513726				
UCKL1	ENSG00000198276			0.88514975	0.03513726				
ZBTB12	ENSG00000204366			1.01477249	0.03513726				
WDR24	ENSG00000127580			1.06046328	0.03518804				
0	ENSG00000237214			1.06329493	0.03518804				
STRN4	ENSG00000090372			0.92605459	0.03523725				
CIAO3	ENSG00000103245			0.95713678	0.03523725				
GNB2	ENSG00000172354			0.98398976	0.03523725				
APRT	ENSG00000198931			1.02445875	0.03523725				
SGSM3	ENSG00000100359			0.9138506	0.03535143				
C1orf159	ENSG00000131591			1.11083546	0.03535143				
PLCD3	ENSG00000161714			0.95830126	0.03535143				
ATP5ME	ENSG00000169020			0.92464248	0.03535143				
FMNL1	ENSG00000184922			0.94221812	0.03535143				
TNK2	ENSG00000061938			1.00301173	0.03550213				
SBNO2	ENSG00000064932			1.0502634	0.03550213				
CBARP	ENSG00000099625			1.19709485	0.03550213				
GAS6	ENSG00000183087			1.12308656	0.03550213				
NUDT14	ENSG00000183828			1.09548463	0.03550213				
0	ENSG00000229119			0.83833396	0.03550213				
RPL13AP5	ENSG00000236552			0.93368836	0.03550213				
LSR	ENSG00000105699			1.05556829	0.03557513				
EXD3	ENSG00000187609			1.07051934	0.03557513				
TWF2	ENSG00000247596			1.20003973	0.03557513				
RHBDF2	ENSG00000129667			1.01599488	0.03569034				
FBLN1	ENSG00000077942			0.86446803	0.03583581				
THAP7	ENSG00000184436			1.03658489	0.03583581				
AP2A1	ENSG00000196961			0.88675225	0.03583581				
C8orf82	ENSG00000213563			0.89357054	0.03583581				
NPAS1	ENSG00000130751			1.10332175	0.03597261				
HYAL2	ENSG00000068001			1.00848305	0.03609156				
COTL1	ENSG00000103187			0.63803028	0.03609156				
TMEM129	ENSG00000168936			0.93077536	0.03609156				
ADGRB2	ENSG00000121753			0.99610403	0.03609373				
FLYWCH2	ENSG00000162076			1.1318042	0.03609373				
KIF1A	ENSG00000130294			0.81618711	0.0364999				
NCDN	ENSG00000020129			0.84286284	0.03687177				

Table S2. Genes affected by expression of SWI/SNF ATPase subunits at gene expression level (DESeq2)

		BRG1-wt		BRG1-mut		BRM-wt		BRM-mut	
	Number of genes affected ->	255		658		101		2	
**Name**	**GeneID**	**log2 (FC)**	**P-adj**	**log2 (FC)**	**P-adj**	**log2 (FC)**	**P-adj**	**log2 (FC)**	**P-adj**
FAM174C	ENSG00000228300			1.16440458	0.03698002				
PRRT2	ENSG00000167371			0.78965534	0.03742448				
IGSF8	ENSG00000162729			1.07882135	0.03748403				
C19orf48	ENSG00000167747			0.93993249	0.03748403				
ARRDC1	ENSG00000197070			1.00225857	0.03748403				
FBRSL1	ENSG00000112787			0.92016656	0.03750864				
BLVRB	ENSG00000090013			1.02609001	0.03752346				
METRN	ENSG00000103260			0.84411185	0.03752346				
MAPK8IP3	ENSG00000138834			0.98312541	0.03752346				
ADAM11	ENSG00000073670			0.86880875	0.03772603				
ID3	ENSG00000117318			0.93194694	0.03772603				
SPATA2L	ENSG00000158792			1.19287043	0.03772603				
NAA10	ENSG00000102030			0.92139011	0.03776004				
WASH3P	ENSG00000185596			0.95971415	0.03776004				
LIMD2	ENSG00000136490			1.00131241	0.03791337				
JMJD4	ENSG00000081692			0.87185554	0.03853138				
IDUA	ENSG00000127415			1.16169166	0.03853138				
TXNRD2	ENSG00000184470			0.78590072	0.03853138				
ZNF511	ENSG00000198546			0.71430703	0.03853138				
ELFN1-AS1	ENSG00000236081			1.06480864	0.03853138				
THAP4	ENSG00000176946			0.97143195	0.03858479				
TM7SF2	ENSG00000149809			0.86582339	0.03859217				
WHRN	ENSG00000095397			0.94422742	0.03871412				
ARHGAP39	ENSG00000147799			0.97922203	0.03871412				
ZBTB7A	ENSG00000178951			0.74402274	0.03896995				
ABCA7	ENSG00000064687			0.93052013	0.03901083				
CHPF	ENSG00000123989			1.13840922	0.03909019				
SLC25A22	ENSG00000177542			1.10465392	0.03917108				
CYBC1	ENSG00000178927			0.71329603	0.03952579				
SNHG19	ENSG00000260260			1.13291119	0.03952579				
TESC	ENSG00000088992			0.87388968	0.03955845				
MAEA	ENSG00000090316			0.82785461	0.03955845				
RPS2P5	ENSG00000240342			0.87837667	0.03955845				
METTL26	ENSG00000130731			0.92007498	0.03957835				
CBX6	ENSG00000183741			0.61327433	0.03970477				
NT5M	ENSG00000205309			0.90674737	0.03970477				
SH3GLB2	ENSG00000148341			0.86574687	0.03977066				
BRICD5	ENSG00000182685			1.09305042	0.03977066				
NAGLU	ENSG00000108784			0.86726505	0.03983189				
ZBTB17	ENSG00000116809			1.01726779	0.03983189				
ARAP1	ENSG00000186635			0.81166445	0.04008551				
HPS6	ENSG00000166189			0.85497399	0.0401819				
CENPB	ENSG00000125817			0.75725099	0.04018677				
EEF1A2	ENSG00000101210			1.010623	0.04021935				
PTPRS	ENSG00000105426			0.76120689	0.04021935				
PRPF31	ENSG00000105618			0.66140004	0.04021935				
KCNN1	ENSG00000105642			0.94309886	0.04021935				
ENKD1	ENSG00000124074			0.99394456	0.04021935				
ANAPC11	ENSG00000141552			1.00696607	0.04021935				
CTXN1	ENSG00000178531			1.18950903	0.04021935				

Table S2. Genes affected by expression of SWI/SNF ATPase subunits at gene expression level (DESeq2)

		BRG1-wt		BRG1-mut		BRM-wt		BRM-mut	
	Number of genes affected ->	255		658		101		2	
**Name**	**GeneID**	**log2 (FC)**	**P-adj**	**log2 (FC)**	**P-adj**	**log2 (FC)**	**P-adj**	**log2 (FC)**	**P-adj**
TBCD	ENSG00000141556			0.60309902	0.04032938				
KCNH3	ENSG00000135519			1.04725985	0.04042312				
XAB2	ENSG00000076924			1.03732374	0.04044715				
PAK4	ENSG00000130669			1.04911516	0.04050803				
TRMT2A	ENSG00000099899			0.98163697	0.04079493				
ZNHIT1	ENSG00000106400			0.79970806	0.04079493				
LZTS2	ENSG00000107816			0.83697405	0.04079493				
MXD4	ENSG00000123933			0.83758109	0.04079493				
SGSM2	ENSG00000141258			0.79109849	0.04079493				
MAP3K11	ENSG00000173327			0.87324597	0.04079493				
METRNL	ENSG00000176845			1.04672037	0.04079493				
NOC2L	ENSG00000188976			0.7937162	0.04079493				
ARRDC1-AS	ENSG00000203993			0.81082352	0.04082972				
RUVBL2	ENSG00000183207			0.99714959	0.04088356				
IFT140	ENSG00000187535			0.74421221	0.04088356				
CLPP	ENSG00000125656			0.9739163	0.04093298				
GIT1	ENSG00000108262			0.88365034	0.04095711				
CACFD1	ENSG00000160325			0.9562518	0.04103225				
TEDC2	ENSG00000162062			1.00013945	0.04103225				
SSH3	ENSG00000172830			0.88767604	0.04115602				
FCHO1	ENSG00000130475			0.89408934	0.04118472				
HSPG2	ENSG00000142798			1.07008299	0.04123728				
RNF166	ENSG00000158717			0.9472517	0.04125525				
ARHGAP33	ENSG00000004777			1.03464311	0.04134471				
GADD45GIP	ENSG00000179271			1.05023264	0.04134471				
XYLT2	ENSG00000015532			0.84233511	0.04147729				
TUBB3	ENSG00000258947			0.79725992	0.04148123				
ASB6	ENSG00000148331			0.79653825	0.04164413				
MIDN	ENSG00000167470			0.79149894	0.04164413				
TMEM161A	ENSG00000064545			1.0186508	0.04191639				
TKFC	ENSG00000149476			0.69616816	0.04191639				
FAM234A	ENSG00000167930			0.94977694	0.04210728				
MICOS13	ENSG00000174917			1.0606987	0.04210728				
PLEKHJ1	ENSG00000104886			0.82683486	0.04278231				
CDT1	ENSG00000167513			1.00716392	0.04278231				
MBD3	ENSG00000071655			0.68873034	0.04294131				
CHCHD5	ENSG00000125611			1.07028874	0.04294131				
RCN3	ENSG00000142552			1.088414	0.04294131				
TERT	ENSG00000164362			0.84041804	0.04294131				
CLCN7	ENSG00000103249			1.01092413	0.04333149				
TNNT1	ENSG00000105048			0.77320249	0.04333149				
MEIS3	ENSG00000105419			0.72490335	0.04333149				
COPE	ENSG00000105669			1.02972154	0.04333149				
MCF2L	ENSG00000126217			0.95290768	0.04333149				
PUSL1	ENSG00000169972			1.02548363	0.04333149				
IRAK1	ENSG00000184216			0.8490465	0.04333149				
PEMT	ENSG00000133027			1.01771021	0.04345699				
FBRS	ENSG00000156860			0.89920872	0.04346615				
TSR3	ENSG00000007520			0.99029636	0.04347014				
ATP6V0E2	ENSG00000171130			0.81547403	0.04348558				

Table S2. Genes affected by expression of SWI/SNF ATPase subunits at gene expression level (DESeq2)

		BRG1-wt		BRG1-mut		BRM-wt		BRM-mut	
	Number of genes affected ->	255		658		101		2	
**Name**	**GeneID**	**log2 (FC)**	**P-adj**	**log2 (FC)**	**P-adj**	**log2 (FC)**	**P-adj**	**log2 (FC)**	**P-adj**
TCF3	ENSG00000071564			0.79941754	0.04350776				
EDF1	ENSG00000107223			0.82741985	0.04350776				
PSKH1	ENSG00000159792			0.78354309	0.04350776				
CHMP6	ENSG00000176108			0.87835372	0.04350776				
KHSRP	ENSG00000088247			0.6565997	0.04363408				
ERF	ENSG00000105722			1.00218159	0.04363408				
NRGN	ENSG00000154146			1.03393077	0.04363408				
MST1P2	ENSG00000186301			0.7337032	0.04363408				
MUS81	ENSG00000172732			0.8387422	0.04363704				
SAPCD2	ENSG00000186193			0.81043776	0.04363704				
CDC42EP2	ENSG00000149798			0.94798835	0.0437037				
MMP17	ENSG00000198598			1.13936421	0.0437037				
MYBBP1A	ENSG00000132382			0.87426378	0.04394582				
PEX10	ENSG00000157911			0.64656563	0.04405345				
RPS21	ENSG00000171858			0.79680691	0.04405345				
NOC4L	ENSG00000184967			1.07358454	0.04405345				
FTLP3	ENSG00000226608			0.95196097	0.04405345				
MOSPD3	ENSG00000106330			0.94634424	0.04409323				
ARF5	ENSG00000004059			0.68218391	0.04410549				
HSD17B14	ENSG00000087076			0.92299237	0.04410549				
TUBB4A	ENSG00000104833			0.63219065	0.04425612				
FAM171A2	ENSG00000161682			1.08946667	0.04425612				
B4GALNT4	ENSG00000182272			0.96726038	0.04429748				
PTOV1-AS2	ENSG00000269352			1.06462252	0.04445968				
GRAMD4	ENSG00000075240			0.76701916	0.04468508				
PCBP4	ENSG00000090097			0.76007525	0.04468508				
TIMM13	ENSG00000099800			1.06990608	0.04468508				
PPM1F	ENSG00000100034			0.77175892	0.04468508				
EEF1D	ENSG00000104529			0.87369553	0.04468508				
PKN3	ENSG00000160447			0.91146739	0.04468508				
SPSB3	ENSG00000162032			1.09731987	0.04468508				
SEZ6L2	ENSG00000174938			0.9029381	0.04468508				
TAPBP	ENSG00000231925			0.65620879	0.04468508				
WDR13	ENSG00000101940			0.9315873	0.04474342				
KCNQ4	ENSG00000117013			0.90011208	0.04474342				
ZBTB46	ENSG00000130584			0.89689276	0.04474342				
ZNF787	ENSG00000142409			0.97785303	0.04474342				
TMEM132A	ENSG00000006118			0.82442898	0.04494457				
REEP6	ENSG00000115255			0.9104302	0.04494457				
MRPL34	ENSG00000130312			0.72234594	0.04494457				
DPYSL4	ENSG00000151640			1.02491501	0.04494457				
GPR137	ENSG00000173264			0.97389223	0.04494457				
RBM10	ENSG00000182872			0.82648998	0.04494457				
TOP1MT	ENSG00000184428			0.71600408	0.04494457				
0	ENSG00000223612			1.04337775	0.04494457				
TFPT	ENSG00000105619			0.96526528	0.0455787				
CYB5R3	ENSG00000100243			0.79907385	0.04582794				
MFSD12	ENSG00000161091			0.83887492	0.04582794				
SUN2	ENSG00000100242			0.85019142	0.04593306				
PRX	ENSG00000105227			0.71636522	0.04593306				

Table S2. Genes affected by expression of SWI/SNF ATPase subunits at gene expression level (DESeq2)

		BRG1-wt		BRG1-mut		BRM-wt		BRM-mut	
	Number of genes affected ->	255		658		101		2	
**Name**	**GeneID**	**log2 (FC)**	**P-adj**	**log2 (FC)**	**P-adj**	**log2 (FC)**	**P-adj**	**log2 (FC)**	**P-adj**
ZMIZ2	ENSG00000122515			0.88048646	0.04593306				
NBEAL2	ENSG00000160796			0.83516069	0.04593306				
TEKT4P2	ENSG00000188681			0.72070389	0.04593306				
UQCC3	ENSG00000204922			0.99985714	0.04593306				
0	ENSG00000248015			0.88824954	0.04593306				
OPRL1	ENSG00000125510			0.96329742	0.04596277				
ATP13A1	ENSG00000105726			0.8420075	0.04607184				
NHP2	ENSG00000145912			0.81276955	0.04607184				
POP7	ENSG00000172336			0.80156433	0.04607184				
PTBP1	ENSG00000011304			0.72793446	0.04607876				
SIRT6	ENSG00000077463			1.0190069	0.04607876				
NACAD	ENSG00000136274			0.95604895	0.04607876				
	ENSG00000184750			0.85308989	0.04607876				
MICAL1	ENSG00000135596			0.67979909	0.0460853				
FAM53B	ENSG00000189319			0.76041841	0.04646773				
SNAPC2	ENSG00000104976			1.00307633	0.04687985				
ZNF276	ENSG00000158805			0.64287751	0.04687985				
PPFIA3	ENSG00000177380			0.93297517	0.04717181				
NACC2	ENSG00000148411			0.57832485	0.04772047				
LRRC45	ENSG00000169683			0.91828023	0.04772047				
	ENSG00000182500			0.82306627	0.04772047				
CLIP2	ENSG00000106665			0.88871495	0.04794147				
ITPKA	ENSG00000137825			1.14872949	0.0479594				
DEAF1	ENSG00000177030			0.86690774	0.0479594				
TMCC2	ENSG00000133069			0.77181757	0.04795957				
SPHK2	ENSG00000063176			0.93446239	0.04814501				
ACOT11	ENSG00000162390			0.94991354	0.04828465				
CASKIN1	ENSG00000167971			1.09105011	0.04870184				
AP1M1	ENSG00000072958			0.79234528	0.04912387				
MAP2K2	ENSG00000126934			0.89101557	0.04912387				
GATAD2A	ENSG00000167491			0.74731911	0.04914996				
ZC3H3	ENSG00000014164			1.04426894	0.04944235				
DGCR6L	ENSG00000128185			1.03596469	0.04944235				
ADAMTS7	ENSG00000136378			1.07004988	0.04944235				
NFIC	ENSG00000141905			0.68547354	0.04944235				
MID1IP1	ENSG00000165175			0.95178262	0.04944235				
FKRP	ENSG00000181027			0.66658649	0.04944235				
TSSC4	ENSG00000184281			1.04432436	0.04948235				
SPTBN4	ENSG00000160460			0.83179448	0.04970692				
GSTP1	ENSG00000084207			0.89236022	0.04981661				
RAB11FIP3	ENSG00000090565			0.82835631	0.04981661				
GIGYF1	ENSG00000146830			0.78975697	0.04981661				
RPS6KA4	ENSG00000162302			0.7683924	0.04981661				
ARHGEF40	ENSG00000165801			0.62700697	0.04981661				
MCRIP2	ENSG00000172366			1.1110145	0.04981661				
NAA38	ENSG00000183011			1.03489674	0.04981661				
PALM3	ENSG00000187867			0.72584763	0.04981661				
RAB40C	ENSG00000197562			1.05180333	0.04981661				
SMARCA2	ENSG00000080503					2.20437254	9.8827E-12	2.10781518	1.063E-36
CNGA1	ENSG00000198515					1.36989602	0.00041189		

Table S2. Genes affected by expression of SWI/SNF ATPase subunits at gene expression level (DESeq2)

		BRG1-wt		BRG1-mut		BRM-wt		BRM-mut	
	Number of genes affected ->	255		658		101		2	
**Name**	**GeneID**	**log2 (FC)**	**P-adj**	**log2 (FC)**	**P-adj**	**log2 (FC)**	**P-adj**	**log2 (FC)**	**P-adj**
MX1	ENSG00000157601					1.11858463	0.00360621		
CMPK2	ENSG00000134326					1.10009173	0.00953094		
LINC00330	ENSG00000235097					1.09386035	0.00953094		
CHRNA9	ENSG00000174343					1.0542867	0.01121663		
PSG5	ENSG00000204941					1.00801416	0.01332378		
MX2	ENSG00000183486					0.99219808	0.01583793		
OAS2	ENSG00000111335					0.98860134	0.01637485		
CRYAB	ENSG00000109846					1.02410421	0.01708279		
AKAP14	ENSG00000186471					1.01581263	0.02002492		
SP100	ENSG00000067066					0.94466022	0.02288591		
ABCA4	ENSG00000198691					0.97672159	0.0317826		
OLR1	ENSG00000173391					1.03922472	0.03646203		

Table S3. Genes affected at both expression and alternative splicing levels.

expression affected ->	102	25	81	6	0	splicing affected ->	29	56	60	26
**GeneID**	**Name**	**BRG1-wt**	**BRG1-mut**	**BRM-wt**	**BRM-mut**		**BRG1-wt**	**BRG1-mut**	**BRM-wt**	**BRM-mut**
ENSG00000127616	SMARCA4	1	1	0	0		2	2	1	0
ENSG00000170421	KRT8	1	0	1	0		0	1	0	0
ENSG00000149591	TAGLN	1	0	0	0		0	0	1	0
ENSG00000188313	PLSCR1	1	0	1	0		1	1	1	0
ENSG00000140511	HAPLN3	1	1	1	0		0	1	0	0
ENSG00000128641	MYO1B	1	0	1	0		1	1	1	0
ENSG00000140545	MFGE8	1	1	0	0		1	0	0	0
ENSG00000059378	PARP12	1	0	1	0		0	0	1	1
ENSG00000143507	DUSP10	1	0	1	0		0	0	0	1
ENSG00000154310	TNIK	1	0	0	0		0	0	1	0
ENSG00000140416	TPM1	1	0	0	0		0	0	0	1
ENSG00000189171	S100A13	1	0	0	0		0	0	1	0
ENSG00000106785	TRIM14	1	0	0	0		0	1	1	0
ENSG00000136068	FLNB	1	0	0	0		1	1	1	0
ENSG00000145287	PLAC8	1	0	0	0		0	0	1	0
ENSG00000163531	NFASC	1	0	0	0		0	0	1	0
ENSG00000149557	FEZ1	1	0	0	0		1	0	0	0
ENSG00000147459	DOCK5	1	0	0	0		0	1	0	0
ENSG00000163947	ARHGEF3	1	0	0	0		0	1	1	0
ENSG00000147852	VLDLR	1	0	0	0		1	1	0	1
ENSG00000113161	HMGCR	1	0	0	0		0	0	0	1
ENSG00000128342	LIF	1	1	0	0		0	0	0	1
ENSG00000197614	MFAP5	1	0	0	0		0	0	1	0
ENSG00000127863	TNFRSF19	1	0	0	0		0	0	1	0
ENSG00000175662	TOM1L2	1	0	0	0		0	0	2	0
ENSG00000072310	SREBF1	0	1	0	0		1	0	2	0
ENSG00000067840	PDZD4	0	1	0	0		0	1	0	1
ENSG00000122490	PQLC1	0	1	0	0		1	0	1	0
ENSG00000161677	JOSD2	0	1	0	0		0	1	0	0
ENSG00000167700	MFSD3	0	1	0	0		0	1	1	0
ENSG00000162585	FAAP20	0	1	0	0		0	2	1	0
ENSG00000106009	BRAT1	0	1	0	0		1	1	1	1
ENSG00000133275	CSNK1G2	0	1	0	0		0	1	0	0
ENSG00000142330	CAPN10	0	1	0	0		0	1	1	0
ENSG00000185504	FAAP100	0	1	0	0		0	1	0	0
ENSG00000137818	RPLP1	0	1	0	0		1	1	0	1
ENSG00000023191	RNH1	0	1	0	0		0	1	1	1
ENSG00000002330	BAD	0	1	0	0		0	1	1	0
ENSG00000105357	MYH14	0	1	0	0		0	1	0	0
ENSG00000119242	CCDC92	0	1	0	0		1	1	1	0
ENSG00000167674	HDGFL2	0	1	0	0		1	1	0	0
ENSG00000185340	GAS2L1	0	1	0	0		1	1	1	1
ENSG00000182087	TMEM259	0	1	0	0		0	1	0	0
ENSG00000196700	ZNF512B	0	1	0	0		1	1	0	0
ENSG00000130706	ADRM1	0	1	0	0		0	0	1	0
ENSG00000169660	HEXDC	0	1	0	0		0	1	1	0
ENSG00000101246	ARFRP1	0	1	0	0		1	0	0	0
ENSG00000099385	BCL7C	0	1	0	0		0	0	1	0
ENSG00000011451	WIZ	0	1	0	0		0	1	1	1
ENSG00000100425	BRD1	0	1	0	0		0	0	1	0
ENSG00000103202	NME4	0	1	0	0		0	1	1	0
ENSG00000105397	TYK2	0	1	0	0		0	2	0	0
ENSG00000127586	CHTF18	0	1	0	0		0	0	1	0
ENSG00000213722	DDAH2	0	1	0	0		0	0	1	0
ENSG00000130529	TRPM4	0	1	0	0		0	2	0	0
ENSG00000196411	EPHB4	0	1	0	0		0	0	1	1
ENSG00000067221	STOML1	0	1	0	0		0	1	0	0
ENSG00000075624	ACTB	0	1	0	0		1	0	0	1
ENSG00000124243	BCAS4	0	1	0	0		1	1	0	1

Table S3. Genes affected at both expression and alternative splicing levels.

**expression affected ->**	**102**	**25**	**81**	**6**	**0**	**splicing affected ->**	**29**	**56**	**60**	**26**
**GeneID**	**Name**	**BRG1-wt**	**BRG1-mut**	**BRM-wt**	**BRM-mut**		**BRG1-wt**	**BRG1-mut**	**BRM-wt**	**BRM-mut**
ENSG00000067836	ROGDI	0	1	0	0		1	1	1	0
ENSG00000101220	C20orf27	0	1	0	0		0	1	0	0
ENSG00000111671	SPSB2	0	1	0	0		0	0	0	1
ENSG00000149541	B3GAT3	0	1	0	0		0	0	0	1
ENSG00000130734	ATG4D	0	1	0	0		0	1	0	0
ENSG00000162244	RPL29	0	1	0	0		0	0	1	1
ENSG00000179526	SHARPIN	0	1	0	0		0	0	0	1
ENSG00000063241	ISOC2	0	1	0	0		0	0	1	0
ENSG00000110717	NDUFS8	0	1	0	0		0	1	0	0
ENSG00000103363	ELOB	0	1	0	0		0	1	1	0
ENSG00000167792	NDUFV1	0	1	0	0		0	1	0	0
ENSG00000101199	ARFGAP1	0	1	0	0		0	0	1	0
ENSG00000123159	GIPC1	0	1	0	0		0	1	1	0
ENSG00000060069	CTDP1	0	1	0	0		0	0	1	0
ENSG00000177697	CD151	0	1	0	0		1	0	0	0
ENSG00000213465	ARL2	0	1	0	0		0	0	1	0
ENSG00000127445	PIN1	0	1	0	0		1	1	1	0
ENSG00000198276	UCKL1	0	1	0	0		1	1	1	0
ENSG00000169020	ATP5ME	0	1	0	0		1	1	1	0
ENSG00000061938	TNK2	0	1	0	0		1	1	1	1
ENSG00000168936	TMEM129	0	1	0	0		0	1	0	0
ENSG00000184470	TXNRD2	0	1	0	0		0	0	0	1
ENSG00000178927	CYBC1	0	1	0	0		1	0	0	0
ENSG00000090316	MAEA	0	1	0	0		0	0	1	0
ENSG00000130731	METTL26	0	1	0	0		1	0	0	1
ENSG00000105426	PTPRS	0	1	0	0		0	0	1	0
ENSG00000130669	PAK4	0	1	0	0		1	0	1	0
ENSG00000015532	XYLT2	0	1	0	0		0	1	0	0
ENSG00000149476	TKFC	0	1	0	0		0	0	1	0
ENSG00000164362	TERT	0	1	0	0		0	0	1	0
ENSG00000184216	IRAK1	0	1	0	0		0	2	1	1
ENSG00000171130	ATP6V0E2	0	1	0	0		0	1	0	1
ENSG00000090097	PCBP4	0	1	0	0		0	1	1	0
ENSG00000104529	EEF1D	0	1	0	0		0	2	1	0
ENSG00000231925	TAPBP	0	1	0	0		1	2	0	0
ENSG00000182872	RBM10	0	1	0	0		1	1	1	0
ENSG00000122515	ZMIZ2	0	1	0	0		0	0	1	0
ENSG00000145912	NHP2	0	1	0	0		0	0	1	0
ENSG00000077463	SIRT6	0	1	0	0		0	0	0	1
ENSG00000135596	MICAL1	0	1	0	0		0	1	0	0
ENSG00000063176	SPHK2	0	1	0	0		0	1	1	1
ENSG00000141905	NFIC	0	1	0	0		0	0	1	0
ENSG00000184281	TSSC4	0	1	0	0		0	2	1	0

Table S4. siRNA used in this study.

Target	Sequence	Reference
scrC	GUCCGAGGGGGUUGUAAUCTT	This study
SMARCA4	GAAGAAGAUUCCAGAUCCATT	This study
SMARCA2	GGAUGACGCUGAAGUAGAATT	This study
hnRNPU	GUCACUAACUACAAGUGGA	Fei T, Chen Y, Xiao T, Li W, Cato L, Zhang P, Cotter MB, Bowden M, Lis RT,Zhao SG, Wu Q, Feng FY, Loda M, He HH, Liu XS, Brown M. Genome-wide CRISPR screen identifies HNRNPL as a prostate cancer dependency regulating RNA splicing. ProcNatl Acad Sci U S A. 2017 Jun 27;114(26):E5207-E5215. doi:10.1073/pnas.1617467114. Epub 2017 Jun 13. PubMed PMID: 28611215; PubMed Central PMCID: PMC5495225.
hnRNPL	CAUCAUGCCUGGUCAGUCA	Fei T, Chen Y, Xiao T, Li W, Cato L, Zhang P, Cotter MB, Bowden M, Lis RT,Zhao SG, Wu Q, Feng FY, Loda M, He HH, Liu XS, Brown M. Genome-wide CRISPR screen identifies HNRNPL as a prostate cancer dependency regulating RNA splicing. ProcNatl Acad Sci U S A. 2017 Jun 27;114(26):E5207-E5215. doi:10.1073/pnas.1617467114. Epub 2017 Jun 13. PubMed PMID: 28611215; PubMed Central PMCID: PMC5495225.

Table S5. Primers used in this study.

Target			Strand	Sequence
ARPP0	cDNA	common	F	GACCTGGAAGTCCAACTACTTC
ARPP0	cDNA	common	R	TGAGGTCCTCCTTGGTGAACAC
BRG1	cDNA	common	F	AGGCAAAATCCAGAAGCTGA
BRG1	cDNA	common	R	CGCTTGTCCTTCTTCTGGTC
BRM	cDNA	common	F	CATCTTTGACAGCGACTGGA
BRM	cDNA	common	R	TCTGATCCACGTTCAGCTTG
GADD45A	cDNA	2 isoforms	R	CCTGGATCAGGGTGAAGTGG
GADD45A	cDNA	common	F	CCAAGGGGCTGAGTGAGTTC
GADD45A	cDNA	common	R	TCCTTCCTGCATGGTTCTTTGT
GADD45A	cDNA	iso1	F	CACTGTCGGGGTGTACGAAG
GADD45A	cDNA	iso1,2	R	GTTGATGTCGTTCTCGCAGC
GADD45A	cDNA	iso2	F	AGAAGACCGAAAGCGACCC
GADD45A	cDNA	iso3	R	GAATGTGGATTACGTTGAGCAGC
GADD45A	cDNA	iso3 / 2 isoforms	F	CTGGAGAGCAGAAGACCGAAAG
GADD45A	ChIP	ex4	F	GAATGTTCAAGTGTTTTCTCCTCA
GADD45A	ChIP	ex4	R	TGTAGCGACTTTCCCGGC
GADD45A	ChIP	promoter	F	CCAGCTCACGATTTCCCAGT
GADD45A	ChIP	promoter	R	GGATGGCAGGGAACCAAGTT
GADD45A	ChIP, ChRIP	ex2	F	CGCGTGTAGGATGGATAAGGT
GADD45A	ChIP, ChRIP	ex2	R	GCTTCGTACACCCCGACAG
MAZ	cDNA	2 isoforms	F	GCACACAGTACGACACGAGG
MAZ	cDNA	2 isoforms	R	AGGGGACTTCAACACAACCG
MAZ	cDNA	ex5	F	CGCACACAGTACGACACGA
MAZ	cDNA	ex5	R	TGCCGTGGTGAAGCCTTTGT
MAZ	cDNA	skip ex5	F	GTCACGTGTGTGGCAAGATG
MAZ	cDNA	skip ex5	R	CCAGTACCTTTGTTGCAGAGC
MAZ	ChIP	ex6	F	GTGAGCTCTCAGCCACTTCC
MAZ	ChIP	ex6	R	GAGGCTCCTTGGTTGGTAGG
MAZ	ChIP	promoter	F	TGAGGCCTGCGACCGTTAG
MAZ	ChIP	promoter	R	CAGCGGCCGTTATTTCGC
MAZ	ChIP, ChRIP	ex5	F	CTAGGAGATCAGCCCCGTCT
MAZ	ChIP, ChRIP	ex5	R	CTGCCGTGGTGAAGCCTAT
MYL6	cDNA	2 isoforms	F	GGCATGAGGACAGCAATGGT
MYL6	cDNA	2 isoforms	R	ATTTGGTGAATGCTGACGGC
MYL6	cDNA	common	F	CGAGAGTCGGAGCCATTACT
MYL6	cDNA	common	R	TCTTGCCATCACCTGTTCGG
MYL6	cDNA	ex6	F	GTCGAAGGACTTCGGGTGTT
MYL6	cDNA	ex6	R	CCGACAGGATATGCCTCACA
MYL6	ChIP	ex7	F	TTGCAGTCTGGTAGTCCCCT
MYL6	ChIP	ex7	R	GGAGACTGGGAAGGTCCTCA
MYL6	ChIP	promoter	F	CTGGAGAAAGGAACAGCGGA
MYL6	ChIP	promoter	R	TGCAGTAATGGCTCCGACTC
MYL6	ChIP, ChRIP	ex6	F	CATATCCTGTCGGGGTGACG
MYL6	ChIP, ChRIP	ex6	R	ACACTGGGGAGATCAACTAGAGG
non_transcribed_region	ChIP	chr1:68071267-68071286	F	CCAGGAAAGGACGCTTCAGT
non_transcribed_region	ChIP	chr1:68071462-68071443	R	GGGCATGTGCTGAACCAAAG

Table S6. Antobodies used in this study.

Antibody	Reference	Company
α-ARID1	ab272905	Abcam
α-ARID2	A302-230A	Bethyl Laboratories Inc
	ab245529	Abcam
α-BAF155/SMARCC1	ab725003	Abcam
α-BAF180/PBRM1	A700-019	Bethyl Laboratories Inc
α-BRD9	58906	Cell Signaling
	A303-781A	Bethyl Laboratories Inc
α-BRG1	Östlund-Farrants et al. 1997	
α-BRM	ab15597	Abcam
α-CTD	ab817	Abcam
α-CTD_Ser2-P	ab5095	Abcam
α-CTD_Ser5-P	ab5131	Abcam
α-DXH9	ab26271	Abcam
α-H3	ab1791	Abcam
α-H3K27ac	ab177178	Abcam
α-H3K36me3	ab9050-100	Abcam
α-H3K4me3	ab8580	Abcam
α-H3K9ac	ab12179	Abcam
α-hnRNPL	ab6106	Abcam
α-IgG	ab46540	Abcam
α-INI1	ab181976	Abcam
α-SAM68	ab109197	Abcam
α-SAP155	ab39578	Abcam
α-THOC2	ab129485	Abcam
